# Clinical effectiveness of manual therapy for the management of musculoskeletal and non-musculoskeletal conditions: systematic review and update of UK evidence report

**DOI:** 10.1186/2045-709X-22-12

**Published:** 2014-03-28

**Authors:** Christine Clar, Alexander Tsertsvadze, Rachel Court, Gillian Lewando Hundt, Aileen Clarke, Paul Sutcliffe

**Affiliations:** 1 Populations, Evidence and Technologies, Division of Health Sciences, Warwick Medical School, University of Warwick, Coventry CV4 7AL, England; 2 Social Science and Systems in Health, Division of Health Sciences, Warwick Medical School, University of Warwick, Coventry CV4 7AL, England

**Keywords:** Clinical effectiveness, Manual therapy, Systematic review, Musculoskeletal, Bronfort

## Abstract

**Background:**

This systematic review updated and extended the "UK evidence report" by Bronfort et al. (Chiropr Osteopath 18:3, 2010) with respect to conditions/interventions that received an 'inconclusive’ or 'negative’ evidence rating or were not covered in the report.

**Methods:**

A literature search of more than 10 general medical and specialised databases was conducted in August 2011 and updated in March 2013. Systematic reviews, primary comparative studies and qualitative studies of patients with musculoskeletal or non-musculoskeletal conditions treated with manual therapy and reporting clinical outcomes were included. Study quality was assessed using standardised instruments, studies were summarised, and the results were compared against the evidence ratings of Bronfort. These were either confirmed, updated, or new categories not assessed by Bronfort were added.

**Results:**

25,539 records were found; 178 new and additional studies were identified, of which 72 were systematic reviews, 96 were randomised controlled trials, and 10 were non-randomised primary studies. Most 'inconclusive’ or 'moderate’ evidence ratings of the UK evidence report were confirmed. Evidence ratings changed in a positive direction from inconclusive to moderate evidence ratings in only three cases (manipulation/mobilisation [with exercise] for rotator cuff disorder; spinal mobilisation for cervicogenic headache; and mobilisation for miscellaneous headache). In addition, evidence was identified on a large number of non-musculoskeletal conditions not previously considered; most of this evidence was rated as inconclusive.

**Conclusions:**

Overall, there was limited high quality evidence for the effectiveness of manual therapy. Most reviewed evidence was of low to moderate quality and inconsistent due to substantial methodological and clinical diversity. Areas requiring further research are highlighted.

## Background

Manual therapy is a non-surgical type of conservative management that includes different skilled hands/fingers-on techniques directed to the patient’s body (spine and extremities) for the purpose of assessing, diagnosing, and treating a variety of symptoms and conditions [1-4]. Manual therapy constitutes a wide variety of different techniques which may be categorised into four major groups: a) manipulation (thrust manipulation), b) mobilisation (non-thrust manipulation), c) static stretching, and d) muscle energy techniques. The definition and purpose of manual therapy varies across health care professionals.

Spinal manipulation and mobilisation are commonly used treatment modalities for back pain, particularly by physical therapists, osteopaths, and chiropractors. Back pain is an important health problem with serious societal and economic consequences for the developed world. It is estimated that in the USA 80% of people will experience back problems at some point during their lifetime [5]. Back pain is also very prevalent in the UK, affecting around 29% of the population annually [6].

The use of chiropractic, osteopathic, and other forms of services delivering various types of manual therapies has been steadily increasing in the Western World [7]. For example, in the United States, 33% of people with low back pain are treated by a chiropractor [8]. A UK-based study surveyed the prevalence of back pain and the use of chiropractic/osteopathy services in a randomly selected sample of adults aged 18–64 years living in four counties of England [9]. Of the respondents with back pain (15.6%), 13.4% had consulted with osteopaths and/or chiropractic practitioners.

One descriptive review summarised surveys reporting rates of use of complementary and alternative medicine (CAM) therapies for management of low back pain and other musculoskeletal and non-musculoskeletal conditions [10]. Results of this review showed that chiropractors were used by 6% to 12% of the surveyed population, the majority of which complained of back pain.

Previous research has shown short-term benefit of spinal manual therapy (i.e., manipulation, mobilisation) especially in reducing back pain [11-20]. There is little and mostly inconclusive evidence from randomised trials on the effectiveness of manual therapy including chiropractic manipulation for non-musculoskeletal conditions, specifically for patients with dysmenorrhoea, hypertension, chronic obstructive lung disease, asthma, infantile colic, premenstrual syndrome, otitis media, nocturnal enuresis [7,8,20].

The annual incidence of major harms or complications associated with the use of manipulative procedures is low. In general, manipulations using thrust techniques carry a greater risk of major complications than the non-thrusting, low-velocity, low-amplitude soft-tissue approaches [21]. Systematic reviews using a variety of data sources come to conflicting conclusions regarding serious adverse events that can result from spinal manipulations, especially cervical manipulations (including stroke and death) [22-29].

The current review builds on the "UK evidence report" by Bronfort, Haas, Evans, Leininger and Triano [20] on the effectiveness of manual therapies commissioned by the UK General Chiropractic Council (GCC). The UK evidence report concluded that spinal manipulation/mobilisation was effective in adults for: acute, sub-acute, and chronic low back pain; migraine and cervicogenic headache; cervicogenic dizziness; manipulation/mobilisation was effective for several extremity joint conditions; and thoracic manipulation/mobilisation was effective for acute/sub-acute neck pain. The evidence was inconclusive for cervical manipulation/mobilisation alone for neck pain of any duration, and for manipulation/mobilisation for mid back pain, sciatica, tension-type headache, coccydynia, temporomandibular joint disorders, fibromyalgia, premenstrual syndrome, and pneumonia in older adults. Spinal manipulation was not effective for asthma and dysmenorrhoea when compared to sham manipulation, or for stage 1 hypertension when added to an antihypertensive diet. In children, the evidence was inconclusive regarding the effectiveness for otitis media and enuresis, and it was not effective for infantile colic and asthma when compared to sham manipulation. The evidence was inconclusive for knee osteoarthritis, fibromyalgia, myofascial pain syndrome, migraine headache, and premenstrual syndrome. In children, the evidence was inconclusive for asthma and infantile colic.

Bronfort et al. [20] referred to the limitations of the available evidence and a range of issues that needed exploring in a more extensive review. The current work aimed to:

• Synthesise evidence in addition to the randomised controlled trials (RCTs) and systematic reviews captured by Bronfort et al. [20] such as controlled cohort studies, non-randomised controlled clinical trials (CCTs), and qualitative studies, focussing on evidence rated as ‘inconclusive’ or ‘negative’ by Bronfort, or not covered in the report

• Synthesise evidence additional to Bronfort et al. [20] (RCTs and systematic reviews published since Bronfort and additional study types)

• Compare conclusions from the additional studies summarised (new RCTs and systematic reviews and additional study types) to those of Bronfort et al. [20] focusing in particular on areas where it was stated that the available evidence was inconclusive or that manual therapy was not effective

## Methods

The PRISMA checklist for the current paper can be found in Additional file 1.

### Search strategy

We used a varied range of sources to identify relevant literature. A comprehensive literature search was undertaken in the major medical, health-related, science and health economic electronic bibliographic databases. We paralleled the comprehensive searches undertaken by Bronfort et al. [20] through a clearly defined search strategy using the databases: MEDLINE (Ovid), EMBASE, Mantis, Index to Chiropractic Literature, CINAHL, the specialised databases Cochrane Airways Group trial register, Cochrane Complementary Medicine Field register, and Cochrane Rehabilitation Field register (via CENTRAL). We supplemented these searches by using the following other databases: Science Citation Index, AMED, CDSR, NHS DARE, NHS HTA, NHS EED, CENTRAL (full search), and ASSIA, Social Science Citation Index.

Search terms were restricted to terms related to manual therapy and broader terms like ‘physiotherapy’ were not included. The search included both free text and MeSH terms, as well as terms for the eligible study types (from pretested search strategies). To keep the search as broad as possible, no condition terms were included. There was no language restriction in the searches but due to limited resources only studies published in English, French, German, and Spanish were included. The main search was carried out in August 2011 (see Additional file 2). A search update was undertaken in March 2013.

### Inclusion criteria

Studies were eligible for inclusion if they were full text reports of systematic reviews, RCTs or controlled clinical trials (CCTs), cohort studies with a comparison group, or qualitative studies of patients' views on manual therapy. Primary studies had to include at least 20 participants. Studies had to include participants of any age and in any setting treated for any musculoskeletal or non-musculoskeletal condition who were treated with any manual treatment/therapy were included (alone or in combination). To provide an overall picture, any condition was included for which a trial documenting manual treatment was available. Interventions had to include an element of manipulation or mobilisation, and emphasis was on interventions typically carried out by a manual therapist/chiropractor/osteopath. Comparison was against any other therapy. Outcomes assessed included pain intensity, pain-related disability, analgesic use, function, mobility, activities of daily living, characteristic symptoms or indicators of disease, patient satisfaction, quality of life, views/themes from qualitative data, adverse events (e.g. strokes, fractures, pain), and mortality. The focus of the present review was on evidence rated as ‘inconclusive’ or ‘negative’ by Bronfort et al. [20] or not covered in the report.

### Study selection

Two independent reviewers applied the inclusion/exclusion criteria to the studies identified through the searches, screened the titles/abstracts and then the full text of any records appearing to fulfil the inclusion criteria. Any disagreements over the inclusion of records were resolved by discussion.

### Data extraction

Data were extracted by two reviewers using *a priori* developed data extraction forms. The data extracted included: a) study characteristics (e.g., author name, year of publication, country, study design, aim, duration, follow-up, quality rating), b) types of participants (e.g., number, age, gender, inclusion/exclusion criteria), c) types of interventions including comparators (e.g., intervention groups, comparison, dose, providers), d) outcomes (e.g. pain, function, adverse events).

### Quality assessment

The following assessment tools were used for appraising any new and additional evidence: AMSTAR (for systematic reviews); [30-32] Cochrane Risk of Bias (for RCTs); [33] CRD checklist (for controlled cohort studies); [34] and CASP (for qualitative studies) [35]. Based on the quality results, studies were rated as high (more than two thirds of criteria met), medium (more than a third of criteria met) or low quality (a third or fewer criteria met).

### Rating of evidence

Using the same criteria as Bronfort et al. [20] (based on consistency between studies, study size, quality etc.), the evidence was rated as ‘high quality positive/negative evidence’, ‘moderate quality positive/negative evidence’, or ‘inconclusive favourable/non-favourable/unclear evidence.

### Data synthesis

To obtain an overview of new and potentially relevant studies omitted by Bronfort et al. [20], all systematic reviews and RCTs included by Bronfort were tabulated, by condition as classified in the report. Then eligible studies identified in our search strategy were entered in an Excel table and filtered by the relevant condition and any studies not already included by Bronfort et al. [20] were checked for their relevance and listed (with systematic reviews, RCTs and other study types listed in separate columns) if they were judged to be relevant additional studies. This process was followed for all conditions, and conditions not included by Bronfort were added. Studies were only included in the table after obtaining and checking their full text publication. When summarising systematic reviews on broader topics than the one considered in this review (e.g. of complementary therapies or physiotherapy in general), only sections of relevance to the current review were considered. Meta-analyses were not carried out as interventions and participant populations were very heterogeneous.

## Results

### Search results

**Figure 1:**
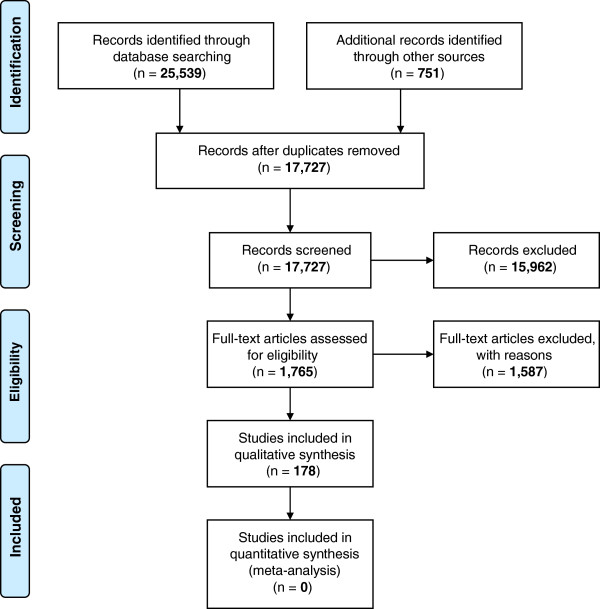
PRISMA 2009 flow diagram.

The initial database searches yielded 25,539 records (16,976 after deduplication). Of these, 178 were summarised in more detail (72 systematic reviews, 96 RCTs, 10 non-randomised primary studies), see Figure 1 for study flow chart. Reasons for exclusion included: absence of comparison group, irrelevant outcomes, study in healthy volunteers, ineligible intervention, ineligible condition, relevant intervention similar in all comparison groups, conference abstracts or commentaries, non-systematic review. The kappa statistic of inter-rater agreement was calculated for a 20% convenience sample of studies screened by two reviewers. Kappa was 0.74 (95% CI: 0.70, 0.77, equivalent to 93.9% agreement) indicating ‘substantial’ agreement [36].

Tables 1 and 2 summarise the number of studies rated by Bronfort et al. [20] to be ‘inconclusive’ or ‘negative’ compared to the new/additional studies identified in the current review for musculoskeletal conditions, headache and other disorders (Table 1) and non-musculoskeletal conditions and adverse events (Table 2).

**Table 1:** Number of studies in UK evidence report and current review – Musculoskeletal conditions, headache disorders, fibromyalgia

**Condition**	**UK evidence report**		**Current review (additional studies)**		
	***Systematic reviews***	***RCTs***	***Systematic reviews***	***RCTs***	***Other primary study types***
**Conditions/Interventions with inconclusive or negative evidence in the UK evidence report**					
**Musculoskeletal**					
Sciatica/radiating leg pain	3	*Details of RCTs in reviews not listed*		2 & 1 ongoing	
Neck pain (cervical manipulation/mobilisation only)	*Unclear*	*Unclear*		7	
Non-specific mid back pain	0	7 *[not all thoracic back pain]*	1	1 ongoing	
Coccydynia	0	1			
Ankle and foot conditions	2	16	3	6 & 1 ongoing	
Carpal tunnel syndrome	4	2	4	3	
Lateral epicondylitis	3	11	8	9 & 1 ongoing	2 controlled clinical trials & 1 cohort
Temporo-mandibular disorders	2	5	1 ongoing	5	
Shoulder conditions	2	6	15	12	
**Headache disorders and fibromyalgia**					
Cervicogenic headache	4	7	2	2	
Tension-type headache	5	12	1	4	
Miscellaneous headache	1	1	2	2	
Fibromyalgia	3	8	3	2	

**Table 2:** Number of studies in UK evidence report and current review - Non-musculoskeletal, adverse events

**Condition**	**UK evidence report**		**Current review (additional studies)**		
	***Systematic reviews***	***RCTs***	***Systematic reviews***	***RCTs***	***Other primary study types***
**Conditions/Interventions with inconclusive or negative evidence in the UK evidence report and additional conditions not covered by the UK evidence report**					
**Non-musculoskeletal**					
Asthma	4	5	3	1	1 qualitative
ADHD/Learning disorders	*Not reported*	*Not reported*	1	2	
Cancer care	*Not reported*	*Not reported*	1	4	
Cerebral palsy	*Not reported*	*Not reported*		3	
Cervicogenic dizziness/balance	2	2	1	1 & 1 ongoing	
Chronic fatigue	*Not reported*	*Not reported*	1		
Chronic pelvic pain	*Not reported*	*Not reported*	2	3	
Cystic fibrosis	*Not reported*	*Not reported*		1	
Dysfunctional voiding (paediatric)	*Not reported*	*Not reported*		1	
Gastrointestinal	*Not reported*	*Not reported*	1	2	
Hypertension	1	3	1		1 controlled clinical trial
Infantile colic	6	8	4	1	
Insomnia			1		
Menopausal symptoms	*Not reported*	*Not reported*		1	
Myofascial pain syndrome	1	15	2	4	
Otitis media	3	2	2	1 ongoing	
Parkinson’s	*Not reported*	*Not reported*		1	
Paediatric nocturnal enuresis	2	2	1		
Peripheral arterial disease	*Not reported*	*Not reported*		1	1
Pneumonia/respiratory disorders	1	1	3	2 & 1 ongoing	
Pregnancy/post-natal/neonatal	*Not reported*	*Not reported*	2	2 & 1 ongoing	1
Rehabilitation	*Not reported*	*Not reported*		3	2 controlled clinical trials & 1 cohort
Systemic sclerosis	*Not reported*	*Not reported*		2	
Dysmenorrhoea	2	5			
Premenstrual syndrome	3	3			
**Adverse events**	5	*Primary studies:* 6	7	7	

### Update on clinical effectiveness–conditions/interventions rated ‘inconclusive’, ‘negative’ or not covered in the UK evidence report

The following section provides a summary of the conditions/interventions rated ‘inconclusive’, ‘negative’ or not covered in the UK evidence report. Details of study characteristics and study quality can be found in Additional files 3 and Additional file 4.

#### Musculoskeletal conditions

Tables 3, 4 and 5 provide a comparison between the overall evidence ratings included in the UK evidence report and new/additional studies in the current review for musculoskeletal conditions.

**Table 3:** Comparison of evidence in UK evidence report and current review for musculoskeletal spinal conditions

**Condition**	**Intervention**	**UK evidence report evidence**			**New/additional evidence**			**New evidence?**
		**Inconclusive**	**Moderate**	**High**	**Inconclusive**	**Moderate**	**High**	
**Musculoskeletal *- Spinal***								
Sciatica/radiating leg pain	Spinal manipulation/mobilisation	Favourable			Favourable			Yes
Neck pain (acute/subacute/chronic)	Cervical spinal manipulation/mobilisation alone	Favourable			Favourable			Yes
	Manipulation and mobilisation with/without soft tissue treatment				Favourable			Yes
Mid back pain	Spinal manipulation	Favourable			Favourable			No
Coccydynia	Spinal manipulation	Favourable			Favourable			No
Temporomandibular disorders	Mobilisation/massage	Favourable			Favourable			No
	Mandibular manipulation				Unclear			Yes
	Intra-oral myofascial therapy				Favourable			Yes
	Osteopathic manual therapy (cervical and temporomandibular joint regions)				Favourable			Yes
Myofascial pain syndrome	Ischaemic compression				Favourable			Yes
• active upper trapezius trigger points, neck pain	Trigger point release				Non-favourable			Yes
	Integrated neuromuscular inhibition technique				Favourable			Yes

**Table 4:** Comparison of evidence in UK evidence report and current review for musculoskeletal upper extremity disorders

**Condition**	**Intervention**	**UK evidence report evidence**			**New/additional evidence**			**New evidence?**
		**Inconclusive**	**Moderate**	**High**	**Inconclusive**	**Moderate**	**High**	
**Musculoskeletal *- Upper extremity disorders***								
Carpal tunnel syndrome	Mobilisation	Favourable			Favourable			No
	Trigger point therapy	Favourable			Favourable			Yes
	Diversified chiropractic care				Unclear			Yes
	Neurodynamic technique				Unclear			Yes
	Soft tissue mobilisation (with or without Graston instrument)				Unclear			Yes
Lateral epicondylitis	Manipulation	Non-favourable			Non-favourable			No
	Manual tender point therapy	Favourable			Favourable			No
	Mobilisation with exercise	Favourable			Favourable			
Shoulder disorders • Shoulder girdle pain/dysfunction	Manipulation/mobilisation (mobilisation with movement)		Positive			Positive		No
• Rotator cuff disorder	Manipulation/mobilisation (with exercise)	Favourable				Positive		Yes
• Adhesive capsulitis	High grade mobilisation		Positive			Positive		No
	Mobilisation with movement				Favourable			Yes
	Osteopathy (Niel-Asher technique)				Favourable			Yes
	Manual therapy with exercise				Favourable			Yes
• Minor neurogenic shoulder pain	Cervical lateral glide mobilisation and/or high velocity low amplitude manipulation with soft tissue release and exercise				Favourable			Yes
• Soft tissue shoulder disorders	Myofascial treatments (ischaemic compression, deep friction massage, therapeutic stretch)					Positive		Yes

**Table 5:** Comparison of evidence in UK evidence report and current review for musculoskeletal lower extremity disorders

**Condition**	**Intervention**	**UK evidence report evidence**			**New/additional evidence**			**New evidence?**
		**Inconclusive**	**Moderate**	**High**	**Inconclusive**	**Moderate**	**High**	
**Musculoskeletal *- Lower extremity disorders***								
Ankle sprains	Manipulation/mobilisation	Favourable			Favourable			No
	Muscle energy technique				Favourable			Yes
Ankle fracture rehabilitation	Mobilisation		Negative			Negative		No
	Kaltenborn-based manual therapy				Favourable			Yes
Morton’s neuroma/metatarsalgia	Manipulation/mobilisation	Favourable			Favourable			No
Hallux limitus	Manipulation/mobilisation	Favourable			Favourable			No
Plantar fasciitis	Manipulation/mobilisation with exercise		Positive			Positive		No
	Trigger point therapy				Favourable			Yes
Hallux abducto valgus	Manipulation/mobilisation	Favourable			Favourable			Yes

##### 

**Sciatica and back-related leg-pain** Two new/additional medium [37] to high quality [38] RCTs relating to sciatica and back-related leg pain were identified, and a protocol of an on-going study [39]. The medium quality RCT [37] randomised 40 patients with sciatica to receive chiropractic spinal manipulation (high velocity, low-amplitude, short lever technique) or surgical microdiskectomy. At 12 weeks of follow-up there was significant improvement in quality of life, pain and disability in both intervention groups, with no significant difference between groups. The high quality RCT [38] randomised 134 patients with non-specific low back pain (with or without sciatica) to orthopaedic manual therapy (mobilisation, high velocity low-force manipulation, translatoric thrust manipulation), the McKenzie method or advice only. At 12 months follow-up, all groups showed significant improvement in pain and disability, but there was no significant difference between groups.

*Summary:* Inconclusive (favourable) evidence for spinal manipulation/mobilisation in treating sciatica and back-related leg-pain (no change from the UK evidence report). The evidence suggests that chiropractic or orthopaedic manipulation may be effective in reducing symptoms of sciatica in adults, however, it is not clear due to the small sample size of the trials, if these manual treatment techniques are more beneficial compared to surgery, McKenzie method, or advice only.

##### 

**Neck pain (cervical manipulation/mobilisation alone)** One low quality, [40] four medium quality [41-43] and two high quality RCTs [44,45] examined the effect of cervical spinal manipulation or mobilisation alone for neck pain of any duration [40-46].

A medium quality RCT [46] compared the effects of joint mobilisation applied to either symptomatic or asymptomatic cervical levels in 48 patients with chronic non-specific neck pain. Outcomes were only measured immediately following the treatment and while there were some within-group improvements in pain parameters, there were no significant differences between groups. In a medium quality RCT, [42] 47 patients were randomised to receive a three-week treatment with cervical manipulation (cervical/upper thoracic segmental high velocity, low amplitude movements), mobilisation (cervical/upper thoracic segmental low velocity, low amplitude movements), or the activator instrument. At 12 months post-treatment, the proportion of patients who improved on the Patient Global Impression of Change scale was not significantly different across the three study groups, neither were any changes in disability, pain, or quality of life. However, there were significant within-group improvements from baseline in disability and pain intensity for the manipulation and activator instrument groups. Fifteen patients in the manipulation and four patients in each group of the mobilisation and activator experienced minor adverse events (e.g. mild headache, mild dizziness, mild arm weakness). Klein et al. [45] compared a single strain-counter strain intervention with sham treatment in a high quality RCT including 61 patients with neck pain. After the treatment, there was no significant difference between groups in mobility restriction or pain. Leaver et al. [44] conducted a high quality RCT comparing the effectiveness of cervical manipulation (high-velocity, low-amplitude thrust technique) versus mobilisation (low-velocity, oscillating passive movements) administered to 182 patients with non-specific neck pain (less than 3 months of duration) for two weeks. At three months of follow-up, the median number of days to recovery was not significantly different between the manipulation and mobilisation groups, and there was also no significant difference between the two groups in the mean post-treatment pain intensity, in disability, in function, and in global perceived effect. The most frequent adverse events were minor and included increased neck pain (22%) and headache (22%). Other less frequent events were dizziness (7%), nausea (6%), and paraesthesia (7%). The frequency of adverse events was not significantly different between the study groups. Martel et al. [43] conducted a medium quality RCT of 98 patients with non-specific chronic neck pain, investigating the effectiveness of spinal manipulative therapy (standardised passive palpation on the cervical and thoracic spine) compared to spinal manipulative therapy plus home exercise, or no treatment for 10 months. After the treatment phase, all study groups experienced significant improvements in disability and lateral flexion. However, the between-group differences for all outcome measures were statistically non-significant. Puentedura et al. [41] conducted a medium quality RCT comparing the effectiveness of 2-week thoracic thrust joint manipulation (TJM) plus cervical range of motion (ROM) exercises to that of cervical thoracic thrust joint manipulation plus cervical ROM exercises in 24 adults with acute neck pain. At six months of follow-up, the cervical TJM group compared to the thoracic TJM group experienced significantly improved scores for neck disability and overall success. Minor transient adverse events (increased neck pain, fatigue, headache, upper back pain) were reported by 70%-80% of the participants in the thoracic TJM group versus 7% in the cervical TJM group. In a low quality RCT, Schomacher et al. [40] randomised 126 adult participants with chronic neck pain to receive a single 4-minute mobilisation technique (intermittent translatoric traction at the zygopophyseal joint between C2 and C7 with Kaltenborn’s grade II force) applied to symptomatic levels (concordant segment) versus asymptomatic levels (three levels below/above concordant segment) of the cervical spine. The immediate post-treatment between-group differences for the mean change in pain were not statistically significant.

*Summary:***Inconclusive (favourable) evidence for cervical spinal manipulation/mobilisation alone in treating neck pain** (no change from the UK evidence report). **Inconclusive (favourable) evidence for manipulation and mobilisation with/without soft tissue treatment** (not evaluated in the UK evidence report). The evidence suggests there are similar improvements in the manipulation and/or mobilisation intervention groups compared to active treatment, however, some trials also found no improvement in comparison to a control group.

##### 

**Non-specific mid-back pain** One additional low quality systematic review [47] and one on-going RCT [48] were identified on non-specific mid-back pain. The systematic review included only one RCT [49] eligible for the current review, but this had already been included in the UK evidence report.

*Summary:***Inconclusive (favourable) evidence** (no change from the UK evidence report). It cannot be established from the current evidence whether manual therapy is more effective than non-treatment, placebo, or other treatments for the treatment of non-specific mid-back pain.

##### 

**Ankle and foot conditions** Three additional systematic reviews [50-52] (2 high quality, [50,52] 1 medium quality [51]) and six additional RCTs [53-58] (1 high quality, [58] 2 medium quality [53,56] and 3 low quality [54,55,57]) and one ongoing RCT [59] were identified on the treatment of ankle and foot conditions using manual therapy.

A high quality Cochrane review examined the effect of rehabilitation interventions for ankle fractures [50]. With respect to manual therapy, one trial with a high risk of bias [57] and one trial with a low risk of bias [58] were identified. The trial by Wilson and colleagues included only 12 participants in total, who had an ankle fracture treated with or without surgery. The intervention group received physiotherapy including Kaltenborn-based manual therapy to the talocrural and talocalcaneal joints, both groups also received an exercise intervention. After five weeks of treatment, there was no statistically significant improvement in activity limitation or ankle plantarflexion range of motion, but the ankle dorsiflexion range of motion was statistically significant in favour of manual therapy. Lin et al. compared treatment with manual therapy (anterior-posterior joint mobilisation over the talus) plus a standard physiotherapy programme (experimental) with the standard physiotherapy programme only in 94 patients with ankle fracture within one week of cast removal. There was no significant difference between groups in functional, pain or quality of life parameters at 24 weeks’ follow-up. The review authors concluded that there is no evidence that manual therapy after a period of immobilisation may improve ankle range of motion in patients after ankle fracture. Another high quality systematic review [52] examined the effects of manipulative therapy for lower extremity conditions. The authors identified one high, ten moderate and two low quality trials concerning manual therapy after ankle inversion sprain, one high and one moderate quality trial concerning plantar fasciitis, one moderate and one low quality trial concerning metatarsalgia, four moderate quality trials concerning decreased proprioception/balance/function secondary to foot and ankle injury/decreased range of motion/joint dysfunction, one moderate quality trial concerning hallux limitus and two moderate quality trials concerning hallus abducto valgus. They concluded that there was moderate evidence for manual therapy (mobilisation/manipulation) of the knee and/or full kinetic chain and of the ankle and/or foot, combined with multimodal or exercise therapy for ankle inversion sprain and limited evidence regarding long term effects. There was also moderate evidence for manual therapy (mobilisation/manipulation/stretching) of the ankle and/or foot combined with multimodal or exercise therapy for short-term treatment of plantar fasciitis. There was limited evidence for manual therapy (manipulation/mobilisation) of the ankle and/or foot combined with multimodal or exercise therapy for short-term treatment of metatarsalgia and hallux limitus/rigidus and for loss of foot and/or ankle proprioception and balance. There was insufficient evidence for manual therapy (mobilisation/manipulation) of the ankle and/or foot for hallux abducto valgus. The authors suggested that further high quality research is needed.

A low quality RCT [54] examined the effects of a muscle energy technique versus manipulation in the treatment of 40 patients with chronic recurrent ankle sprain. After six chiropractic treatments over three weeks, there was significant improvement over time in the One Leg Standing Test (eyes open and closed), the McGill Pain Questionnaire, the Functional Evaluation Scale, and in dorsiflexion and plantarflexion; however, there was no significant difference between the two groups. Adverse events were reported but no serious adverse events were seen. Du Plessis et al. [53] conducted a medium quality trial of chiropractic treatment in patients with hallux abducto valgus. Thirty patients were included and the intervention group was treated four times over two weeks with graded joint mobilisation of the first metatarsophalangeal joint plus joint manipulation, while the control group received a night splint. At the end of the intervention, there was no significant difference between the groups in terms of pain and foot function scores (with both groups showing improved values). However, these improvements were not maintained in the control group, while they were maintained in the intervention group (significant difference between groups in favour of the manual therapy group at the one month follow-up, p < 0.01). Hallux dorsiflexion was significantly greater in the manual therapy group both at the end of the intervention and at the end of the one month follow-up. Adverse events were reported but no serious adverse events were seen. Another medium quality RCT [56] examined the effects of manual therapy in the treatment of plantar heel pain. The trial included 60 patients treated four times weekly for four weeks. Both groups received a self-stretching intervention (directed at the calf muscles and plantar fascia) and the intervention group also received myofascial trigger point manual therapy. After the intervention, results for pressure pain thresholds were significantly better for the manual therapy than for the stretching only group (p < 0.03) and results for the physical function and bodily pain subscales on the SF-36 quality of life questionnaire were also improved in favour of manual therapy. No significant differences were seen in any other subscales of the SF-36. Similarly, a low quality RCT [55] examined the effects of myofascial therapy in 30 patients with plantar fasciitis and found significant pain and foot function values in the intervention group compared to the control.

*Summary:***Inconclusive (favourable) evidence** that manipulation, mobilisation, and a muscle energy technique are of benefit in the treatment of ankle sprains (not evaluated in UK evidence report). **Inconclusive (favourable) evidence** for Kaltenborn-based manual therapy for rehabilitation following ankle fracture (not evaluated in the UK evidence report). **Inconclusive (favourable) evidence** for hallux abducto valgus that mobilisation/manipulation is more effective in leading to improvements in the intermediate term than night splints (no change from UK evidence report). **Inconclusive (favourable) evidence** for trigger point therapy in treating plantar fasciitis treatment (no change from the UK evidence report). **Inconclusive (favourable) evidence** for manual therapy (manipulation/mobilisation) of the ankle and/or foot combined with multimodal or exercise therapy (no change from the UK evidence report) in treating Morton’s neuroma, metatarsalgia, hallux limitus/rigidus.

##### 

**Carpal tunnel syndrome** Three additional medium quality systematic reviews, [60,61] one high quality systematic review [62] and three additional RCTs [63-65] on the effectiveness of manual therapy in carpal tunnel syndrome were identified. The medium quality reviews [60,61] and a high quality review [62] did not include any eligible trials not already considered by the UK evidence report and were therefore not considered here. Two medium quality RCTs [63,64] were included in one of the additional systematic reviews [66]. Only one medium quality RCT [65] was not included in any of the new reviews.

The medium quality systematic review [66] summarised evidence on the effectiveness of non-surgical treatments for carpal tunnel syndrome. The authors concluded that there is limited evidence that carpal bone mobilisation is more effective with respect to symptom improvement than no treatment in the short term in the treatment of carpal tunnel syndrome. There was no evidence found for the effectiveness of neurodynamic treatment versus carpal bone mobilisation in the short term, for the effectiveness of a neurodynamic technique plus splinting compared with a sham therapy plus splinting group in the short term, or for the effectiveness of Graston instrument-assisted soft tissue mobilisation plus home exercises compared with soft tissue mobilisation plus home exercises in the midterm. There was no evidence for the effectiveness of chiropractic therapy compared with medical treatment for in the midterm.

A medium quality RCT [65] was and compared 15 sessions of trigger point therapy over five weeks with sham treatment in 55 patients with carpal tunnel syndrome. After the end of the intervention, there was significant improvement in the severity of symptoms, functional status and perceived improvement in the intervention group compared to control (p < 0.05).

*Summary:***Inconclusive (favourable) evidence** for carpal bone mobilisation and for trigger point therapy in the treatment of carpal tunnel syndrome (no change from the UK evidence report). **Inconclusive (unclear) evidence** for neurodynamic treatment, soft-tissue mobilisation (with or without Graston instrument), and diversified chiropractic care in the management of carpal tunnel syndrome (not evaluated in the UK evidence report).

##### 

**Lateral epicondylitis (tennis elbow)** Eight additional low to moderate quality systematic reviews, [60,67-73] eight additional RCTs for low to moderate quality, [74-81] one ongoing RCT, [82] and three low quality non-randomised comparative studies were identified [83-85]. Four of the additional RCTs [75,77-79] were included in the new additional reviews.

One systematic review of medium quality [69] evaluated the effectiveness of manipulative therapy (MT) in treating adults with lateral epicondylitis. The review identified and included 13 randomised and non-randomised trials of fair quality overall. The review results indicated beneficial effects of Mulligan’s mobilisation with movement (versus no treatment, placebo, or corticosteroid injection) and manual therapy applied to the cervical spinal region (versus placebo). Cyriax physiotherapy was found to be more effective than conventional therapy (stretching, exercise, and modalities), but less effective than corticosteroid injection or supervised exercise. Kohia et al. [70] systematically reviewed the effectiveness of various physical therapy treatments for lateral epicondylitis in adults (medium quality). In total, 16 RCTs of physical therapy were included in the review. The findings indicated that in the short-term (6 months or less), corticosteroid injections were more beneficial than physical therapy (elbow manipulation and exercise) or Cyriax physiotherapy. However, in the longer term (six months or longer), there was no difference between physical therapy (elbow manipulation and exercise) versus corticosteroid injections or no treatment. Moreover, radial head mobilisation was more effective compared to standard treatment (ultrasound, massage, stretching, exercise for wrist) at a follow-up of 15 weeks. The physical therapy protocol (pulsed ultrasound, friction massage, and stretching, exercise for wrist) was more effective than a brace with or without pulsed ultrasound. Cyriax physiotherapy was more beneficial than light therapy but less beneficial than supervised exercise of wrist extensors. And finally, the use of wrist manipulation led to greater improvements in lateral epicondylitis than a combination of ultrasound, friction massage, and muscle strengthening. According to the review authors, no single treatment technique was shown to be the most effective in treatment of lateral epicondylitis. In one systematic review of medium quality, [71] the authors explored the effectiveness of physiotherapy, steroid injections, and relative rest for the treatment of adult lateral epicondylitis. The review identified and included 30 studies with quality scores ranging from 2 to 9 (out of 11). After 6 weeks of follow-up, steroid injections and multimodal physiotherapy (arm stretching, strengthening, ultrasound, and massage) were more effective than relative rest. However, after 3 months, multimodal physiotherapy was better than steroid injections but as effective as relative rest. The authors concluded that early active interventions such as steroid injections and multimodal physiotherapy may improve symptoms of lateral epicondylitis in adults. In a medium quality systematic review, [73] evidence was summarised on the effectiveness of conservative treatments (e.g., ultrasound, acupuncture, rebox, exercise, wait and see, mobilisation/manipulation, laser) for lateral epicondylitis in adults. In total, 31 trials of conservative treatment were included, of which four trials reported on effectiveness of mobilisation/manipulation relative to placebo, standard physiotherapy, corticosteroid injections, or manipulation in combination with treatments. The results indicated that mobilisation/manipulation led to greater improvements in symptoms of lateral epicondylitis compared to placebo or standard physiotherapy. However, at one year of follow-up, there was no difference between corticosteroid injections and manipulation/mobilisation (Cyriax group). The authors concluded that level 2b (Sackett’s evidence rating) evidence indicated benefits of mobilisation/manipulation in treating lateral epicondylitis.

In one pilot study of low quality, [74] the authors randomised 30 adults with lateral epicondylitis to receive either the chiropractic mobilisation (augmented soft tissue technique) or no treatment for five weeks. After three months of follow-up, the groups demonstrated significant improvements in the Patient-Rated Tennis Elbow Evaluation scale, in pain and in pain-free grip strength when compared to baseline. However, no between-group difference for these measures was statistically significant. In one trial of medium quality, [76] 60 adult participants with lateral epicondylitis were randomised to 4-week Cyriax physiotherapy versus phonophoresis with diclofenac gel and supervised exercise. At 4 and 8 weeks, both groups demonstrated significant improvements in pain (VAS scale), pain-free grip strength (dynamometer), and functional status when compared to baseline. At both follow-ups, there were significantly greater mean improvements in pain, pain-free grip strength, and functional status with Cyriax physiotherapy compared to phonophoresis. In a non-randomised controlled experimental trial of low quality, [83] the effect of the Mulligan technique (mobilisation, movement and taping) plus traditional treatment (thermal treatment, massage, ultrasound, exercise) was compared to that of traditional treatment alone given for 4 weeks to 34 participants with lateral epicondylitis. After 4 weeks, both groups demonstrated significant improvements in function, pain, and pain-free grip strength when compared to baseline with the mean improvements from baseline in pain and function being significantly greater in the Mulligan technique group compared to traditional treatment alone. A low quality RCT [80] compared the effects of myofascial release with sham ultrasound in 68 computer professionals with lateral epicondylitis (12 sessions over 4 weeks). At 12 weeks follow-up, values on the Patient-rated Tennis Elbow Evaluation Scale were significantly more improved in the intervention group than in the control group. A low quality RCT [81] compared the effectiveness of a supervised exercise programme with that of Cyriax physiotherapy (12 sessions over 4 weeks) in 20 patients with lateral epicondylitis. After the end of the treatment, pain and function (Tennis Elbow Function Scale) were significantly improved in both groups, but significantly more in the exercise group than in the Cyriax physiotherapy group.

In one observational cohort study of low quality, [84] the authors retrospectively compared the effectiveness of adding cervical spine manual therapy (passive mobilisation, mobilisation with movement, muscle energy techniques) to local management directed at the elbow (pulsed ultrasound, iontophoresis, deep tissue massage, stretching, strengthening exercise for muscles of the upper extremity, cold packs, elbow joint mobilisation) administered to patients with lateral epicondylitis. The authors reviewed and divided charts of 112 participants into two groups of the cervical spine manual therapy plus local management (n = 51) versus local management alone (n = 61). The self-reported outcome of success (i.e., return to all functional activities without recurrence of elbow symptoms after discharge from physical therapy) was ascertained via telephone follow-up interviews (72–74 weeks after discharge) with a response rate of 85% (95 responders). Compared to the local management group, the cervical spine manual therapy group experienced a numerically higher rate of success in fewer visits. In a non-randomised controlled experimental trial of low quality [85] manual therapy (soft mobilisation of the cervical spine/cervicothoracic junction and flexion mobilisation in the cervical joints) plus extracorporeal low-energy shockwave therapy (ESWT) was compared to that of ESWT alone given to 60 participants with chronic lateral epicondylitis. At 12 months of follow-up, both treatment groups experienced significant improvements in pain compared to baseline. However, there was no statistically significant difference between groups.

*Summary:***Inconclusive (non-favourable) evidence** was found for the treatment of lateral epicondylitis (tennis elbow) with manipulation alone (no change from the UK evidence report). The reviewed evidence indicated some benefits of manual therapy in reducing symptoms in patients with lateral epicondylitis, when in combination with other treatments (exercise, traditional physiotherapy, local management, standard therapy), when compared to no treatment, or baseline values (within-group change), however, the evidence was still rated **inconclusive (favourable) evidence** (no change from the UK evidence report). When comparing manual therapy to other treatments (e.g., placebo, phonophoresis, low-energy shockwave therapy, relative rest), there was **inconclusive or inconsistent (favourable) evidence** (no change from the UK evidence report).

##### 

**Shoulder conditions** Fifteen new or additional systematic reviews [60,86-99] were identified that included assessments of manual therapy for shoulder pain and disorders with inconclusive results in the UK evidence report, as well as twelve new or additional RCTs [100-112]. However, eleven of the reviews were either included in other more comprehensive reviews or did not include any studies in addition to those in the UK evidence report or to those included in more specific reviews, [60,89-93,95-99] and nine of the RCTs were included in relevant new reviews and will therefore not be described separately here [100,101,104-110]. The remaining systematic reviews were rated medium quality [86-88,94]. The new RCTs not described in any of the reviews were of high [103,111] and medium [112] quality.

In one of the new systematic reviews, [86] the authors examined the effects of manipulative therapy with or without multimodal therapy for shoulder disorders. They identified 23 RCTs, five non-randomised trials, and seven non-controlled primary studies. The included studies used a variety of intervention techniques including mobilisation, manipulation with and without exercise, combination with soft tissue treatment in some studies, mobilisation with movement, myofascial treatments, and cervical lateral glide mobilisation. Each condition category examined (other than shoulder osteoarthritis) included at least one high quality study. The authors concluded that for rotator cuff disorders and for shoulder complaints, dysfunctions, disorders or pain, there was fair evidence for manual and manipulative therapy of the shoulder, shoulder girdle and/or full kinetic chain combined with multimodal or exercise therapy; similarly for frozen shoulder (adhesive capsulitis), there was fair evidence for manual and manipulative therapy of the shoulder, shoulder girdle and/or full kinetic chain combined with multimodal or exercise therapy (manual therapy included high velocity low amplitude manipulation, mid-or end-range mobilisation, mobilisation with movement). For shoulder soft tissue disorders there was fair evidence for using soft tissue or myofascial treatments (ischaemic compression, deep friction massage, therapeutic stretch). For minor neurogenic shoulder pain there was limited evidence for cervical lateral glide mobilisation and/or high velocity low amplitude manipulation with soft tissue release and exercise. There was insufficient evidence for the manual treatment of shoulder osteoarthritis (no trials in this patient group). Another medium quality systematic review [87] examined the effectiveness of manual therapy for impingement-related shoulder pain. They considered systematic reviews, RCTs and quasi-RCTs of manual or exercise therapy in patients with pain arising locally in a shoulder with grossly abnormal mobility. The review included eight systematic reviews and six RCTs, of which three included exercise interventions only and three included both exercise and manual therapy (mobilisation). Of the included reviews, five reported evidence to favour manual therapy plus exercise over exercise alone. The evidence from the three additional RCTs was inconclusive, but with a tendency towards improved outcomes with interventions including both manual therapy and exercise. No evidence was found for the effectiveness of mobilisation alone. None of the systematic reviews and only one of the RCTs included a specific statement on adverse events; in the one RCT no adverse events were reported. The authors concluded that there is limited evidence to support the effectiveness of manual therapy and exercise interventions for impingement-related shoulder pain. This primarily related to sub-acute and chronic complaints and short and medium term effectiveness, with the conclusions being based on research of varying methodological quality, with varying risk of bias, and affected by weaknesses in the reporting quality. Cautious interpretation was also warranted due to the heterogeneity of populations, interventions and outcomes.

A medium quality systematic review [88] examined the effectiveness of manual physical therapy for painful shoulder conditions. Treatment had to be by physical therapists and manual therapy interventions including low and high velocity mobilisations had to be directed at the glenohumeral joint only, without mobilisation of adjacent structures. Seven RCTs with a mean PEDro quality score of 7.86 of 10 (range 6 to 9) were included, and interventions included mobilisation with movement, the Cyriax approach, and static mobilisation performed at end-range or mid-ranges of motion. Of the included trials, three examined mobilisation with movement and two of these found a significant improvement in range of motion in the intervention group compared to the control, while the highest percentage change in range of motion was found in the intervention group in the third study. Significant improvement in pain compared to control was seen in one of two studies, and significant functional improvement in one study and highest percentage change in function in a second study. One study on Cyriax manual therapy found significant improvement in range of motion compared to the control, while three studies examining mobilisation at the end-range of motion all found a significant improvement in range of motion and end-range mobilisation compared to the control, while two studies reported no significant change in pain measures and two of three studies reported significantly improved function compared to the control. Mid-range mobilisation appeared to be less effective with no effect on range of motion or function and only one of four studies reporting a significant improvement in pain. The review authors concluded that the included studies demonstrated a benefit of manual therapy for improvements in mobility and a trend towards improving pain measures, while increases in function and quality of life were questionable. Similarly, Pribicevic et al. [94] examined in their medium quality review the effectiveness of manipulative therapy for the treatment of shoulder pain (excluding adhesive capsulitis). Treatment had to include a manipulative thrust technique (chiropractic or physiotherapy). The authors included 22 case reports, four case series, and four RCTs. The RCTs had quality scores of 5 to 8 out of 10. One included chiropractic manipulations and three included physiotherapeutic manipulations. All trials provided some limited evidence that the groups receiving the manipulation intervention had better outcomes (in terms of pain, recovery, improvement) than the control groups. The authors concluded that the evidence was limited, as only two RCTs of reasonably sound methodology could be identified and that there is need for well-designed trials investigating multi-modal chiropractic treatment.

A low quality RCT examined the effects of manual therapy (mobilisation of the glenohumeral joint and soft tissues using Kaltenborn’s roll-glide techniques, Cyriax deep transverse massage, Mulligan’s mobilisation with movement and typical techniques of glenohumeral joint mobilisation in the anteroposterior direction) in 30 patients with chronic rotator cuff injury [102]. The duration of the treatment was unclear (at least 15 treatments) and the intervention was combined with standard rehabilitation (TENS, ultrasound, exercise). A range of mobility parameters as well as pain were significantly more improved in the manual therapy group than in the control group after the intervention. The authors did not report on adverse effects. Another RCT [103] was high quality and examined the effects of myofascial trigger point treatment in 72 patients with chronic unilateral non-traumatic shoulder pain (excluding adhesive capsulitis). The treatment involved inactivation of active myofascial trigger points by manual compression, which was combined with other manual techniques, namely deep stroking or strumming and intermittent cold application. Patients were also instructed to perform simple gentle static stretching and relaxation exercises at home several times a day to apply heat and received ergonomic advice. There was a ‘wait and see’ control group that received physiotherapy after the trial period. Treatment was given once a week for up to 12 weeks. After 12 weeks, the patients in the intervention group had significantly improved values for disability (DASH questionnaire), current pain, pain in the past seven days and most severe pain in the past seven days compared to the control. The Global Perceived Effect was also significantly better in the intervention than in the control group (55% versus 14% with improvement), as was the number of muscles with active trigger points. The authors did not report on adverse effects. A medium quality RCT [112] compared therapy according to the fascial distortion model with classic manual therapy in 60 patients with frozen shoulder. Patients received four treatment sessions over four weeks. Six weeks after the end of treatment function and pain improved in both groups, but significantly more so in the fascial distortion model group than in the classic manual therapy group. Patients found the fascial distortion model treatment more uncomfortable than classic manual therapy, but no serious adverse effects were seen. A high quality RCT [111] compared the effectiveness of end-range mobilisation/scapular mobilisation treatment in addition to standard physical therapy, compared to standard therapy alone in 34 patients with frozen shoulder syndrome. The main treatment groups included patients meeting criteria from a kinematics prediction, and an additional control group included patients not fulfilling the criteria. Treatment was provided twice weekly for eight weeks. At eight weeks, results for several range of motion parameters and function were significantly better for the intervention group fulfilling the criteria compared to the control group fulfilling the criteria. However, there was no difference between the intervention group and the control group not fulfilling the criteria. These results supported the use of a prediction method.

*Summary:***Moderate (positive) evidence** for use of manual therapy combined with exercise in the treatment of rotator cuff disorders (change from inconclusive (favourable) evidence in UK evidence report). **Inconclusive (favourable) evidence** for the effectiveness of mobilisation with movement (not evaluated in UK evidence report) or osteopathy (Niel-Asher technique) (not evaluated in UK evidence report) or manual therapy with exercise in the treatment of adhesive capsulitis (not evaluated in UK evidence report). **Inconclusive (favourable) evidence** for the effectiveness of cervical lateral glide mobilisation and/or high velocity low amplitude manipulation with soft tissue release and exercise in minor neurogenic shoulder pain (not evaluated in the UK evidence report). **Moderate (positive) evidence** for using myofascial treatments (ischaemic compression, deep friction massage, therapeutic stretch) for soft tissue disorders of the shoulder (not evaluated in the UK evidence report).

##### 

**Temporomandibular disorders** One systematic review protocol [113] and five RCTs [114-118] (3 low quality, [115,116,118] 2 high quality [114,117]) were identified on manual therapy for temporomandibular disorders.

Craane et al. [114] conducted a high quality RCT of 49 participants with temporomandibular closed lock who either received physical therapy (including joint mobilisation, exercises, and massage) or a control treatment. Over a year of follow-up, all pain variables decreased, and all function variables increased significantly over time for both groups, but there was no significant difference between the groups. In a low quality RCT, [116] 50 adults with temporomandibular disorders were randomised to receive osteopathic manual therapy or conventional conservative therapy (oral appliance, physical therapy, hot/cold packs, transcutaneous electrical nerve stimulation) for 6 months. At 8 months of follow-up, the osteopathic group compared to the conventional conservative therapy group experienced significant improvement in maximal mouth opening and lateral movement of the head around its axis, but the mean jaw pain score between the two groups was not significantly different. In a high quality RCT, [117] 30 participants with myogenous temporomandibular disorders were randomly assigned to receive one of the three treatments for 5 weeks: intra-oral myofascial therapy (IMT), IMT plus self-care (mandibular home exercises) and education (lecture on basic temporomandibular joint anatomy, biomechanics, disc displacement, dysfunction), or no treatment. At 6 months of post-treatment follow-up, both IMT groups compared to no treatment group experienced significant improvements in pain scores at rest, opening, and clenching (p < 0.01). Moreover, the IMT alone group had a significant improvement in pain at rest (p = 0.04), pain on opening (p < 0.01), and opening range (p < 0.01) compared to IMT combination with education and self-care. A low quality randomised trial [118] compared the effectiveness of a single manipulation procedure plus non-steroidal anti-inflammatory drugs (NSAIDs) to that of NSAIDs alone in 305 adults with temporomandibular joint disc displacement (closed lock). The total success rate for the manual therapy group during the entire follow-up time was 172/204 (84.3%) while the success rates in the control group were 0%. No formal comparisons between intervention and control groups were presented. In a low quality RCT, [115] 30 patients with myofascial pain lasting for at least six months were randomised to a single session of botulinum toxin injections or multiple session fascial manipulation (three 50 min sessions over two to four weeks). At three months follow-up, there were significant reductions in pain perception in both groups, but no significant difference between groups in most of the parameters measured. There was a tendency towards greater pain reduction in the manipulation group and greater increase in range of motion in the botulinum toxin group.

*Summary:* Results on the comparative effectiveness/safety of manual therapy for temporomandibular disorders remain **inconclusive (favourable) evidence** for mobilisation, massage, myofascial or osteopathic manipulation (no change from the UK evidence report).

#### Headache and other conditions

Table 6 provides a comparison between the overall evidence ratings included in the UK evidence report and new/additional studies in the current review for headache and other conditions.

**Table 6:** Comparison of evidence in UK evidence report and current review for headache and other conditions

**Condition**	**Intervention**	**UK evidence report evidence**			**New/additional evidence**			**New evidence?**
		**Inconclusive**	**Moderate**	**High**	**Inconclusive**	**Moderate**	**High**	
**Headache and other**								
Cervicogenic headache	Spinal manipulation		Positive			Positive		No
	Self-mobilising apophyseal glides		Positive			Positive		No
	Friction massage and trigger points	Non-favourable			Non-favourable			No
	Mobilisation	Unclear				Positive		Yes
Tension-type headache	Spinal manipulation	Unclear			Unclear			Yes
	Osteopathic care				Favourable			Yes
	Spinal mobilisation				Favourable			Yes
Miscellaneous headache	Mobilisation	Favourable				Positive		Yes
Cervicogenic dizziness	Self-mobilising apophyseal glides		Positive			Positive		No
	Manipulation/mobilisation				Favourable			Yes
Balance in elderly people	Diversified chiropractic care				Unclear			Yes
Fibromyalgia	Spinal manipulation	Unclear			Unclear			No
	Cranio-sacral therapy	Favourable			Favourable			Yes
	Massage-myofascial release therapy	Favourable			Favourable			Yes

##### 

**Cervicogenic headache** Two new high quality systematic reviews, [119,120] one high quality RCT [121] and one medium quality RCT [122] were identified on manual therapy for cervicogenic headache.

A high quality systematic review [120] evaluated the effects of spinal manipulative therapy on cervicogenic headache. The results from six of nine trials suggested that spinal manipulative therapy was more beneficial in treating the headaches compared to physical therapy, light massage, drug therapy, or no intervention. The remaining three trials showed no significant difference in headache intensity, duration, or frequency between spinal manipulative therapy and placebo, physical therapy, massage, or wait list controls. The systematic review by Chaibi et al. [119] did not include any new evidence in addition to the studies already identified and concluded that while the relevant RCTs suggested that physiotherapy and spinal manipulative therapy might be an effective treatment in the management of cervicogenic headache the studies were difficult to evaluate as only one included a non-treatment control group and most included participants with infrequent cervicogenic headache. One high quality RCT [121] compared the effects of temporomandibular manual therapy techniques plus cervical manual therapy to cervical manual therapy alone in 43 adults with cervicogenic headache. At 6 months of follow-up, the experimental group experienced significantly reduced headache intensity and temporomandibular measures (pain intensity during mouth opening, presence of deviation, and sounds) compared to the control. In a medium quality RCT, [122] 38 patients with recurrent headache and neck pain for at least two months (age 18 to 40 years) were randomised to mobilisation or massage (12 treatment sessions over 6 weeks for each treatment). Mobilisation involved low velocity/high amplitude oscillatory movements to the upper cervical vertebrae. Massage included myofascial release, manual cervical traction, trigger point therapy, facilitated stretching techniques. All participants also followed a programme of stretching and active exercises. In both groups, headache was significantly reduced after the intervention and function was significantly increased, but for most variables, the improvements were significantly greater in the cervical mobilisation group.

*Summary:***Moderate (positive) evidence** for mobilisation techniques in cervicogenic headache (change from inconclusive (unclear) evidence in the UK evidence report). **Inconclusive (non-favourable) evidence** for friction massage and trigger points in cervicogenic headache (no change from the UK evidence report).

##### 

**Tension-type headache** Four new and additional RCTs [123-127] (3 medium quality, [124-127] 1 low quality [123]) assessed the effects of manual therapy in tension-type headache. A new systematic review [128] did not include any evidence over and above the studies already considered by the UK evidence report and concluded that the evidence that spinal manipulation alleviates tension type headaches was encouraging, but inconclusive.

A low quality RCT [123] compared the effects of a direct versus an indirect myofascial release technique with control (soft stroking) in the treatment of tension-type headache. Sixty-three patients received one hour sessions twice a week for 12 weeks. Days with headache and headache frequency were reduced significantly more in both interventions groups than in the control group. There were no statistically significant differences between the two myofascial release groups and no serious adverse events. In their medium quality RCT, Anderson et al. [124] assessed the effect of adding osteopathic manual treatment to progressive muscular relaxation exercise compared to progressive muscular relaxation exercise alone in 29 patients with tension-type headache. Two weeks after the four week treatment, patients who received the combination treatment experienced a significantly reduced frequency of headache compared to patients assigned to progressive muscular relaxation exercise alone. The between-group differences for other headache parameters (headache rating, headache index, and headache intensity) were not statistically significant. In another medium quality RCT, [125,126] the effectiveness of manual therapy (cervical/thoracic spine mobilisation, exercises, postural correction) was compared to usual care by the general practitioner in 82 patients with chronic tension-type headache. Immediately after the end of treatment (at eight weeks), patients in the manual therapy group experienced significantly greater improvements in headache frequency, headache pain intensity, headache-related disability, cervical range of movement, and endurance of the neck flexor muscles, than the control group, but not in the use of pain medication. At 26 weeks of follow-up, the between-group differences were maintained significant only for headache frequency and headache pain intensity in favour of manual therapy. The medium quality RCT by Vernon et al. [127] compared the effectiveness of cervical manipulation, medical treatment (10–25 mg/day amitriptyline), and a combination of the two treatments in 20 adults with tension-type headache. The treatment duration was 14 weeks. There was a significant effect of the combination treatment compared to each treatment alone on headache frequency.

*Summary:***Inconclusive (favourable) evidence** for manual therapy (osteopathic care, spinal mobilisation) in treating tension-type headache (not evaluated in the UK evidence report). **Inconclusive (unclear) evidence** for spinal manipulation in treating tension-type headache (no change from the UK evidence report).

##### 

**Miscellaneous headaches** Two medium quality systematic reviews [129,130] and two medium quality RCTs [131,132] were identified on manual therapy for miscellaneous headaches.

The systematic review by Bryans et al. [129] investigated evidence on benefits and harms of manual therapy/chiropractic treatment in adults with miscellaneous headaches (migraine, tension-type headache, cervicogenic headache). The review included 21 relevant publications including the following: 11 randomised trials, 5 controlled trials, and 5 systematic reviews. The reviewed evidence indicated benefits of spinal manipulation for adults with episodic/chronic migraine and cervicogenic headache, but not for those with episodic tension-type headache. Evidence regarding benefits of spinal manipulation for chronic tension-type headache was inconclusive. Cranio-cervical mobilisation and joint mobilisation were shown to be of benefit for episodic/chronic tension-type headaches and cervicogenic headache, respectively. Evidence regarding benefits of manual traction, connective tissue manipulation, Cyriax’ mobilisation or exercise for tension-type headaches was inconclusive. Harms were adequately reported in only six trials and overall risks were low. Another systematic review [130] investigated if 6–12 visits to a chiropractor to receive spinal manipulative therapy or mobilisation would confer benefits for adults with headaches. The review included 47 randomised trials. The results did not support claims of restricting chiropractic care to 6–12 visits. The data indicated that a minimum of 24 visits would be needed to stabilise headaches.

A medium quality RCT [131] compared the effectiveness of 6-week manual therapy (combination of spinal mobilisation and stabilising exercise) plus usual care (education, prophylactic and attack medication) to that of usual care alone in 37 adults with miscellaneous headaches (tension-type, cervicogenic, migraine). There were no significant between-group differences in perceived effect, headache impact test-6, headache frequency, pain intensity, medication intake, and absenteeism at 26 weeks of follow-up. A pilot RCT of medium quality [132] compared the effects of manual therapy (Trager approach: gentle mobilisation of the joint areas of the head, neck, upper back, and shoulders), attention treatment (visit and discussion with physician about medication intake, previous week’s headaches, and perception of well-being), or no treatment (i.e., only medication group) in 33 participants taking pain medication for miscellaneous chronic headaches (i.e., tension-type, cluster, migraine). At 6 weeks of follow-up, both the manual therapy and attention groups experienced a significantly greater mean reduction (from baseline) in headache duration compared to the no treatment control group, as well as a greater improvement in quality of life. There was no significant difference between groups in post-treatment or between-group differences in mean change of medication use, headache intensity, and the number of headache episodes.

*Summary:***Moderate (positive) evidence** for manipulation and mobilisation for miscellaneous headache (change from inconclusive (favourable) evidence in the UK evidence report).

##### 

**Fibromyalgia** Three medium quality systematic reviews [133-135] assessed manual therapy in patients with fibromyalgia. However, two reviews [133,134] did not include studies not already included in the UK evidence report and both concluded that there is insufficient evidence to support the effectiveness of manual therapy in the treatment of fibromyalgia. The other medium quality review [135] only included three very small studies (<25 participants) on manipulative (chiropractic or osteopathic) therapy and concluded that there was not enough evidence for the effectiveness of manipulative therapy in fibromyalgia. Two new RCTs (1 medium quality, [136] 1 low quality [137]) not included in any systematic reviews were identified.

The medium quality RCT assessed the effects of cranio-sacral therapy in 92 women with fibromyalgia [136]. After 20 weeks of treatment, there was a significant improvement in the clinical global impression of improvement and the clinical global impression of severity and a significant reduction in pain at 13 of 18 tender points. However, most of these differences were not maintained one year after the treatment. The low quality RCT [137] assessed the effects of massage-myofascial release therapy in 59 patients with fibromyalgia. After 20 weeks of treatment, there was a significant improvement in pain (VAS), at 8 of 18 tender points, and four of eight quality of life domains (SF-36). Most of these changes were not maintained six months after the intervention.

*Summary:***Inconclusive (favourable) evidence** for the use of chiropractic spinal manipulation in fibromyalgia (no change from the UK evidence report). **Inconclusive (favourable) evidence** for effectiveness of cranio-sacral therapy and massage-myofascial release therapy for fibromyalgia.

#### Non-musculoskeletal conditions

Table 7 provides a comparison between the overall evidence ratings included in the UK evidence report and new/additional studies in the current review for non-musculoskeletal conditions.

**Table 7:** Comparison of evidence in UK evidence report and current review for non-musculoskeletal conditions

**Condition**	**Intervention**	**UK evidence report evidence**			**New/additional evidence**			**New evidence?**
		**Inconclusive**	**Moderate**	**High**	**Inconclusive**	**Moderate**	**High**	
Asthma	Spinal manipulation		Negative		Unclear			yes
	Osteopathic manual therapy	Favourable			Favourable			no
	Cranio-sacral therapy				Favourable			yes
Attention deficit hyperactivity disorder	Osteopathic treatment				Unclear			yes
Cancer care	Chiropractic care				Unclear			yes
	Massage including myofascial release/strain/counterstrain					Positive		yes
	Manipulation in osteosarcoma					Negative		yes
Cerebral palsy	Osteopathic manual therapy (cranio-sacral, cranial, myofascial release)				Unclear			yes
Chronic fatigue syndrome/myalgic encephalomyelitis	Osteopathic manual therapy				Favourable			yes
Chronic pelvic pain • interstitial cystitis/painful bladder syndrome/chronic prostatitis	Myofascial therapy				Favourable			yes
• chronic pelvic pain in women	Distension of painful pelvic structures				Favourable			yes
• chronic prostatitis/chronic pelvic pain/female urination disorders	Osteopathic manual therapy				Favourable			yes
Cystic fibrosis	Mobilisation				Unclear			yes
Paediatric dysfunctional voiding	Osteopathic manual therapy				Favourable			yes
Paediatric nocturnal enuresis	Spinal manipulation	Favourable			Favourable			no
Infant colic	Spinal manipulation		Negative		Favourable			yes
	Cranial osteopathic manual therapy	Favourable			Favourable			no
Dysmenorrhoea	Spinal manipulation		Negative			Negative		no
Premenstrual syndrome	Spinal manipulation	Unclear			Unclear			no
Menopausal symptoms	Fox’s low force osteopathic technique plus cranial techniques				Favourable			yes
Gastrointestinal disorders •reflux disease, duodenal ulcer	Spinal manipulation				Unclear			yes
• irritable bowel syndrome	Osteopathic manual therapy				Favourable			yes
Hypertension stage 1 hypertension	Spinal manipulation added to diet		Negative			Negative		no
	Upper cervical (NUCCA) spinal manipulation	Favourable			Favourable			no
	Instrument assisted spinal manipulation	Unclear			Unclear			no
	Osteopathic manual therapy				Unclear			yes
	Gonstead full spine chiropractic care				Unclear			yes
Intermittent claudication	Osteopathic manual therapy				Favourable			yes
Venous insufficiency	Myofascial release manual therapy combined with kinesiotherapy				Favourable			
Insomnia	Spinal manipulation				Unclear			yes
Otitis media	Osteopathic manual therapy	Unclear			Unclear			no
Parkinson’s disease	Osteopathic manual therapy				Favourable			yes
Pneumonia in elderly adults	Osteopathic manual therapy	Favourable			Favourable			no
Chronic obstructive pulmonary disease in elderly adults	Osteopathic manual therapy				Favourable			yes
Back pain during pregnancy	Spinal manipulation				Favourable			yes
Care during labour/delivery	Spinal manipulation				Unclear			yes
Care of preterm infants	Physiotherapeutic/osteopathic manual therapy				Unclear			yes
Surgery rehabilitation	Osteopathic manual therapy				Favourable			yes
Stroke rehabilitation	Mobilisation				Unclear			yes
Systemic sclerosis	McMennell joint manipulation				Unclear			yes

##### 

**Asthma** We identified three additional systematic reviews on manual therapy for asthma, [138-140] one additional medium quality RCT of cranio-sacral therapy for asthma in adults, [141] and one high quality qualitative study on complementary therapy use in patients with asthma [142]. Only one medium quality systematic review [139] included relevant studies over and above those already included in other reviews.

The review investigated chiropractic treatment for asthma and included eight studies, of which three were RCTs and one was a CCT, while the rest were uncontrolled studies. Three of the included studies were in children. In the comparative studies, no significant differences between comparison groups were seen in respiratory parameters, symptoms or subjective measures. In the uncontrolled studies, improvements were generally seen in subjective measures – however, improvements in subjective measures were also seen in the control groups of comparative studies. Only one study reported on adverse events (none reported). The review authors concluded that some patients may experience chiropractic care as beneficial, but overall there were no significant effects in any outcomes versus sham treatment. However, the quality of the evidence was generally poor and more evidence is required using valid and reliable outcome measurements.

The medium quality RCT [141] included 89 adults with asthma and compared the effects of cranio-sacral therapy only, acupuncture only, combined cranio-sacral therapy and acupuncture, attention control and waiting list control. The study was underpowered for this number of comparison groups and as no significant difference could be found between the intervention groups and between the control groups, intervention groups and control groups lumped together (i.e. no results were presented for cranio-sacral therapy alone). The intervention groups (acupuncture and/or cranio-sacral therapy) showed no significant difference to the control groups in pulmonary function measures or depression (Beck Depression Scale), however, medication use was significantly reduced both post-intervention and at six months follow-up in the intervention groups (i.e. the same lung function could be maintained at a lower level of medication use), and the Asthma Quality of Life score was significantly more improved post-intervention (not at six months follow-up) than in the control groups. An effect of provider continuity was also found, with the effects on quality of life being stronger in the groups having had 12 treatment sessions with a single provider, and with these groups also having a significantly reduced anxiety level (Beck Anxiety Interventory). No adverse effects were seen.

In the qualitative study, [142] 50 patients with asthma (21 adults and 29 children with their parents) were interviewed about their use of complementary therapies. Of these, 13 did not use complementary therapies. Reasons for non-use of complementary therapies included general scepticism, trust in conventional doctors, and not having tried any complementary therapies yet, despite being interested and open. The main complementary therapies used by the rest were breathing techniques (e.g. the Buteyko Method) and homeopathy, with some reported use of chiropractics, osteopathy and cranial osteopathy. Reasons for using complementary therapies included concerns about the side effects of conventional medications, medication dependency, and medication escalation (push factors). Pull factors included the desire for more natural or non-invasive treatments, the quality of the consultation (holistic approach, time taken, listening), a commitment to alternative philosophies of health, and experiences of effectiveness. Other important factors included the fact that complementary therapy use provided a greater scope for self-help and taking control, and that it allowed an exploration of a broader range of causes of asthma than conventional approaches. No specific statements on the views of manual therapy were offered.

*Summary:***Inconclusive (unclear) evidence** for use of spinal manipulation in treating asthma (change from moderate (negative) evidence in the UK evidence report). **Inconclusive (favourable) evidence** for osteopathic manual therapy in treating asthma (no change from UK evidence report). **Inconclusive (favourable) evidence** for cranio-sacral therapy in treating asthma (not evaluated in the UK evidence report).

##### 

**Attention Deficit/Hyperactivity Disorder (ADHD)/Learning disabilities** One medium quality systematic review [143] and two low quality RCTs [144,145] were identified on the use of manual therapy in children or adolescents with attention deficit/hyperactivity disorder (ADHD).

One systematic review [143] sought to assess the effects of chiropractic treatment in children or adolescents with ADHD. However, the authors found no studies fulfilling their inclusion criteria. The two low quality RCTs – that had very limited descriptions of study methodology and the study populations – both assessed the effects of osteopathic treatment of children with ADHD. Children had three [145] and four [144] osteopathic treatments separated by several weeks. Both trials reported improved outcomes on the ADHD Connors scale for the intervention group compared to the control group, however, no statistical analyses were reported.

*Summary:* Given the severe methodological limitations of the included studies, there is **inconclusive (unclear) evidence** regarding the effectiveness of osteopathic treatment for ADHD (not evaluated in the UK evidence report).

##### 

**Cancer care** One low quality systematic review [146] assessed chiropractic care of patients with cancer. No comparative studies were identified. While the review reports evidence that patients with cancer frequently consult chiropractors, no evidence regarding the effects of the chiropractic treatment was reported. Two high quality RCTs, [147,148] one medium quality, [149] and one low quality RCT [150] assessed the effects of manual therapy (myofascial release massage, mobilisation) in cancer patients. One moderate quality controlled cohort study [151] assessed adverse effects of manipulative therapy in patients with osteosarcoma.

Cantarero-Villanueva et al. [147] compared an eight week multimodal programme (core stability exercises and myofascial release massage, instruction DVD) with usual care in 78 breast cancer survivors. Immediately after treatment and at six months, fatigue, mood state, trunk curl endurance, and leg strength showed greater improvement in the experimental group compared to control group. Fernandez-Lao et al. [150] compared the effect of two 40 min sessions of myofascial release massage focussed on the neck-shoulder area with usual care plus 40 minutes attention control in 20 breast cancer survivors with cancer-related fatigue. After the intervention, a significant improvement was seen in the manual therapy group with respect to tension-anxiety, fatigue, and mood state in general compared to the control. Lopez-Sendin et al. [148] compared a physiotherapy intervention (six sessions of about 30 minutes for two weeks) involving several massage techniques and strain/counterstrain techniques over the tender points with a sham touch intervention in 92 patients with terminal cancer and pain. Risk areas were avoided. After the interventions, there was significantly greater improvement in some pain parameters and in mood in the intervention group than in the control group. A medium quality RCT (Pace do Amaral 2012) [149] examined the effects of manual therapy in combination with upper limb exercises with exercises alone for shoulder rehabilitation in 131 women after surgery for breast cancer. Manual therapy consisted in mobilisations and massage. Patients received 12 ± 2 sessions over one month. Shoulder range of motion and function were significantly improved in both groups, but there was no significant difference between groups.

With respect to adverse events, one moderate quality controlled cohort study (Wu 2010) [151] assessed the prognosis of patients with osteosarcoma who had or had not had manipulative therapy (patients had sought manipulative therapy because of non-specific symptoms, not for cancer treatment). Tumour characteristics and demographic characteristics were similar between the two groups, however, the patients who had received manipulative therapy had a significantly worse prognosis over the 42 to 50 month follow-up period than the non-manipulation group (lower survival rate, more lung metastases, more local recurrence).

*Summary:***Moderate (positive) evidence** for the effectiveness of massage techniques involving manual therapy elements in breast cancer survivors and terminal cancer patients but **inconclusive (unclear) evidence** for use of chiropractic care for cancer care (not evaluated in the UK evidence report). In some types of cancer such as osteosarcoma, manipulative therapy may have significant adverse effects and was contraindicated.

##### 

**Cerebral palsy in children** Three RCTs (1 low quality, [152] two medium quality [153,154]) were identified that assessed the effects of osteopathy in children with cerebral palsy. One of the trials was low quality and two were medium quality. A systematic review [155] on interventions assessing sleep quality in children with cerebral palsy did not include any studies over and above those already reviewed.

A low quality trial [152] assessed the effects of osteopathy (cranio-sacral and myofascial release techniques) versus acupuncture and attention control in 50 children with cerebral palsy. Outcomes were based on parents’ perceptions only (and parents were not reported to have been blinded). Statistical differences between groups were not reported. Most improvements were seen in leg or hand use and in sleep, and these appeared similar between the two intervention groups. Improvements in speech/drooling and cognition appeared to be more for the acupuncture group than the osteopathy group, while there were similar improvements in mood. The sample number was small and the significance of any differences between groups remained unclear. The second trial [153] was medium quality and again compared osteopathy with acupuncture or attention control in 55 children with cerebral palsy. Osteopathy consisted of direct or indirect techniques in the cranial field and/or myofascial release (10 sessions over 24 weeks), compared with 30 sessions of acupuncture (scalp, body and auricular acupuncture). No significant effects of acupuncture were seen for any of the gross motor function or disability outcomes, while osteopathy resulted in a significant effect for two of the six gross motor and disability outcomes assessed (Gross Motor Function Measurement percent and Functional Independence Measure for Children mobility). The medium quality RCT by Wyatt et. al. [154] compared the effects of six sessions of cranial osteopathy with an attention control group in 142 children with cerebral palsy. After six months, there were no significant differences between the two groups in gross motor function or quality of life. Similarly, there were no significant differences regarding sleep-related parameters, parental assessment of the child’s pain and main carer’s quality of life. However, significantly more parents in the osteopathy group rated their child’s global health as ‘better’ after six months than in the control group – but parents were not blinded to the intervention condition.

*Summary:***Inconclusive (unclear) evidence** for the effectiveness of osteopathic manual therapy in the treatment of cerebral palsy (not evaluated in the UK evidence report).

##### 

**Cervicogenic dizziness/balance** One high quality systematic review was identified on the effects of manual therapy with or without vestibular rehabilitation in the management of cervicogenic dizziness, [156] as well as one low quality RCT on the effects of chiropractic care in elderly adults with impaired balance [157] and a protocol of an ongoing trial on the effects of manual therapy treatments for people with cervicogenic dizziness and pain [158].

The high quality systematic review [156] included five RCTs (three of these were Chinese studies) and eight non-controlled cohort studies. One of the RCTs was good quality, while the rest were moderate quality. Six of the studies (two RCTs) used manipulation/mobilisation only as an intervention, while the rest used a multimodal approach. None of the trials used a vestibular rehabilitation intervention. Twelve studies (including all RCTs) found an improvement in dizziness and associated symptoms after manual therapy, and two of the RCTs found an improvement in balance performance. Adverse events were only reported by three studies, but two of these found no adverse events and one only minor ones. The review authors concluded that there is moderate evidence in a favourable direction to support the use of manual therapy (spinal mobilisation and/or manipulation) for cervicogenic dizziness but that research is needed on combining manual therapy with vestibular rehabilitation.

A low quality RCT [157] compared the effect of a limited or extended course of chiropractic care on balance, chronic pain, and associated dizziness in 34 older adults with impaired balance. In the limited chiropractic care group, patients were treated twice a week for eight weeks using the diversified technique (manipulation, soft tissue treatments, hot packs), in the extended schedule group patients received additional monthly treatments for ten months. Outcome reporting of falls in this study were unreliable as patients were asked at each treatment/assessment visit there were unequal numbers of visits between groups and patients with more visits reported more falls. There was no significant difference between groups in scores on the Berg Balance Scale, depression, the Pain Disability Index, or dizziness.

*Summary:***Inconclusive (favourable) evidence** for the effectiveness of manipulation/mobilisation for cervicogenic dizziness (not evaluated in the UK evidence report). **Inconclusive (unclear) evidence** for diversified chiropractic treatment in the improvement of balance in elderly people (not evaluated in the UK evidence report).

##### 

**Chronic fatigue syndrome/myalgic encephalomyelitis** One high quality systematic review was identified that studied the effects of alternative medical interventions (including manual therapy) on patients with chronic fatigue syndrome or fibromyalgia [134]. The authors identified one low quality RCT assessing the effects of osteopathic manual therapy in 58 patients with myalgic encephalomyelitis. In that trial there was a significant improvement in symptoms in the intervention group but not in the control group (significant difference between groups).

*Summary:***Inconclusive (favourable) evidence** for osteopathic manual therapy improving symptoms of myalgic encephalomyelitis (not evaluated in the UK evidence report).

##### 

**Chronic pelvic pain** Two high quality systematic reviews [159,160] and three RCTs (1 medium quality, [161,162] and 2 low quality [163,164]) were identified that assessed the effects of manual therapy in chronic pelvic pain.

A high quality systematic review [159] assessed the effects of osteopathic manual therapy on female urination disorders. The review included two RCTs and three CCTs of osteopathic treatment for female urinary incontinence or voiding disorder. All studies had a high risk of bias. There was a significant therapeutic effect of osteopathic treatment (outcome not reported) when compared with a no treatment control group, but no difference when compared with pelvic floor muscle training. Another high quality systematic review [160] examined the effects of physiotherapy management (including manual therapy) in women with chronic pelvic pain. Three RCTs of adequate quality relevant to manual therapy were included. There was level 1d evidence (high risk of bias) that physiotherapeutic distension of painful pelvic structures combined with pain counselling improves pain experience compared with usual treatment.

One medium quality RCT [161,162] compared the effects of 10 weeks of myofascial physical therapy or general full body Western massage in 47 adults with interstitial cystitis/painful bladder syndrome or men with chronic prostatitis/chronic pelvic pain. Overall, significantly more patients had moderate or marked symptom improvement with myofascial therapy than with massage therapy (57% versus 21%, ‘responders’). When considering the subgroups with interstitial cystitis/painful bladder syndrome or with chronic prostatitis/chronic pelvic pain, a significant difference between groups was only seen for the former (50% versus 7%, p = 0.03), while a substantial proportion of the latter were also ‘responders’ to massage therapy (64% myofascial therapy, 40% massage therapy). Significantly more improvement was seen for both the Interstitial Cystitis Symptom and Problem Index for the myofascial therapy group than the massage group, while there was no difference in urinary frequency or urgency, sexual function, pain, or quality of life (SF-12). A low quality RCT [163] compared the effects of distension of painful pelvic structure (two sessions) in 50 women with chronic pelvic pain with a counselling control group. At the end of the treatment, the intervention group had significantly reduced pelvic pain, painful intercourse, low back pain, sleep disturbance, sleep quality, mental fatigue, and anger than the control group. There was no significant difference in depression or mood. Another low quality RCT [164] compared the effects of eight weeks of osteopathic care with a simple exercise control group in 35 men with chronic prostatitis/chronic pelvic pain syndrome. Six weeks after the last treatment, the osteopathy group had had a significantly improved International Prostate Symptom Score, Chronic Prostatitis Symptom Index, and quality of life score compared to the control group.

*Summary:***Inconclusive (favourable) evidence** for the use of osteopathic treatment in female urination disorders (not evaluated in UK evidence report). **Inconclusive (favourable) evidence** for the use of myofascial therapy in interstitial cystitis/painful bladder syndrome or chronic prostatitis/chronic pelvic pain (not evaluated in the UK evidence report). **Inconclusive (favourable) evidence** for distension of painful pelvic structures in chronic pelvic pain in women and for osteopathic manual therapy in men with chronic prostatitis/chronic pelvic pain (not evaluated in the UK evidence report).

##### 

**Cystic fibrosis** One small medium quality RCT assessed the effects of musculoskeletal treatments including mobilisations to the rib cage and thoracic spine in 20 adults with cystic fibrosis [165]. Patients in the intervention group received six treatment sessions, patients in the control group received usual care only. After 12 weeks, there were no significant differences between groups in pain or FEV1. However, quality of life had increased significantly more in the intervention group than in the control group.

*Summary:***Inconclusive (unclear) evidence** for the use of mobilisations (rib cage and thoracic spine) in patients with cystic fibrosis (not evaluated in the UK evidence report).

##### 

**Paediatric dyfunctional voiding** One low quality RCT was identified that assessed manual therapy in paediatric dysfunctional voiding [166]. Children (n = 21) with vesicoureteral reflux and/daytime incontinence were randomised to standard therapy or standard therapy plus four sessions of manual physical therapy based on an osteopathic approach. Overall, children who received osteopathic manual therapy had significantly more (p = 0.008) improvement of symptoms after 10 weeks of treatment than children in the control group, however, significance was not quite reached in subgroups with vesicoureteral reflux only or with daytime incontinence only (possibly partially due to small numbers). Adverse effects were not assessed.

*Summary:***Inconclusive (favourable) evidence** for osteopathic manual therapy improving symptoms of paediatric dysfunctional voiding (not evaluated in the UK evidence report).

##### 

**Gastrointestinal disorders** One additional medium quality systematic review [167] and two additional low quality RCTs [168,169] were identified that investigated manual treatment for gastrointestinal disorders.

The systematic review [167] included one RCT and one CCT that reported the effects of chiropractic spinal manipulation in patients with gastroesophageal reflux disease and duodenal ulcer. Given the paucity and low quality of the reviewed evidence, the review could not draw any definitive conclusions regarding the effects of spinal manipulation versus ischaemic compression or conventional treatment.

One additional low quality RCT assessed the benefits and harms of osteopathy compared to standard care at 1, 3, and 6 months of post-baseline follow-up for 39 patients with irritable bowel syndrome [169]. The post-treatment change at 6 months was statistically significant in favour of osteopathy versus standard care for overall/global assessment, Functional Bowel Disorder Severity Index score, and quality of life. Similarly, the end-point mean symptom score was significantly reduced in favour of the osteopathy over standard care group. There was no occurrence of adverse events. A low quality RCT [168] compared two sessions (at 0 and 7 days) of osteopathy with sham osteopathy in 30 patients with irritable bowel syndrome. The severity of irritable bowel syndrome decreased in both groups, but at day 7 the decrease was significantly more marked in the osteopathy group. However, there was no significant difference between the groups at the one month follow-up.

*Summary:***Inconclusive (unclear) evidence** for spinal manipulation for gastrointestinal disorders (not evaluated in the UK evidence report). **Inconclusive (favourable) evidence** for osteopathic manual therapy for irritable bowel syndrome (not evaluated in the UK evidence report).

##### 

**Hypertension** We identified one new medium quality systematic review [170] and one additional medium quality non-randomised clinical trial not included in any systematic review [171] on the use of manual therapy in the treatment of hypertension.

A systematic review [170] examined the effects of spinal manipulative therapy on hypertension. Results of five RCTs using a variety of spinal techniques were reported (Gonstead chiropractic adjusting, NUCCA technique, "diversified adjustments", Activator instrument, and osteopathic manipulative therapy). Two included trials with a low risk of bias found no significant differences for diversified adjustments plus diet versus diet only or of Gonstead chiropractic adjusting versus brief massage or control on systolic or diastolic blood pressure (however, the trial of Gonstead chiropractic care had a very small sample size). Of three trials with unclear risk of bias, two (both using largely only a single adjustment) found a significantly greater reduction of both systolic and diastolic blood pressure with spinal manipulation using the Activator instrument or the NUCCA technique versus control, while one trial found no significant difference in a cross-over trial between the effects of osteopathic manipulative therapy and sham massage on blood pressure.

A non-randomised clinical trial [171] examined the effects of biweekly osteopathic manipulative therapy plus pharmacological treatment versus pharmacological treatment only on blood pressure and intima media thickness (femoral and carotid bifurcation) over 12 months in 63 patients with hypertension. After adjusting for a range of confounding factors, osteopathic treatment was significantly associated with both a larger decrease in systolic blood pressure and in intima media thickness than pharmacological treatment alone.

*Summary:***Inconclusive (favourable) evidence** for upper cervical NUCCA manipulation for stage 1 hypertension (no change from the UK evidence report). **Inconclusive (unclear) evidence** for instrument assisted spinal manipulation for hypertension (not evaluated in the UK evidence report). **Inconclusive (unclear) evidence** for effectiveness of Gonstead full spine chiropractic care or osteopathic manipulative therapy for hypertension (not evaluated in the UK evidence report).

##### 

**Infantile colic** Four potentially relevant new systematic reviews [138,172-174] including manual treatments for infant colic were identified. Three of the systematic reviews did not include any new studies not already considered by the UK evidence report or eligible according to the inclusion criteria of the current review [138,172,174]. A high quality Cochrane review included six relevant RCTs [173]. Overall, the associated meta-analysis found a significant reduction in crying time with manipulative treatment. However, the studies included in the review were generally small and methodologically prone to bias, and the authors concluded that it was not possible to arrive at a definitive conclusion about the effectiveness of manipulative therapies for infantile colic. The review also included a new high quality RCT [175] of chiropractic manual therapy that found reduced crying time in the treated infants, irrespective of parent blinding.

*Summary:***Inconclusive (favourable) evidence** for cranial osteopathic manual therapy in treating infantile colic. **Inconclusive (favourable) evidence** for spinal manipulation in treating infantile colic (change from moderate (negative) evidence reported in the UK evidence report).

##### 

**Insomnia** One low quality systematic review [176] assessed the effects of chiropractic spinal manipulative therapy on primary insomnia. No relevant controlled studies were identified (the only controlled study mentioned was in fact of healthy volunteers (not mentioned by the reviewers) and thus no relevant outcomes were reported).

*Summary:***Inconclusive (unclear) evidence** on the benefits of manual therapy in people with primary insomnia (not evaluated in the UK evidence report).

##### 

**Menopausal symptoms** One small low quality RCT [177] assessed the effects of Fox’s low force osteopathic technique and cranial methods in the treatment of menopausal symptoms in 30 women aged between 50 and 60 years, compared to a placebo procedure. The treatment was applied once a week for 10 weeks and follow-up was at 15 weeks. Four of six menopausal symptoms were improved in the intervention group after the end of the intervention period compared to control, and three were reduced after the five week follow-up period. At the follow-up, there was also a significant reduction in neck pain compared to control in those patients who had had chronic neck pain at the start of the trial; the difference was nearly significant for back pain (small numbers).

*Summary:***Inconclusive (favourable) evidence** for the effectiveness of combined use of Fox’s low force osteopathic techniques and cranial techniques in the treatment of menopausal symptoms (not evaluated in the UK evidence report).

##### 

**Myofascial pain syndrome** Two additional medium quality systematic reviews assessing the effectiveness of manual therapy in myofascial pain syndrome were identified [178,179]. However, no review included any trials over and above those mentioned in the UK evidence report. Three additional medium quality RCTs [180-182] and one low quality RCT [183] were identified on the effects of manual therapy in people with myofascial pain.

Two trials [180,181] only assessed outcomes immediately after a single treatment and therefore longer term effects are unclear. In the first trial, [180] the effects of ischaemic compression therapy with trigger point therapy using the Activator instrument in 52 participants were compared with active upper trapezius trigger points. Improvements were seen in both groups on pain, pressure pain threshold and a global impression of improvement, but there was no significant difference between the two intervention groups. In the second trial, [181] the effects of ischaemic compression, trigger point pressure release, and sham treatment in 45 patients with sub-acute mechanical neck pain were compared with active upper trapezius trigger points. After the intervention, there was no significant difference between the three groups in neck pain, pressure pain threshold or lateral cervical flexion. However, there were significantly more participants in the ischaemic compression group who reported an improvement (pain reduction of at least 20 mm (VAS)) than in the sham group. None of the two trials reported on adverse events. In another trial, [182] 60 patients with non-specific sub-acute neck pain and active upper trapezius trigger points were treated 12 times over a period of four weeks using a muscle energy technique or an integrated neuromuscular inhibition technique (ischaemic compression plus strain-counterstrain plus muscle energy technique). After the intervention, participants in the integrated neuromuscular inhibition group had significantly better outcomes for pain, neck disability and lateral cervical flexion than participants in the muscle energy group. The authors did not report on adverse events. Sarrafzadeh et al. [183] compared the effects of pressure release with those of phonophoresis of hydrocortisone or ultrasonic therapy (six sessions for each treatment) in 60 women with latent upper trapezius myofascial trigger points. After the treatment, pain intensity, pain pressure threshold and active cervical lateral flexion were significantly more improved in the pressure release and phonophoresis groups than in the ultrasound group.

*Summary:***Inconclusive (favourable) evidence** for ischaemic compression (manual or using an Activator instrument) in the deactivation of upper trapezius trigger points (not evaluated in the UK evidence report). **Inconclusive (non-favourable) evidence** indicating that trigger point release is not as effective as ischaemic compression in deactivating active upper trapezius trigger points and improving associated neck pain (not evaluated in the UK evidence report). **Inconclusive (favourable) evidence** for an integrated neuromuscular inhibition technique in the management of neck pain with active upper trapezius trigger points (not evaluated in the UK evidence report).

##### 

**Otitis media** One new high quality systematic review was identified on the treatment of otitis media in children with spinal manipulative therapy [184]. One ongoing trial was identified on a five week standardised osteopathic manipulative medicine protocol plus standard care compared to standard care only in children between six months and two years with acute otitis media [185]. One systematic review [138] did not include any evidence over and above that already reviewed.

The review by Pohlmann et al. [184] summarised 49 studies of all types (including four clinical trials) but only limited quality evidence was identified and the authors concluded that there was currently no evidence to support or refute using spinal manipulative therapy for otitis media and no evidence to suggest that spinal manipulative therapy produces serious adverse effects for children with otitis media.

*Summary:***Inconclusive (unclear) evidence** for osteopathic manual therapy in treating otitis media (no change from the UK evidence report).

##### 

**Parkinson’s disease** One small low quality controlled trial [186] assessed the effect of a single 30 minute session of osteopathic manual therapy on gait performance in patients with Parkinson’s disease. Gait parameters were significantly improved in comparison to the control group, but no other patient-relevant outcomes were assessed and long term effects of osteopathic manipulation in Parkinson’s disease remain unclear. Adverse effects were not assessed. Additionally, an ongoing trial of different rehabilitation programmes (involving joint mobilisation) in Parkinson’s disease was identified [187].

*Summary:***Inconclusive (favourable) evidence** for the effectiveness of osteopathic manual therapy in Parkinson’s disease (not evaluated in the UK evidence report).

##### 

**Paediatric nocturnal enuresis** One high quality new systematic (Cochrane) review was identified that assessed the effects of complementary and miscellaneous interventions (including chiropractic) for nocturnal enuresis in children [188]. However, the review did not include any new trials fulfilling our inclusion criteria that were not already considered by the UK evidence report. Another new high quality systematic review of the use of chiropractic spinal manipulation in paediatric health conditions [138] also did not include any relevant trials not already included in the UK evidence report.

*Summary:***Inconclusive (favourable) evidence** for spinal manipulation in paediatric nocturnal enuresis (no change from the UK evidence report).

##### 

**Peripheral arterial disease** One medium quality RCT [189] was identified that assessed the effects of myofascial release manual therapy combined with kinesiotherapy compared to kinesiotherapy alone in 65 postmenopausal women with venous insufficiency. After 10 weeks of treatment (20 sessions of myofascial release manual therapy), there were significant improvements in basal metabolism, intracellular water, diastolic blood pressure, venous blood flow velocity, pain, and emotional role in the myofascial therapy group compared to control.

One medium quality non-randomised controlled trial was identified of osteopathic manipulative therapy in patients with intermittent claudication [190]. Thirty male patients were treated for six months with a variety of osteopathic manual techniques plus standard pharmacological treatment or standard pharmacological treatment only. After the six months, patients in the intervention group had significantly improved values for the ankle-brachial pressure index at rest and after exercise, claudication pain time and total walking time on a treadmill, with no significant changes occurring in the control group (difference between groups not reported – these were presumably insignificant). Four of eight quality of life measures were significantly more improved in the intervention group than in the control group (physical function, role limitations/physical, bodily pain, general health); there were no significant differences in mental health, role limitations/emotional, social function or vitality.

*Summary:***Inconclusive (favourable) evidence** for the effectiveness of osteopathic manual therapy in the treatment of intermittent claudication and of myofascial release manual therapy combined with kinesiotherapy in the treatment of venous insufficiency.

##### 

**Pneumonia and COPD** One high quality Cochrane review [191] was identified that assessed the effects of chest physiotherapy in adults with pneumonia, as well as a medium quality systematic review of manual therapy for COPD [192] which also included a new medium quality RCT [193] of osteopathic manipulative treatment in elderly patients with chronic obstructive pulmonary disease (COPD). Additionally, we identified a new high quality RCT of osteopathic manual treatment in severe COPD [194] which was not included in any of the reviews. There was also an ongoing RCT of osteopathic manipulative treatment in elderly patients with pneumonia [195]. Another systematic review [140] did not include any further eligible studies relevant to this section.

A Cochrane review [191] included two RCTs of osteopathic manipulative therapy for adults with pneumonia. Both included a standardised osteopathic manipulative treatment protocol versus sham (light touch) treatment which was applied twice a day for 10 to 15 minutes during the hospital stay in 21 and 58 patients with a mean age of 77 to 82 years. There was no significant effect of osteopathic treatment on mortality, cure rate, duration of fever, rate of improvement of chest X-ray, or duration of oral antibiotic therapy. Hospital stay in the osteopathy group was significantly reduced by two days compared to control and both the duration of total antibiotic therapy and intravenous therapy were reduced by about two days in the osteopathy versus control groups. The review authors concluded that osteopathic manipulative therapy may reduce the mean duration of hospital stay and antibiotic treatment but that further high quality evidence was needed before chest physiotherapy could be recommended as an adjunct to conventional therapy in pneumonia in adults. An ongoing RCT [195] used a second control group on conventional therapy only. Another systematic review [192] included seven studies (five RCTS) of manual therapy for COPD, six of which were rated high risk of bias and one low risk of bias. Four studies included osteopathic spinal manipulation, one used massage, one muscle stretching, and one passive movements. Comparison was against routine management, light touch or no intervention. After the osteopathic interventions, changes in respiratory parameters were variable, but an improvement was generally seen in subjective parameters. The authors concluded that there was no evidence to support or refute the use of manual therapy techniques in clinical practice to improve lung function in COPD patients.

The trial by Zanotti et al. [194] compared pulmonary rehabilitation (exercise training, educational support, psychological counselling, nutritional intervention) plus soft manipulation with pulmonary rehabilitation plus osteopathic manipulative treatment in 20 patients with stable severe COPD. Treatment was given five days a week for four weeks. After the treatment, exercise capacity (six minute walk test) improved significantly in both groups, but significantly more in the group receiving osteopathic treatment. Furthermore, there was a significant reduction in residual volume in the osteopathy group (significant difference to control).

*Summary:***Inconclusive (favourable) evidence** for osteopathic manipulative treatment of pneumonia in older adults (no change from the UK evidence report). **Inconclusive (favourable) evidence** for osteopathic manipulative treatment in patients with COPD (not evaluated in the UK evidence report).

##### 

**Pregnancy/obstetric care/neonatal care** Two systematic reviews (1 medium quality, [196] 1 high quality [197]) and three medium quality primary controlled studies [198-200] were identified that reported on the effectiveness of manipulative therapy used in pregnancy, obstetric and/or neonatal care settings, as well as a protocol of an ongoing trial on osteopathic manipulative treatment in neonatal intensive care units [201].

One systematic review of medium quality [196] evaluated the evidence on the effects of spinal manipulative therapy on back pain and other symptoms related to pregnancy. The review identified 32 relevant publications, most of which were non-randomised and uncontrolled and their results supported that the use of spinal manipulative therapy during pregnancy was associated with reduced back pain. Evidence regarding labour, delivery and adverse events were insufficient to be conclusive. The authors concluded that since there is a limited number of effective treatments for pregnancy-related back pain, clinicians might consider spinal manipulative therapy as a treatment option, if no contraindications are present. A high quality Cochrane review [197] assessed the effectiveness of massage, reflexology and other manual methods for pain management in labour. However, all studies included were of massage therapy and do therefore not fulfill the inclusion criteria of the current review.

The medium quality RCT [199] randomised 57 pregnant women with low back pain to exercise, spinal manipulation, or Neuro-Emotional Technique (treatment once monthly until 28 weeks gestation, twice monthly until 36 weeks gestation, and weekly thereafter). At least 50% of participants in each treatment group experienced clinically meaningful improvement in symptoms for the Roland Morris Disability Questionnaire. Also at least 50% of the exercise and spinal manipulation participants experienced clinically meaningful improvement in pain. However, there were no significant differences between groups in any of the outcomes. In a medium quality RCT, [198] 72 very preterm (gestational age <32 weeks) infants born with very low birth weight (< 1500 g) were randomised to receive developmental physical therapy (34 infants) or no physical therapy (38 infants) for 4 months. The Alberta Infant Motor Scale (AIMS) was used to assess the effects of physical therapy on motor development in the infants at 4 months post-randomisation. At the 4-month assessment, there were no significant differences on AIMS between the treatment and no treatment groups (the median percentile rank: 65 versus 72.5, p = 0.191). In a medium quality comparative cohort study of 350 preterm infants during hospitalisation, [200] the authors investigated the effect of osteopathic manipulative treatment on gastrointestinal function and length of hospital stay. Osteopathic manipulative treatment in addition to conventional care was compared to conventional care alone. The results indicated that the infants who had received osteopathic manipulative treatment were at significantly lower risk for having daily gut symptoms as well as having reduced lengths of hospital stay compared to the control group.

*Summary:***Inconclusive (favourable) evidence** for spinal manipulative therapy for back pain during pregnancy (not evaluated in the UK evidence report). **Inconclusive (unclear) evidence** for manual therapy during labour or delivery (not evaluated in the UK evidence report). **Inconclusive (unclear) evidence** for manual therapy in the care of preterm infants (not evaluated in the UK evidence report).

##### 

**Rehabilitation** There were three new and additional RCTs (1 low quality, [202] 2 medium quality [203,204]) and three non-randomised studies [205-207] (2 low quality cohort studies, [205,207] 1 medium quality cohort study [206]) on manual treatments in the rehabilitation of non-musculoskeletal disorders. Five studies enrolled post-surgery adults receiving manual therapy as part of rehabilitation process. In these studies, participants had undergone cholecystectomy, [204] abdominal hysterectomy, [202] abdominal surgery, [205] knee/hip arthroplasty [206] and coronary artery bypass graft (CABG) surgery [207]. In one study, the participants received manual therapy as a post-stroke rehabilitation treatment [203].

A medium quality RCT [203] randomised 76 adults after a stroke to receive conventional physiotherapy alone or with additional three different doses of 30, 60, or 120 minutes of manual therapy (joint/soft tissue mobilisation, massage, tactile stimulation, active-assisted movements, soft tissue stretch, and/or compression) for two weeks. No statistically significant differences in either post-treatment Motricity Index or Action Research Arm Test were observed across the control (conventional physiotherapy alone) and three treatment groups. There was no occurrence of adverse events. Sleszynski et al. [204] randomised 42 adults who had had cholecystectomy to receive a form of spinal manual therapy (i.e., thoracic lymphatic pump) or incentive spirometry (IS) and compared the mean forced vital capacity, forced expiratory volume, and incidence of atelectasis (complication of abdominal surgery) between the two treatments Medium quality trial). The 5-day post-treatment frequency of atelectasis was similar in the two treatment groups. There was a faster recovery of forced vital capacity and forced expiratory volume in participants receiving the manual therapy versus incentive spirometry. A low quality RCT, [202] randomised 39 women after post-abdominal hysterectomy to receive placebo (pre- and post-operative), osteopathic manual therapy (post-operative), morphine (pre-operative), or the combination of morphine (pre-operative) and osteopathic manual therapy (post-operative). There were no significant between-group differences in pain, nausea, or vomiting mean scores at any time of the 48-hour follow-up post-surgery. Total 24-hour post-operative morphine dose was significantly lower in the pre-operative morphine plus post-operative osteopathic manual therapy and in the osteopathic manual therapy groups compared to the pre-operative morphine alone group.

A retrospective cohort study of low quality explored the effect of osteopathic manipulative treatment on the length of hospital stay in adults who had developed ileus after abdominal surgery [205]. The records of 331 post-abdominal surgery participants with diagnosis of ileus were identified and divided into groups: a) patients who had received osteopathic manipulative treatment and b) patients who had not received osteopathic manipulative treatment. The results indicated a significantly shorter stay for the osteopathic manipulative treatment recipient group versus the control group. Yurvati et al. [207] conducted a cohort study to determine the effects of osteopathic manipulative treatment on cardiac haemodynamics in 29 adults after coronary artery bypass graft surgery. The treatment group consisted of 10 participants treated with osteopathic manipulative treatment after surgery and the control group, identified through a chart review, consisted of 19 subjects who underwent surgery but were not treated with post-surgery osteopathic manipulative treatment. The treatment and control post-surgery groups were compared with respect to changes in mixed venous oxygen saturation and cardiac index. This study was judged to be of low quality. Mean mixed venous oxygen saturation and cardiac index improved significantly more in the osteopathic manipulative treatment group compared to control. In another cohort study of medium quality, [206] the authors assessed the effects of osteopathic manipulative treatment on distance walked, days to independent negotiation of stairs, length of hospital stay, need for supplemental analgesics, and perception of pain in 76 adult participants who had knee or hip arthroplasty. The post-operative mean number of days to independent negotiation of stairs in the osteopathic manipulative treatment group was significantly shorter compared to the control group. There was no statistically significant difference in the distance ambulated, length of hospital stay, and need for supplemental analgesics.

*Summary:***Inconclusive (favourable) evidence** for osteopathic manual therapy for surgery rehabilitation (not evaluated in the UK evidence report – except for knee/hip arthroplasty). **Inconclusive (unclear) evidence** for mobilisation for stroke rehabilitation (not evaluated in the UK evidence report).

##### 

**Systemic sclerosis** Two small low quality RCTs by the same research group, [208,209] examined the use of McMennell joint manipulation within the context of a comprehensive rehabilitation programme for patients with systemic sclerosis.

The emphasis was on hand involvement, although one of the studies also examined parameters related to face involvement. Both trials did not report any formal comparisons between intervention and control groups. In both trials, some mobility parameters (Hand Mobility in Scleroderma Test) were improved both after the nine week intervention and after a nine week post-intervention follow-up. Some quality of life measures (SF-36) were only improved after the intervention but not at the nine week follow-up. In one trial, disability measures were improved in the intervention group both after the intervention and at follow-up, while in the other trial the disability improvement did not persist at the follow-up measurement. However, as these results were not statistically compared with those of the comparison group (results reported as unchanged) any benefits of the intervention have to remain unclear.

*Summary:***Inconclusive (unclear) evidence** for McMennell joint manipulation used in a complex rehabilitation programme in systemic sclerosis (not evaluated in the UK evidence report).

No new or additional studies were found for the following conditions: coccydynia, dysmenorrhoea, premenstrual syndrome.

##### 

**Adverse events** Seven systematic reviews [24,25,28,29,210-213] and seven primary studies [214-220] were identified specifically concerning adverse events of manual therapy. Mild-to-moderate adverse events of transient nature (e.g., worsening symptoms, increased pain, soreness, headache, dizziness, tiredness, nausea, vomiting) were relatively frequent. For example, evidence from high, medium, and low quality systematic reviews specifically focussing on adverse events suggested that approximately half of the individuals receiving manual therapy experienced mild-to-moderate adverse event which had resolved within 24–74 hours. In agreement with the UK evidence report, evidence indicated that serious (or major) adverse events after manual therapy were very rare (e.g., cerebrovascular events, disc herniation, vertebral artery dissection, cauda equine syndrome, stroke, dislocation, fracture, transient ischemic attack). Evidence on safety of manual therapies in children or paediatric populations was scarce; the findings from two low quality cohort studies and one survey were consistent with those for adults that transient mild to moderate intensity adverse events in manual treatment were common compared to more serious or major adverse events which were very rare. However, the evidence on adverse events in manual therapy warrants caution due to relative paucity of evidence and poor methodological quality of the included primary studies.

## Discussion

The current report summarised new and additional systematic reviews, RCTs and non-randomised primary studies not included by Bronfort et al. [20] focussing on conditions/interventions with ‘inconclusive’ or negative’ evidence ratings in the UK evidence report, or those not included. 178 studies were included. The most common study design was the RCT. There were relatively few non-randomised comparative and qualitative studies meeting the current inclusion criteria.

The majority of conditions previously reported to have ‘inconclusive’ evidence ratings by Bronfort remained the same. Evidence ratings changed in a positive direction from inconclusive to moderate (positive) evidence ratings in only three cases (manipulation/mobilisation [with exercise] for rotator cuff disorder, spinal mobilisation for cervicogenic and mobilisation for miscellaneous headache). New moderate (positive) evidence was identified for soft tissue shoulder disorders using myofascial treatments (ischaemic compression, deep friction massage, therapeutic stretch) not reported in the UK evidence report. In addition, evidence was identified on a large number of non-musculoskeletal conditions that had not previously been considered by Bronfort, most of this evidence was rated as inconclusive; although moderate (positive) evidence was identified for the use of massage including myofascial release/strain/counterstrain for cancer care.

Despite a noted shortfall in the quality of the evidence, the current review also supported the "moderate (positive)" evidence ratings by Bronfort for the use of:

• Manipulation/mobilisation (with movement) for shoulder girdle pain/dysfunction;

• High grade mobilisation for adhesive capsulitis;

• Myofascial treatments (ischaemic compression, deep friction massage, therapeutic stretch) for soft tissue shoulder disorders;

• Manipulation/mobilisation (with exercise) for plantar fasciitis;

• Self-mobilising apophyseal glides for cervicogenic headache;

• Self-mobilising apophyseal glides for cervicogenic dizziness; and

• Massage including myofascial release/strain/counterstrain for cancer care.

Both Bronfort et al. [20] and the current review considered the evidence for treating a large range of non-musculoskeletal conditions, but despite finding additional evidence in some cases, the current review was unable to change the inconclusive evidence ratings for these conditions including:

• Asthma using osteopathic manual therapy;

• Paediatric nocturnal enuresis using spinal manipulation;

• Infant colic using cranial osteopathic manual therapy (although new evidence appeared more favourable than that reported in the UK evidence report);

• Premenstrual syndrome using spinal manipulation;

• Stage 1 hypertension using upper cervical (NUCCA) spinal manipulation;

• Stage 1 hypertension using instrumental assisted spinal manipulation;

• Otitis media and pneumonia in elderly adults using osteopathic manual therapy; and

• Pneumonia in elderly adults using osteopathic manual therapy.

### Limitations and strengths

The clinical effectiveness review was limited by the extent of information provided in the included primary studies and clinical/methodological diversity of the included evidence. Most studies had small sample sizes and methodological limitations. For the majority of RCTs it was not clear if the methods for randomization were adequate and the treatment allocation was appropriately concealed. In many cases, either the studies were not blinded or the blinding status of outcome assessors could not be determined. It should be noted that in most situations where physical treatments were applied, blinding was very difficult or impossible to achieve. The lack of description of adequacy of randomisation methods, treatment allocation concealment, and blinding in the studies complicated valid interpretation of the review results. Furthermore, there was a substantial clinical and methodological diversity across the included studies that may have contributed to the observed inconsistencies in the results. For example, there has been a large variation in types of manual therapy and their modes of application across studies, which was compounded by differences in control treatments thereby limiting comparability between the study results. Moreover, the therapy provider’s experience, training, and approaches used varied across the trials and this variation may have additionally impacted on the trial results. The above-mentioned clinical diversity limited the extent of statistical pooling of trial results. Poorly and scarcely reported harms data limited our ability to make meaningful comparisons of rates of adverse events between the treatments.

We attempted to take into account a user perspective by considering qualitative studies, however, we only identified a very limited number of studies reflecting patient views of manual therapy.

One of the main strengths of the clinical effectiveness review is its broad scope in terms of reviewed interventions, populations/conditions, and outcome measures. This review identified, appraised, and summarised a large amount of relevant literature. The review authors employed systematic, comprehensive, and independent strategies to minimise the risk of bias in searching, identifying, selecting, extracting, and appraising the evidence. The broad search strategy, not restricted by the language or year of publication, was applied to multiple electronic and other bibliographic sources.

### Research needs/recommendations

The current research has highlighted the need for long-term large pragmatic head-to-head trials reporting clinically relevant and validated efficacy outcomes. If ethically justifiable, future trials need to include a sham or no treatment arm to allow the assessment and separation of non-specific effects (e.g., patient’s expectation) from treatment effects. Furthermore, future research needs to explore which characteristics of manual therapies (e.g., mode of administration, length of treatments, number of sessions, and choice of spinal region/points) are important in terms of their impact on clinically relevant and patient-centred outcomes. Also, strong efforts are needed to improve quality of reporting of primary studies of manual therapies.

The following key research needs and recommendations were highlighted from the report findings:

• Studies need to be developed that involve qualitative research methods to explore patient attitudes, satisfaction with and the acceptability of manual therapy treatments, this could also take the form of mixed methods studies exploring both effectiveness and patient views;

• Greater consistency is needed across research groups in this area in terms of definition of participants, interventions, comparators and outcomes;

• More research is needed on non-musculoskeletal conditions; and

• High quality, long-term, large, randomised trials reporting effectiveness and cost-effectiveness of manual therapy are needed for more definitive conclusions.

## Conclusion

We consider that it is unlikely that the evidence which is available provides a reliable representation of the likely success of manual therapy as provided in the UK. The magnitude of the benefits and harms of all manual therapy interventions across the many conditions reported on cannot be reliably estimated due to the paucity, poor methodological quality and clinical diversity of included studies.

The differences in the therapy providers’ experience, training, and approaches may have additionally contributed to the inconsistent results. Limited research has been published on many non-musculoskeletal conditions. There were considerable gaps in the evidence, inconsistent reporting on techniques and interventions used (with often a lack of description of techniques), and many studies failed to consider the generalisability of the findings to the range of settings in which manual therapy is practised in the UK.

## Competing interests

This project is an extension of a report funded by The Royal College of Chiropractors, available from: http://www2.warwick.ac.uk/fac/med/research/hscience/pet/reportforcollegeofchiropractors/. The authors declare that they have no competing interests.

## Authors’ contributions

PS and GLH developed the review. CC, AT and PS conducted the review, this included: screening and retrieving papers, assessing against inclusion criteria, appraising the quality of papers and abstracting data from papers for narrative synthesis. RC developed the search strategy and undertook searches. All authors were involved in writing draft and final versions of the review. All authors read and approved the final manuscript.

## Authors’ information

CC, AT, PS, RC and AC all work in Warwick Evidence at the University of Warwick and undertake systematic reviews on the clinical and cost effectiveness of health care interventions for the National Institute for Health Research Health Technology Assessment Programme on behalf of a range of policy makers, including the National Institute for Health and Care Excellence (NICE). AC is a Professor of Public Health Research, Director of Warwick Evidence and Acting Director of the Division of Health Sciences. GLH is a Professor of Social Sciences in Health at the University of Warwick.

## Supplementary Material

Additional file 1 PRISMA 2009 Checklist. Click here for file

Additional file 2 Search strategies. Click here for file

Additional file 3 Data tables for included papers – study characteristics, results and conclusions. Click here for file

Additional file 4 Quality assessments of all included papers. Click here for file
