# Supplementary material for: Clinical effectiveness of manual therapy for the management of musculoskeletal and non-musculoskeletal conditions: systematic review and update of UK evidence report
Source: Chiropr Man Therap. 2014 Mar 28;22:12. doi: 10.1186/2045-709X-22-12 (PMC3997823; doi:10.1186/2045-709X-22-12)
Supplement: Additional file 2 — Search strategies. [file 2045-709X-22-12-S2.docx]

# Additional file 2. Search strategies

**Medline via Ovid searched on 25/08/2011**

| 1 | Musculoskeletal Manipulations/ | 647 |
| --- | --- | --- |
| 2 | Manipulation, Orthopedic/ | 3196 |
| 3 | Manipulation, Chiropractic/ | 599 |
| 4 | Manipulation, Spinal/ | 947 |
| 5 | Manipulation, Osteopathic/ | 275 |
| 6 | Chiropractic/ | 2910 |
| 7 | ((orthopaedic or orthopedic or chiropract$ or chirother$ or osteopath$ or spine or spinal or vertebra$ or craniocervical or craniosacral or "cranio sacral" or cervical or lumbar or occiput or invertebral or thoracic or sacral or sacroilial or joint$) adj3 (manipulat$ or adjustment$ or mobilis$ or mobiliz$ or traction$)).tw. | 3748 |
| 8 | ((manual or manipulat$ or mobilis$ or mobiliz$) adj (therap$ or intervention$ or treat$ or rehab$)).tw. | 2087 |
| 9 | 1 or 2 or 3 or 4 or 5 or 6 or 7 or 8 | 10834 |
| 10 | Osteopathic Medicine/ | 2395 |
| 11 | osteopath$.tw. | 3382 |
| 12 | chiropractic$.tw. | 2684 |
| 13 | chirother$.tw. | 16 |
| 14 | 10 or 11 or 12 or 13 | 6949 |
| 15 | 9 or 14 | 14942 |
| 16 | "friction massage$".tw. | 22 |
| 17 | naprapath$.tw. | 13 |
| 18 | Rolfing.tw. | 17 |
| 19 | "myofascial release".tw. | 53 |
| 20 | "Bowen technique".tw. | 5 |
| 21 | "apophyseal glide$".tw. | 7 |
| 22 | "bone setting".tw. | 47 |
| 23 | bonesetting.tw. | 14 |
| 24 | "body work$".tw. | 103 |
| 25 | "high-velocity low-amplitude".tw. | 94 |
| 26 | HVLA.tw. | 21 |
| 27 | ((Maitland or Kaltenborn or Evejenth or Evjenth or Mulligan or McKenzie or Cyriax or Mills or Mennell or Stoddard) adj3 (manipulat$ or adjustment$ or mobilis$ or mobiliz$ or traction$)).tw. | 17 |
| 28 | 16 or 17 or 18 or 19 or 20 or 21 or 22 or 23 or 24 or 25 or 26 or 27 | 386 |
| 29 | 15 or 28 | 15151 |
| 30 | meta.ab. | 37484 |
| 31 | synthesis.ab. | 356691 |
| 32 | literature.ab. | 333797 |
| 33 | randomized.hw. | 385278 |
| 34 | published.ab. | 229952 |
| 35 | meta-analysis.pt. | 30214 |
| 36 | extraction.ab. | 106463 |
| 37 | trials.hw. | 241415 |
| 38 | controlled.hw. | 476605 |
| 39 | search.ab. | 111279 |
| 40 | medline.ab. | 37563 |
| 41 | selection.ab. | 186391 |
| 42 | sources.ab. | 136598 |
| 43 | trials.ab. | 231023 |
| 44 | review.ab. | 521671 |
| 45 | review.pt. | 1668378 |
| 46 | articles.ab. | 43106 |
| 47 | reviewed.ab. | 273309 |
| 48 | english.ab. | 34846 |
| 49 | language.ab. | 55323 |
| 50 | 30 or 31 or 32 or 33 or 34 or 35 or 36 or 37 or 38 or 39 or 40 or 41 or 42 or 43 or 44 or 45 or 46 or 47 or 48 or 49 | 3593074 |
| 51 | comment.pt. | 449950 |
| 52 | letter.pt. | 723862 |
| 53 | editorial.pt. | 282269 |
| 54 | Animals/ | 4854330 |
| 55 | Humans/ | 12014638 |
| 56 | 54 and 55 | 1282233 |
| 57 | 54 not 56 | 3572097 |
| 58 | 51 or 52 or 53 or 57 | 4613893 |
| 59 | 50 not 58 | 3118764 |
| 60 | 29 and 59 | 3786 |
| 61 | meta-analysis.mp,pt. | 47915 |
| 62 | review.pt. | 1668378 |
| 63 | search$.tw. | 167947 |
| 64 | 61 or 62 or 63 | 1800589 |
| 65 | 29 and 64 | 1754 |
| 66 | 60 or 65 | 3869 |
| 67 | randomized controlled trial.pt. | 314563 |
| 68 | controlled clinical trial.pt. | 83211 |
| 69 | randomized.ab. | 220397 |
| 70 | placebo.ab. | 127540 |
| 71 | drug therapy.fs. | 1488387 |
| 72 | randomly.ab. | 159149 |
| 73 | trial.ab. | 227916 |
| 74 | groups.ab. | 1056224 |
| 75 | 67 or 68 or 69 or 70 or 71 or 72 or 73 or 74 | 2752777 |
| 76 | exp animals/ not humans.sh. | 3654092 |
| 77 | 75 not 76 | 2335094 |
| 78 | 29 and 77 | 2268 |
| 79 | exp Cohort Studies/ | 1124315 |
| 80 | cohort$.tw. | 181429 |
| 81 | controlled clinical trial.pt. | 83211 |
| 82 | Epidemiologic Methods/ | 27602 |
| 83 | limit 82 to yr="1971-1988" | 9410 |
| 84 | 79 or 80 or 81 or 83 | 1268588 |
| 85 | 29 and 84 | 1737 |
| 86 | 66 or 78 or 85 | 5540 |
| 87 | interview$.mp. | 191377 |
| 88 | experience$.mp. | 552122 |
| 89 | qualitative.tw. | 86147 |
| 90 | qualitative research/ | 11344 |
| 91 | 87 or 88 or 89 or 90 | 772947 |
| 92 | 29 and 91 | 1194 |
| 93 | 86 or 92 | 6056 |
| 94 | Economics/ | 26136 |
| 95 | exp "costs and cost analysis"/ | 159102 |
| 96 | economics, dental/ | 1829 |
| 97 | exp "economics, hospital"/ | 17368 |
| 98 | economics, medical/ | 8493 |
| 99 | economics, nursing/ | 3851 |
| 100 | economics, pharmaceutical/ | 2258 |
| 101 | (economic$ or cost or costs or costly or costing or price or prices or pricing or pharmacoeconomic$).ti,ab. | 343421 |
| 102 | (expenditure$ not energy).ti,ab. | 14521 |
| 103 | value for money.ti,ab. | 654 |
| 104 | budget$.ti,ab. | 14687 |
| 105 | 94 or 95 or 96 or 97 or 98 or 99 or 100 or 101 or 102 or 103 or 104 | 457195 |
| 106 | ((energy or oxygen) adj cost).ti,ab. | 2340 |
| 107 | (metabolic adj cost).ti,ab. | 607 |
| 108 | ((energy or oxygen) adj expenditure).ti,ab. | 13432 |
| 109 | 106 or 107 or 108 | 15754 |
| 110 | 105 not 109 | 453621 |
| 111 | letter.pt. | 723862 |
| 112 | editorial.pt. | 282269 |
| 113 | historical article.pt. | 278980 |
| 114 | 111 or 112 or 113 | 1272089 |
| 115 | 110 not 114 | 428994 |
| 116 | Animals/ | 4854330 |
| 117 | Humans/ | 12014638 |
| 118 | 116 not (116 and 117) | 3572097 |
| 119 | 115 not 118 | 404419 |
| 120 | 29 and 119 | 562 |
| 121 | 93 or 120 | **6232** |

**Embase via Ovid searched on 25/08/2011**

| 1 | manipulative medicine/ | 7272 |
| --- | --- | --- |
| 2 | bodywork/ | 45 |
| 3 | chiropractic/ | 2951 |
| 4 | craniosacral therapy/ | 53 |
| 5 | orthopedic manipulation/ | 1881 |
| 6 | osteopathic medicine/ | 2414 |
| 7 | ((orthopaedic or orthopedic or chiropract$ or chirother$ or osteopath$ or spine or spinal or vertebra$ or craniocervical or craniosacral or "cranio sacral" or cervical or lumbar or occiput or invertebral or thoracic or sacral or sacroilial or joint$) adj3 (manipulat$ or adjustment$ or mobilis$ or mobiliz$ or traction$)).tw. | 4560 |
| 8 | ((manual or manipulat$ or mobilis$ or mobiliz$) adj (therap$ or intervention$ or treat$ or rehab$)).tw. | 2891 |
| 9 | osteopath$.tw. | 4117 |
| 10 | chiropractic$.tw. | 3238 |
| 11 | chirother$.tw. | 40 |
| 12 | "friction massage$".tw. | 41 |
| 13 | naprapath$.tw. | 18 |
| 14 | Rolfing.tw. | 27 |
| 15 | "myofascial release".tw. | 84 |
| 16 | "Bowen technique".tw. | 6 |
| 17 | "apophyseal glide$".tw. | 9 |
| 18 | "bone setting".tw. | 60 |
| 19 | bonesetting.tw. | 14 |
| 20 | "body work$".tw. | 141 |
| 21 | "high-velocity low-amplitude".tw. | 121 |
| 22 | HVLA.tw. | 32 |
| 23 | ((Maitland or Kaltenborn or Evejenth or Evjenth or Mulligan or McKenzie or Cyriax or Mills or Mennell or Stoddard) adj3 (manipulat$ or adjustment$ or mobilis$ or mobiliz$ or traction$)).tw. | 39 |
| 24 | 1 or 2 or 3 or 4 or 5 or 6 or 7 or 8 or 9 or 10 or 11 or 12 or 13 or 14 or 15 or 16 or 17 or 18 or 19 or 20 or 21 or 22 or 23 | 19709 |
| 25 | meta-analys$.mp. | 74979 |
| 26 | search$.tw. | 203498 |
| 27 | review.pt. | 1696600 |
| 28 | 25 or 26 or 27 | 1878448 |
| 29 | 24 and 28 | 2776 |
| 30 | random:.tw. | 641600 |
| 31 | placebo:.mp. | 253896 |
| 32 | double-blind:.tw. | 116791 |
| 33 | 30 or 31 or 32 | 808498 |
| 34 | 24 and 33 | 2033 |
| 35 | exp cohort analysis/ | 98966 |
| 36 | exp longitudinal study/ | 44517 |
| 37 | exp prospective study/ | 168438 |
| 38 | exp follow up/ | 537174 |
| 39 | cohort$.tw. | 227949 |
| 40 | 35 or 36 or 37 or 38 or 39 | 887958 |
| 41 | 24 and 40 | 1364 |
| 42 | interview$.tw. | 194152 |
| 43 | qualitative.tw. | 101743 |
| 44 | exp health care organization/ | 870597 |
| 45 | 42 or 43 or 44 | 1110257 |
| 46 | 24 and 45 | 2741 |
| 47 | health-economics/ | 30325 |
| 48 | exp economic-evaluation/ | 169204 |
| 49 | exp health-care-cost/ | 163072 |
| 50 | exp pharmacoeconomics/ | 137702 |
| 51 | 47 or 48 or 49 or 50 | 388203 |
| 52 | (econom$ or cost or costs or costly or costing or price or prices or pricing or pharmacoeconomic$).ti,ab. | 436803 |
| 53 | (expenditure$ not energy).ti,ab. | 17340 |
| 54 | (value adj2 money).ti,ab. | 934 |
| 55 | budget$.ti,ab. | 18435 |
| 56 | 52 or 53 or 54 or 55 | 455470 |
| 57 | 51 or 56 | 685131 |
| 58 | letter.pt. | 735696 |
| 59 | editorial.pt. | 376448 |
| 60 | note.pt. | 442547 |
| 61 | 58 or 59 or 60 | 1554691 |
| 62 | 57 not 61 | 613975 |
| 63 | (metabolic adj cost).ti,ab. | 657 |
| 64 | ((energy or oxygen) adj cost).ti,ab. | 2542 |
| 65 | ((energy or oxygen) adj expenditure).ti,ab. | 15191 |
| 66 | 63 or 64 or 65 | 17722 |
| 67 | 62 not 66 | 609976 |
| 68 | exp animal/ | 1623481 |
| 69 | exp animal-experiment/ | 1457412 |
| 70 | nonhuman/ | 3690694 |
| 71 | (rat or rats or mouse or mice or hamster or hamsters or animal or animals or dog or dogs or cat or cats or bovine or sheep).ti,ab,sh. | 4063433 |
| 72 | 68 or 69 or 70 or 71 | 5880755 |
| 73 | exp human/ | 12433930 |
| 74 | exp human-experiment/ | 292054 |
| 75 | 73 or 74 | 12435312 |
| 76 | 72 not (72 and 75) | 4640149 |
| 77 | 67 not 76 | 566499 |
| 78 | 24 and 77 | 1020 |
| 79 | 29 or 34 or 41 or 46 or 78 | **7546** |

**AMED via Ovid searched on 30/08/2011**

**N.b. no search filters are available for AMED. Therefore, due to high numbers retrieved from the subject search, I have translated the Medline filters used.**

| 1 | manipulation/ | 624 |
| --- | --- | --- |
| 2 | musculoskeletal manipulations/ | 86 |
| 3 | exp manipulation chiropractic/ | 851 |
| 4 | exp manipulation osteopathic/ | 213 |
| 5 | spinal manipulation/ | 706 |
| 6 | peripheral manipulation/ | 74 |
| 7 | chiropractic/ | 5953 |
| 8 | osteopathy/ | 1312 |
| 9 | mobilisation/ | 283 |
| 10 | peripheral mobilisation/ | 125 |
| 11 | spinal mobilisation/ | 124 |
| 12 | ((orthopaedic or orthopedic or chiropract$ or chirother$ or osteopath$ or spine or spinal or vertebra$ or craniocervical or craniosacral or "cranio sacral" or cervical or lumbar or occiput or invertebral or thoracic or sacral or sacroilial or joint$) adj3 (manipulat$ or adjustment$ or mobilis$ or mobiliz$ or traction$)).tw. | 2659 |
| 13 | ((manual or manipulat$ or mobilis$ or mobiliz$) adj (therap$ or intervention$ or treat$ or rehab$)).tw. | 1397 |
| 14 | osteopath$.tw. | 1804 |
| 15 | chiropractic$.tw. | 7038 |
| 16 | chirother$.tw. | 32 |
| 17 | "friction massage$".tw. | 28 |
| 18 | naprapath$.tw. | 8 |
| 19 | Rolfing.tw. | 25 |
| 20 | "myofascial release".tw. | 51 |
| 21 | "Bowen technique".tw. | 7 |
| 22 | "apophyseal glide$".tw. | 6 |
| 23 | "bone setting".tw. | 6 |
| 24 | bonesetting.tw. | 3 |
| 25 | "body work$".tw. | 38 |
| 26 | "high-velocity low-amplitude".tw. | 95 |
| 27 | HVLA.tw. | 23 |
| 28 | ((Maitland or Kaltenborn or Evejenth or Evjenth or Mulligan or McKenzie or Cyriax or Mills or Mennell or Stoddard) adj3 (manipulat$ or adjustment$ or mobilis$ or mobiliz$ or traction$)).tw. | 20 |
| 29 | 1 or 2 or 3 or 4 or 5 or 6 or 7 or 8 or 9 or 10 or 11 or 12 or 13 or 14 or 15 or 16 or 17 or 18 or 19 or 20 or 21 or 22 or 23 or 24 or 25 or 26 or 27 or 28 | 11202 |
| 30 | meta-analysis.mp,pt. | 631 |
| 31 | review.mp,pt. | 14913 |
| 32 | search$.tw. | 3188 |
| 33 | 30 or 31 or 32 | 16591 |
| 34 | 29 and 33 | 975 |
| 35 | randomized controlled trial.pt. | 1997 |
| 36 | controlled clinical trial.pt. | 70 |
| 37 | randomized.ab. | 5335 |
| 38 | placebo.ab. | 1981 |
| 39 | clinical trials/ or randomized controlled trials/ or double blind method/ or random allocation/ | 3365 |
| 40 | randomly.ab. | 3839 |
| 41 | trial.ab. | 5572 |
| 42 | groups.ab. | 16288 |
| 43 | 35 or 36 or 37 or 38 or 39 or 40 or 41 or 42 | 25693 |
| 44 | exp animals/ not humans.sh. | 5883 |
| 45 | 43 not 44 | 24839 |
| 46 | 29 and 45 | 1003 |
| 47 | cohort studies/ | 259 |
| 48 | follow up studies/ | 896 |
| 49 | longitudinal studies/ | 110 |
| 50 | prospective studies/ | 370 |
| 51 | cohort$.tw. | 3023 |
| 52 | 47 or 48 or 49 or 50 or 51 | 4143 |
| 53 | 29 and 52 | 132 |
| 54 | interview$.mp. | 7711 |
| 55 | experience$.mp. | 14946 |
| 56 | qualitative.tw. | 3977 |
| 57 | 54 or 55 or 56 | 21625 |
| 58 | 29 and 57 | 741 |
| 59 | Economics/ | 2048 |
| 60 | exp "costs and cost analysis"/ | 1023 |
| 61 | (economic$ or cost or costs or costly or costing or price or prices or pricing or pharmacoeconomic$).ti,ab. | 5729 |
| 62 | (expenditure$ not energy).ti,ab. | 225 |
| 63 | value for money.ti,ab. | 18 |
| 64 | budget$.ti,ab. | 166 |
| 65 | 59 or 60 or 61 or 62 or 63 or 64 | 7429 |
| 66 | ((energy or oxygen) adj cost).ti,ab. | 285 |
| 67 | (metabolic adj cost).ti,ab. | 66 |
| 68 | ((energy or oxygen) adj expenditure).ti,ab. | 441 |
| 69 | 66 or 67 or 68 | 724 |
| 70 | 65 not 69 | 7063 |
| 71 | letter.pt. | 4564 |
| 72 | editorial.pt. | 5336 |
| 73 | 71 or 72 | 9899 |
| 74 | 70 not 73 | 6897 |
| 75 | exp Animals/ | 65654 |
| 76 | Humans/ | 59771 |
| 77 | 75 not (75 and 76) | 5883 |
| 78 | 74 not 77 | 6869 |
| 79 | 29 and 78 | 536 |
| 80 | 34 or 46 or 53 or 58 or 79 | **2749** |

**Cochrane Airways Group trial register, Cochrane Complementary Medicine Field register and Cochrane Rehabilitation Field register via the Cochrane Library (CENTRAL) searched on 30/08/2011**

#1 MeSH descriptor Musculoskeletal Manipulations, this term only

#2 MeSH descriptor Manipulation, Orthopedic, this term only

#3 MeSH descriptor Manipulation, Chiropractic, this term only

#4 MeSH descriptor Manipulation, Spinal, this term only

#5 MeSH descriptor Manipulation, Osteopathic, this term only

#6 MeSH descriptor Chiropractic, this term only

#7 ((orthopaedic or orthopedic or chiropract* or chirother* or osteopath* or spine or spinal or vertebra* or craniocervical or craniosacral or "cranio sacral" or cervical or lumbar or occiput or invertebral or thoracic or sacral or sacroilial or joint*) NEAR/3 (manipulat* or adjustment* or mobilis* or mobiliz* or traction*)):ti,kw,ab

#8 ((manual or manipulat* or mobilis* or mobiliz*) NEXT (therap* or intervention* or treat* or rehab*)):ti,kw,ab

#9 (#1 OR #2 OR #3 OR #4 OR #5 OR #6 OR #7 OR #8)

#10 MeSH descriptor Osteopathic Medicine, this term only

#11 osteopath*:ti,kw,ab

#12 chiropractic*:ti,kw,ab

#13 chirother*:ti,kw,ab

#14 (#10 OR #11 OR #12 OR #13)

#15 (#9 OR #14)

#16 ("friction massage" OR "friction massages" OR naprapath* OR Rolfing OR "myofascial release" OR "Bowen technique" OR "apophyseal glide" OR "apophyseal glides" OR "bone setting" OR bonesetting OR "body work" OR "body works" OR "high-velocity low-amplitude" OR HVLA):ti,kw,ab

#17 ((Maitland or Kaltenborn or Evejenth or Evjenth or Mulligan or McKenzie or Cyriax or Mills or Mennell or Stoddard) NEAR/3 (manipulat* or adjustment* or mobilis* or mobiliz* or traction*)):ti,kw,ab

#18 (#16 OR #17)

#19 (#15 OR #18) 1608

#20 (SR-AIRWAYS) in Clinical Trials 26755

#21 (SR-COMPMED) in Clinical Trials 39144

#22 (SR-REHAB) in Clinical Trials 5377

#23 (#19 AND #20) **22**

#24 (#19 AND #21) **810**

#25 (#19 AND #22) **298**

**Cochrane Database of Systematic Reviews (CDSR) and CENTRAL via the Cochrane Library searched on 30/08/2011**

#1 MeSH descriptor Musculoskeletal Manipulations, this term only

#2 MeSH descriptor Manipulation, Orthopedic, this term only

#3 MeSH descriptor Manipulation, Chiropractic, this term only

#4 MeSH descriptor Manipulation, Spinal, this term only

#5 MeSH descriptor Manipulation, Osteopathic, this term only

#6 MeSH descriptor Chiropractic, this term only

#7 ((orthopaedic or orthopedic or chiropract* or chirother* or osteopath* or spine or spinal or vertebra* or craniocervical or craniosacral or "cranio sacral" or cervical or lumbar or occiput or invertebral or thoracic or sacral or sacroilial or joint*) NEAR/3 (manipulat* or adjustment* or mobilis* or mobiliz* or traction*)):ti,kw,ab

#8 ((manual or manipulat* or mobilis* or mobiliz*) NEXT (therap* or intervention* or treat* or rehab*)):ti,kw,ab

#9 (#1 OR #2 OR #3 OR #4 OR #5 OR #6 OR #7 OR #8)

#10 MeSH descriptor Osteopathic Medicine, this term only

#11 osteopath*:ti,kw,ab

#12 chiropractic*:ti,kw,ab

#13 chirother*:ti,kw,ab

#14 (#10 OR #11 OR #12 OR #13)

#15 (#9 OR #14)

#16 ("friction massage" OR "friction massages" OR naprapath* OR Rolfing OR "myofascial release" OR "Bowen technique" OR "apophyseal glide" OR "apophyseal glides" OR "bone setting" OR bonesetting OR "body work" OR "body works" OR "high-velocity low-amplitude" OR HVLA):ti,kw,ab

#17 ((Maitland or Kaltenborn or Evejenth or Evjenth or Mulligan or McKenzie or Cyriax or Mills or Mennell or Stoddard) NEAR/3 (manipulat* or adjustment* or mobilis* or mobiliz* or traction*)):ti,kw,ab

#18 (#16 OR #17)

#19 (#15 OR #18)

CDSR **36** (33 reviews, 3 protocols)

DARE 96

CENTRAL **1405**

Methodology database 34

HTA 17

NHS EED 20

Cochrane Groups 0

TOTAL **1608**

**CINAHL via EBSCO searched on 02/09/2011**

n.b. search reads from bottom to top

| **#** | **Query** | **Results** |
| --- | --- | --- |
| S108 | S62 or S75 or S79 or S84 or S107 | **3263** |
| S107 | S46 and S106 | 877 |
| S106 | S102 NOT S105 | 91959 |
| S105 | S103 NOT (S103 AND S104) | 19393 |
| S104 | MH Human | 643247 |
| S103 | MH Animals | 20680 |
| S102 | S97 NOT S101 | 92156 |
| S101 | S98 or S99 or S100 | 284664 |
| S100 | PT commentary | 123175 |
| S99 | PT letter | 107805 |
| S98 | PT editorial | 125645 |
| S97 | S95 or S96 | 100024 |
| S96 | TI (cost or costs or economic* or pharmacoeconomic* or price* or pricing*) OR AB (cost or costs or economic* or pharmacoeconomic* or price* or pricing*) | 68711 |
| S95 | S91 or S94 | 46377 |
| S94 | S92 or S93 | 11637 |
| S93 | MH Health Resource Utilization | 7062 |
| S92 | MH Health Resource Allocation | 4823 |
| S91 | S85 NOT S90 | 38689 |
| S90 | S86 or S87 or S88 or S89 | 354375 |
| S89 | MH Business+ | 53776 |
| S88 | MH Financing, Organized+ | 71712 |
| S87 | MH Financial Support+ | 226604 |
| S86 | MH Financial Management+ | 28046 |
| S85 | MH Economics+ | 359283 |
| S84 | S46 and S83 | 279 |
| S83 | S80 or S81 or S82 | 69741 |
| S82 | TX qualitative stud* | 40219 |
| S81 | MH Audiorecording | 21422 |
| S80 | TI interview OR AB interview | 23731 |
| S79 | S46 and S78 | 984 |
| S78 | S76 or S77 | 135735 |
| S77 | TI cohort* OR AB cohort* | 35468 |
| S76 | (MH "Prospective Studies+") | 120544 |
| S75 | S46 and S74 | 1557 |
| S74 | S63 or S64 or S65 or S66 or S67 or S68 or S69 or S70 or S71 or S72 or S73 | 589111 |
| S73 | TX allocat* random* | 248 |
| S72 | MH Quantitative Studies | 6760 |
| S71 | MH Placebos | 6004 |
| S70 | TX placebo* | 21404 |
| S69 | TX random* allocat* | 2543 |
| S68 | MH Random Assignment | 26198 |
| S67 | TX randomi* control* trial* | 30470 |
| S66 | TX ((singl* N1 blind*) or (singl* N1 mask*)) or TX ((doubl* N1 blind*) or (doubl* N1 mask*)) or TX ((tripl* N1 blind*) or (tripl* N1 mask*)) or TX ((trebl* N1 blind*) or (trebl* N1 mask*)) | 491906 |
| S65 | TX clinic* N1 trial* | 102677 |
| S64 | PT Clinical trial | 48879 |
| S63 | MH Clinical Trials+ | 97579 |
| S62 | S46 and S61 | 700 |
| S61 | S52 NOT S60 | 33079 |
| S60 | S56 OR S59 | 301587 |
| S59 | S57 NOT (S57 AND S58) | 19393 |
| S58 | MH Human | 643247 |
| S57 | MH Animals | 20680 |
| S56 | S53 or S54 or S55 | 284664 |
| S55 | PT Editorial | 125645 |
| S54 | PT Letter | 107805 |
| S53 | PT Commentary | 123175 |
| S52 | S47 or S48 or S49 or S50 or S51 | 38152 |
| S51 | TX systematic review OR TX systematic overview | 28467 |
| S50 | MH Literature Review+ | 11509 |
| S49 | TX metaanalys* | 329 |
| S48 | TX meta analys* | 15229 |
| S47 | MH Meta Analysis | 10675 |
| S46 | S22 or S28 or S45 | 33846 |
| S45 | S29 or S30 or S31 or S32 or S33 or S34 or S35 or S36 or S37 or S38 or S39 or S44 | 1297 |
| S44 | S40 or S41 or S42 or S43 | 39 |
| S43 | TX (Maitland N3 traction*) or TX (Kaltenborn N3 traction*) or TX (Evejenth N3 traction*) or TX (Evjenth N3 traction*) or TX (Mulligan N3 traction*) or TX (McKenzie N3 traction*) or TX (Cyriax N3 traction*) or TX (Mills N3 traction*) or TX (Mennell N3 traction*) or TX (Stoddard N3 traction*) | 5 |
| S42 | TX (Maitland N3 mobili?*) or TX (Kaltenborn N3 mobili?*) or TX (Evejenth N3 mobili?*) or TX (Evjenth N3 mobili?*) or TX (Mulligan N3 mobili?*) or TX (McKenzie N3 mobili?*) or TX (Cyriax N3 mobili?*) or TX (Mills N3 mobili?*) or TX (Mennell N3 mobili?*) or TX (Stoddard N3 mobili?*) | 27 |
| S41 | TX (Maitland N3 adjustment*) or TX (Kaltenborn N3 adjustment*) or TX (Evejenth N3 adjustment*) or TX (Evjenth N3 adjustment*) or TX (Mulligan N3 adjustment*) or TX (McKenzie N3 adjustment*) or TX (Cyriax N3 adjustment*) or TX (Mills N3 adjustment*) or TX (Mennell N3 adjustment*) or TX (Stoddard N3 adjustment*) | 0 |
| S40 | TX (Maitland N3 manipulat*) or TX (Kaltenborn N3 manipulat*) or TX (Evejenth N3 manipulat*) or TX (Evjenth N3 manipulat*) or TX (Mulligan N3 manipulat*) or TX (McKenzie N3 manipulat*) or TX (Cyriax N3 manipulat*) or TX (Mills N3 manipulat*) or TX (Mennell N3 manipulat*) or TX (Stoddard N3 manipulat*) | 11 |
| S39 | TX HVLA | 41 |
| S38 | TX high-velocity low-amplitude | 140 |
| S37 | TX body work* | 685 |
| S36 | TX bonesetting | 4 |
| S35 | TX bone setting | 54 |
| S34 | TX apophyseal glide* | 9 |
| S33 | TX Bowen technique | 37 |
| S32 | TX myofascial release | 215 |
| S31 | TX Rolfing | 72 |
| S30 | TX Naprapath* | 7 |
| S29 | TX friction massage* | 45 |
| S28 | S23 or S24 or S25 or S26 or S27 | 27527 |
| S27 | TX chirother* | 2 |
| S26 | TX chiropractic* | 24748 |
| S25 | TX osteopath* | 3416 |
| S24 | MH Osteopathy | 1205 |
| S23 | MH Osteopathic Medicine | 77 |
| S22 | S1 or S2 or S3 or S4 or S5 or S6 or S7 or S8 or S9 or S10 or S11 or S16 or S21 | 18486 |
| S21 | S17 or S18 or S19 or S20 | 6188 |
| S20 | TX (manual N1 rehab*) or TX (manipulat* N1 rehab*) or TX (mobile?* N1 rehab*) | 40 |
| S19 | TX (manual N1 treat*) or TX (manipulat* N1 treat*) or TX (mobile?* N1 treat*) | 557 |
| S18 | TX (manual N1 intervention*) or TX (manipulat* N1 intervention*) or TX (mobile?* N1 intervention*) | 179 |
| S17 | TX (manual N1 therap*) or TX (manipulat* N1 therap*) or TX (mobile?* N1 therap*) | 5741 |
| S16 | S12 or S13 or S14 or S15 | 6268 |
| S15 | TX (orthop#edic N3 traction*) or TX (chiropract* N3 traction*) or TX (chirother* N3 traction*) or TX (osteopath* N3 traction*) or TX (spine N3 traction*) or TX (spinal N3 traction*) or TX (vertebra* N3 traction*) or TX (craniocervical N3 traction*) or TX (craniosacral N3 traction*) or TX (cervical N3 traction*) or TX (lumbar N3 traction*) or TX (occiput N3 traction*) or TX (invertebral N3 traction*) or TX (thoracic N3 traction*) or TX (sacral N3 traction*) or TX (sacroilial N3 traction*) or TX (joint* N3 traction*) | 242 |
| S14 | TX (orthop#edic N3 mobili?*) or TX (chiropract* N3 mobili?*) or TX (chirother* N3 mobili?*) or TX (osteopath* N3 mobili?*) or TX (spine N3 mobili?*) or TX (spinal N3 mobili?*) or TX (vertebra* N3 mobili?*) or TX (craniocervical N3 mobili?*) or TX (craniosacral N3 mobili?*) or TX (cervical N3 mobili?*) or TX (lumbar N3 mobili?*) or TX (occiput N3 mobili?*) or TX (invertebral N3 mobili?*) or TX (thoracic N3 mobili?*) or TX (sacral N3 mobili?*) or TX (sacroilial N3 mobili?*) or TX (joint* N3 mobili?*) | 1367 |
| S13 | TX (orthop#edic N3 adjustment*) or TX (chiropract* N3 adjustment*) or TX (chirother* N3 adjustment*) or TX (osteopath* N3 adjustment*) or TX (spine N3 adjustment*) or TX (spinal N3 adjustment*) or TX (vertebra* N3 adjustment*) or TX (craniocervical N3 adjustment*) or TX (craniosacral N3 adjustment*) or TX (cervical N3 adjustment*) or TX (lumbar N3 adjustment*) or TX (occiput N3 adjustment*) or TX (invertebral N3 adjustment*) or TX (thoracic N3 adjustment*) or TX (sacral N3 adjustment*) or TX (sacroilial N3 adjustment*) or TX (joint* N3 adjustment*) | 430 |
| S12 | TX (orthop#edic N3 manipulat*) or TX (chiropract* N3 manipulat*) or TX (chirother* N3 manipulat*) or TX (osteopath* N3 manipulat*) or TX (spine N3 manipulat*) or TX (spinal N3 manipulat*) or TX (vertebra* N3 manipulat*) or TX (craniocervical N3 manipulat*) or TX (craniosacral N3 manipulat*) or TX (cervical N3 manipulat*) or TX (lumbar N3 manipulat*) or TX (occiput N3 manipulat*) or TX (invertebral N3 manipulat*) or TX (thoracic N3 manipulat*) or TX (sacral N3 manipulat*) or TX (sacroilial N3 manipulat*) or TX (joint* N3 manipulat*) | 4826 |
| S11 | MH Trager Method | 20 |
| S10 | MH Rolfing | 58 |
| S9 | MH Hellerwork | 5 |
| S8 | MH Structural-Functional-Movement Integration | 33 |
| S7 | MH Craniosacral Therapy | 220 |
| S6 | MH Chiropractic | 8641 |
| S5 | MH Manipulation, Osteopathic | 235 |
| S4 | MH Myofascial Release | 159 |
| S3 | MH Manipulation, Chiropractic | 2718 |
| S2 | MH Manipulation, Orthopedic | 1283 |
| S1 | MH Manual Therapy | 1906 |

CINAHL Totals

Subject search = 33846

Subject search plus SIGN SR filter = 700

Subject search plus SIGN RCT filter = 1557

Subject search plus Cohort filter = 984

Subject search plus Qualitative filter = 279

Subject search plus Economic filter = 877

Subject search AND (all filters combined with OR) = 3263

**SCI and SSCI via Web of Science searched on 06/09/2011**

| # 41 | [2,585](http://apps.webofknowledge.com/summary.do?product=WOS&doc=1&qid=187&SID=P2jap2m5E6gCG3I2fCM&search_mode=CombineSearches) | #40 AND #25  *Databases=SCI-EXPANDED, SSCI Timespan=All Years*  *Lemmatization=On* |
| --- | --- | --- |
| # 40 | [3,199,001](http://apps.webofknowledge.com/summary.do?product=WOS&doc=1&qid=186&SID=P2jap2m5E6gCG3I2fCM&search_mode=CombineSearches) | #39 OR #29 OR #28 OR #27 OR #26  *Databases=SCI-EXPANDED, SSCI Timespan=All Years*  *Lemmatization=On* |
| # 39 | [1,005,197](http://apps.webofknowledge.com/summary.do?product=WOS&doc=1&qid=184&SID=P2jap2m5E6gCG3I2fCM&search_mode=AdvancedSearch) | #34 NOT #38  *Databases=SCI-EXPANDED, SSCI Timespan=All Years*  *Lemmatization=On* |
| # 38 | [29,406](http://apps.webofknowledge.com/summary.do?product=WOS&doc=1&qid=183&SID=P2jap2m5E6gCG3I2fCM&search_mode=CombineSearches) | #37 OR #36 OR #35  *Databases=SCI-EXPANDED, SSCI Timespan=All Years*  *Lemmatization=On* |
| # 37 | [20,390](http://apps.webofknowledge.com/summary.do?product=WOS&doc=1&qid=182&SID=P2jap2m5E6gCG3I2fCM&search_mode=AdvancedSearch) | TS=((energy or oxygen) NEAR/1 expenditure)  *Databases=SCI-EXPANDED, SSCI Timespan=All Years*  *Lemmatization=On* |
| # 36 | [1,520](http://apps.webofknowledge.com/summary.do?product=WOS&doc=1&qid=181&SID=P2jap2m5E6gCG3I2fCM&search_mode=AdvancedSearch) | TS=(metabolic NEAR/1 cost)  *Databases=SCI-EXPANDED, SSCI Timespan=All Years*  *Lemmatization=On* |
| # 35 | [8,646](http://apps.webofknowledge.com/summary.do?product=WOS&doc=1&qid=180&SID=P2jap2m5E6gCG3I2fCM&search_mode=AdvancedSearch) | TS=((energy or oxygen) NEAR/1 cost)  *Databases=SCI-EXPANDED, SSCI Timespan=All Years*  *Lemmatization=On* |
| # 34 | [1,016,684](http://apps.webofknowledge.com/summary.do?product=WOS&doc=1&qid=179&SID=P2jap2m5E6gCG3I2fCM&search_mode=CombineSearches) | #33 OR #32 OR #31 OR #30  *Databases=SCI-EXPANDED, SSCI Timespan=All Years*  *Lemmatization=On* |
| # 33 | [57,314](http://apps.webofknowledge.com/summary.do?product=WOS&doc=1&qid=178&SID=P2jap2m5E6gCG3I2fCM&search_mode=AdvancedSearch) | TS=budget*  *Databases=SCI-EXPANDED, SSCI Timespan=All Years*  *Lemmatization=On* |
| # 32 | [929](http://apps.webofknowledge.com/summary.do?product=WOS&doc=1&qid=177&SID=P2jap2m5E6gCG3I2fCM&search_mode=AdvancedSearch) | TS="value for money"  *Databases=SCI-EXPANDED, SSCI Timespan=All Years*  *Lemmatization=On* |
| # 31 | [26,364](http://apps.webofknowledge.com/summary.do?product=WOS&doc=1&qid=176&SID=P2jap2m5E6gCG3I2fCM&search_mode=AdvancedSearch) | TS=(expenditure* not energy)  *Databases=SCI-EXPANDED, SSCI Timespan=All Years*  *Lemmatization=On* |
| # 30 | [957,320](http://apps.webofknowledge.com/summary.do?product=WOS&doc=1&qid=175&SID=P2jap2m5E6gCG3I2fCM&search_mode=AdvancedSearch) | TS=(economic* or cost or costs or costly or costing or price or prices or pricing or pharmacoeconomic*)  *Databases=SCI-EXPANDED, SSCI Timespan=All Years*  *Lemmatization=On* |
| # 29 | [940,671](http://apps.webofknowledge.com/summary.do?product=WOS&doc=1&qid=174&SID=P2jap2m5E6gCG3I2fCM&search_mode=AdvancedSearch) | TS=(interview* or experience* or qualitative)  *Databases=SCI-EXPANDED, SSCI Timespan=All Years*  *Lemmatization=On* |
| # 28 | [208,875](http://apps.webofknowledge.com/summary.do?product=WOS&doc=1&qid=173&SID=P2jap2m5E6gCG3I2fCM&search_mode=AdvancedSearch) | TS=cohort*  *Databases=SCI-EXPANDED, SSCI Timespan=All Years*  *Lemmatization=On* |
| # 27 | [1,023,892](http://apps.webofknowledge.com/summary.do?product=WOS&doc=1&qid=172&SID=P2jap2m5E6gCG3I2fCM&search_mode=AdvancedSearch) | TS=(random* or placebo* or double-blind*) or TS=(double SAME blind*) or TI=trial*  *Databases=SCI-EXPANDED, SSCI Timespan=All Years*  *Lemmatization=On* |
| # 26 | [356,113](http://apps.webofknowledge.com/summary.do?product=WOS&doc=1&qid=171&SID=P2jap2m5E6gCG3I2fCM&search_mode=AdvancedSearch) | TS=("meta analysis" or meta-analys* or "systematic review" or "systematic reviews" or search*)  *Databases=SCI-EXPANDED, SSCI Timespan=All Years*  *Lemmatization=On* |
| # 25 | [8,668](http://apps.webofknowledge.com/summary.do?product=WOS&doc=1&qid=170&SID=P2jap2m5E6gCG3I2fCM&search_mode=CombineSearches) | #24 OR #23 OR #17 OR #4  *Databases=SCI-EXPANDED, SSCI Timespan=All Years*  *Lemmatization=On* |
| # 24 | [3,599](http://apps.webofknowledge.com/summary.do?product=WOS&doc=1&qid=169&SID=P2jap2m5E6gCG3I2fCM&search_mode=AdvancedSearch) | TS=((orthopaedic or orthopedic or chiropract* or chirother* or osteopath* or spine or spinal or vertebra* or craniocervical or craniosacral or "cranio sacral" or cervical or lumbar or occiput or invertebral or thoracic or sacral or sacroilial or joint*) NEAR/1 (manipulat* or adjustment* or mobilis* or mobiliz* or traction*))  *Databases=SCI-EXPANDED, SSCI Timespan=All Years*  *Lemmatization=On* |
| # 23 | [2,098](http://apps.webofknowledge.com/summary.do?product=WOS&doc=1&qid=168&SID=P2jap2m5E6gCG3I2fCM&search_mode=CombineSearches) | #22 OR #21 OR #20 OR #19 OR #18  *Databases=SCI-EXPANDED, SSCI Timespan=All Years*  *Lemmatization=On* |
| # 22 | [1,300](http://apps.webofknowledge.com/summary.do?product=WOS&doc=1&qid=167&SID=P2jap2m5E6gCG3I2fCM&search_mode=AdvancedSearch) | TS=("manual therapy" or "manual therapies" or "manual therapeutics" or "manual therapist" or "manual therapists" or "manual intervention" or "manual interventions" or "manual treatment" or "manual treatments" or "manual rehabilitation")  *Databases=SCI-EXPANDED, SSCI Timespan=All Years*  *Lemmatization=On* |
| # 21 | [603](http://apps.webofknowledge.com/summary.do?product=WOS&doc=1&qid=166&SID=P2jap2m5E6gCG3I2fCM&search_mode=AdvancedSearch) | TS=("manipulative therapy" or "manipulative therapies" or "manipulative therapeutics" or "manipulative therapist" or "manipulative therapists" or "manipulative intervention" or "manipulative interventions" or "manipulative treatment" or "manipulative treatments" or "manipulative rehabilitation")  *Databases=SCI-EXPANDED, SSCI Timespan=All Years*  *Lemmatization=On* |
| # 20 | [186](http://apps.webofknowledge.com/summary.do?product=WOS&doc=1&qid=165&SID=P2jap2m5E6gCG3I2fCM&search_mode=AdvancedSearch) | TS=("manipulation therapy" or "manipulation therapies" or "manipulation therapeutics" or "manipulation therapist" or "manipulation therapists" or "manipulation intervention" or "manipulation interventions" or "manipulation treatment" or "manipulation treatments" or "manipulation rehabilitation")  *Databases=SCI-EXPANDED, SSCI Timespan=All Years*  *Lemmatization=On* |
| # 19 | [13](http://apps.webofknowledge.com/summary.do?product=WOS&doc=1&qid=164&SID=P2jap2m5E6gCG3I2fCM&search_mode=AdvancedSearch) | TS=("mobilisation therapy" or "mobilisation therapies" or "mobilisation therapeutics" or "mobilisation therapist" or "mobilisation therapists" or "mobilisation intervention" or "mobilisation interventions" or "mobilisation treatment" or "mobilisation treatments" or "mobilisation rehabilitation")  *Databases=SCI-EXPANDED, SSCI Timespan=All Years*  *Lemmatization=On* |
| # 18 | [113](http://apps.webofknowledge.com/summary.do?product=WOS&doc=1&qid=163&SID=P2jap2m5E6gCG3I2fCM&search_mode=AdvancedSearch) | TS=("mobilization therapy" or "mobilization therapies" or "mobilization therapeutics" or "mobilization therapist" or "mobilization therapists" or "mobilization intervention" or "mobilization interventions" or "mobilization treatment" or "mobilization treatments" or "mobilization rehabilitation")  *Databases=SCI-EXPANDED, SSCI Timespan=All Years*  *Lemmatization=On* |
| # 17 | [407](http://apps.webofknowledge.com/summary.do?product=WOS&doc=1&qid=162&SID=P2jap2m5E6gCG3I2fCM&search_mode=CombineSearches) | #16 OR #15 OR #14 OR #13 OR #12 OR #11 OR #10 OR #9 OR #8 OR #7 OR #6 OR #5  *Databases=SCI-EXPANDED, SSCI Timespan=All Years*  *Lemmatization=On* |
| # 16 | [94](http://apps.webofknowledge.com/summary.do?product=WOS&doc=1&qid=161&SID=P2jap2m5E6gCG3I2fCM&search_mode=AdvancedSearch) | TS=((Maitland or Kaltenborn or Evejenth or Evjenth or Mulligan or McKenzie or Cyriax or Mills or Mennell or Stoddard) NEAR/3 (manipulat* or adjustment* or mobilis* or mobiliz* or traction*))  *Databases=SCI-EXPANDED, SSCI Timespan=All Years*  *Lemmatization=On* |
| # 15 | [24](http://apps.webofknowledge.com/summary.do?product=WOS&doc=1&qid=160&SID=P2jap2m5E6gCG3I2fCM&search_mode=AdvancedSearch) | TS=HVLA  *Databases=SCI-EXPANDED, SSCI Timespan=All Years*  *Lemmatization=On* |
| # 14 | [80](http://apps.webofknowledge.com/summary.do?product=WOS&doc=1&qid=159&SID=P2jap2m5E6gCG3I2fCM&search_mode=AdvancedSearch) | TS="high-velocity low-amplitude"  *Databases=SCI-EXPANDED, SSCI Timespan=All Years*  *Lemmatization=On* |
| # 13 | [118](http://apps.webofknowledge.com/summary.do?product=WOS&doc=1&qid=158&SID=P2jap2m5E6gCG3I2fCM&search_mode=AdvancedSearch) | TS=("body work" OR "body works" OR "body working")  *Databases=SCI-EXPANDED, SSCI Timespan=All Years*  *Lemmatization=On* |
| # 12 | [5](http://apps.webofknowledge.com/summary.do?product=WOS&doc=1&qid=157&SID=P2jap2m5E6gCG3I2fCM&search_mode=AdvancedSearch) | TS=bonesetting  *Databases=SCI-EXPANDED, SSCI Timespan=All Years*  *Lemmatization=On* |
| # 11 | [20](http://apps.webofknowledge.com/summary.do?product=WOS&doc=1&qid=156&SID=P2jap2m5E6gCG3I2fCM&search_mode=AdvancedSearch) | TS="bone setting"  *Databases=SCI-EXPANDED, SSCI Timespan=All Years*  *Lemmatization=On* |
| # 10 | [8](http://apps.webofknowledge.com/summary.do?product=WOS&doc=1&qid=155&SID=P2jap2m5E6gCG3I2fCM&search_mode=AdvancedSearch) | TS=(apophyseal NEAR/1 glide*)  *Databases=SCI-EXPANDED, SSCI Timespan=All Years*  *Lemmatization=On* |
| # 9 | [2](http://apps.webofknowledge.com/summary.do?product=WOS&doc=1&qid=154&SID=P2jap2m5E6gCG3I2fCM&search_mode=AdvancedSearch) | TS="Bowen technique"  *Databases=SCI-EXPANDED, SSCI Timespan=All Years*  *Lemmatization=On* |
| # 8 | [41](http://apps.webofknowledge.com/summary.do?product=WOS&doc=1&qid=153&SID=P2jap2m5E6gCG3I2fCM&search_mode=AdvancedSearch) | TS="myofascial release"  *Databases=SCI-EXPANDED, SSCI Timespan=All Years*  *Lemmatization=On* |
| # 7 | [15](http://apps.webofknowledge.com/summary.do?product=WOS&doc=1&qid=152&SID=P2jap2m5E6gCG3I2fCM&search_mode=AdvancedSearch) | TS=Rolfing  *Databases=SCI-EXPANDED, SSCI Timespan=All Years*  *Lemmatization=On* |
| # 6 | [6](http://apps.webofknowledge.com/summary.do?product=WOS&doc=1&qid=151&SID=P2jap2m5E6gCG3I2fCM&search_mode=AdvancedSearch) | TS=naprapath*  *Databases=SCI-EXPANDED, SSCI Timespan=All Years*  *Lemmatization=On* |
| # 5 | [22](http://apps.webofknowledge.com/summary.do?product=WOS&doc=1&qid=150&SID=P2jap2m5E6gCG3I2fCM&search_mode=AdvancedSearch) | TS=(friction NEAR/1 massage*)  *Databases=SCI-EXPANDED, SSCI Timespan=All Years*  *Lemmatization=On* |
| # 4 | [4,684](http://apps.webofknowledge.com/summary.do?product=WOS&doc=1&qid=149&SID=P2jap2m5E6gCG3I2fCM&search_mode=CombineSearches) | #3 OR #2 OR #1  *Databases=SCI-EXPANDED, SSCI Timespan=All Years*  *Lemmatization=On* |
| # 3 | [39](http://apps.webofknowledge.com/summary.do?product=WOS&doc=1&qid=148&SID=P2jap2m5E6gCG3I2fCM&search_mode=AdvancedSearch) | TS=chirother*  *Databases=SCI-EXPANDED, SSCI Timespan=All Years*  *Lemmatization=On* |
| # 2 | [2,856](http://apps.webofknowledge.com/summary.do?product=WOS&doc=1&qid=147&SID=P2jap2m5E6gCG3I2fCM&search_mode=AdvancedSearch) | TS=chiropractic*  *Databases=SCI-EXPANDED, SSCI Timespan=All Years*  *Lemmatization=On* |
| # 1 | [1,948](http://apps.webofknowledge.com/summary.do?product=WOS&doc=1&qid=146&SID=P2jap2m5E6gCG3I2fCM&search_mode=AdvancedSearch) | TS=osteopath*  *Databases=SCI-EXPANDED, SSCI Timespan=All Years*  *Lemmatization=On* |

MANTIS searched via ChiroAccess (https://www.chiroaccess.com) on 14/09/2011

| (meta analysis[all] OR meta-analys*[all] OR systematic review[all] OR systematic reviews[all]) AND (chiropractic[discipline] OR osteopathic medicine[discipline] OR physical therapy[discipline]) Restrict Search Years to: 2009 to 2011 | 25 |
| --- | --- |
| (random*[all] OR placebo*[all] OR double-blind*[all] OR double blind*[all] or trial*[ti]) AND (chiropractic[discipline] OR osteopathic medicine[discipline] OR physical therapy[discipline]) Restrict Search Years to: 2009 to 2011 | 124 |
| (cohort*[all] OR prospective[all]) AND (chiropractic[discipline] OR osteopathic medicine[discipline] OR physical therapy[discipline]) Restrict Search Years to: 1996 to 2011 | 322 |
| (qualitative[all] OR interview*[all]) AND (chiropractic[discipline] OR osteopathic medicine[discipline] OR physical therapy[discipline]) Restrict Search Years to: 1996 to 2011 | 378 |
| TOTAL  (n.b. it is not possible in MANTIS to combine sets, so I removed duplicates in Reference Manager) | 849 |
| After duplicates removed | **788** |

Index to Chiropractic Literature searched on 15/09/2011 (http://www.chiroindex.org)

| [S1](http://www.chiroindex.org/?action=set&setId=959320) | Subject:"Review Literature as Topic", Year: from 2009 to 2011 | 22 |
| --- | --- | --- |
| [S2](http://www.chiroindex.org/?action=set&setId=959321) | Subject:"Meta-Analysis as Topic", Year: from 2009 to 2011 | 2 |
| [S3](http://www.chiroindex.org/?action=set&setId=959322) | , Year: from 2009 to 2011, Publication Type:Review | 80 |
| [S4](http://www.chiroindex.org/?action=set&setId=959323) | All Fields:"meta analysis", Year: from 2009 to 2011 | 11 |
| [S5](http://www.chiroindex.org/?action=set&setId=959324) | All Fields:"meta analyse", Year: from 2009 to 2011 | 1 |
| [S6](http://www.chiroindex.org/?action=set&setId=959325) | All Fields:"meta analyses", Year: from 2009 to 2011 | 3 |
| [S7](http://www.chiroindex.org/?action=set&setId=959326) | All Fields:"systematic review", Year: from 2009 to 2011 | 29 |
| [S8](http://www.chiroindex.org/?action=set&setId=959327) | All Fields:"systematic reviews", Year: from 2009 to 2011 | 12 |
| [S9](http://www.chiroindex.org/?action=set&setId=959328) | All Fields:search*, Year: from 2009 to 2011 | 86 |
| [S10](http://www.chiroindex.org/?action=set&setId=959329) | S1 OR S2 OR S3 OR S4 OR S5 OR S6 OR S7 OR S8 OR S9 | 131 |
| [S11](http://www.chiroindex.org/?action=set&setId=959331) | Subject:"Randomized Controlled Trials as Topic", Year: from 2009 to 2011 | 6 |
| [S12](http://www.chiroindex.org/?action=set&setId=959332) | , Year: from 2009 to 2011, Publication Type:Randomized Controlled Trial | 24 |
| [S13](http://www.chiroindex.org/?action=set&setId=959333) | , Year: from 2009 to 2011, Publication Type:Controlled Clinical Trial | 1 |
| [S14](http://www.chiroindex.org/?action=set&setId=959334) | All Fields:random*, Year: from 2009 to 2011 | 128 |
| [S15](http://www.chiroindex.org/?action=set&setId=959335) | All Fields:placebo*, Year: from 2009 to 2011 | 16 |
| [S16](http://www.chiroindex.org/?action=set&setId=959336) | All Fields:"double blind", Year: from 2009 to 2011 | 2 |
| [S17](http://www.chiroindex.org/?action=set&setId=959337) | All Fields:"double blinding", Year: from 2009 to 2011 | 0 |
| [S18](http://www.chiroindex.org/?action=set&setId=959338) | All Fields:"double blinded", Year: from 2009 to 2011 | 3 |
| [S19](http://www.chiroindex.org/?action=set&setId=959339) | Article Title:trial*, Year: from 2009 to 2011 | 48 |
| [S20](http://www.chiroindex.org/?action=set&setId=959340) | S11 OR S12 OR S13 OR S14 OR S15 OR S16 OR S17 OR S18 OR S19 | 139 |
| [S21](http://www.chiroindex.org/?action=set&setId=959345) | Subject:"Cohort Studies", Year: from 1996 to 2011 | 8 |
| [S22](http://www.chiroindex.org/?action=set&setId=959347) | Subject:"Prospective Studies", Year: from 1996 to 2011 | 3 |
| [S23](http://www.chiroindex.org/?action=set&setId=959351) | All Fields:cohort*, Year: from 1996 to 2011 | 96 |
| [S24](http://www.chiroindex.org/?action=set&setId=959352) | All Fields:prospective, Year: from 1996 to 2011 | 165 |
| [S25](http://www.chiroindex.org/?action=set&setId=959354) | S21 OR S22 OR S23 OR S24 | 231 |
| [S26](http://www.chiroindex.org/?action=set&setId=959358) | Subject:"Qualitative Research", Year: from 1996 to 2011 | 2 |
| [S27](http://www.chiroindex.org/?action=set&setId=959359) | Subject:"Interviews as Topic", Year: from 1996 to 2011 | 10 |
| [S28](http://www.chiroindex.org/?action=set&setId=959361) | All Fields:interview*, Year: from 1996 to 2011 | 131 |
| [S29](http://www.chiroindex.org/?action=set&setId=959362) | All Fields:qualitative, Year: from 1996 to 2011 | 63 |
| [S30](http://www.chiroindex.org/?action=set&setId=959366) | S26 OR S27 OR S28 OR S29 | 174 |
| [S31](http://www.chiroindex.org/?action=set&setId=959367) | S10 OR S20 OR S25 OR S30 | **593** |

ASSIA via CSA Illumina searched on 16/09/2011

(DE=chiropractic) or(KW=(orthopaedic or orthopedic or chiropract* or chirother* or osteopath* or spine or spinal or vertebra* or craniocervical or craniosacral or "cranio sacral" or cervical or lumbar or occiput or invertebral or thoracic or sacral or sacroilial or joint*) within 3 (manipulat* or adjustment* or mobilis* or mobiliz* or traction*)) or(KW=(manual or manipulat* or mobilis* or mobiliz*) within 3 (therap* or intervention* or treat* or rehab*)) or(DE=("osteopathy" or "cranial osteopathy")) or(KW=("friction massage*" or naprapath* or Rolfing or "myofascial release" or "Bowen technique" or "apophyseal glide*" or "bone setting" or bonesetting or "body work*" or "high-velocity low-amplitude" or HVLA)) or(KW=(Maitland or Kaltenborn or Evejenth or Evjenth or Mulligan or McKenzie or Cyriax or Mills or Mennell or Stoddard) within 3 (manipulat* or adjustment* or mobilis* or mobiliz* or traction*))
