# Supplementary material for: Clinical effectiveness of manual therapy for the management of musculoskeletal and non-musculoskeletal conditions: systematic review and update of UK evidence report
Source: Chiropr Man Therap. 2014 Mar 28;22:12. doi: 10.1186/2045-709X-22-12 (PMC3997823; doi:10.1186/2045-709X-22-12)
Supplement: Additional file 3 — Data tables for included papers – study characteristics, results and conclusions. [file 2045-709X-22-12-S3.docx]

# Additional file 3. Data tables for included papers – study characteristics, results and conclusions

**Table A. Systematic reviews – study characteristics, results and conclusions**

| **Study** | **Inclusion criteria and methodology** | **Included studies** | **Results and Conclusions** |
| --- | --- | --- | --- |
| **Overarching reviews** |  |  |  |
| Brantingham 2012  **Focus:** manipulative therapy (MT) for lower extremity conditions  **Quality:** high | **INCLUSION CRITERIA**  **Study design:** any  **Participants:** peripheral (extremity diagnosis)  **Interventions:** manipulative therapy  **Outcomes:** pain, function  **METHODOLOGY**  5 relevant databases searched, 03/2008 to 5/2011 (review update), English studies only; details on study selection, data extraction, quality assessment; excluded studies not listed.  **Data analysis:** text and tables  **Subgroups / sensitivity analyses:** none | **N included trials:** 48 studies on knee hip, ankle and foot conditions.  **Study quality:** no summary given but quality forms part of the evidence ratings  **Study characteristics:** details given in the tables; n=5 hip, n=20 knee, n=13 ankle, n=11 foot  **Excluded studies eligible for current review:** not reported | - Evidence level of B (fair evidence) for MT combined with multimodal or exercise therapy for short-term treatment of hip osteoarthritis and a level of C (limited evidence) for MT combined with multimodal or exercise therapy for long-term treatment of hip osteoarthritis - Evidence level of B for MT of the knee and/or full kinetic chain and of the ankle and/or foot, combined with multimodal or exercise therapy for short-term treatment of knee osteoarthritis, patellofemoral pain syndrome, and ankle inversion sprain and a level of C for MT of the knee and/or full kinetic chain and of the ankle and/or foot, combined with multimodal or exercise therapy for long-term treatment of knee OA, patellofemoral pain syndrome, and ankle inversion sprain - Evidence level of B for MT of the ankle and/or foot combined with multimodal or exercise therapy for short-term treatment of plantar fasciitis but a level of C for MT of the ankle and/or foot combined with multimodal or exercise therapy for short-term treatment of metatarsalgia and hallux limitus/rigidus and (for a new category) for loss of foot and/or ankle proprioception and balance - Evidence level of I (insufficient evidence) for MT of the ankle and/or foot combined with multimodal or exercise therapy for hallux abducto valgus |
| **Ankle and foot conditions** |  |  |  |
| Lin 2012  **Focus:** rehabilitation for ankle fractures in adults  **Quality:** high | **INCLUSION CRITERIA**  **Study design:** RCTs  **Participants:** patients presenting for rehabilitation following ankle fracture  **Interventions:** any intervention employed by any health professional to assist with rehabilitation following ankle fracture  **Outcomes:** activity limitation, quality of life, patient satisfaction, ankle dorsiflexion and plantarflexion, strength, swelling, adverse events  **METHODOLOGY**  7 relevant databases searched, no date, language or publication restriction; duplicate study selection, data extraction and quality assessment; details on quality assessment and individual studies; excluded studies listed  **Data analysis:** text, tables, meta-analysis  **Subgroups / sensitivity analyses:** rehabilitation after surgical vs after conservative management; true vs quasi-randomisation, concealed versus unconcealed allocation, blind vs non-blind outcome assessment, minimal vs significant drop-outs | **N included trials:** 2 RCTs of manual therapy  **Study quality:** Wilson 1991: 3/6 (high risk of bias), Lin 2008: 6/6 (low risk of bias)  **Study characteristics:** Wilson 1991: n=12, ankle fracture treated with or without surgery, physiotherapy after cast removal, Kaltenborn-based manual therapy, 5 weeks; Lin 2008: n=94; ankle fractures treated with cast immobilisation, start of manual therapy within 7 days of cast removal, large anterior-posterior glides of the talus, 2 sessions a week for 4 weeks versus no manual therapy; standard physiotherapy in both groups  **Excluded studies eligible for current review:** no | **RESULTS**   - Wilson 1991: after 5 weeks’ treatment, no statistically significant improvement in activity limitation or ankle plantarflexion range of motion, ankle dorsiflexion range of motion statistically significant in favour of manual therapy - Lin 2008: no significant difference between groups in functional, pain or quality of life parameters at 24 weeks’ follow-up   **CONCLUSIONS**   - no evidence that manual therapy after a period of immobilisation may improve ankle range of motion in patients after ankle fracture |
| **Carpal tunnel syndrome** |  |  |  |
| Huisstede 2010  **Focus:** effectiveness of non-surgical treatments for carpal tunnel syndrome  **Quality:** medium | **INCLUSION CRITERIA**  **Study design:** systematic reviews or RCTs  **Participants:** patients with carpal tunnel syndrome (not caused by acute trauma or systemic disease)  **Interventions:** any non-surgical  **Outcomes:** pain, function, recovery  **METHODOLOGY**  5 relevant databases searched, no date or language limit; duplicate study selection, data extraction and quality assessment; details on quality assessment and individual studies; excluded studies not listed.  **Data analysis:** text and tables  **Subgroups / sensitivity analyses:** none | **N included trials:** 4 RCTs of manual therapy  **Study quality:** Bialosky 2009, Burke 2007: high quality; Davis 1998, Tal-Akabi 2000: low quality  **Study characteristics:** Tal Akabi 2000: n=21, carpal bone mobilisation versus neurodynamic treatment (median nerve mobilisation) versus control, 3 weeks; Bialosky 2009: n=40, neurodynamic technique plus splinting versus splinting, 3 weeks; Burke 2007; n=22, Graston-instrument assisted soft tissue mobilisation plus exercise versus manual soft tissue mobilisation plus exercise, 6 months; Davis 1998: n=91, chiropractic treatment (manual thrusts, myofascial massage and loading, ultrasound, wrist splint versus medical treatment (ibuprofen) and wrist splint, 13 weeks  **Excluded studies eligible for current review:** not reported | **RESULTS**   - Tal Akabi 2000: carpal bone mobilisation led to significantly greater improvement in symptoms than control; no significant difference between carpal bone mobilisation and neural mobilisation (pain, function, improvement) - Bialosky 2009: no significant differences between groups with respect to pain, disability (Dash questionnaire) or grip strength - Burke 2007: no significant difference between groups with respect to pain, range of motion, grip strength, the Boston Carpal Tunnel questionnaire - Davis 1998: no significant difference for hand function   **CONCLUSIONS**   - limited evidence that carpal bone mobilisation is more effective than no treatment in the short term - no evidence found for the effectiveness of neurodynamic versus carpal bone mobilisation in the short term, for the effectiveness of a neurodynamic technique plus splinting compared with a sham therapy plus splinting group in the short term, or for the effectiveness of Graston instrument-assisted soft tissue mobilisation plus home exercises compared with soft tissue mobilisation plus home exercises to treat carpal tunnel syndrome in the midterm - no evidence for the effectiveness of chiropractic therapy compared with medical treatment for carpal tunnel syndrome in the midterm |
| **Lateral epicondylitis (tennis elbow)** |  |  |  |
| Herd 2008  **Focus:** effectiveness of manipulative therapy in treating lateral epicondylalgia (LE)  **Quality:** medium | **INCLUSION CRITERIA**  **Study design:** RCTs and non-RCTs  **Participants:** adults with LE  **Interventions:** joint manipulation/mobilisation  **Outcomes:** pain, grip strength, pressure pain threshold, range of motion  **METHODOLOGY**  **Data analysis:** narrative, tables, methodological quality assessment PEDro score  **Subgroups / sensitivity analyses:** not reported | **N included trials:** 13  **Study quality:** mean PEDro score 5.15 (1-8)  **Study characteristics:** studies included adult men/women with LE, 5 studies had short-term follow-up (< 3months), 4 studies had long-term follow-up (6 months or longer), and 2 studies had a year-long follow-up  **Excluded studies eligible for current review:** none | **RESULTS**  Mulligan’s mobilisation with movement and MT to the cervical spine were effective  **CONCLUSIONS**  The review identified paucity and low quality of evidence |
| Kohia 2008  **Focus:** effectiveness of various physical therapy (PT) treatments for LE in adults  **Quality:** medium | **INCLUSION CRITERIA**  **Study design:** RCTs  **Participants:** adults with LE  **Interventions:** Cyriax physiotherapy, wrist manipulation, standard physical therapy, ultrasound, bracing, shockwave therapy  **Outcomes:** global improvement, pain, grip strength, pressure pain threshold, range of motion, pain-free grip, quality of life , self-reported progression of the condition  **METHODOLOGY**  **Data analysis:** four relevant databases searched from 1994 to 2006; narrative synthesis, tables; methodological quality assessment using Megens and Harris criteria and Sackett’s hierarchical levels (I-V) and three grades of recommendation (A, B, and C)  **Subgroups / sensitivity analyses:** not reported | **N included trials:** 16  **Study quality:** level I – grade A (7 trials), level II – grade B (9 trials)  **Study characteristics:** randomised studies in LE adults reporting effectiveness of physical therapy interventions such as Cyriax physiotherapy, wrist manipulation, standard physical therapy, ultrasound, bracing, shockwave therapy  **Excluded studies eligible for current review:** none | **RESULTS**  Corticosteroid injections more beneficial versus PT (elbow manipulation and exercise) or Cyriax physiotherapy (6 months or less) (**Grade-A recommendation**); no difference between PT (elbow manipulation and exercise) versus corticosteroid injections or no treatment (6 months or longer) (**Grade-A recommendation**); radial head mobilisation better than standard treatment (ultrasound, massage, stretching, exercise for wrist) in a short-term follow-up (15 weeks); PT protocol (pulsed ultrasound, friction massage, and stretching, exercise for wrist) better than a brace with/without pulsed ultrasound (**Grade-A recommendation**); Cyriax PT better than light therapy, but worse than supervised exercise of wrist extensors; wrist manipulation better than a combination of ultrasound, friction massage, and muscle strengthening (**Grade-B recommendation**)  **CONCLUSIONS**  no single treatment technique shown to be the most effective in treatment of LE |
| Nimgade 2005  **Focus:** the effectiveness of physiotherapy, steroid injections, and relative rest for the treatment of adult LE  **Quality:** medium | **INCLUSION CRITERIA**  **Study design:** RCTs and non-RCTs  **Participants:** adults with LE  **Interventions:** physiotherapy, steroid injections, and relative rest  **Outcomes:** pain, strength, and function  **METHODOLOGY**  Searched 3 databases (for the period of 1966-2004) and bibliographic citations of relevant studies  **Data analysis:** narrative synthesis, tables; methodological quality assessment using the Cochrane Collaboration guidelines for grading controlled trials (internal validity: 11 items, external validity: 6 items, and statistical criteria: 2 items)  **Subgroups / sensitivity analyses:** not reported | **N included trials:** 30  **Study quality:** study quality score ranged from 2 to 9 (out of 11)  **Study characteristics:** randomised and non-randomised studies in LE adults (males and females) reporting effectiveness of physiotherapy, steroid injections, and relative rest  **Excluded studies eligible for current review:** none | **RESULTS**  At 6 weeks, steroid injections and multimodal physiotherapy (arm stretching, strengthening, ultrasound, and massage) were more effective than relative rest.  After 3 months, the multimodal physiotherapy was better than steroid injections, but as effective as relative rest  **CONCLUSIONS**  The active interventions such as steroid injections and multimodal physiotherapy may improve symptoms of LE in adults but this needs to be confirmed in future large and high quality studies |
| Trudel 2004  **Focus:** the effectiveness of conservative treatments for LE in adults  **Quality:** medium | **INCLUSION CRITERIA**  **Study design:** randomised/non-randomised controlled clinical trials  **Participants:** adults with LE  **Interventions:** conservative treatments (e.g., ultrasound, acupuncture, rebox, exercise, wait and see, mobilisation, and/or manipulation, laser)  **Outcomes:** pain, grip strength, pressure pain threshold, range of motion, pain-free grip, muscle function, endurance for activity  **METHODOLOGY**  Searched 4 databases (for the period of 1983 to 2003) and bibliographic citations of relevant studies  **Data analysis:** narrative synthesis, tables; methodological quality assessment using 23 criteria by MacDermid; the evidence was rated using Sackett’s levels of evidence  **Subgroups / sensitivity analyses:** not reported | **N included trials:** 31  **Study quality:** level 2b studies  **Study characteristics:** randomised and non-randomised studies in LE adults (males and females) reporting effectiveness of conservative treatment (physiotherapy, manipulation/mobilisation)    **Excluded studies eligible for current review:** none | **RESULTS**  Mobilisation/manipulation was more effective in improving symptoms of LE compared to placebo or standard physiotherapy. At one year of follow-up, there was no difference between corticosteroid injections and manipulation/mobilisation (Cyriax group)  **CONCLUSIONS**  The authors concluded that level 2b (Sackett’s evidence rating) evidence indicates benefits of mobilisation/manipulation in treating LE |
| **Shoulder conditions** |  |  |  |
| Brantingham 2011  **Focus:** effectiveness of manipulative therapy for shoulder pain and disorders  **Quality:** medium | **INCLUSION CRITERIA**  **Study design:** systematic reviews or primary studies  **Participants:** patients with a shoulder peripheral diagnosis  **Interventions:** manipulative therapy with or without multimodal or adjunctive therapy  **Outcomes:** as reported  **METHODOLOGY**  5 relevant databases searched from 1983, English language; no details on study selection, independent data extraction by three authors; quality assessment using PEDro and whole systems research scores; details on individual studies; excluded studies not listed.  **Data analysis:** text and tables  **Subgroups / sensitivity analyses:** different shoulder disorders | **N included trials:** 23 RCTs, 5 CCTs, 7 before and after studies, case reports and case series  **Study quality:** rotator cuff disorders: 7 high or very high quality studies, 3 medium, 1 low; shoulder complaints / disorders: 6 high or very high, 1 medium; frozen shoulder: 3 high or very high, 3 medium; shoulder soft tissue disorders: 2 high, 1 medium; neurogenic shoulder pain: 2 high; shoulder osteoarthritis: no specific RCTs  **Study characteristics:** n=1 to 172; interventions: mobilisation, manipulation with and without exercise, combined in some studies with soft tissue treatment, mobilisation with movement, myofascial treatments, cervical lateral glide mobilisation  **Excluded studies eligible for current review:** not reported | **RESULTS / CONCLUSIONS**   - *Rotator cuff disorders:* fair evidence for manual and manipulative therapy of the shoulder, shoulder girdle and/or full kinetic chain combined with multimodal or exercise therapy - *Shoulder complaints, dysfunctions, disorders or pain:* fair evidence for manual and manipulative therapy of the shoulder/shoulder girdle and full kinetic chain combined with exercise or a multimodal treatment approach - *Frozen shoulder (adhesive capsulitis):* fair evidence for manual and manipulative therapy of the shoulder, shoulder girdle and/or full kinetic chain combined with multimodal or exercise therapy (manual therapy included high velocity low amplitude manipulation, mid- or end-range mobilisation, mobilisation with movement) - *Shoulder soft tissue disorders:* fair evidence for using soft tissue or myofascial treatments (ischaemic compression, deep friction massage, therapeutic stretch) - *Neurogenic shoulder pain:* limed evidence for cervical lateral glide mobilisation and / or high velocity low amplitude manipulation with soft tissue release and exercise in the treatment of minor neurogenic shoulder pain - *Osteoarthritis of the shoulder:* insufficient evidence (no trials in this patient group) |
| Braun 2009  **Focus:** effectiveness of manual therapy for impingement-related shoulder pain  **Quality:** medium | **INCLUSION CRITERIA**  **Study design:** systematic reviews, RCTs, quasi-RCTs  **Participants:** patients with pain arising locally in a shoulder with grossly abnormal mobility; diagnosed 'shoulder impingement' disorders; shoulder bursitis; tendinitis, tendinopathy and degenerative changes of any rotator cuff muscle; positive findings for 'painful arc'; impingement signs or tests; pain in the shoulder with emphasis on provocation through elevation or lowering of the arm; impaired rotator cuff function or integrity  **Interventions:** manual or exercise therapy compared to any conservative or surgical or no treatment  **Outcomes:** pain, function, disability, symptoms, quality of life, range of motion, strength, work absenteeism, costs, adverse events  **METHODOLOGY**  6 relevant databases searched, primary studies post cut-off dates of reviews (Jan 2005) to Oct 2008, English or German; duplicate selection or data extraction not mentioned; quality assessment using AMSTAR and PEDro scale; details on quality assessment and individual studies; excluded studies listed.  **Data analysis:** text and tables  **Subgroups / sensitivity analyses:** none | **N included trials:** 8 systematic reviews, 6 RCTs  **Study quality:** both systematic reviews and RCTs had a range of quality deficits  **Study characteristics:** n=30 to 112, 3 RCTs included exercise only, 3 included exercise and manual therapy (mobilisation)  **Excluded studies eligible for current review:** no | **RESULTS**   - 5 reviews: evidence to favour manual therapy plus exercise over exercise alone - Evidence of three relevant additional trials inconclusive (with a tendency towards improved outcomes with manual therapy and exercise) - No evidence found for the effectiveness of mobilisation alone - None of the systematic reviews and only one of the RCTs included a specific statement on adverse events; in the one RCT no adverse events were reported   **CONCLUSIONS**  There is limited evidence to support the effectiveness of manual therapy and exercise interventions for impingement-related shoulder pain; this primarily relates to subacute and chronic complaints and short and medium term effectiveness; the conclusions are based on research of varying methodological quality, with varying risk of bias, and are affected by weaknesses in the reporting quality; cautious interpretation is warranted due to heterogeneity of populations, interventions and outcomes |
| Camarinos 2009  **Focus:** effectiveness of manual physical therapy for painful shoulder conditions  **Quality:** medium | **INCLUSION CRITERIA**  **Study design:** RCTs  **Participants:** adults 18 to 80 years with shoulder  **Interventions:** physical therapy for conservative management of shoulder pain, treatment by physical therapists; the interventions of interest were manual therapy interventions including low and high velocity mobilisations directed to the glenohumeral joint *without* additional mobilisation of adjacent structures  **Outcomes:** active or passive range of motion, a functional outcome measure specific to the shoulder, quality of life measure, pain measure  **METHODOLOGY**  4 relevant databases searched, English language, published between 1996 and 2009; reference lists, hand searching of a couple of relevant journals; study selection, data extraction and quality assessment by more than one author; details on quality assessment (PEDro scores) and individual studies; excluded studies not listed.  **Data analysis:** text and tables  **Subgroups / sensitivity analyses:** none | **N included trials:** 7 RCTs  **Study quality:** average PEDro score 7.86, range 6 to 9  **Study characteristics:** participants: n=14 to 100, interventions: mobilisation with movement, Cyriax approach, static mobilisation performed at end-range or mid-ranges of motion  **Excluded studies eligible for current review:** none | **RESULTS**   - *Mobilisation with movement (n=3):* significant improvement in range of motion in two of three studies, highest percentage change in range of motion in third study; significant improvement in pain in one of two studies; significant functional improvement in one study and highest percentage change in function in second study - *Cyriax manual therapy (n=1):* significant improvement in range of motion compared to control - *Mobilisations at end-range of motion (n=3):* improvement in range of motion and end-range mobilisation reported in all studies; two studies reported no significant difference in pain measures, two of three studies reported significantly improved function compared to control - *Mid-range mobilisation (n=4):* no effect on range of motion, only one reported a significant improvement in pain and none reported a significant difference in function   **CONCLUSIONS**  The included studies demonstrated a benefit of manual therapy for improvements in mobility and a trend in improving pain measures, while increases in function and quality of life were questionable |
| Pribicevic 2010  **Focus:** effectiveness of manipulative therapy for the treatment of shoulder pain  **Quality:** medium | **INCLUSION CRITERIA**  **Study design:** case reports, case series, RCTs  **Participants:** patients with shoulder pain or related specific clinical diagnosis; adhesive capsulitis excluded  **Interventions:** treatment by registered practitioner of chiropractic, physiotherapy or medicine; treatment typical of the profession and included manipulative thrust technique  **Outcomes:** any outcomes  **METHODOLOGY**  5 relevant databases searched, from 1985, English language; bibliographies searched; methods of study selection and data extraction unclear; quality assessment using PEDro scale; details on quality assessment and individual studies; excluded studies not listed.  **Data analysis:** text and tables  **Subgroups / sensitivity analyses:** none | **N included trials:** 22 case reports, 4 case series, 4 RCTs  **Study quality:** RCTs scored 5 to 8 out of 10  **Study characteristics:** case reports and case series all of chiropractic treatment; RCTs: n=15 to 172, interventions: 1 RCT with chiropractic manipulations, 3 with physiotherapeutic manipulations  **Excluded studies eligible for current review:** not reported | **RESULTS**   - Munday 2007: manipulation superior to placebo in the short term treatment of shoulder impingement syndrome - Winters 1997: manipulation significantly better than classic physiotherapy in reducing pain and recurrence (general shoulder complaints) - Bergman 2004: after 12 weeks significantly more patients in the manipulation than usual care group reported full recovery or very large improvement; no difference at 12 months (shoulder dysfunctions) - Savolainen 2004: at 12 months, VAS pain was reduced in favour of the thoracic manipulation group (neck and shoulder pain in occupational health)   **CONCLUSIONS**  Evidence is limited, only two RCTs of reasonably sound methodology; need for well-designed trials investigating multi-modal chiropractic treatment |
| **Cervicogenic headache** |  |  |  |
| Posadzki 2011  **Focus:** effectiveness/safety of spinal manipulation therapy (SMT) in cervicogenic headache (CGH)  **Quality:** high | **INCLUSION CRITERIA**  **Study design:** RCTs  **Participants:** adults with CGH  **Interventions**: manipulative procedures (chiropractic, osteopathy)  **Outcomes:** headache intensity, duration, frequency  **METHODOLOGY**  7 relevant databases searched; no language limit; some details on study selection; quality assessment of studies presented; studies not presenting original data, abstracts, conference proceedings, outcomes of interest not reported were excluded; excluded studies not listed  **Data analysis:** text and tables  **Subgroups / sensitivity analyses:** not reported | **N included studies:** 9 RCTs  **Study quality:** Cochrane Risk of Bias tool and Jadad score; most trials had major methodological flaws; two trials (Borusiak 2010 and Jull 2002) had low risk of bias with Jadad score of 4 and three trials (Bitterli 1977, Howe 1983, Li 2007) had high risk of bias with Jadad score of 0-1  **Study characteristics:** populations across studies were relatively homogenous, but control interventions were different ranging from sham manipulation, light massage, drugs, physical therapy to no intervention  **Excluded studies eligible for current review:** not reported | **RESULTS**  6 trials, which were conducted by chiropractors, suggested the benefit of SMT in treating the headaches over physical therapy, light massage, drug therapy, or no intervention. The remaining 3 trials, which were conducted by non-chiropractors, showed no significant difference in headache intensity, duration, or frequency between SMT and placebo, physical therapy, massage, or wait list controls  **CONCLUSIONS**  Given the clinical heterogeneity, inconsistency in results, and low methodological quality of the reviewed studies, the evidence regarding the effectiveness of SMT for CGH is rendered inconclusive |
| **Miscellaneous headaches** |  |  |  |
| Bryans 2011  **Focus:** effectiveness/safety of spinal manipulation therapy (SMT), mobilisation, or manual traction in adults with miscellaneous headaches (migraine, tension-type headache, cervicogenic headache)  **Quality:** high | **INCLUSION CRITERIA**  **Study design:** systematic reviews, RCTs, CCTs  **Participants:** adults with miscellaneous headaches (migraine, tension-type headache, cervicogenic headache)  **Interventions**: spinal manipulation therapy (SMT), mobilisation, or manual traction  **Outcomes:** headache intensity, duration, frequency, quality of life, disability, medicine use  **METHODOLOGY**  8 relevant databases searched; English publications; hand search of reference lists; details on study selection; quality assessment of studies presented; excluded studies and reasons for exclusions are listed; assessed strength of evidence using pre-defined rules and recommendations for practice are developed  **Data analysis:** text and tables  **Subgroups / sensitivity analyses:** not reported | **N included studies:** 11 RCTs, 5 controlled trials, and 5 systematic reviews  **Study quality:** the Cochrane Collaboration Back Review Group (controlled studies; score range: 3-9) and Oxman and Guyatt (systematic reviews; score range: 6-9)  **Study characteristics:** studies differed in inclusion criteria and included adults with miscellaneous headaches (migraine, tension-type headache, or cervicogenic headache). Most studies reported pain relief, pain duration, frequency, pain medication use, and quality of life  **Excluded studies eligible for current review:** not reported | **RESULTS**   - Spinal manipulation was shown beneficial for adults with episodic/chronic migraine and cervicogenic headache, but not for those with episodic tension-type headache - Craniocervical mobilisation and joint mobilisation were effective for episodic/chronic tension-type headaches and cervicogenic headache, respectively - It is not clear if spinal manipulation, manual traction, connective tissue manipulation, Cyriax’ mobilisation or exercise are effective for tension-type headaches - Risks of harms reported in 6 trials were low   **CONCLUSIONS**   - The guideline panel recommend the use of spinal manipulation for the management of adults with episodic/chronic migraine (moderate evidence level) and cervicogenic headache (moderate evidence level) - The guideline panel cannot recommend the use of spinal manipulation for the management of episodic tension-type headache (moderate evidence level) - The guideline panel recommend the use of craniocervical mobilisation and joint mobilisation for episodic/chronic tension-type headaches and cervicogenic headache, respectively - No recommendation on spinal manipulation, manual traction, connective tissue manipulation, Cyriax’ mobilisation or exercise for chronic tension-type headache |
| **Fibromyalgia** |  |  |  |
| Terhorst 2011  **Focus:** effectiveness of complementary and alternative medicine in the treatment of pain in fibromyalgia  **Quality:** medium | **INCLUSION CRITERIA**  **Study design:** RCTs  **Participants:** adults diagnosed with fibromyalgia according to the American College of Rheumatology, Yunus, or Smythe criteria  **Interventions**: a complementary or alternative therapy compared to control  **Outcomes:** pain  **METHODOLOGY**  11 relevant databases searched; hand search of reference lists; details on study selection; quality assessment of studies presented; study details shown  **Data analysis:** text and tables  **Subgroups / sensitivity analyses:** not reported, analysis by type of therapy | **N included studies:** 3 small RCTs on manipulative therapy  **Study quality:** manipulative therapy: 2 low and 1 moderate quality  **Study characteristics:** 1 trial of chiropractic versus waiting list (n=19), 1 trial of osteopathy versus treatment as usual (n=12), 1 trial of chiropractic plus resistance training versus resistance training (n=21)  **Excluded studies eligible for current review:** not reported | **RESULTS**   - The two chiropractic studies had significant effects in favour of the intervention   **CONCLUSIONS**  No overall conclusions can be drawn, as there was only a very limited number of very small studies on manipulative therapy  **Research recommendations**  Patients with fibromyalgia should be subdivided into subgroups based on baseline characteristics; studies with larger sample sizes are needed |
| **Asthma** |  |  |  |
| Kaminskyj 2010  **Focus:** effectiveness of chiropractic treatment for asthma  **Quality:** medium | **INCLUSION CRITERIA**  **Study design:** prospective and retrospective studies including RCTs, controlled clinical/quasi-experimental trials; cohort, case-control, case series and survey designs  **Participants:** patients diagnosed with asthma  **Interventions:** chiropractic treatment  **Outcomes:** any outcome relevant to asthma or breathing  **METHODOLOGY**  7 databases searched, hand-searching of conference proceedings, bibliographies of relevant articles; search terms not shown; unclear if duplicate study selection; description of quality assessment; unclear if duplicate validity assessment and data extraction  **Limitations:** English language, published 1980 to March 2009  **Data analysis:** text and tables  **Subgroups / sensitivity analyses:** none | **N included trials:** 8 (3 RCTs, 1 CCT, 1 case study, 1 case series, 2 surveys)  **N participants:** 275 plus 5607 from one survey  **Trial quality:** four studies <10/27 on Down’s and Black checklist, four studies ≥15/27  **Study characteristics:** 3 studies in children (1 to 17 years); in all comparative trials the comparator was sham treatment; treatment in comparative studies up to 4 months  **Excluded studies eligible for current review:** none  **Further information available on:** study characteristics, individual study results, study quality | **RESULTS**   - in comparative studies, no significant differences between comparison groups in respiratory parameters, symptoms or subjective measures - in uncontrolled studies, improvements were generally seen in subjective measures (symptoms), but some improvement in peak flow was also seen; subjective improvements were also in control groups of comparative studies - no adverse effects seen (but only reported by one study)   **CONCLUSIONS**  Some patients may experience chiropractic care as beneficial, but overall no significant effect in any outcomes versus sham treatment; low quality evidence  **Research recommendations**  More evidence required using valid and reliable outcome measurement |
| **ADHD / learning disabilities** |  |  |  |
| Karpouzis 2010  **Focus:** systematic review of chiropractic treatment for attention deficit / hyperactivity disorder in children or adolescents  **Quality:** medium | **INCLUSION CRITERIA**  **Study design:** systematic reviews, randomised or quasi-randomised controlled trials, comparative studies with or without concurrent controls  **Participants:** children aged 0 to 17 years; diagnosis of attention deficit / hyperactivity disorder (ADHD) consistent with DSM-III, DSM-IV, DSM-IV-TR or ICD-10 criteria; diagnosis by paediatrician, psychiatrist, medical doctor, clinical or educational psychologist  **Interventions:** chiropractic treatment  **Outcomes:** validated psychometric outcome measure as recommended by the American Academy of Child and Adolescent Psychiatry  **METHODOLOGY**  9 databases searched, hand-searching of 2 journals; partial duplicate study selection; description of quality assessment (Jadad and 15-item checklist by Hawk); list of excluded studies  **Limitations:** full text, English language  **Data analysis:** text and tables  **Subgroups / sensitivity analyses:** none | **Number of included trials:** none  **Number of participants:** none  **Trial quality:** only low quality studies identified that did not fulfil inclusion criteria  **Study characteristics:** NA  **Excluded studies eligible for current review:** none  **Further information available on:** ADHD rating scales, characteristics of excluded studies | **RESULTS**  None of the identified studies fulfilled the inclusion criteria  **CONCLUSIONS**  There is no high quality evidence to evaluate the efficacy of chiropractic care for paediatric and adolescent ADHD; the claims made by chiropractors that chiropractic care improved ADHD symptomatology for young people is only supported by low levels of scientific evidence (e.g. case reports, case series)  **Research recommendations**  Adequately-sized RCTs using clinically relevant outcomes and standardised measures to examine the effectiveness of chiropractic care versus non-treatment/placebo control or standard care are needed |
| **Cancer care** |  |  |  |
| Alcantara 2011  **Focus:** chiropractic care of patients with cancer  **Quality:** low | **INCLUSION CRITERIA**  **Study design:** any type of primary study  **Participants:** patients with cancer  **Interventions:** chiropractic care  **Outcomes:** not specified  **METHODOLOGY**  9 relevant databases searched, 4 journals hand searched, bibliographies searched, no date limit; studies selected independently by two authors, no details on data extraction; no quality assessment; excluded studies not listed; no systematic tabulation of studies.  **Data analysis:** text  **Subgroups / sensitivity analyses:** none | **N included trials:** 60 case reports, 2 case series, 21 commentaries, 2 survey studies, 2 reviews  **Study quality:** not reported  **Study characteristics:** no high quality studies included, no effects on patient outcomes reported  **Excluded studies eligible for current review:** not reported | **RESULTS / CONCLUSIONS**  Patients with cancer seek care from chiropractors but the effects of such care were not described |
| **Cervicogenic dizziness / balance** |  |  |  |
| Lystad 2011  **Focus:** effects of manual therapy with or without vestibular rehabilitation in the management of cervicogenic dizziness  **Quality:** high | **INCLUSION CRITERIA**  **Study design:** prospective controlled or non-controlled intervention studies  **Participants:** patients with cervicogenic dizziness  **Interventions:** manual therapy (spinal manipulation or mobilisation) alone or manual therapy in combination with vestibular rehabilitation (exercise-based)  **Outcomes:** as reported by the studies  **METHODOLOGY**  4 relevant databases searched, website searches, bibliographies and relevant reviews searched, no language restriction, no date limit; studies selected independently by two authors; data extraction in a spreadsheet; quality assessment using the Maastricht-Amsterdam criteria (by two reviewers independently; excluded studies listed; systematic tabulation of studies.  **Data analysis:** text and tables  **Subgroups / sensitivity analyses:** none | **N included trials:** 5 RCTs, 8 non-controlled cohort studies  **Study quality:** RCTs: 1 good quality (Reis 2008), 4 moderate quality; cohort studies: all poor quality  **Study characteristics:** participants: sample sizes 12 to 168; interventions: 6 studies (2 RCTs) used only manipulation and /or mobilisation, self-mobilising apophyseal glides in 1 RCT (Reid 2008), 7 studies (3 RCTs) used multi-modal approach (several different interventions and home exercise programme), none used manual therapy in conjunction with vestibular rehabilitation  **Excluded studies eligible for current review:** no | **RESULTS**   - 12 studies (all 5 RCTs) found improvement in dizziness and associated symptoms after manual therapy - 2 RCTs found improvement in balance performance (posturography) - Only 3 studies reported adverse events: no adverse events in 2 RCTs, minor adverse events in one cohort study   **CONCLUSIONS**  There is moderate evidence in a favourable direction to support the use of manual therapy (spinal mobilisation and / or manipulation) for cervicogenic dizziness; research needed on combining manual therapy with vestibular rehabilitation |
| **Chronic fatigue syndrome / myalgic encephalomyelitis** |  |  |  |
| Porter 2010  **Focus:** alternative medical interventions in the treatment / management of myalgic encephalomyelitis and fibromyalgia (emphasis in this table on the former)  **Quality:** high | **INCLUSION CRITERIA**  **Study design:** RCTs and CCTs  **Participants:** patients with myalgic encephalitis / chronic fatigue syndrome according to established case definitions  **Interventions:** CAM interventions as defined by the National Center for Complementary and Alternative Medicine  **Outcomes:** laboratory test results, physical functioning, psychologic functioning, quality of life  **METHODOLOGY**  5 relevant databases searched, website searches, 2 journals hand searched, bibliographies searched, no date limit; studies selected independently by four authors; data extraction conducted by one reviewer and checked by another; quality assessment using the Jadad scale; excluded studies listed; systematic tabulation of studies.  **Data analysis:** text and tables  **Subgroups / sensitivity analyses:** none | **N included trials:** 1 RCT for manual therapy in myalgic encephalomyelitis  **Study quality:** low  **Study characteristics:** osteopathic manual therapy in 58 patients with myalgic encephalomyelitis compared to no treatment  **Excluded studies eligible for current review:** no | **RESULTS**  Trial showed overall beneficial effects and improvement in symptoms  **CONCLUSIONS**  Osteopathic manual therapy may have potential for future high quality clinical research |
| **Chronic pelvic pain** |  |  |  |
| Franke 2013  **Focus:** effects of osteopathic treatment on female lower urinary tract disorders  **Quality:** high | **INCLUSION CRITERIA**  **Study design:** RCTs and CCTs  **Participants:** women, >18 years, with a diagnosed female urination disorder; exclusions: neurologic disorders, tumours, urinary tract infections, pregnancy  **Interventions:** osteopathic treatments  **Outcomes:** urologic symptoms  **METHODOLOGY**  7 relevant databases searched, ongoing trials searched, keywords shown, citation tracking, bibliographies searched; studies selected independently by 2 authors; data extraction and quality assessment conducted independently by 2 reviewers; quality assessment using the Cochrane Risk of Bias Tool; systematic tabulation of studies.  **Data analysis:** meta-analysis; text and tables  **Subgroups / sensitivity analyses:** none | **N included trials:** 2 RCTs, 3 CCTs  **Study quality:** 4 studies with low risk of bias, 1 with high risk of bias  **Study characteristics:** studies addressed voiding dysfunction, urinary incontinence, urge and stress incontinence; number of participants: 47 to 90; interventions: osteopathic manual therapy compared to no treatment or pelvic floor muscle training; duration: 4 to 12 weeks  **Excluded studies eligible for current review:** not reported | **RESULTS**   - The osteopathic treatment groups should a significant improvement in symptoms compared to control (effect size -3.38 (95% CI: -5.46, -1.31, p=0.001) overall, effect size -6.34 (95% CI: -10.85, -1.84, p=0.008) when compared to no treatment) - There was no significant difference when comparing osteopathic treatment with pelvic floor muscle training   **CONCLUSIONS**  The studies point to an improvement of the symptoms associated with female lower urinary tract disorders with osteopathic treatment |
| Loving 2012  **Focus:** effects of physiotherapy in the management of adult female chronic pelvic pain  **Quality:** high | **INCLUSION CRITERIA**  **Study design:** prospective quantitative study design  **Participants:** women, >19 years, with chronic pelvic pain (pelvic adhesion, pelvic congestion syndrome, bladder pain syndrome, urethral pain syndrome and irritable bowel syndrome); exclusions: malignancy, primary dysmenorrhoea, endometriosis, pregnancy, infections, active chronic pelvic inflammatory disease and vulvodynia/vulvar pain syndrome; exclusions: neurologic disorders, tumours, urinary tract infections, pregnancy  **Interventions:** physiotherapeutic intervention alone or in combination with other medical or psychological therapies  **Outcomes:** pain, quality of life, physical activity  **METHODOLOGY**  8 relevant databases searched, keywords listed, bibliographies searched, authors contacted; obviously irrelevant studies removed by one author, remainder independently selected by 2 authors; data extracted by one author and checked by a second; quality assessment using the Cochrane Risk of Bias Tool; systematic tabulation of studies.  **Data analysis:** text and tables  **Subgroups / sensitivity analyses:** none | **N included trials:** 6 RCTs, 1 cohort study, 3 case-series; 3 RCTs relevant to manual therapy  **Study quality:** 3 RCTs with low risk of bias and 3 RCTs with high risk of bias  **Study characteristics:** sample sizes 21 to 370; mean ages 30.5 to 43 years; 6 studies minimum duration 6 months of chronic pelvic pain symptoms; manual therapy interventions of 3 relevant RCTs: myofascial therapy, chiropractic techniques and manual trigger point therapy, distension of pelvic floor  **Excluded studies eligible for current review:** not reported | **RESULTS**   - There was level 1d evidence (high risk of bias) that physiotherapeutic distension of painful pelvic structures combined with pain counselling improves pain experience compared to treatment as usual - No evidence was found for myofascial therapy or chiropractic treatment   **CONCLUSIONS**  The authors did not find any convincing evidence for the use of manual therapy in female chronic pelvic pain; they concluded that there was some evidence to support the use of multidisciplinary intervention approaches |
| **Gastrointestinal disorders** |  |  |  |
| Ernst 2011  **Focus:** effectiveness of spinal manipulation in patients with gastrointestinal disorders  **Quality:** medium | **INCLUSION CRITERIA**  **Study design:** controlled studies  **Participants:** studies concerning any gastrointestinal disorders  **Interventions**: manual procedures  **Outcomes:** pain relief, symptom severity, clinical remission  **METHODOLOGY**  6 relevant databases searched; no language limit; some details on study selection and data extraction; studies of infant colic were excluded; excluded studies not listed  **Data analysis:** text and tables  **Subgroups / sensitivity analyses:** none | **N included trials:** 2 controlled trials: 1 RCT and 1 non-RCT  **Study quality:** Jadad score (0-1); Hains 2007 low quality (Jadad score 1), Pikalov 1994 low quality (Jadad score 0)  **Study characteristics:** Hains 2007: 62 adults with gastro-oesophageal reflux disease treated with spinal manipulation versus ischaemic compression for 7 weeks (20 sessions); Pikalov 1994: 35 adults with duodenal ulcer treated with spinal manipulation (3-14 sessions; duration: not reported) plus conventional treatment versus conventional treatment only  **Excluded studies eligible for current review:** not reported | **RESULTS**  No significant differences in outcome measures (symptom severity score, clinical parameters) between the manual therapy and control groups  **CONCLUSIONS**  Evidence is inconclusive based on two low quality studies; it cannot be established whether manual therapy is more effective than ischaemic compression or conventional treatment in patients with gastrointestinal disorders |
| **Hypertension** |  |  |  |
| Mangum 2012  **Focus:** effects of spinal manipulative therapy for hypertension  **Quality:** medium | **INCLUSION CRITERIA**  **Study design:** observational or therapy trial  **Participants :** patients with hypertension  **Interventions:** spinal manipulative therapy  **Outcomes:** blood pressure  **METHODOLOGY**  5 relevant databases searched, non-English studies and abstracts excluded; studies selected by three authors; quality rated by all authors, data extraction unclear; quality assessment using the Cochrane Risk of Bias tool; excluded studies not listed; systematic tabulation of studies.  **Data analysis:** text and tables  **Subgroups / sensitivity analyses:** none | **N included trials:** 10 studies, but only results for 5 studies with low or unclear risk of bias reported (5 RCTs, 2 non-randomised CCTs, 3 case reports)  **Study quality:** of RCTs, 2 low risk of bias, 3 unclear risk of bias  **Study characteristics:** 21 to 128 patients included; spinal manipulative treatment (SMT) single session to up to 20 treatments over 2 months; types of SMT: Gonstead chiropractic adjusting, NUCCA technique, “diversified adjustments”, Activator instrument, osteopathic manipulative therapy  **Excluded studies eligible for current review:** not reported | **RESULTS**   - Goertz 2002, low risk of bias, 12 sessions of “diversified adjustments” plus diet versus diet only - Plaugher 2002, low risk of bias, Gonstead chiropractic adjusting (up to 20 treatments), versus brief massage or control - Bakris 2007, unclear risk of bias, SMT NUCCA technique weekly for 8 weeks (but 85% had only one adjustment) - Abram 1988, unclear risk of bias, single Activator SMT versus placebo and no treatment - Morgan 1985, unclear risk of bias, cross-over, 6 weeks osteopathic manipulative therapy versus sham massage  \| **Study** \| **Intervention BP, study end (mmHg, 95% CI)** \| **Control BP, study end**  **(mmHg, 95% CI)** \| **p** \| \| --- \| --- \| --- \| --- \| \| Goertz 2002 \| SP -3.5 (-5.7 to -1.3)  DP -4.0 (-5.3 to -2.7) \| SP -4.9 (‑6.7 to ‑3.1)  DP -5.6 (‑6.8 to ‑4.4) \| NS \| \| Plaugher 2002 \| SP -2.3 (-6.4 to +1.8)  DP -4.8 (-12.6 to +3.0) \| No treatment  SP -7.7 (-14.5 to -0.9)  DP -9.0 (-16.8 to -1.2)  Brief massage  SP -1.3 (-9.4 to +11.9)  DP -1.7 (-6.2 to +2.9) \| NS \| \| Bakris 2007 \| SP -17.2 (-20.7 to -13.7)  DP -10.3 (-14.6 to -6.0) \| SP -3.2 (-7.5 to +1.1)  DP -1.8 (-4.5 to +0.9) \| <0.05 \| \| Abram 1988 \| SP -14.7 (-17.3 to -12.1)  DP -13.0 (-15.4 to -10.6) \| Placebo  SP +1.4 (-3.2 to +6.0)  DP -1.4 (-3.3 to +0.5) \| <0.05 \| \| Morgan 1985 \| First half of cross-over  SP -6.3 (-12.2 to -0.4)  DP -3.6 (-8.5 to +1.3) \| First half of cross-over  SP -0.2 (-2.4 to +2.0)  DP -0.5 (-3.2 to +2.2) \| NS \|   SP: systolic blood pressure, DP: diastolic blood pressure  **CONCLUSIONS**  There is lack of low bias evidence to support the use of spinal manipulative therapy for the treatment of hypertension; further high quality evidence is needed |
| **Infantile colic** |  |  |  |
| Dobson 2012  **Focus:** Cochrane review of manipulative therapies for infantile colic  **Quality:** high | **INCLUSION CRITERIA**  **Study design:** RCTs  **Participants:** infants <6 months with colic (defined as 'crying excessively')  **Interventions:** manipulative therapies of chiropractic, osteopathy, cranial osteopathy, craniosacral therapy and cranial manipulation, compared with no treatment, placebo/sham, standard care or waiting list control  **Outcomes:** hours crying time, presence / absence of colic, frequency of crying, parental/family quality of life, parental stress / anxiety / depression, sleeping time, parental satisfaction, adverse outcomes  **METHODOLOGY**  18 relevant databases searched, up to spring 2012; search strategy shown; wide range of supplementary sources searched; bibliographies searched; independent study selection by two reviewers; independent data extraction and quality assessment by two reviewers; excluded studies listed.  **Data analysis:** meta-analysis; tables and text  **Subgroups / sensitivity analyses:** planned, but only limited numbers of studies | **N included trials:** 6 RCTs  **Study quality:** 2 RCTs with deficit in allocation concealment, 4 RCTs with deficits in blinding of participants / personnel, 3 RCTs with deficits in blinding of outcome assessment, 5 RCTs with (potential) attrition and reporting bias  **Study characteristics:** 28 to 100 infants randomised (age birth to 12 weeks); interventions: chiropractic in 4 RCTs, 1 RCT each osteopathy and cranial osteopathy; generally 3 to 5 treatments  **Excluded studies eligible for current review:** no | **RESULTS**   - Change in daily hours crying (n=5 RCTs) weighted mean difference (WMD) -1.20 (95% CI: -1.89, -0.51) - One study reported on significant improvements of sleeping time with treatment (mean difference 1.17 (95% CI: 0.22, 2.12, p=0.02) - No significant difference found for 'full recovery' - Only one study reported on adverse events and none were recorded   **CONCLUSIONS**  The studies involved too few participants and were of insufficient quality to draw confident conclusions about the usefulness and safety of manipulative therapies. |
| **Insomnia** |  |  |  |
| Kingston 2010  **Focus:** chiropractic as a treatment for primary insomnia  **Quality:** low | **INCLUSION CRITERIA**  **Study design:** RCTs and case studies  **Participants:** primary insomnia  **Interventions:** chiropractic spinal manipulative therapy  **Outcomes:** at least one patient outcome measure (e.g. sleep diaries, Pittsburgh Sleep Quality index)  **METHODOLOGY**  4 relevant databases searched, up to 2006; obviously no systematic development of search strategy; hand searching of potentially relevant journals (not specified); independent study selection by two reviewers; no details on data extraction; no details on quality assessment; excluded studies not listed.  **Data analysis:** text  **Subgroups / sensitivity analyses:** none | **N included trials:** 15 studies meeting the selection criteria mentioned (but they do not all seem to have been relevant), none of the studies was an RCT and only one had a control group  **Study quality:** not reported but obviously low  **Study characteristics:** no systematic reporting or tabulation; Cutler 2005 investigated cranial osteopathic manipulation but outcome reporting appears not to have been consistent  **Excluded studies eligible for current review:** not reported | **RESULTS / CONCLUSIONS**  There is minimal evidence to support chiropractic treatment for primary insomnia; high quality trials are needed |
| **Otitis media** |  |  |  |
| Pohlmann 2012  **Focus:** systematic review of spinal manipulative therapy for otitis media in children  **Quality:** high | **INCLUSION CRITERIA**  **Study design:** any  **Participants:** acute or chronic otitis media in children ≤6 years  **Interventions:** spinal manipulative therapy  **Outcomes:** (not specified, as reported by included studies) infection, pain, auditory function, adverse events  **METHODOLOGY**  6 relevant databases searched, reference lists searched, English studies only; studies selected and quality assessed independently by two authors; quality assessment using a range of checklists depending on study type; systematic tabulation of studies.  **Data analysis:** text and tables  **Subgroups / sensitivity analyses:** none | **N included trials:** 15 case reports, 5 case series, 3 controlled trials, 8 reviews  **Study quality:** controlled trials: 2 excellent quality, one good quality  **Study characteristics:** between 20 and 84 participants; one each acute, recurrent and chronic otitis media; two on osteopathic manipulation and one on chiropractic manipulation  **Excluded studies eligible for current review:** not reported | **RESULTS**   - Only one of the three controlled trials showed decreased symptoms of otitis media with manipulation - Only minor adverse events were seen   **CONCLUSIONS**  There is no evidence to support or refute using spinal manipulative therapy for otitis media |
| **Pneumonia and other respiratory diseases** |  |  |  |
| Heneghan 2012  **Focus:** effectiveness of manual therapy for chronic obstructive pulmonary disease (COPD)  **Quality:** medium | **INCLUSION CRITERIA**  **Study design:** RCTs, non-randomised controlled trials, before-and-after studies  **Participants:** adults with known history of chronic obstructive airways disease, including patients described as having COPD, emphysema and chronic bronchitis  **Interventions:** manual therapy versus control, sham, or alternative manual therapy  **Outcomes:** any lung function parameter  **METHODOLOGY**  6 relevant databases searched, bibliographies searched, keywords given; English articles only; irrelevant studies excluded by one reviewer, full text articles evaluated by two independent reviewers; independent data extraction by two reviewers; study quality assessed according to Cochrane guidelines; systematic tabulation of studies.  **Data analysis:** text and tables  **Subgroups / sensitivity analyses:** none | **N included trials:** 5 RCTs, 2 before-and-after studies  **Study quality:** 6 with high risk of bias, 1 with low risk of bias  **Study characteristics:** sample size 5 to 35; most focussed on participants with mild to moderate COPD; interventions: 4 studies used osteopathic manipulation, 1 study each used massage, muscle stretching, and passive movements  **Excluded studies eligible for current review:** no | **RESULTS**   - Performance based measures of pulmonary function (FEV1, FVC) changed minimally (<1.5%) with osteopathic manual therapy - Patient reported measures for ‘improved health’ and ‘breathing difficulty’ however did improve following osteopathic manual therapy compared to control   **CONCLUSIONS**  Evidence for manual therapy as an adjunctive management approach for COPD is lacking. More exploratory research is first required to better understand the nature and extent of changes in the musculoskeletal system in patients with COPD and their possible relationship with pulmonary function. |
| Yang 2010/2013  **Focus:** Cochrane review of chest physiotherapy for pneumonia in adults  **Quality:** high | **INCLUSION CRITERIA**  **Study design:** RCTs  **Participants:** adults with any type of pneumonia  **Interventions:** chest physiotherapy (including osteopathy)  **Outcomes:** mortality, cure rate, duration of hospital stay, healing time, rate of improvement of chest X-ray, and various other secondary outcomes  **METHODOLOGY**  6 relevant databases searched, journals hand searched, no language or publication restrictions; studies selected and data extracted independently by two authors; quality assessment using the Cochrane risk of bias instrument; excluded studies listed; systematic tabulation of studies.  **Data analysis:** meta-analyses; text and tables  **Subgroups / sensitivity analyses:** different types of chest physiotherapies | **N included trials:** 6 RCTs, including 2 RCTs on osteopathic manipulative treatment  **Study quality:** 2 osteopathic RCTs rated ‘moderate risk of bias’  **Study characteristics:** standardised osteopathic manipulative treatment protocols versus sham (light touch) treatment (twice a day 10 to 15 mins); 21 to 58 patients, mean age 77 to 82 years  **Excluded studies eligible for current review:** no  **Further information available on:** duration of leukocytosis, leukocyte count | **RESULTS**   - No significant effect of osteopathic treatment on: mortality, cure rate, duration of fever, rate of improvement of chest X-ray, duration of oral antibiotic therapy - Hospital stay in the osteopathy group was significantly reduced by 2 days (p=0.006) compared to control - Both duration of total antibiotic therapy and intravenous therapy were reduced by about 2 days in the osteopathy versus control groups (p=0.001 and 0.0009)   **CONCLUSIONS**  Osteopathic manipulative therapy may reduce the mean duration of hospital stay and antibiotic treatment but the authors suggest that further high quality evidence is needed before chest physiotherapy can be recommended as an adjunct to conventional therapy in pneumonia in adults |
| **Pregnancy / obstetric care / neonatal care** |  |  |  |
| Khorsan 2009  **Focus:** effectiveness/safety of spinal manipulation therapy (SMT) in pregnancy-related conditions  **Quality of systematic review:** medium | **INCLUSION CRITERIA**  **Study design:** systematic reviews, randomised, non-randomised controlled trials, cohort controlled studies, case-control studies, case series, case reports  **Participants:** pregnant women with back pain and other pregnancy-related symptoms  **Interventions**: manipulative procedures (chiropractic, osteopathy)  **Outcomes:** back pain relief, pregnancy-related outcomes  **METHODOLOGY**  7 relevant databases searched; no language limit; hand search of reference lists; some details on study selection; quality assessment of studies presented; studies not presenting original data, abstracts, conference proceedings, outcomes of interest not reported, those reporting non-manual or only soft tissue treatments were excluded; excluded studies not listed.  **Data analysis:** text and tables  **Subgroups / sensitivity analyses:** not reported | **N included studies:** 1 randomised trial, 2 systematic reviews, 1 cohort study, 2 case-control studies, 6 case reports, 6 case series  **Study quality:** Scottish Intercollegiate Guidelines Network (SIGN) checklist; 13 studies were assessed for quality using SIGN: low (n=4), neutral (n=7), and high (n=2)  **Study characteristics:** studies differed in inclusion criteria, treatment protocols, and definition of outcomes. Most studies reported pain relief. Others reported pain medication use, length of labour and mode of delivery  **Excluded studies eligible for current review:** not reported | **RESULTS**  Limited evidence supported that use of SMT during pregnancy was associated with reduced back pain. Evidence regarding treatment during labour and delivery and regarding adverse events was insufficient  **CONCLUSIONS**  Since there is limited number of effective treatments for pregnancy-related back pain, clinicians may consider SMT as a treatment option, if no contraindications are present |
| **Adverse events** |  |  |  |
| Carlesso 2010  **Focus:** To explore, assess, and synthesise the risk of adverse events associated with cervical manual therapies (manipulation, mobilisation) in adults with neck pain  **Quality of systematic review:** medium | **INCLUSION CRITERIA**  **Study design:** randomised trials, non-randomised trials, cohort studies, and cross sectional surveys  **Participants:** adults with neck pain/disorders with radicular findings or cervicogenic headache receiving manual therapies  **Interventions**: manual interventions including cervical manipulation (high velocity low amplitude force applied to the cervical vertebrae) and mobilisation (low velocity manual force applied with varying amplitude to the cervical vertebrae or soft tissue techniques)  **Outcomes:** any adverse events following manual treatment  **METHODOLOGY**  5 relevant databases and 3 trial registries searched from 1998 to 2009 without language restriction; hand search of reference lists for grey literature; details on study selection; quality assessment of studies presented; excluded studies and reasons for exclusions are listed; evidence was graded for strength (high, moderate, low, very low)  **Data analysis:** text and tables  **Subgroups / sensitivity analyses:** not reported | **N included studies:** 14 RCTs and three cohort studies  **Study quality:** the Cochrane tool (RCTs), a modified Critical Appraisal Skills Program (CASP) form (cohort studies), and the McHarm scale (adverse events)  **Study characteristics:** Chronic neck pain (5 studies), acute/subacute neck pain (1 study), subacute and chronic neck pain (4 studies), mixed duration neck pain (5 studies), duration not specified (2 studies); cervicogenic headache (3 studies), mechanical neck pain (6 studies), non-specific neck pain (6 studies); RCTs had moderate to high risk for harms quality  **Excluded studies eligible for current review:** not reported | **RESULTS**  Manipulation versus control  Transient neurological symptoms  RR=1.96, 95% CI: 1.09, 3.54  Neck pain  RR=1.25, 95% CI: 0.84, 1.87  **CONCLUSIONS**  The authors were unable to draw definitive conclusions regarding the occurrence of adverse events after manipulation due to the paucity, bias, and low quality of reported evidence |
| Carnes 2009  Carnes 2010  **Focus:** To explore and provide prevalence, incidence, and risk of adverse events associated with manual therapies; provide definitions and characterise the nature of adverse events  **Quality of systematic review:** high | **INCLUSION CRITERIA**  **Study design:** systematic reviews, randomised/non-randomised trials, cohort studies, case-control studies, and case series  **Participants:** children and adults receiving manual therapies  **Interventions**: manual interventions that involve physical contact excluding any mechanical devices including manipulation (high velocity and small/large amplitude), mobilisation (low grade velocity and small/large amplitude, neuromuscular/cranio-sacral techniques), and massage  **Outcomes:** adverse events  **METHODOLOGY**  12 relevant databases searched from inception to 2008; hand search of reference lists; details on study selection; quality assessment of studies presented; excluded studies and reasons for exclusions are listed  **Data analysis:** text and tables  **Subgroups / sensitivity analyses:** not reported | **N included studies:** 17 reviews (systematic, non-systematic), 31 RCTs, 9 cohort studies (prospective), and 34 other study designs (surveys, retrospective, cross-sectional, and case series)  **Study quality:** a modified Critical Appraisal Skills Programme (CASP) for non-randomised studies; Koes’s criteria (1995) for quality appraising of randomised trials; specific adverse event quality criteria was also used  **Study characteristics:** included studies reporting adverse events ranged in quality and design and represented surveys, case notes, observational studies (cross-sectional, retrospective, and prospective cohort). The quality score of randomised trials ranged from 58-70. About half of the studies were conducted by chiropractors; 13 studies were done by neurologists and medics, 8 studies by physiotherapists, and 3 studies by osteopaths; studies were conducted in Europe (n=18),UK (n=6), USA/Canada (n=15), and Australia/New Zealand (n=4). Most studies focused on spinal manipulation.  **Excluded studies eligible for current review:** not reported | **RESULTS**  No deaths, cerebrovascular accidents or stroke were reported in any randomised study or prospective cohort study  RCTs   - Mild to moderate adverse events in manual therapy versus general practitioner care (pooled RR=1.91, 95% CI: 1.39, 2.64) - Manual therapy versus exercise (pooled RR=1.04, 95% CI: 0.83, 1.31) - Manual therapy versus placebo (pooled RR=1.84, 95% CI: 0.93, 3.62) - Manual therapy versus drug therapy (pooled RR=0.05, 95% CI: 0.0, 0.20)   Cohort studies   - The incidence of major adverse events: 0.007%. - The pooled incidence of mild to moderate adverse events 41.00% (95% CI: 17.00, 68.00). - The annual risk of stroke associated with cervical manipulation was estimated to be around 1 per 50,000 to 100,000 patients   **CONCLUSIONS**  The risk of major events (e.g., death, vascular event) in individuals receiving manual therapy is very low, lower than from taking medication; about half of the subjects receiving manual therapy experience mild to moderate adverse events 24-72 hours after intervention; the risk of events with manual therapy is lower than that with drug therapy but higher than usual care |
| Gouveia 2009  **Focus:** To explore, assess, and synthesise the risk of adverse events associated with chiropractic techniques (manipulation)  **Quality of systematic review:** medium | **INCLUSION CRITERIA**  **Study design:** randomised trials, cohort studies, case-control studies, case reports, and surveys  **Participants:** patients who received chiropractic spinal manipulation  **Interventions**: chiropractic spinal manipulation  **Outcomes:** any adverse events following chiropractic spinal manipulation  **METHODOLOGY**  2 relevant databases searched from 1966 to 2007 without language restriction; hand search of reference lists details on study selection; quality assessment not presented; excluded studies and reasons for exclusions not listed  **Data analysis:** text and tables  **Subgroups / sensitivity analyses:** not reported | **N included studies:** 1 RCT, 6 cohort studies, 12 surveys  **Study quality:** not presented  **Study characteristics:** randomised study conducted in USA; cohort studies of spinal manipulative therapy conducted in New Zealand, Norway, Sweden, UK, Belgium; surveys conducted in Germany, Sweden, South Africa, Australia, USA, Denmark, Ireland, UK, and France  **Excluded studies eligible for current review:** not reported | **RESULTS**  Frequency of adverse events (benign and transitory)  33% to 61%   - Frequency of stroke: 5 per 100,000 manipulations - Frequency of serious adverse events: 1.46 per 10,000,000 per manipulations - Frequency of death:2.68 per 10,000,000 per manipulations   RCT (manipulation versus mobilisation)  Any adverse events  OR=1.44, 95% CI: 0.85, 2.43  Case-control studies  Vertebral artery dissections within 30 days  OR=6.62, 95% CI: 1.4, 30.0  Pain before stroke  OR=3.76, 95% CI: 1.3, 11.0  **CONCLUSIONS**  Chiropractic techniques are associated with common occurrence of benign and transitory adverse events; serious adverse events such as stroke are rare as reported in prospective observational studies; the authors were unable to draw definitive conclusions regarding the occurrence of adverse events after manipulation due to the paucity, bias, and low quality of reported evidence |
| Haldeman 1999  **Focus:** To explore and review types of manipulation techniques associated with vertebrobasilar artery dissection  **Quality of systematic review:** low | **INCLUSION CRITERIA**  **Study design:** case reports  **Participants:** patients with vertebrobasilar artery dissection  **Interventions**: spinal manipulation  **Outcomes:** any adverse events following chiropractic spinal manipulation  **METHODOLOGY**  3 relevant databases (MEDLINE, Chirolars, and Chiropractic Research Abstracts Collection) searched from 1966 to 1993; search was restricted to English publications; hand search of reference lists details on study selection; quality assessment not presented; reasons for exclusions were listed  **Data analysis:** text and tables  **Subgroups / sensitivity analyses:** not reported | **N included studies:** 367 case reports  **Study quality:** not presented  **Study characteristics:** 160 case reports (spontaneously occurring), 115 case reports (after manipulation), and 95 case reports (trivial and major trauma)  **Excluded studies eligible for current review:** not reported | **RESULTS**   - Of the 367 cases, 115 (31%) had occurred after the administration of cervical manipulation, 160 (43%) had occurred spontaneously, and 26% after trauma - Only 45 (40%) of the 115 reports of cases associated with manipulation, provided some information on type of procedures used during cervical manipulation, most of which was associated with rotation (26 cases) and twisting movements (5 cases). The remaining 14 cases were associated with traction, passive mobilisation, thrust with traction, violent jerking, stretch-twist, and flexion-extension procedures   **CONCLUSIONS**  The paucity of information due to underreporting and inconsistent occurrence of specific types of manipulation techniques prevented the authors from ascertaining what type of manipulation or procedure is most likely to cause vertebrobasilar artery dissection |
| Miley 2008  **Focus:** To systematically review and explore relevant evidence if cervical manipulation causes vertebral artery dissection (VAD) and associated stroke  **Quality of systematic review:** low | **INCLUSION CRITERIA**  **Study design:** randomised trials, cohort studies, case-control studies, case reports, and surveys  **Participants:** patients who received cervical manipulation, patients with VAD/stroke  **Interventions**: cervical manipulation  **Outcomes:** VAD/stroke  **METHODOLOGY**  3 relevant databases (MEDLINE, Embase, CINAHL) searched from 1950 to 2007; evidence was assessed using the Bradford Hill’s 7 criteria for causation (dose response, large effect, consistency, biologic plausibility, reversibility, specificity, and temporal sequence); strength of evidence graded (weak, moderate, strong); study quality assessment not presented; excluded studies and reasons for exclusions not listed  **Data analysis:** text and tables  **Subgroups / sensitivity analyses:** not reported | **N included studies:** 1 systematic review, 8 cohort studies, 3 case-control study, 4 case, 1 survey  **Study quality:** not presented  **Study characteristics:** not reported  **Excluded studies eligible for current review:** not reported | **RESULTS**   - Five of the seven criteria for causation (dose response, large effect, consistency, biologic plausibility, and temporal sequence) were met and supported weak to moderate strength of evidence suggesting a causal association between cervical manipulative therapy and VAD with associated stroke - In a large case-control study, in younger patients (< 45 years), visits to chiropractors were associated with a higher risk of VAD/stroke (OR=5.03, 95% CI: 1.32, 43.87). The association was not significant in patients 45 years or older - VAD/stroke incidence estimate attributable to cervical manipulation: 1.3 cases per 100,000 persons   **CONCLUSIONS**  The authors conclude that the weak to moderate strength evidence suggests causal association between the use of cervical manipulative therapy and VAD/stroke |
| Stevinson 2002  **Focus:** To systematically review evidence on adverse events associated with spinal manipulation  **Quality of systematic review:** low | **INCLUSION CRITERIA**  **Study design:** systematic reviews, cohort studies, case-control studies, case reports, and surveys  **Participants:** patients who received spinal manipulation, patients with adverse events spinal after manipulation  **Interventions**: spinal manipulation  **Outcomes:** Any adverse event  **METHODOLOGY**  3 relevant databases (MEDLINE, Embase, Cochrane library) searched up to 2001; no language restrictions were applied; experts were contacted; reference lists of potentially relevant reports were scanned; study quality assessment not presented; excluded studies and reasons for exclusions not listed  **Data analysis:** text and tables  **Subgroups / sensitivity analyses:** not reported | **N included studies:** 3 systematic reviews, 4 cohort studies, 1 case-control study, 5 case series, 17 case reports, 3 surveys  **Study quality:** not presented  **Study characteristics:** not reported  **Excluded studies eligible for current review:** not reported | **RESULTS**   - Minor transient adverse events occurred in about half of the patients receiving spinal manipulation; the most common events were local discomfort, headache, tiredness, and dizziness - The incidence of serious adverse events based on case series and case reports ranges from 1 event per 1,000,000-2,000,000 participants to 1 event per 400,000 participants. The most common serious adverse events were vertebrobasilar accidents, disc herniation, and cauda equine syndrome - In a large case-control study, in younger patients (< 45 years), visits to chiropractors were associated with a higher risk of VAD/stroke (OR=5.03, 95% CI: 1.32, 43.87). The association was not significant in patients 45 years or older   **CONCLUSIONS**  Although mild-moderate transient adverse events are common after spinal manipulation, serious adverse events are very rare |
| Stuber 2012  **Focus:** To systematically review evidence on adverse events associated with spinal manipulation in women during pregnancy or postpartum periods  **Quality of systematic review:** medium | **INCLUSION CRITERIA**  **Study design:** Systematic reviews, randomised trials, cohort studies, case-control studies, case series, case reports, and surveys  **Participants:** women during pregnancy or postpartum periods after spinal manipulation with or without adverse event  **Interventions**: spinal manipulation (high velocity low amplitude)  **Outcomes:** Any adverse event  **METHODOLOGY**  3 relevant databases (MEDLINE, CINAHL, Index to Chiropractic Literature) searched up to 2011; no language restrictions were applied; reference lists of potentially relevant reports were scanned; English- and French-language peer reviewed publications were eligible; study quality assessed using the Scottish Intercollegiate Guidelines Network (SIGN) tools; excluded studies not listed; reasons for exclusions listed (conference proceedings, cross-sectional, descriptive studies, and narrative reviews)  **Data analysis:** text and tables  **Subgroups / sensitivity analyses:** not reported | **N included studies:** 2 systematic reviews, 1 cohort study, 4 case reports  **Study quality:** the overall SIGN rating for systematic reviews: “++” (good quality); the overall SIGN rating for the cohort study: “+” (acceptable)  **Study characteristics:** The majority of study participants had neck, headache, and/or low back pain. In case reports, women’s age ranged from 23 to 38 years. Publication year range: 1978-2009  **Excluded studies eligible for current review:** not reported | **RESULTS**  **Systematic reviews** (Stuber 2008, Khorsan 2009)   - Absence of adverse events (Stuber 2008) - One case report with adverse event (Khorsan 2009)   **Cohort study** (Murphy 2009)   - Three women (3/78; 3.8%) experienced increased pain   **Case reports**   - Memory loss, poor coordination of the right hand, difficulty with articulation, and unsteady gait (Ng 2001) - Vertigo, total occlusion of the left vertebral artery (Parkin 1978) - Swelling/neck pain, type II odontoid fracture with ventral displacement producing spinal cord compression, paravertebral haematoma, a tumour in the C2 vertebral body (Schmitz 2005) - Lower extremity numbness/neck pain, right sided epidural haematoma (Heiner 2009)   **CONCLUSIONS**  There is paucity of data on adverse events after spinal manipulation in women during pregnancy or postpartum periods. This could be explained by the rarity of such events |

**Table B. Primary studies (RCTs and non-RCTs) – study characteristics and results**

| **Study and Participants** | **Interventions** | **Outcomes** |
| --- | --- | --- |
| **Sciatica and back-related leg pain** |  |  |
| McMorland 2010  Canada  **Focus**: RCT to compare the effectiveness of spinal manipulation and surgical treatment on quality of life, disability, and pain intensity in patients with sciatica  **Duration**: 12 weeks (spinal manipulation)  **Follow**-**up**: 52 weeks  **Quality**: medium  **PARTICIPANTS**:  **N**: 40 (40% female)  **Age**: 36.4-42.85 years (range of means)  **Inclusion**: patients aged 18 years or older with leg-dominant symptoms with objective signs of nerve root tethering with or without neurologic deficit correlated with evidence of appropriate root compression on magnetic resonance imaging; must have failed to respond to at least 3 months of non-operative management (analgesics, lifestyle modification, physiotherapy, massage, and/or acupuncture; patients receiving concurrent or previous spinal manipulation  **Exclusions**: substance abuse, neurological deficits (cauda equine, foot drop), radicular symptoms < 3 months, systemic or visceral disease, haemorrhagic disorders, osteopenia, osteoporosis, pregnancy, dementia, unable to speak/read English | **Intervention type:** chiropractic  **Intervention (n=20):** chiropractic spinal manipulation (high velocity, low-amplitude, short lever technique)  **Comparison (n=20):** surgical microdiskectomy  **Dose:**  Chiropractic manipulation  2-3 visits per week (weeks 1-4), 1-2 visits per week (weeks 4-8), number of visits was based on patients’ symptoms (weeks 8-12)  Surgical microdiskectomy  Single procedure  **Providers:** a doctor of chiropractic | **Results**  Follow-up of 12 weeks post-baseline   \| **Change in outcome** \| **Spinal manipulation** \| **Surgery** \| **p-value** \| \| --- \| --- \| --- \| --- \| \| Improvement rate (n/N) \| 12/20 (60%) \| 17/20 (85%) \| NS (p=NR) \| \| Pain intensity (McGill Pain Questionnaire)  Mean (SD) \| 19.4 (14.3) \| 13.0 (16.3) \| NS (p=0.754) \| \| Pain intensity (Aberdeen Back Pain Scale)  Mean (SD) \| 35.6 (18.9) \| 25.8 (23.7) \| NS (p=0.836) \| \| Disability (Roland-Morris Disability Index)  Mean (SD) \| 9.0 (6.2) \| 7.2 (6.9) \| NS (p=0.760) \| \| Quality of life (total SF-36 score)  Mean (SD) \| 484.6 (148.9) \| 500.3 (179.7) \| NS (p=0.683) \|   ***Specific adverse effects:*** most common minor adverse events in both groups were post-procedural episodes of self-limiting increased soreness |
| Paatelma 2008  Finland  **Focus:** RCT to evaluate the effectiveness of orthopaedic manual therapy and McKenzie method compared to advice only with respect to pain intensity and disability in patients with non-specific low back pain (with/without sciatica in one or both legs)  **Duration:** 3 months  **Follow-up:** 3, 6, and 12 months  **Quality:** high  **PARTICIPANTS:**  **N:** 134 (35% female)  **Age:** 44 years  **Inclusion:** employed adults 18-65 years with acute or chronic non-specific low back pain (with/without sciatica in one or both legs)  **Exclusion**: pregnancy, low back surgery less than 2 months previously, serious spinal pathology | **Intervention type:** physiotherapy  **Intervention (n=45**): orthopaedic manual therapy (mobilisation, high velocity low-force manipulation, translatoric thrust manipulation of the thoracic-lumbar junction)  **Intervention (n=52):** McKenzie method (education, the book *Treat Your Own Back*, instructions in exercises repeated several times a day)  **Comparison (n=37):** advice only (counselling from a physiotherapist regarding the good prognosis for low back pain, pain tolerance, medication, early return to work; advice to avoid bed rest and be as active as possible through exercise activities)  **Dose:** orthopaedic manual therapy (3-7 visits each 30-45 minutes for 3 months); McKenzie method (3-7 visits each 30-45 minutes for 3 months); advice only (1 visit of 45-60 minutes for 3 months)  **Providers:** physical therapists with certification in the method used in the study; orthopaedic manual therapy was provided by a specialist with 20 years of experience in the field; McKenzie method was provided by a physiotherapist with 10 years of experience in the method; advice only programme was provided by a physiotherapist with 5 years of clinical experience in treating low back pain | **Results**  The mean improvements for the manipulation group in pain and disability were not significantly different from those observed for the McKenzie method (data not reported) and the advice only groups. No numerical data was given for the comparison of orthopaedic manual therapy versus McKenzie method   \| **Change in outcome**  (12 months post-baseline) \| **Orthopaedic manual therapy** \| **p-value**  **(orthopaedic manual therapy**  **versus advice only group)** \| \| --- \| --- \| --- \| \| Leg pain (VAS)  Mean difference  (95% CI) \| -10 (-25, 5) \| NS  (p=0.273) \| \| Low back pain (VAS)  Mean difference  (95% CI) \| -4 (-17, 9) \| NS  (p=0.714) \| \| Roland-Morris Disability Index  Mean difference  (95% CI) \| -3 (-6, 0) \| NS (p=0.068) \|   ***Specific adverse effects:*** not reported |
| **Neck pain (manipulation / mobilisation only)** |  |  |
| Aquino 2009  Brazil  **Focus**: RCT compared the effects of joint mobilisation applied to either symptomatic or asymptomatic cervical levels in patients with chronic non-specific neck pain  **Duration**: not reported  **Follow**-**up**: not reported  **Quality**: medium  **PARTICIPANTS:**  **N:** 48 (73% female)  **Age**: 33 years  **Inclusion**: adults 18-65 years with chronic neck pain (3 months or longer)  **Exclusions**: vertebral artery insufficiency, osteoporosis, tumour, infection, fracture, trauma, cervical spine surgery in the last 12 months, pregnancy, neurological deficit, treatment with physiotherapy | **Intervention type:** physiotherapy  **Intervention 1 (n=24):** mobilisation according to Maitland technique (postero-anterior central vertebral pressure, postero-anterior unilateral vertebral pressure, and transversal vertebral pressure) applied to a randomly chosen cervical vertebral level  **Intervention 2 (n=24):** mobilisation according to Maitland technique (postero-anterior central vertebral pressure, postero-anterior unilateral vertebral pressure, and transversal vertebral pressure) applied to the most symptomatic vertebral level  **Dose:** 1 session  **Providers:** well-trained physiotherapist | **Results**  Immediately after the end of treatment, significant within-group mean improvements from baseline (p<0.001) for pain scores during most painful moment and during vertebral palpation, but not for pain at resting position (experimental group: 0.54 and control group: 0); none of the differences between the two groups for any of the outcome measures was significant   \| **Change in outcome**  (Immediately post-treatment after baseline) \| **Cervical mobilisation applied to randomly chosen cervical vertebral level** \| **Cervical mobilisation applied to the most symptomatic vertebral level** \| **Mean difference**  **95% CI**  **p-value** \| \| --- \| --- \| --- \| --- \| \| Pain at rest (11-point scale)  Mean (SD) \| 0.54 (2.48) \| 0 (2.57) \| -0.52  (-1.87, 0.83)  NS \| \| Pain during most painful moment (11-point scale) Mean (SD) \| 2.67 (3.14) \| 2.62 (2.34) \| -0.13  (-1.63, 1.38)  NS \| \| Pain during vertebral palpation (11-point scale) Mean (SD) \| 2.42 (2.20) \| 2.37 (1.84) \| -0.16  (-1.31, 0.99)  NS \|   ***Specific adverse effects:*** not reported |
| Gemmell 2010  UK  **Focus**: RCT attempted to determine the relative effectiveness and harms of cervical manipulation, mobilisation, and the activator instrument in patients with subacute non-specific neck pain  **Duration**: 3 weeks  **Follow**-**up**: 12 months post-treatment  **Quality**: medium  **PARTICIPANTS:**  **N:** 47 (69%-87% female)  **Age**: 45 years  **Inclusion**: adults18-64 years with subacute non-specific neck pain present for 4 weeks or longer but no longer than 12 weeks; baseline pain intensity at least 4 points on 11-point Numerical Rating Scale  **Exclusions**: treatment with any of the study therapy, tumour, infection, fracture, trauma, radiculopathy, inflammatory arthropathy, blood coagulation disorders, long-term use of corticosteroids, cervical spine surgery, stroke or transient ischaemic attack | **Intervention type:** chiropractic  **Intervention 1 (n=16):** cervical manipulation (cervical/upper thoracic segmental high velocity, low amplitude movements)  **Intervention 2 (n=15):** cervical mobilisation (cervical/upper thoracic segmental low velocity, low amplitude movements)  **Intervention 3 (n=16):** activator instrument (high velocity, low amplitude force in the physiological range of the joint applied to cervical/upper thoracic segments)  **Dose:** 2 treatments per week for 3 weeks treated until symptom free or received maximum of 6 treatments; single session 10-15 minutes of duration  **Providers:** 2 chiropractic clinicians with 15-30 years of experience | **Results**  12 months post-treatment   - The proportion of patients who improved on PGIC was not significantly different across the manipulation, mobilisation, and activator instrument groups (73% versus 77% versus 50%) - None of the between-group differences for disability (the neck Bournemouth Questionnaire), pain intensity (NRS), or quality of life (SF-36) were statistically significant  \| **Change in outcome** \| **Activator**  **versus manipulation** \| **Activator**  **versus mobilisation** \| **Manipulation versus mobilisation** \| \| --- \| --- \| --- \| --- \| \| Patient Global Impression of Change  OR 95% CI \| 3.8  0.39, 37.18 \| 3.3  0.27, 40.61 \| 1.2  0.09, 15.96 \| \| The neck Bournemouth Questionnaire  Mean (95% CI) \| 6.54  -9.03, 22.10 \| 5.68  -12.33, 23.69 \| -0.86  -17.28, 15.59 \| \| Pain intensity (11-point NRS)  Mean (95% CI) \| 1.72  -1.17, 4.62 \| 1.30  -2.05, 4.65 \| -0.42  -3.47, 2.63 \| \| SF-36 (mental component subscale)  Mean (95% CI) \| 0.42  -7.74, 8.59 \| -1.75  -11.19, 7.69 \| -21.17  -10.78, 6.44 \| \| SF-36 (physical component subscale)  Mean (95% CI) \| -4.41  -12.48, 3.66 \| -4.53  -13.87, 4.80 \| -0.12  -8.64, 8.39 \|   ***Specific adverse effects:*** Minor transient adverse events (e.g., mild headache, mild dizziness, mild arm weakness, etc.) reported by 15 participants in the manipulation group versus 4 participants in each mobilisation and activator group |
| Klein 2013  Germany  **Focus:** RCT comparing a single strain-counterstrain intervention with sham for improving range of motion in patients with neck pain  **Duration:** single treatment  **Follow-up:** immediately after the intervention  **Quality:** high  **PARTICIPANTS:**  **N:** 61 (60 to 87% female)  **Age**: 41.9 to 47.9 years  **Inclusion**: adults 18-65 years with an acute episode of non-specific neck pain and blocking of cervical joints on manual investigation; most patients with recurrent or chronic complaints  **Exclusions**: if manual therapy contra-indicated and if measurement with a magnetic device presented a risk to the patient | **Intervention type:** osteopathy  **Intervention (n=30):** strain-counterstrain treatment  **Control (n=31):** sham treatment  **Dose:** single session  **Providers:** 1 GP with additional qualification in manual therapies and osteopathy | **Results**  After the intervention   \| **Outcome** \| **Strain-counterstrain** Mean (SD) \| **Sham**  Mean (SD) \| **p** \| \| --- \| --- \| --- \| --- \| \| Mobility restriction (%) \| 33.6 (13.6) \| 32.5 (16.0) \| NS \| \| Pain intensity 0-5) \| 1.6 (1.1) \| 1.9 (1.3) \| NS \| \| Patient assessment  Much worse  Slightly worse  Unchanged  Slightly better  Much better \| 0  3%  37%  53%  7% \| 0  3%  55%  36%  7% \| NS \|   ***Specific adverse effects:*** Mild transient adverse effects in 4 patients in the intervention group and 1 patient in the sham group (pain, dizziness) |
| Leaver 2010  Australia  **Focus**: RCT compared the effectiveness of cervical manipulation versus mobilisation in patients with acute non-specific neck pain  **Duration**: 2 weeks  **Follow**-**up**: 3 months  **Quality**: high  **PARTICIPANTS:**  **N:** 182 (65% female)  **Age**: 39 years  **Inclusion**: adults 18-70 years with non-specific neck pain less than 3 months  **Exclusions**: neck pain related to trauma, serious pathology (neoplasm), whiplash injury, infection, radiculopathy, myelopathy, cervical spine surgery, neck pain less than 2 out of 10 on NRS | **Intervention type:** physiotherapy / chiropractic / osteopathy  **Intervention (n=91):** cervical manipulation (high-velocity, low-amplitude thrust technique)  **Comparison (n=91):** cervical mobilisation (low-velocity, oscillating passive movements)  **Dose:** 4 treatments over 2 weeks  **Providers:** practitioners with postgraduate qualifications in specific training of neck manipulation and mobilisation (from physiotherapy, chiropractic, osteopathy); all practitioners had at least 2 years of clinical experience in routinely using manipulation and mobilisation techniques | **Results**  3 months of follow-up  The median number of days to recovery (the first of seven consecutive days for which the patient rated the degree of interference as “not at all”) was not significantly different between the manipulation and mobilisation groups (47 days versus 43 days, respectively; hazard ratio: 0.98, 95% CI: 0.66, 1.46)   \| **Change in outcome**  (6 months post-baseline) \| **Cervical manipulation** Mean (SD) \| **Cervical mobilisation** Mean (SD) \| **Mean difference**  95% CI \| \| --- \| --- \| --- \| --- \| \| Pain Numeric Rating Scale \| 1.6 (2.0) \| 1.4 (1.7) \| 0.2,  -0.4, 0.7 [NS] \| \| Neck Disability Index (NDI) \| 5.3 (6.2) \| 5.5 (6.6) \| -0.2,  -2.1, 1.7 [NS] \| \| Patient Specific Functional Scale \| 8.6 (2.0) \| 8.6 (1.8) \| 0.0,  -0.6, 0.5 [NS] \| \| Physical health (SF-12) \| 50.2 (6.2) \| 50.6 (7.8) \| -0.4,  -2.5, 1.7 [NS] \| \| Mental health (SF-12) \| 52.2 (8.9) \| 52.7 (8.7) \| -0.5,  -3.1, 2.2 [NS] \| \| Global perceived effect* \| 3.3 (1.7) \| 3.4 (1.9) \| -0.1,  -0.6, 0.4 [NS] \|   * from ‘much worse’ (-5) to ‘completely recovered’ (+5)  ***Specific adverse effects:*** Two participants in the mobilisation group had serious adverse events unrelated to the treatment (cardiac surgery and severe arm pain/weakness). Most frequent adverse events were minor: increased neck pain (28%) and headache (22%). Other less frequent events were dizziness (7%), nausea (6%), and paraesthesia (7%). The frequency of adverse events was not significantly different between the study groups. |
| Martel 2011  Canada  **Focus**: RCT investigated the efficacy of spinal manipulative therapy (SMT) compared to no treatment in patients with non-specific chronic neck pain  **Duration**: 10 months  **Follow**-**up**: 10 months  **Quality**: medium  **PARTICIPANTS:**  **N:** 98 (40%-80% female)  **Age**: 40 years  **Inclusion**: adults 18-60 years with neck pain 12 weeks or more, no current chiropractic therapy  **Exclusions**: neck pain related to trauma, serious pathology (neoplasm), whiplash injury, infection, osteoarthritis, cardiovascular disease, cervical spine surgery, pregnancy | **Intervention type:** chiropractic  **Intervention (n=33):** spinal manipulative therapy (standardised passive palpation on the cervical and thoracic spine) plus home exercise (range of motion exercise, stretching/mobilisation, strengthening exercise of the cervical/upper thoracic spine, flexion/extension, rotation)  **Intervention (n=36):** spinal manipulative therapy (standardised passive palpation on the cervical and thoracic spine)  **Comparison (n=29):** no treatment (attention group; clinical visits, distribution of diaries)  **Dose:** spinal manipulative therapy (maximum of 4 treatments per session given once a month which lasted 10-15 minutes); home exercise (3 sessions of 20-30 minutes per week)  **Providers:** chiropractors with at least 3 years of experience | **Results**  10 months of follow-up  After the treatment phase, all study groups experienced significant improvements in disability and lateral flexion; however, the between-group differences for all outcome measures were statistically non-significant   \| **Outcome** \| **SMT + home exercise** Mean (SD) \| **SMT**  Mean (SD) \| **No treatment**  Mean (SD) \| \| --- \| --- \| --- \| --- \| \| Pain (VAS score) \| 1.6 (2.3) \| 2.1 (2.3) \| 2.9 (2.9) \| \| Neck Disability Index (NDI) \| 11.3 (11.8) \| 13.7 (12.1) \| 21.5 (14.0) \| \| Flexion-extension (degrees) \| 115.6 (22.5) \| 114.1 (21.0) \| 106.1 (23.3) \| \| Rotation (degrees) \| 126.7 (25.7) \| 126.9 (29.5) \| 119.5 (15.4) \| \| Lateral flexion (degrees) \| 70.8 (23.7) \| 67.1 (13.6) \| 70.5 (11.1) \| \| Physical health (SF-12) \| 54.1 (7.2) \| 53.1 (6.9) \| 52.1 (8.2) \| \| Mental health (SF-12) \| 49.8 (8.7) \| 52.3 (8.4) \| 49.9 (10.1) \|   ***Specific adverse effects:*** no serious adverse events |
| Puentedura 2011  USA  **Focus**: RCT compared the effectiveness of thoracic TJM plus cervical ROM exercise versus cervical TJM in adults with acute neck pain  **Duration**: 2 weeks  **Follow**-**up**: 6 months  **Quality**: medium  **PARTICIPANTS:**  **N:** 24 (67% female)  **Age**: 33 years  **Inclusion**: adults18-60 years with acute neck pain with NDI score of 10/50 or greater; participant had to meet at least 4 of the 6 criteria (symptom duration < 30 days, no symptom distal to the shoulder, no aggravation of symptoms by looking up, FABQ physical activity subscale < 12, decreased thoracic spine kyphosis T3-T5, cervical ROM<30°)  **Exclusions**: serious pathology (neoplasm), cervical stenosis, nerve root compression, whiplash injury within 6 weeks prior to study, cervical spine surgery, rheumatoid arthritis, osteoporosis, osteopenia, or ankylosing spondylitis | **Intervention type:** physiotherapy  **Intervention 1 (n=10):** thoracic thrust joint manipulation (high velocity, midrange/end range, distraction or anterior-posterior force applied to the mid/upper thoracic spine on the lower/mid thoracic spine in a sitting position) plus cervical ROM exercise (3-finger cervical rotation) followed by standardised exercise programme (3-finger cervical rotation, bilateral shoulder shrugs / adductions / abductions, scapular retractions, upper/lower cervical flexion and extension, Thera-Band rows, and lateral pull downs)  **Intervention 2 (n=14):** cervical TJM plus cervical ROM exercise followed by standardised exercise programme  **Dose:** 5 sessions over 2 weeks; thoracic TJM plus cervical ROM (2 sessions), cervical TJM plus cervical ROM exercise (2 sessions), standardised exercise programme (3 sessions)  **Providers:** physical therapists | **Results**   \| **Change in outcome**  (6 months post-baseline) \| **Cervical thrust joint manipulation** \| **Thoracic thrust joint manipulation** \| **p-value** \| \| --- \| --- \| --- \| --- \| \| Neck Disability Index (NDI) score \| 3.7 (SD 5.7) \| 9.9 (SD 3.9) \| p=0.004 \| \| Numeric Pain Rating Scale (NPRS) \| 0.1 (SD 0.1) \| 2.3 (SD 1.1) \| p<0.001 \| \| Fear-Avoidance Beliefs Questionnaire (FABQ) \| 2.1 (SD 3.5) \| 5.2 (SD 3) \| p=0.04 \| \| Success rate (met or exceeded pre-specified minimal clinically important difference for NDI, NPRS, and global rating of change scales)* \| 10/14 (71.4%) \| 10%  (1/10) \| p=NR \|   * Minimal clinically important difference: NDI (7 points), NPRS (1.3 points), and global rating of change (at least +5)  ***Specific adverse effects:*** Minor transient adverse events (increased neck pain, fatigue, headache, upper back pain) reported by 70%-80% of the participants in the thoracic TJM group versus 7% in the cervical TJM |
| Schomacher 2009  Germany  **Focus:** RCT to compare the effects of analgesic mobilisation applied either to symptomatic or asymptomatic segments of the cervical spine in adults with chronic neck pain  **Duration:** 4 minutes  **Follow-up:** immediate post-treatment  **Quality:** low  **PARTICIPANTS:**  **N:** 126 (NR female)  **Age:** 49 years  **Inclusion:** adults >17 years with chronic neck pain (no diagnosis necessary), able to sit and lie down, demonstrate active/passive movements  **Exclusion**: conditions in which active and passive movements could harm the patient, nerve root compression, and acute inflammation | **Intervention type:** physiotherapy  **Intervention (n=59**): mobilisation technique (intermittent translatoric traction at the zygopophyseal joint between C2 and C7 with Kaltenborn’s grade II force) applied to symptomatic levels of the cervical spine (concordant segment)  **Comparison (n=67):** mobilisation technique applied to asymptomatic levels of the cervical spine (3 levels below/above concordant segment)  **Dose:** a single 4-minute mobilisation technique  **Providers:** a physiotherapist-researcher with training in musculoskeletal treatment and orthopaedic manual therapy; 20 years of experience | **Results**  Both treatment groups improved significantly (p<0.01) in terms of pain and sensation after treatment versus before treatment. The between–group post-treatment differences were not statistically significant   \| **Change in outcome**  (Immediate after treatment) \| **Manual therapy (localised segment)** \| **Manual therapy**  **(3 levels below/above localised segment)** \| **p-value** \| \| --- \| --- \| --- \| --- \| \| Neck pain intensity (NRS) endpoint mean scores \| 1.8 (SD 1.4) \| 2.0 (SD 1.6) \| NS (p=NR) \| \| Sensation of movement (NRS) endpoint mean scores \| 2.0 (SD 1.3) \| 2.1 (SD 1.7) \| NS (p=NR) \| \| Neck pain intensity (NRS) mean change score \| 1.3 (SD 1.2) \| 1.7 (SD 1.5) \| NS (p=0.12) \| \| Sensation of movement (NRS) mean change score \| 1.9 (SD 1.4) \| 2.2 (SD 1.6) \| NS (p=0.15) \|   ***Specific adverse effects:*** not reported |
| **Ankle and foot conditions** |  |  |
| Kuhar 2007  India  **Focus:** RCT of the effects of myofascial release in the treatment of plantar fasciitis  **Duration:** 10 days  **Follow-up:** no post-intervention follow-up  **Quality:** low  **PARTICIPANTS:**  **N:** 30 (55% female)  **Age:** 43 SD10 years  **Inclusion:** clinically diagnosed with plantar fasciitis ≥6 weeks, heel pain felt maximally over plantar aspect of heel, pain in the heel on the first step in the morning, no history of heel pain at rest | **Intervention type:** physiotherapy  **Intervention (n=15):** conventional therapy (ultrasound, contrast bath, towel curl, active ankle exercises, Achilles tendon stretching, plantar fascia stretching with tennis ball) plus myofascial release using thumb, finger cupping and fingers technique for 15 mins  **Comparison (n=15):** conventional treatment only  **Dose:** daily treatments for 10 days  **Providers:** not reported | **Results**   \|  \| **Intervention** \| **Control** \| **p** \| \| --- \| --- \| --- \| --- \| \| **Pain (VAS)** \| 1.6 SD0.73 \| 3.67 SD1.49 \| 0.000 \| \| **Foot function index** \| 16.20 SD3.89 \| 19.80 SD4.36 \| 0.024 \|   ***Specific adverse effects:*** not reported |
| Joseph 2010  South Africa  **Focus:** RCT of the effect of muscle energy technique versus manipulation in the treatment of chronic recurrent ankle sprain  **Duration:** 3 weeks  **Follow-up:** no post-intervention follow-up  **Quality:** low  **PARTICIPANTS:**  **N:** 40 (53% female)  **Age:** 28.4 to 30.5 years  **Inclusion:** age 18 to 50 years, mild to moderate chronic recurrent ankle inversion sprain; most recent sprain at least 7 weeks before presentation; at least two of the following: 1. Ankle pain with a rating of 3 to 6 on the numerical rating scale, 2. Additional episodes of giving way, 3. Ankle stiffness | **Intervention type:** chiropractic  **Intervention 1 (n=20):** high velocity low amplitude ankle axial elongation manipulation  **Intervention 2 (n=20):** muscle energy technique (MET) to the ankle joint: 5 repetitions of ankle dorsiflexion to patient resistance with simultaneous anterior to posterior pressure against the talus; post-isometric contraction was followed with gentle increase into dorsiflexion and additional anterior to posterior pressure against the talus  **Dose:** 6 treatments over 3 weeks  **Providers:** not reported | **Results**   - One Leg Standing Test (OLST) eyes open and closed, McGill Pain Questionnaire, Functional Evaluation Scale, dorsiflexion and plantarflexion: significant improvement over time in both groups, but no significant difference between groups  \|  \| **Manipulation**  **(95% CI)** \| **MET**  **(95% CI)** \| **p** \| \| --- \| --- \| --- \| --- \| \| **Pain (NRS)** \| 37.13  (32.7, 41.6) \| 39.6  (33.0, 46.3) \| NS \| \| **OLST eyes closed (s)** \| 10.45  (13.2, 7.7) \| 10.05  (13.2, 6.9) \| NS \| \| **Dorsiflexion (°)** \| 9.75  (13.1, 6.4) \| 7.65  (9.6, 5.7) \| NS \|   ***Specific adverse effects:*** no significant or sever soreness or stiffness in the ankles reported as result of treatment, no one left the trial because of any minor or severe adverse reactions |
| du Plessis 2011  South Africa  **Focus:** RCT of the effects of manual and manipulative therapy compared to night splints for hallux abducto valgus  **Duration:** 2 weeks  **Follow-up:** 1 month  **Quality:** medium  **PARTICIPANTS:**  **N:** 30 (% female equal but not reported)  **Age:** 42 years (25 to 65)  **Inclusion:** symptomatic hallux abducto valgus, pain and reduced function of the first metatarsophalangeal joint (MTP), inability to wear shoes comfortably, age 26 to 64 years | **Intervention type:** chiropractic  **Intervention 1 (n=15):** graded joint mobilisation of the first MTP, joint manipulation, mobilisation/manipulation of other foot and ankle joints as indicated, post-treatment cold therapy  **Intervention 2 (n=15):** night splint  **Dose:** manual therapy: 4 treatments over 2 weeks  **Providers:** chiropractors | **Results**   - No significant difference between intervention and control for pain and function at the end of the intervention, but improvement maintained in the manual therapy group and not in the night splint group   At 1 month follow-up   \|  \| **Manual therapy**  **(95% CI)** \| **Night splint**  **(95% CI)** \| **p** \| \| --- \| --- \| --- \| --- \| \| Pain (VAS, %) \| 1.2  (0, 3) \| 17.7  (10, 24) \| <0.01 \| \| Foot function scores (%) \| 2.3  (0, 6) \| 32.4  (19, 45) \| <0.01 \| \| Hallux dorsiflexion (°) \| 50.8  (47, 55) \| 37.7  (33, 46) \| 0.02 \|   ***Specific adverse effects:*** 2 manual therapy patients experienced transient discomfort and/or stiffness that quickly resolved |
| Renan-Ordine 2011  Brazil  **Focus:** RCT of the effects of myofascial trigger point manual therapy combined with a stretching programme for the management of plantar heel pain  **Duration:** 1 month  **Follow-up:** no post-intervention follow-up  **Quality:** medium  **PARTICIPANTS:**  **N:** 60 (85% female)  **Age:** 44 SD10 years  **Inclusion:** age 18 to 60 years, unilateral plantar heel pain: 1. Insidious onset of sharp pain under the plantar heel surface upon weight bearing after a period of non-weight bearing, 2. Plantar heel pain that increases in the morning with the first steps after waking up, 3. Symptoms decreasing with slight level of activity, such as walking; no red flags | **Intervention type:** physiotherapy  **Intervention (n=30):** self-stretching (including calf muscles and plantar fascia specific exercises) plus soft tissue trigger point manual therapy (examined for active trigger points in the gastrocnemius muscle, trigger point pressure release plus neuromuscular technique (longitudinal stroke) over both gastrocnemius muscles )  **Comparison (n=30):** self-stretching  **Dose:** 4 treatments per week for 4 weeks  **Providers:** clinician with 5 years of postgraduate orthopaedic manual therapy training | **Results**   - SF-36 at end of intervention (0 to 100 on each subscale)  \|  \| **Intervention** \| **Control** \| **p** \| \| --- \| --- \| --- \| --- \| \| ***SF-36*** \|  \|  \|  \| \| Physical function \| 65.2 SD12.2 \| 52.8 SD19.4 \| 0.001 \| \| Physical role \| 63.5 SD27.6 \| 50.9 SD32.9 \| NS \| \| Bodily pain \| 56.1 SD13.8 \| 44.7 SD17.5 \| 0.005 \| \| General health \| 60.8 SD12.2 \| 54.9 SD16.2 \| NS \| \| Vitality \| 52.1 SD15.7 \| 44.1 SD19.0 \| NS \| \| Social function \| 68.3 SD18.8 \| 57.0 SD17.8 \| NS \| \| Emotional role \| 78.6 SD27.5 \| 51.9 SD32.5 \| NS \| \| Mental health \| 62.0 SD19.8 \| 60.1 SD22.2 \| NS \| \| ***Pressure pain thresholds*** \|  \|  \|  \| \| Gastrocnemius muscle \| 2.7 SD0.6 \| 2.3 SD0.5 \| <0.03 \| \| Soleus muscle \| 3.0 SD0.9 \| 2.4 SD0.5 \| <0.03 \| \| Calcaneus \| 3.2 SD1.3 \| 2.6 SD0.9 \| <0.03 \|   ***Specific adverse effects:*** not reported |
| **Carpal tunnel syndrome** |  |  |
| Hains 2010  Canada  **Focus:** RCT of the effects of ischaemic compression therapy for chronic carpal tunnel syndrome  **Duration:** 5 weeks  **Follow-up:** 6 months  **Quality:** medium  **PARTICIPANTS:**  **N:** 55 (62% female)  **Age:** 46 SD6.7 to 47 SD7.2 years  **Inclusion:** age 20 to 60 years, suffer from numbness in the hand affecting the thumb, the index finder, the middle finger and half the ring finger on a daily basis for at least 3 months, at least 2 of the following: Tinnel positive sign, Phallen positive sign, sleep problems caused by hand discomfort | **Intervention type:** chiropractic  **Intervention (n=37):** participants examined for trigger points along the biceps, the bicipital aponeurosis, the pronator teres muscle, the axilla of the shoulder; during treatment, pressure was applied for 5 to 15 seconds to each of the identified trigger points; thumb tip pressure (one thumb over the other) was then applied for 5 seconds every 2 cms, along the biceps; for trigger points located in the hollow of the elbow (pronator teres, biceps aponeurosis) and in the axilla (subscapularis), the pressure was maintained for 15 seconds; trigger points were treated using a light pressure, which was gradually increased until it reached the participant’s maximum pain tolerance level  **Comparison (n=18):** control treatment: ischaemic compressions of latent or active trigger points located in the posterior region of the clavicle (supraspinatus area), on the deltoid (anterior and lateral region), and on the centre of the shoulder blade (infraspinatus area); were offered the opportunity to receive further treatment after the end of the control treatment, 13 agreed and received the experimental treatment  **Dose:** 15 treatments, 3 treatments per week  **Providers:** chiropractor | **Results**   - Standardised symptom and functional status questionnaire; perceived improvement numerical scale  \|  \| **Intervention** \| **Control** \| **p** \| \| --- \| --- \| --- \| --- \| \| Improvement in severity of symptoms and functional status \| *15 treatments:*  42% SD21  *6 months:*  36% SD23 \| *15 treatments:*  26% SD18  *after 15 experimental treatments:*  48% SD15 \| <0.05 (after 15 treatments) \| \| Perceived improvement numerical scale \| *15 treatments:*  67% SD26  *6 months:*  56% SD35 \| *15 treatments:*  50% SD25  *after 15 experimental treatments:*  75% SD21 \| <0.021 (after 15 treatments) \|   ***Specific adverse effects:*** not reported |
| **Lateral epicondylitis (tennis elbow)** |  |  |
| Ajimsha 2012  Malaysia  **Focus:** to investigate whether myofascial release reduces the pain and functional disability of lateral epicondylitis in comparison to sham ultrasound in computer professionals  **Duration:** 4 weeks  **Follow-up:** 12 weeks  **Quality:** low  **PARTICIPANTS:**  **N:** 68 (56% female)  **Age:** 29.3 to 30.5 years  **Inclusion:** computer professionals between 20 and 40 years with a diagnosis of lateral epicondylitis in the mouse-operating arm based on the Southampton criteria for lateral epicondylitis, pain lasting ≥1 day in the last 7 days in the lateral elbow region; history of trauma, previous surgery, systemic steroids excluded | **Intervention type:** physiotherapy  **Intervention (n=33):** myofascial release (treating from the common extensor tendon to the wrist, through the periosteum of the ulna and spreading the radius from the ulna)  **Comparison (n=32):** sham ultrasound  **Dose:** 3 times weekly for 4 weeks, 30 min treatment sessions  **Providers:** not reported | **Results**  Patient-Rated Tennis Elbow Evaluation (PRTEE) Scale   \| **Outcome**  At 12 weeks \| **Myofascial release** \| **Sham treatment** \| **p-value** \| \| --- \| --- \| --- \| --- \| \| PRTEE scale (mean (SD)) \| 23.9 (4.1) \| 65.9 (4.5) \| <0.001 \|   ***Adverse events:*** no serious adverse events, 5 patients in intervention group reported increase in pain in first week, but this subsided within a week without any medications |
| Blanchette 2011  Canada  **Focus:** RCT compared the effectiveness of chiropractic mobilisation and no treatment in adults with LE  **Duration:** 5 weeks  **Follow-up:** 3 months  **Quality:** low  **PARTICIPANTS:**  **N:** 30  **Age:** 46 years  **Inclusion:** adults 18 years or older with diagnosis of LE (Cozen, Mill tests) | **Intervention type:** chiropractic  **Intervention (n=15):** chiropractic mobilisation (augmented soft tissue technique)  **Comparison (n=15):** no treatment“ (information on natural history of LE and advice about ergonomic, stretching exercises of the flexors, the wrist extensor muscles, analgesics)  **Dose:** chiropractic mobilisation (2 treatments for 5 weeks); no treatment/advice (1 face-to-face session)  **Providers:** chiropractor with Master’s degree in kinesiology | **Results**  At both follow-ups, the groups demonstrated significant improvements in PRTEE, VAS, and pain-free grip, when compared to baseline. However, no between-group difference for these measures was statistically significant   \| **Change in outcome**  At 3 months \| **Chiropractic mobilisation** \| **No treatment/advice** \| **p-value** \| \| --- \| --- \| --- \| --- \| \| Patient-Rated Tennis Elbow Evaluation Mean (SD) \| 16 (10) \| 17 (13) \| NS (>0.05) \| \| Pain intensity (VAS) Mean (SD) \| 17 (17) \| 21 (17) \| NS (>0.05) \| \| Pain-free grip  Mean (SD) \| 27 (13) \| 28 (14) \| NS (>0.05) \|   ***Specific adverse effects:*** 14 patients in the mobilisation group reported aches and bruises |
| Nagrale 2009  India  **Focus:** RCT compared the effectiveness of Cyriax physiotherapy and phonophoresis with supervised exercise in adults with LE  **Duration:** 4 weeks  **Follow-up:** 8 weeks  **Quality:** medium  **PARTICIPANTS:**  **N:** 60  **Age:** 38.6 years  **Inclusion:** adults 30-60 years with diagnosis of LE >1 month | **Intervention type:** physiotherapy  **Intervention (n=30):** Cyriax physiotherapy (10 minutes of deep transverse friction massage followed by single application of Mill’s manipulation)  **Comparison (n=30):** phonophoresis with supervised exercise and non-steroidal anti-inflammatory gel for 5 minutes  **Dose:** 12 sessions (3 times in 4 weeks)  **Providers:** not reported | **Results**  At 4 and 8 weeks, both groups demonstrated significant improvements in all three measures when compared to baseline. The Cyriax physiotherapy versus phonophoresis experienced significantly greater mean improvements:  Pain (VAS score) at 8 weeks  5.03 (95% CI 4.62, 5.44) versus 2.50 (95% CI 2.122, 2.87)  Pain-free grip strength (in kg) at 8 weeks  25.46 (95% CI 23.13, 27.80) versus 10.93 (95% CI 9.38, 12.48)  Functional status (TEFS score) at 8 weeks  20.93 (95% CI 19.30, 22.56) versus 11.90 (95% CI 10.64, 13.15)  ***Specific adverse effects:*** not reported |
| Viswas 2012  India  **Focus:** RCT to compare the effectiveness of supervised exercise and Cyriax physiotherapy in adults with LE  **Duration:** 4 weeks  **Follow-up:** no post-intervention follow-up  **Quality:** low  **PARTICIPANTS:**  **N:** 20 (50% female)  **Age:** 37.4 to 38.2 years  **Inclusion:** adults 30-45 years with diagnosis of lateral epicondylitis, symptom duration 8-10 weeks | **Intervention type:** physiotherapy  **Intervention (n=10):** Cyriax physiotherapy (deep transverse friction massage, Mill's manipulation)  **Comparison (n=10):** therapeutic exercise (stretching, eccentric strengthening exercise); education manual  **Dose:** 3 sessions per week for 4 weeks  **Providers:** no details given | **Results**   \| **Outcome**  At 4 weeks \| **Cyriax** \| **Exercise** \| **p-value** \| \| --- \| --- \| --- \| --- \| \| Pain (VAS) \| 13.9 \| 7.10 \| 0.009 \| \| Tennis Elbow Function Scale \| 14.35 \| 6.65 \| 0.002 \|   Pain and function significantly increased in both groups compared to baseline  ***Adverse events:*** not reported |
| Amro 2010  Palestine  **Focus:** non-RCT: compared the effect of Mulligan technique plus traditional treatment versus traditional treatment alone in participants with LE  **Duration:** 4 weeks  **Follow-up:** 4 weeks  **Quality:** low  **PARTICIPANTS:**  **N:** 34  **Age:** 37 years  **Inclusion:** adults with diagnosis of subacute LE, positive results on two or more tennis elbow tests | **Intervention type:** physiotherapy  **Intervention (n=17):** Mulligan technique (mobilisation, movement and taping) plus traditional treatment (thermal treatment, massage, ultrasound, exercise)  **Comparison (n=17):** traditional treatment (thermal treatment, massage, ultrasound, exercise)  **Dose:** 3 sessions per week for 4 weeks; each session lasted 30-45 minutes  **Providers:** physiotherapists trained by the researchers | **Results**  At 4 weeks after baseline (immediately after treatment), both groups demonstrated significant improvements in all three measures when compared to baseline (p<0.001). The Mulligan technique group versus traditional treatment demonstrated significantly greater mean improvements in pain and PRTEE but not in pain-free grip strength scores:  Pain (VAS score) at 4 weeks:  5.3 (SD 0.9) versus 3.2 (SD 2.1), p<0.01  Patient-Rated Tennis Elbow Evaluation (PRTEE):  40.7 (SD 15.1) versus 27.7 (SD 21.7), p<0.05  Pain-free grip strength (in kg) at 4 weeks:  4.8 (SD 1.8) versus 1.0 (SD 1.8), p>0.05 (NS)  ***Specific adverse effects:*** not reported |
| Cleland 2004  USA  **Focus:** observational cohort study retrospectively compared the effectiveness of adding cervical spine manual therapy to local management directed at the elbow administered to adult patients with LE  **Duration:** not reported  **Follow-up:** 72-74 weeks  **Quality:** low  **PARTICIPANTS:**  **N:** 112  **Age:** 42 years  **Inclusion:** adults with diagnosis of LE, pain during palpation of LE, pain with resisted wrist/middle finger extension | **Intervention type:** physiotherapy  **Intervention (n=51):** cervical spine manual therapy (passive intervertebral mobilisation, mobilisation with movement, muscle energy techniques) plus local management directed at the elbow (pulsed ultrasound, iontophoresis, deep tissue massage, stretching, strengthening exercise for muscles of the upper extremity, cold packs, elbow joint mobilisation)  **Comparison (n=61):** local management directed at the elbow (pulsed ultrasound, iontophoresis, deep tissue massage, stretching, strengthening exercise for muscles of the upper extremity, cold packs, elbow joint mobilisation)  **Dose:** average number of visits ranging from 4 to 11.5  **Providers:** physical therapists | **Results**  The response rate: 85% (95 responders)  Self-reported outcome of success rate (i.e., return to all functional activities without recurrence of elbow symptoms after discharge from physical therapy) was numerically greater in the cervical spine manual therapy versus local management (80% versus 75%, p-value not reported)  ***Specific adverse effects:*** not reported |
| Rompe 2001  Germany  **Focus:** non-RCT: compared manual therapy plus extracorporeal low-energy shockwave therapy (ESWT) versus ESWT alone in participants with LE  **Duration:** NR  **Follow-up:** 3 and 12 months  **Quality:** low  **PARTICIPANTS:**  **N:** 60  **Age:** 47 years  **Inclusion:** adults with diagnosis of chronic LE (>6 months), pain during palpation of LE, pain with resisted wrist/middle finger extension, chair test, signs of cervical dysfunction with pain at C4-5 and/or C5-6 level with the head in a protracted position | **Intervention type:** physiotherapy  **Intervention (n=30):** manual physiotherapy (soft mobilisation of the cervical spine/cervicothoracic junction and flexion mobilisation in the cervical joints to relieve pain in C4-5 and/or C5-6 levels and correct protraction) plus extracorporeal low-energy shockwave therapy (ESWT)  **Comparison (n=30):** ESWT  **Dose:** 10 sessions of manual therapy  **Providers:** physiotherapists certified for manual therapy | **Results**  Roles and Maudsley scores after12 months  Both treatment groups experienced significant improvements compared to baseline. The difference between the two groups in Roles and Maudsley scores was not statistically significant (excellent outcome: 56% versus 60%, p>0.05)  Pain  Both treatment groups experienced significant improvements compared to baseline. The differences between the two groups in pain scores were not statistically significant   \| **Change in outcome**  At 12 months \| **Manual physiotherapy** \| Low-energy shockwave therapy \| **p-value** \| \| --- \| --- \| --- \| --- \| \| Pressure pain  Mean (SD) \| 2.27 (2.59) \| 1.97 (2.05) \| 0.82 \| \| Thomsen Test  Mean (SD) \| 1.93 (1.97) \| 2.09 (2.01) \| 0.71 \| \| Resisted finger extension  Mean (SD) \| 1.45 (1.84) \| 1.66 (1.79) \| 0.57 \| \| Chair test  Mean (SD) \| 1.91 (2.51) \| 1.97 (2.27) \| 0.76 \|   ***Specific adverse effects:*** not reported |
| **Shoulder conditions** |  |  |
| Bialoszewski 2011  Poland  **Focus:** RCT of the effects of manual therapy on range of motion and pain in patients with chronic glenohumeral rotator cuff injuries  **Duration:** unclear  **Follow-up:** unclear  **Quality:** low  **PARTICIPANTS:**  **N:** 30 (40% female)  **Age:** 51.3 years (38 to 61)  **Inclusion:** confirmed diagnosis of chronic rotator cuff injury without indications for surgical treatment | **Intervention type:** physiotherapy  **Intervention (n=15):** standard rehabilitation (TENS to the glenohumeral joint (20 min session), ultrasound to the supraspinatus insertion region (4 to 9 min session), kinesiotherapy to strengthen the glenohumeral rotator cuff (active, passive and self-assisted exercises)) plus manual therapy (kinesiotherapy of the glenohumeral joint and soft tissues using Kaltenborn’s roll-glide techniques, Cyriax deep transverse massage, Mulligan’s kinesiotherapy with movement and typical techniques of glenohumeral joint kinesiotherapy in the anteroposterior direction)  **Comparison (n=15):** standard rehabilitation only  **Dose:** at least 15 treatments  **Providers:** not reported | **Results**   - The study reports 4 examinations but it is unclear at what points in the progress of the study patients were examined - Shoulder girdle elevation through flexion, shoulder girdle elevation through abduction, external rotation, internal rotation and pain significantly more improved in the group receiving manual therapy compared to standard rehabilitation only     ***Specific adverse effects:*** not reported |
| Bron 2011  The Netherlands  **Focus:** RCT of the effects of myofascial trigger point treatment in patients with chronic shoulder pain  **Duration:** 12 weeks  **Follow-up:** no post-intervention follow-up  **Quality:** high  **PARTICIPANTS:**  **N:** 72 (61% female)  **Age:** 42.8 to 45.0 years (38.7 to 49.9)  **Inclusion:** unilateral non-traumatic shoulder pain for at least 6 months, aged between 18 and 65 years; adhesive capsulitis excluded | **Intervention type:** physiotherapy  **Intervention (n=34):** inactivation of active myofascial trigger points by manual compression, combined with other manual techniques (deep stroking or strumming), intermittent cold application; instruction to perform simple gentle static stretching and relaxation exercises at home several times a day; instructed to apply heat; ergonomic advice  **Comparison (n=31):** wait and see, started physiotherapy after the end of the trial period  **Dose:** once weekly for up to 12 weeks  **Providers:** 5 physiotherapists | **Results**   - Disabilities of Arm, Hand and Shoulder Questionnaire (DASH) (0 to 100, higher score = greater disability), minimal clinically important difference is 10 points - Pain (VAS), minimal clinically important difference is 14 mm, VAS-P1: pain at current moment, VAS-P2: average pain during last 7 days, VAS-P3: most severe pain during last 7 days - Global Perceived Effect (GPE, 1 (much worse) to 8 (completely recovered)) - PROM (passive range of motion) – no significant change   Results after 12 weeks   \|  \| **Intervention** \| **Control** \| **p** \| \| --- \| --- \| --- \| --- \| \| DASH \| 18.4 SD12.3 \| 26.1 SD13.8 \| <0.05 \| \| VAS-P1 \| 17.2 SD19.5 \| 31.0 SD21.0 \| <0.05 \| \| VAS-P2 \| 22.5 SD16.4 \| 33.2 SD23.3 \| <0.05 \| \| VAS-P3 \| 34.0 SD21.9 \| 47.8 SD27.3 \| <0.05 \| \| GPE improved \| 55% \| 14% \| <0.05 \| \| No. of muscles with active trigger points \| 4.8 SD3.0 \| 7.5 SD3.2 \| <0.05 \| \| No. of muscles with latent trigger points \| 4.7 SD2.3 \| 4.4 SD2.3 \| NS \|   ***Specific adverse effects:*** not reported |
| Fink 2012  Germany  **Focus:** RCT of the effects of therapy according to the fascial distortion model compared to conventional manual therapy in patients with frozen shoulder  **Duration:** 2 weeks  **Follow-up:** 6 weeks after the last treatment  **Quality:** medium  **PARTICIPANTS:**  **N:** 60 (63.3% female)  **Age:** 56 years  **Inclusion:** >18 years, painful reduced mobility of the shoulder, constant complaints for >4 weeks; exclusions: severe diseases of the musculoskeletal apparatus, contraindications to manual therapy, shoulder trauma | **Intervention type:** osteopathy/physiotherapy  **Intervention (n=30):** fascial distortion model (FDM) therapy: trigger point massage, treatment of trigger regions in the shoulder-arm region, therapy of cylindrical distortions  **Comparison (n=30):** classic manual therapy (passive mobilisations of the shoulder and stretching)  **Dose:** 2 sessions per week over 2 weeks, about 20 min per session  **Providers:** 2 therapists with >6 years of experience in the respective therapeutic concept | **Results**   \| **Outcome**  6 weeks after treatment \| **Fascial distortion model therapy** \| **Classic manual therapy** \| **p-value** \| \| --- \| --- \| --- \| --- \| \| Pain (mean, SD) \| 1.59 (2.05) \| 3.36 (2.48) \| <0.001 \| \| Function, Constant-Murley-Score (mean, SD) \| 77.8 (21.1) \| 56.6 (23.1) \| <0.001 \| \| Function, DASH-Score (mean, SD) \| 16.1 (16.5) \| 31.4 (18.4) \| <0.001 \| \| Strength (Nm, SD) \| 19.3 (10.1) \| 11.1 (11.2) \| <0.001 \|   ***Specific adverse events:*** most patients in the FDM therapy group (77.8%) found the treatment uncomfortable, compared to 37% in the classic manual therapy group; 14 patients in the FDM group had visible haematomas on their upper arms; none of the participants had serious adverse effects |
| Yang 2012  Taiwan  **Focus:** RCT of the effects of end-range mobilization / scapular mobilisation in patients with frozen shoulder  **Duration:** 3 months  **Follow-up:** 8 weeks  **Quality:** high  **PARTICIPANTS**:  **N:** 34 (73% female)  **Age:** 54.3 to 56.8 years  **Inclusion:** patients with frozen shoulder syndrome (at least 50% loss of passive motion of the shoulder joint), duration of complaints for at least 3 months; exclusions: history of stroke with residual upper-extremity involvement, diabetes mellitus, rheumatoid arthritis, rotator cuff tear, surgical stabilisation of the shoulder, osteoporosis, or malignancies in the shoulder region; disorders of the cervical spine, elbow, wrist, or hand | **Intervention type:** physiotherapy  **Note:** The main treatment groups included patients meeting criteria from a kinematics prediction (n=23, 8 degrees of scapular posterior tipping, 97 degrees of humeral elevation, and 39 degrees of humeral external rotation during arm elevation), and an additional control group included patients not fulfilling the criteria (n=11, not considered here)  **Intervention (n=10):** standard therapy (see below) plus specific end-range mobilisation / scapular mobilisation  **Comparison (n=12):** standardised treatment: passive mid-range mobilisation, flexion and abduction stretching techniques, physical modalities (i.e., ultrasound, shortwave diathermy, and/or electrotherapy), active exercises  **Dose:** 2 sessions per week for 3 months  **Providers:** physical therapists with at least 3 years of experience | **Results**  At 8 weeks:   - Hand-behind-back reach, humeral rotation and FLEX-SF disability scores significantly improved (p=0.002 to <0.0005) in the criteria intervention group compared to the criteria control group   ***Specific adverse events:*** not reported |
| **Temporomandibular disorders** |  |  |
| Craane 2012  Belgium  **Focus:** RCT investigated the effectiveness of physical therapy (joint mobilisation, massage, exercises) in anterior disc displacement without reduction of the temporomandibular joint  **Duration:** 6 weeks  **Follow-up:** one year  **Quality:** high  **PARTICIPANTS**:  **N:** 49 (96% female)  **Age:** 34.7 to 38.5 years  **Inclusion:** adults >18 years with temporomandibular joint disc displacement without reduction; exclusions: orofacial trauma, systemic disorders, cervical disorders, neurologic disorders | **Intervention type:** physiotherapy  **Intervention (n=23):** physical therapy protocol: information and counselling, rest position of the tongue, stretching of painful muscles, rotation exercises, jaw mobilisation; home exercise programme  **Comparison (n=26):** no treatment  **Dose:** 9 physical therapy sessions over 6 weeks (twice weekly for 3 weeks, once weekly for the remaining 3 weeks)  **Providers:** 4 physical therapists who had special training in the management of temporomandibular disorders | **Results**  At 52 weeks:   \| **Outcome** \| **Physical therapy** \| **Control** \| **p-value** \| \| --- \| --- \| --- \| --- \| \| MMOa (maximal active mouth opening) (SD) \| 42.7 (5.7) \| 46.5 (7.1) \| NS \| \| MMOp (maximal passive mouth opening) (SD) \| 45.4 (5.6) \| 49 (7) \| NS \| \| PPTm (pain pressure threshold, masseter muscle) (SD) \| 202.8 (49.8) \| 207.4 (74) \| NS \| \| PPTt (pain  pressure threshold, temporalis muscle) (SD) \| 320.8 (89.1) \| 326.1 (136.8) \| NS \| \| MFIQ (mandibular function impairment questionnaire) (SD) \| 3.7 (4.6) \| 4.1 (3.9) \| NS \| \| Pain VAS (25th- 75th perc) \| 2 (0-16) \| 2.5 (0-13) \| NS \| \| PRItotal (total pain rating index) (25th- 75th perc) \| 3.5 (0-7) \| 3.5 (1-9) \| NS \| |
| Guarda-Nardini 2012  Italy  **Focus:** RCT compared the effectiveness of botulinum toxin injection and fascial manipulation techniques in the management of myofascial pain of jaw muscles  **Duration:** 2 to 4 weeks  **Follow-up:** 3 months  **Quality:** low  **PARTICIPANTS**:  **N:** 30 (73% female)  **Age:** 23 to 69 years  **Inclusion:** Research Criteria for Temporomandibular Disorders diagnosis of myofascial pain with or without limited opening, bilateral pain lasting for at least 6 months | **Intervention type:** physiotherapy  **Intervention (n=15):** fascial manipulation (deep digital pressure)  **Comparison (n=15):** single session of multiple botulinum toxin injections  **Dose:** fascial manipulation: three 50 min sessions over 2 to 4 weeks  **Providers:** one trained therapist with more than 5 years experience | **Results**  At 3 months:   \| **Outcome** \| **Fascial manipulation** \| **Botulinum toxin** \| **p-value** \| \| --- \| --- \| --- \| --- \| \| VAS pain \| -3.5 \| -2.5 \| NS \| \| Mouth opening \| +0.4 \| +2.7 \| NS \| \| Left laterotrusion \| -0.2 \| +1.4 \| <0.01 \| \| Protrusion \| 0.0 \| +1.2 \| NS \| \| Right laterotrusion \| -0.8 \| +1.8 \| <0.01 \|   ***Specific adverse effects:*** not reported |
| Yoshida 2005  Japan  **Focus**: RCT investigated the effectiveness of simple manipulation with or without non-steroidal anti-inflammatory drugs (NSAIDs) in adults with temporomandibular joint disc displacement (closed lock)  **Duration:** single treatment  **Follow-up:** one year  **Quality:** low  **PARTICIPANTS**:  **N:** 305 (75% female)  **Age:** 18-74 years  **Inclusion:** adults >18 years with temporomandibular joint disc displacement (closed lock); exclusions: inability to understand the proposed therapy, current orthodontic treatment, bilateral closed lock, history of drug abuse, psychoses, periodontal disease in the incisor areas | **Intervention type:** physiotherapy  **Intervention (n=204):** jaw manipulation (thumb pressure applied against the labial side of upper anterior tooth while the lingual side of the lower incisor was pulled with the forefinger) plus NSAIDs  **Comparison (n=101):** NSAIDs  **Dose:** single jaw manipulation, NSAIDs (single administration)  **Providers:** not reported | **Results**  The success rate of treatment:   1. The mouth opened ≥36 mm and 2. The mandibular lateral movement increased to ≥6 mm  \| **Change in outcome** \| **Manual therapy plus NSAIDS** \| **NSAIDS** \| **p-value** \| \| --- \| --- \| --- \| --- \| \| N (%) treatment success rate at one year \| 172/204 (84.3%) \| 0% \| NR \| \| Pain (VAS) \| 1.8 after 1 wk with effective therapy, 4.0 with ineffective therapy \| NR \| NR \| \| Maximum mouth opening \| 39.4 mm with effective therapy, 27.1 mm with ineffective therapy \| not significantly changed from initial value of 28.4 mm \| NR \| \| Presence of clicking or crepitus \| present in patients with improvement \| not present \| NR \|   ***Specific adverse effects:*** not reported |
| Kalamir 2010  Australia  **Focus**: RCT investigated the effectiveness of IMT (with or without education and self-care) compared to no treatment in adults with myogenous temporomandibular disorders (TMD)  **Duration:** 5 weeks  **Follow-up:** 6 months post-treatment  **Quality:** high  **PARTICIPANTS**:  **N:** 30 (60% female)  **Age:** 32 years  **Inclusion:** adults 18-50 years with myogenous TMD for at least 3 months; exclusions: malignancy in the last 5 years, toothless, arthritides, fractures, dislocations, instability of jaws or neck, metabolic disease, rheumatologic disorders, haematological disorders | **Intervention type**: chiropractic  **Intervention 1 (n=10):** IMT (intra-oral temporalis release; intra-oral medial and lateral pterygoid technique; intra-oral sphenopalatine ganglion technique)  **Intervention 2 (n=10):** IMT + education (lecture on basic temporomandibular joint anatomy, biomechanics, disc displacement, dysfunction) + self-care (mandibular home exercises)  **Comparison (n=10):** no treatment  **Dose:** mandibular home exercises twice a day; IMT two 15-min sessions per week; education (2-min lectures in 4 visits)  **Providers:** chiropractic practitioner | **Results**  6 months post treatment   \| **Change in outcome** \| **Manual therapy** \| **Manual therapy + education + self-care** \| **No treatment** \| **p-value** \| \| --- \| --- \| --- \| --- \| --- \| \| Pain at rest (graded chronic pain scale)  Mean \| 0.60  [0.0, 1.20] \| 1.80  [0.74, 2.86] \| 3.40  [2.13, 4.67] \| <0.01 \| \| Pain on opening (graded chronic pain scale)  Mean [95% CI] \| 1.10  [0.01, 2.19] \| 2.70  [1.69, 3.71] \| 4.40  [2.71, 6.09] \| <0.01 \| \| Pain on clenching (graded chronic pain scale)  Mean [95% CI] \| 1.50  [0.47, 2.53] \| 1.70  [0.87, 2.53] \| 5.30  [3.68, 6.92] \| <0.01 \| \| Opening range (mm)  Mean [95% CI] \| 41.50  [38.76, 44.24] \| 48.30  [44.59, 52.01] \| 36.60  [30.11, 42.90] \| 0.01 \|   ***Specific adverse effects:*** none in any participant |
| Cuccia 2010  Italy  **Focus**: RCT investigated the effectiveness of osteopathic manual therapy compared to conventional conservative treatment in adults with temporomandibular disorders  **Duration:** 6 months  **Follow-up:** 2 months post-treatment  **Quality:** low  **PARTICIPANTS**:  **N:** 50 (56% female)  **Age:** 38.4 SD15.33 to 40.6 SD11.03 years  **Inclusion:** adults 18-50 years with temporomandibular disorders (temporomandibular index ≥ 0.08), pain intensity of VAS ≥ 40mm; exclusions: adverse event with osteopathic manual therapy, previous treatment for temporomandibular disorders, use of analgesics, anti-inflammatory drugs, dental prosthesis, any other oro-facial pain condition, neurological or psychiatric disorder | **Intervention type**: osteopathy  **Intervention (n=25):** osteopathic manual therapy directed to cervical and temporomandibular joint regions (myofascial release, balanced membranous tension, muscle energy, joint articulation, high velocity low amplitude thrust, and cranial-sacral therapy)  **Comparison (n=25):** conventional conservative treatment (oral appliance, gentle muscle stretching, relaxing exercise, hot/cold packs, transcutaneous electrical nerve stimulation)  **Dose:** osteopathic manual therapy 15-25 min sessions each, conventional not reported  **Providers:** osteopathic manual therapy: doctor of osteopathy, conventional: gnathology specialist | **Results**  2 months post treatment (8 months post-baseline)   \| **Change in outcome** \| **Osteopathic manual therapy** \| **Conventional conservative treatment** \| **p-value** \| \| --- \| --- \| --- \| --- \| \| Pain (VAS scale)  Mean ± SD \| 3.8 ± 1.26 \| 4.4 ± 1.75 \| >0.05 (NS) \| \| Maximal mouth opening (mm)  Mean ± SD \| 42.9 ± 2.69 \| 40.4 ± 2.41 \| 0.001 \| \| Lateral movement of the head around its axis (degrees) Mean ± SD \| 80.5 ± 5.44 \| 72.4 ± 2.95 \| 0.000 \|   ***Specific adverse effects:*** not reported |
| **Cervicogenic headache** |  |  |
| von Piekartz 2011  The Netherlands  **Focus:** RCT investigating effects of temporomandibular (TMD) and cervical manual therapy compared to cervical manual therapy alone in adults with cervicogenic headache (CGH) on headache intensity, neck disability, and TMD outcomes  **Duration:** maximum of 42 days  **Follow-up:** 6 months  **Quality:** high  **PARTICIPANTS:**  **N:** 43 (64% female)  **Age:** 36 years  **Inclusion:** patients with CGH > 3 months, no prior TMD treatment, neck disability index (NDI)>15 points, and at least 1 of the 4 TMD signs present (joint sounds, deviation during mouth opening, extraoral muscle pain, and pain during passive mouth opening); exclusions were orthodontic treatment or experience of neurologic pain in the head in the past 3 years | **Intervention type:** physiotherapy  **Intervention (n=22):** manual therapy (orofacial treatment) applied to the TMD region – consisting of accessory movements to TMD region, masticatory muscle techniques (tender-trigger point treatment and muscle stretching), active/passive movements facilitating optimal function of cranial nerve tissue, coordination exercises, and home exercises; plus usual care (cervical manual therapy applied to the cranio-cervical region)  **Comparison (n=21):** usual care (cervical manual therapy)  **Dose:** each session of 30 minutes daily, 6 sessions  **Providers:** first contact practitioners trained for manual therapy; experimental arm investigators were additionally trained for 200 hours focusing on the assessment of craniomandibular and craniofacial pain | **Results**  At 6 months:   \| **Outcome** \| **Orofacial therapy +**  **usual manual therapy** \| **Usual manual therapy** \| **p-value** \| \| --- \| --- \| --- \| --- \| \| Pain intensity (coloured analogue scale 0-10) \| 2.1 \| 7.0 \| ≤ 0.05 \| \| Neck disability index \| 6.3 \| 16.0 \| NS \| \| Mouth opening (mm) \| 53.5 SD3.2 \| 41.6 SD4.3 \| NS \| \| Pain intensity during mouth opening (VAS mm) \| 0.9 SD8.0 \| 53.0 SD7.0 \| ≤ 0.05 \| \| Deviation present (%) \| 10.0 \| 33.9 \| ≤ 0.05 \| \| Sound (click) present (%) \| 25.0 \| 42.0 \| ≤ 0.05 \|   ***Specific adverse effects:*** not reported |
| Youssef 2013  Egypt  **Focus:** RCT comparing the effects of cervical mobilisations with massage in the management of cervicogenic headache  **Duration:** 6 weeks  **Follow-up:** no post-intervention follow-up  **Quality:** medium  **PARTICIPANTS:**  **N:** 36 (39% female)  **Age:** 31 to 32.4 years  **Inclusion:** 18 to 40 years with recurrent headache and neck pain for ≥2 months, symptoms of cervicogenic headache; exclusions: migraine, cluster headache, cervical radiculopathy, entrapment neuropathy, myelopathy, rheumatoid arthritis, previous surgery of the cervical spine, pregnancy, whiplash trauma | **Intervention type:** physiotherapy  **Intervention (n=18):** passive spinal mobilisations (low velocity / high amplitude, small oscillatory movements to the upper cervical vertebrae)  **Comparison (n=18):** massage therapy (myofascial release, manual cervical traction, trigger point therapy, facilitated stretching techniques)  **Dose:** 12 sessions (2 sessions per week for 6 weeks), 30-40 min sessions  **Providers:** not reported | **Results**  At 6 weeks:   \| **Outcome** \| **Mobilisation** \| **Massage** \| **p-value** \| \| --- \| --- \| --- \| --- \| \| Headache intensity \| 2.2 (0.7) \| 4.3 (0.68) \| 0.00 \| \| Headache frequency \| 1.94 (0.64) \| 3.9 (0.47) \| 0.00 \| \| Headache duration \| 1.3 (0.23) \| 1.62 (0.51) \| 0.008 \| \| Neck Disability Index \| 18.9 (3.7) \| 17.5 (3.5) \| NS \| \| Neck flexion \| 3.9 (0.4) \| 3.52 (0.47) \| 0.02 \| \| Neck extension \| 2.92 (0.26) \| 2.59 (0.41) \| 0.00 \|   Results for neck rotation and lateral flexion also significantly better for the mobilisation group.  ***Specific adverse effects:*** not reported |
| **Tension-type headache** |  |  |
| Ajimsha 2011  India  **Focus:** RCT assessing the effects of myofascial release in patients with tension-type headache  **Duration:** 12 weeks  **Follow-up:** 20 weeks  **Quality:** low  **PARTICIPANTS:**  **N:** 63 (64% female)  **Age:** 43 to 44.7 years  **Inclusion:** 18 to 50 years with a diagnosis of episodic or chronic tension-type headache for at least 12 months; exclusions: history of migraine, secondary headaches | **Intervention type:** physiotherapy  **Intervention 1 (DT-MFR, n=22):** myofascial release, direct technique (upper trapezius release, lateral / anterior cranial techniques, deep posterior myofasciae, release of cranial base and suboccipital myofascia, decompression of the occipital condyles, release of the temporalis fascia, release of the epicranial aponeurosis)  **Intervention 2 (IDT-MFR, n=22):** myofascial release, indirect technique (Gross stretch of the posterior cervical musculature, cranial base release, hair pull, ear pull, stretch of face muscles, gross stretch of the sternocleidomastoid)  **Comparison (n=12):** slow soft stroking over the areas treated in the myofascial release groups  **Dose:** twice weekly for 12 weeks, 1 h sessions  **Providers:** not reported | **Results**  At 20 weeks:   \| **Outcome** \| **DT-MFR** \| **IDT-MFR** \| **Control** \| **p-value** \| \| --- \| --- \| --- \| --- \| --- \| \| Headache frequency  mean (SD) \| 4.9 (1.7) \| 5.7 (1.3) \| 10.4 (2.7) \| 0.51 DT vs IDT, <0.001 DT or IDT vs control \|   ***Specific adverse effects:*** no serious adverse events |
| Anderson 2006  Canada  **Focus:** RCT the effect of adding osteopathic manual treatment (OMT) to progressive muscular relaxation (PMR) exercise in patients with tension-type headache  **Duration**: 3 weeks  **Follow-up**: 5 weeks  **Quality**: medium  **PARTICIPANTS:**  **N**: 29 (NR% female)  **Age**: NR  **Inclusion**: adults>16 years with tension-type headache (frequent episodic, chronic, or probable)  **Exclusions**: pts taking pain medication or receiving manual therapy | **Intervention type:** osteopathy  **Intervention (n=14):** OMT (unwinding, inhibition, and stretching techniques with a focus on pelvis, cranium, cervical and upper thoracic spine, upper ribs; joint mobilisations including functional, muscle energy, strain/counterstrain, and osteoarticular techniques) + progressive muscular relaxation  **Comparison (n=12):** progressive muscular relaxation (pts were given audio tape and typed instructions on exercise on contracting major muscle groups, moving feet up, sensation experience, and then relaxation)  **Dose:** OMT (once a week for 3 weeks)  (once a day 20 min session for 3 weeks)  **Providers:** not reported | **Results**  3 weeks post-treatment   \| **Change in outcome** \| **Osteopathic manual treatment** \| **Progressive muscular relaxation** \| **p-value** \| \| --- \| --- \| --- \| --- \| \| Number of headache free days per week  Mean (SD) \| 1.79  (1.42) \| 0.21  (1.68) \| 0.016 \| \| Headache degree of improvement on VAS  Mean (SD) \| 1.88  (1.39) \| 0.65  (1.95) \| 0.075 \| \| Headache diary rating  (% improvement)  Mean (SD) on VAS \| 57.56  (27.32) \| 15.63  (73.46) \| 0.059 \| \| Improvement in worst headache intensity  Mean (SD) on VAS \| 1.50  (1.09) \| 0.92  (1.50) \| 0.264 \|   ***Specific adverse effects:*** not reported |
| Castien 2011  Castien 2009  The Netherlands  **Focus:** RCT compared the effectiveness of manual therapy (MT) and usual care by the general practitioner in patients with chronic tension-type headache  **Duration:** 8 weeks  **Follow-up:** 26 weeks  **Quality**: medium  **PARTICIPANTS:**  **N:** 82 (78% female)  **Age:** 40 years  **Inclusion:** adults 18-65 years who met chronic tension-type headache criteria according to the classification of headaches of the International Headache Society (occurring on at least 15 days per month for > 3 months, lasting for hours or continuous; at least one of the following characteristics present: bilateral location, pressing quality, mild/moderate intensity, photophobia, phonophobia, mild nausea)  **Exclusion**: rheumatoid arthritis, malignancy, pregnancy, intake of opioids/analgesics on regular basis for > 3 months, receiving MT 2 months before the study enrolment | **Intervention type:** physiotherapy  **Intervention (n=41**): MT consisted of cervical/thoracic spine mobilisation, craniocervical exercises, postural correction  **Intervention (n=41):** usual care by the general practitioner provided information, re-assurance and advice, and discussed the benefits of life-style changes; if necessary, pain medication and NSAIDs were prescribed  **Dose:** usual care by the general practitioner (2-3 visits); MT (up to 9 sessions each 30 minutes duration)  **Providers:** trained manual therapists, registered members of the national association of manual therapists with an average experience of 10 years who additionally completed a course on the mechanical diagnosis and management of disorders of the cervical spine provided by the McKenzie Institute | **Results**   \| **Change in outcome** \| **Manual therapy** \| **Usual care by the general practitioner** \| **Difference**  **p-value**  **(95% CI)** \| \| --- \| --- \| --- \| --- \| \| **8 weeks post-baseline** \| \| \| \| \| 50% reduction in headache frequency (n/N) \| 35/40 (87.5%) \| 11/40  (27.5%) \| <0.05  3.2 (1.9, 5.3) \| \| Headache days frequency \| -9.1 SD3.8 \| -2.7 SD4.3 \| -6.4 (-8.32, -4.56) \| \| Headache pain intensity  (score 0-10) \| -2.7 SD0.9 \| -0.9 SD2.4 \| -1.8 (-3.07, -0.67) \| \| Headache Disability Inventory (score 0-100) \| -17.4 SD16.1 \| -5.8 SD12.8 \| -11.6 (-18.1, -5.1) \| \| Cervical range of movement (degrees) \| 18.8 SD32.5 \| 2.0 SD31.4 \| 16.8 (2.42, 31.32) \| \| Endurance of the neck flexor (sec) \| 13.0 SD16.8 \| 2.9 SD17.2 \| 10.0 (2.35, 17.74) \| \| Headache Impact Test-6 \| -8.9 SD7.1 \| -2.4 SD6.5 \| -6.5 (-9.62, -3.52) \| \| **26 weeks post-baseline** \| \| \| \| \| 50% reduction in headache frequency (n/N) \| 31/38 (81.6%) \| 15/37  (40.5%) \| <0.05  2.0 (1.3, 3.0) \| \| Headache days frequency \| -9.1 SD4.2 \| -4.1 SD4.4 \| -4.9 (-6.95, -2.98) \| \| Headache pain intensity \| -3.1 SD2.8 \| -1.7 SD2.5 \| -1.4 (-2.69, -0.16) \| \| Headache Disability Inventory (score 0-100) \| -20.0 D22.6 \| -9.9 SD18.0 \| -10.1 (-19.5, ‑0.64) \| \| Cervical range of movement (degrees) \| 15.6 SD37.8 \| 5.3 SD45.0 \| 10.2 (-9.16, 29.63) \| \| Endurance of the neck flexor (sec) \| 13.3 SD20.7 \| 13.0 SD25.0 \| 0.3 (-10.38, 11.03) \| \| Headache Impact Test-6 \| -10.6 SD8.4 \| -5.5 SD8.6 \| -5.0 (-9.02, -1.16) \| \| Perceived recovery (n/N) \| 35/38 (87.5%) \| 10/37 (25.0%) \| 62.5 (48.4, 79.3) \|   ***Specific adverse effects:*** not reported |
| van Ettekoven 2006  The Netherlands    **Focus:** RCT investigated the effectiveness of exercise (craniocervical flexion) combined with physiotherapy in patients with tension-type headache  **Duration:** 6 weeks  **Follow-up:** 7 months  **Quality:** high  **PARTICIPANTS:**  **N:** 81 (81% female)  **Age:** 45 years  **Inclusion:** adults 18-65 years who met chronic tension-type headache criteria according to the classification of headaches of the International Headache Society (occurring on at least 15 days per month for > 3 months, lasting for hours or continuous; at least one of the following characteristics present: bilateral location, pressing quality, mild/moderate intensity, photophobia, phonophobia, mild nausea)  **Exclusion**: other types of headache, cervical function problems, physiotherapy for the treatment of tension-type headache received within the last 6 months | **Intervention type:** physiotherapy  **Intervention (n=39**): craniocervical flexion exercise (low-load endurance exercise using a latex band) plus physiotherapy (Western massage, oscillation techniques, and instruction on postural correction)  **Intervention (n=42):** physiotherapy (Western massage incl. friction massage, oscillation techniques (low-velocity, passive cervical joint mobilisation), and instruction on postural correction)  **Dose:** craniocervical flexion exercise (max 15 minute session; exercise done at home twice a day for 10 minute session  **Providers:** explicitly trained experienced senior physiotherapists | **Results**   \| **Change in outcome** \| **Physiotherapy plus craniocervical flexion** \| **Physiotherapy** \| **Difference**  **p-value**  **(95% CI)** \| \| --- \| --- \| --- \| --- \| \| **6 weeks post-baseline** \| \| \| \| \| ≥50% reduction in headache frequency (n/N) \| 32/39  (82%) \| 22/42  (52%) \| NR \| \| Headache days frequency  Mean (SD) \| NR \| NR \| 0.94 (-0.71, 1.81) \| \| Headache pain intensity  (score 0-10) Mean (SD) \| NR \| NR \| -0.04 (-1.09, 1.01) \| \| Headache duration (h/day)  Mean (SD) \| NR \| NR \| -0.18 (-2.07, 1.70) \| \| **6 months post-baseline** \| \| \| \| \| ≥50% reduction in headache frequency (n/N) \| 33/39 (85%) \| 14/42 (35%) \| NR \| \| Headache days frequency Mean (SD) \| NR \| NR \| 1.95 (1.14, 2.76) \| \| Headache pain intensity  (score 0-10) Mean (SD) \| NR \| NR \| 1.78 (0.82, 2.74) \| \| Headache duration (h/day)  Mean (SD) \| NR \| NR \| 2.07 (0.12, 4.03) \| \| **Quality of life (SF-36)** \|  \| \| \| \| Emotional well-being \| NR \| NR \| p=0.014 \| \| Limitations due to mental health \| NR \| NR \| p=0.05 \| \| Vitality \| NR \| NR \| p=0.039 \| \| Bodily pain \| NR \| NR \| p=0.017 \|   ***Specific adverse effects:*** not reported |
| Vernon 2009  Canada  **Focus:** RCT compared the effectiveness of cervical manipulation, medical treatment, and the combination of two treatments in adults with tension-type headache  **Duration:** 10-14 weeks  **Follow-up:** 26 weeks  **Quality:** medium  **PARTICIPANTS:**  **N:** 20 (80% female)  **Age:** mean range (29-43 years)  **Inclusion:** adults 18-50 years who met chronic tension-type headache criteria according to the classification of headaches of the International Headache Society (occurring 10-25 days per month, no more than two unilateral headaches per month, <50 on Zung Depression scale, no contraindications to manipulation/amitriptyline, no history of whiplash injury, not receiving manual treatment within the past year of the trial enrolment)  **Exclusion**: not reported | **Intervention type:** chiropractic  **Intervention 1 (n=5**): chiropractic cervical manipulation 10 weeks of duration (brief minimal preparatory soft tissue massage to the cervical paraspinal tissues followed by high velocity, low amplitude thrusting manipulation to any dysfunctional joints from occiput to third thoracic vertebrae)  **Intervention 2 (n=7):** medical treatment (10-25mg/d amitriptyline for 14 weeks)  **Intervention 3 (n=3):** chiropractic cervical manipulation plus medical treatment (amitriptyline)  **Comparison (n=5):** sham chiropractic plus placebo  **Dose:** manual therapy (3 times per week for 6 weeks followed by once per week for 4 weeks); medical treatment (amitriptyline given at 10 mg/d for the first 2 weeks and followed by 25 mg/d for the remaining 12 weeks)  **Providers:** chiropractors with >5 years of experience | **Results**  **The adjusted analysis**  Number of headache days in the last 28 days of the trial (at 14 weeks follow-up)  Effect of manipulation plus medical treatment: -8.4, 95% CI: -15.8, -1.1 (SS)  Main effect of manipulation: 2.0, 95% CI: -3.0, 7.0 (NS)  Main effect of medical treatment: 3.1, 95% CI: -1.6, 7.8 (NS)  ***Specific adverse effects:*** Nine participants had adverse events, four with manipulation (chiropractic-related events such as minor aggravation of neck pain) and five with amitriptyline (nausea, tiredness, change in sleep, dry mouth, and constipation) |
| **Miscellaneous headaches** |  |  |
| de Hertogh 2009  The Netherlands  Focus: RCT compared manual therapy plus usual care to usual care alone in adults with miscellaneous headaches (migraine, tension-type headache, cervicogenic headache)  Duration: 6 weeks  Follow-up: 27 weeks  Quality: Medium  PARTICIPANTS:  N: 37 (76% female)  Age: 43 years  Inclusion: adults>18 years with miscellaneous headaches (migraine, tension-type headache, cervicogenic headache) accompanied by neck pain at least for 2 months, twice a month or more often, headache impact test (HIT-6) score > 56; exclusions: cluster headache, trigeminal neuralgia, peripheral neuropathies, chronic musculoskeletal disorders, rheumatoid arthritis, Down syndrome, history of surgery in cervical region, pregnancy, manipulation treatment in the past 12 months | **Intervention type:** physiotherapy  **Intervention (n=18):** manual therapy (cervical joint mobilisation and stabilising exercise – craniocervical flexion exercise)  **Comparison (n=19):** usual care (education, prophylactic and attack medication)  **Dose:** 12 sessions 30 min each (twice a week over 6 weeks)  **Providers:** not reported | **RESULTS**   \| **Change in outcome** \| **Manual therapy + usual care** \| **Usual care** \| **p-value** \| \| --- \| --- \| --- \| --- \| \| Global perceived effect (n/N of responders) \| 6/14 \| 7/13 \| NS \| \| Headache impact test–6  Mean (SD) \| 55.21 (9.75) \| 56.80 (6.46) \| NS \| \| Headache intensity at 26 weeks Mean (SD) \| 19.92 (29.09) \| 13.55 (24.23) \| NS \| \| 50% reduction in headache frequency (n/N achieved) \| 12/14 \| 12/13 \| NS \| \| Absenteeism (n/N absent) \| 2/13 \| 2/11 \| NS \|   ***Specific adverse effects:*** not reported |
| Foster 2004  USA  **Focus:** RCT of manual therapy (Trager method) and medication effects in with miscellaneous headaches (migraine, tension-type, cluster)  **Duration:** 6 weeks  **Follow-up:** 6 weeks  **Quality:** medium  **PARTICIPANTS:**  **N:** 33 (86% female)  **Age:** 30 years  **Inclusion:** adults 18-65 years with miscellaneous chronic headaches (migraine, tension-type, cluster) for > 6 months (>1 headache per week), pain intensity range: 25-85 on a VAS of 0-100 scale  **Exclusion**: life threatening aetiology of headache, contraindications to manual therapy | **Intervention type:** Trager method  **Intervention 1 (n=14**): manual therapy/Trager (gentle mobilisation of the joint areas of the head, neck, upper back, and shoulders with slow movements to encourage relaxation and movement patterns) plus medication  **Intervention 2 (n=7):** attention therapy (visit and discussion with physician about medication intake, previous week’s headaches, and perception of well-being) plus medication  **Comparison (n=12):** no treatment (only medication)  **Dose:** manual therapy (one hour sessions) for 6 weeks; attention therapy (15-20 minute sessions) for 6 weeks  **Providers:** physician | **RESULTS**   \| **Change in outcome**  (6 weeks post-baseline) \| **Manual therapy-trager** \| **Attention treatment** \| **No treatment** \| **p-value** \| \| --- \| --- \| --- \| --- \| --- \| \| Headache duration (hours)  Mean change (SD) \| -0.6 (3.6) \| –0.3 (1.6) \| 1.8 (2.7) \| <0.05 (Trager or attention versus no treatment) \| \| Headache QOL score  Mean change (SD) \| 0.4 (0.8) \| 0.8 (0.8) \| -0.5 (0.7) \| 0.001 (Trager or attention versus no treatment) \| \| Medication use (total N of pills taken biweekly)  Mean change (SD) \| -6.7 (9.2) \| -3.8 (7.9) \| 6.2 (18.6) \| NS \| \| Headache intensity (VAS score range: 0-100)  Mean change (SD) \| 0.3 (20.1) \| -4.2 (20.6) \| 6.6 (10.4) \| NS \| \| Headache episodes (N per week)  Mean change (SD) \| -2.5 (4.6) \| -0.3 (9.7) \| 1.3 (5.4) \| NS \|   ***Specific adverse effects:*** not observed |
| **Fibromyalgia** |  |  |
| Castro-Sánchez 2011a  Spain  **Focus:** RCT of the effects of cranio-sacral therapy on pain and heart rate variability in patients with fibromyalgia  **Duration:** 20 weeks  **Follow-up:** 1 year  **Quality:** medium  **PARTICIPANTS:**  **N:** 92 (100% female)  **Age:** 51.3 SD13.1 to 53.9 SD10.1 years  **Inclusion:** patients with fibromyalgia, 16 to 65 years | **Intervention type:** cranio-sacral therapy  **Intervention (n=46):** cranio-sacral therapy; sequence of manipulative  therapy: still point (in feet), pelvic diaphragm release, scapular girdle release, frontal lift, parietal lift, compression–decompression of sphenobasilar fascia, decompression of temporal fascia, compression–decompression of temporomandibular joint and evaluation of dural tube (balance of dura mater)  **Comparison (n=46):** sham therapy with disconnected magnetotherapy equipment  **Dose:** twice a week 1 h sessions for 20 weeks  **Providers:** cranio-sacral and magnetotherapists  **Further information available on:** heart rate, heart rate variability, body composition | **Results**   - *Clinical global impression of improvement (Likert scale):* significantly better in intervention group than control group after treatment and 2 months post-treatment but not 1 year post-treatment - *Clinical global impression of severity (Likert scale):* significantly better in intervention group than control group after treatment but not at 2 months or 1 year post-treatment - *Pain:* 20 weeks: significant reduction in pain at 13 of 18 tender points in intervention group, no reduction in control group, significant difference between groups; 1 year: reduction remained significant for 4 tender points   ***Specific adverse effects:*** not reported |
| Castro-Sánchez 2011a  Spain  **Focus:** RCT of the effects of massage-myofascial release therapy on pain, anxiety, quality of sleep, depression, and quality of life in patients with fibromyalgia  **Duration:** 20 weeks  **Follow-up:** 6 months post-intervention  **Quality:** low  **PARTICIPANTS:**  **N:** 59 (95% female)  **Age:** 49.3 SD11.6 to 46.3 SD12.3 year  **Inclusion:** patients with fibromyalgia syndrome, age 18 to 65 years, no regular physical activity | **Intervention type:** physiotherapy  **Intervention (n=30):** massage-myofascial release protocol: massage-myofascial release at insertion of the temporal muscle, release of falx cerebri by frontal lift, release of tentorium cerebella by synchronization of temporal, assisted release of cervical fascia, release of anterior thoracic wall, release of pectoral region, lumbosacral decompression, release of gluteal fascia, transversal sliding of wrist flexors and fingers, and release of quadriceps fascia  **Comparison (n=29):** sham therapy with disconnected magnetotherapy equipment  **Dose:** *intervention:* weekly 90 min session for 20 weeks; *control:*  **Providers:** physiotherapist specialized in massage-myofascial therapy  **Further information available on:** sleep parameters, state and trait anxiety | **Results**   - *Pain:* 20 weeks: VAS pain score significantly reduced versus baseline and control (p<0.043); significantly greater reduction in pain at 8of 18 tender points in intervention compared to control group; 6 months: reduction remained significant for 3 tender points; no significant difference in VAS score - *Quality of life (SF-36):* significantly better for 4 of 8 domains than placebo at 20 weeks (physical function, physical role, body pain, social function) but not at 6 months - *Beck depression inventory:* no significant difference between groups   ***Specific adverse effects:*** not reported |
| **Myofascial pain syndrome** |  |  |
| Gemmell 2008a  UK  **Focus:** RCT of the immediate effect of a ischaemic compression and activator trigger point therapy on active upper trapezius trigger points  **Duration:** single treatment  **Follow-up:** no post-intervention follow-up  **Quality:** medium  **PARTICIPANTS:**  **N:** 52 (67 to 72% female)  **Age:** 28 SD9.1 to 29 SD8.5 years  **Inclusion:** patients with active upper trapezius trigger points of more than 12 weeks’ duration rated at least 4 on an 11-point numerical rating scale, male and female between 18 and 55 years | **Intervention type:** chiropractic  **Intervention 1 (n=25):** ischaemic compression therapy: continuous,  perpendicular deep thumb pressure to the identified upper trapezius trigger point for 30 to 60 s; pressure was released according to which of the following occurred first: a palpable decrease in trigger point tension or once 60 s had passed  **Intervention 2 (n=27):** Activator trigger point therapy: a force setting of 3 was used (170 N); to treat the trigger point, the Activator instrument was placed perpendicular over the identified TrP and 10 thrusts were delivered, with a rate of one thrust per second  **Dose:** single treatment  **Providers:** chiropractor  **Further information available on:** demographic details | **Results**   - Patient Global Impression of Change (PGIC, 7 point scale, ‘very much improved’ to ‘very much worse’) - Pain numeric rating scale (NRS) - Pressure pain threshold (PPT)   Results reported as % participants undergoing a meaningful clinical improvement   \|  \| **Ischaemic compression** \| **Activator** \| **p** \| \| --- \| --- \| --- \| --- \| \| **PGIC** \| 78% \| 72% \| NS \| \| **NRS** \| 41% \| 36% \| NS \| \| **PPT** \| 30% \| 32% \| NS \|   ***Specific adverse effects:*** not reported |
| Gemmell 2008b  UK  **Focus:** RCT of the immediate effect of a ischaemic compression and trigger point pressure release on neck pain and upper trapezius trigger points  **Duration:** single treatment  **Follow-up:** no post-intervention follow-up  **Quality:** medium  **PARTICIPANTS:**  **N:** 45 (% female not stated)  **Age:** 23 SD1.5 to 24 SD4.6 years  **Inclusion:** participants with mechanical neck pain for <3 months; active upper trapezius trigger point; pain of at least 30 mm on VAS; decreased lateral flexion to the opposite side of the active upper trapezius trigger point, 18 to 55 years | **Intervention type:** chiropractic  **Intervention 1 (n=15):** ischaemic compression therapy: continuous,  perpendicular deep thumb pressure to the identified upper trapezius trigger point for 30 to 60 s; pressure was released according to which of the following occurred first: a palpable decrease in trigger point tension or once 60 s had passed  **Intervention 2 (n=15):** trigger point (TrP) pressure release: clinician applied non-painful slowly increasing pressure with the thumb over the trigger point until a tissue resistance barrier was felt; level of pressure was maintained until release of the tissue barrier was felt, at which time pressure was increased until a new barrier was reached; process was repeated until there was no trigger point tension / tenderness or 90 s had elapsed, whichever occurred first  **Control (n=15):** sham procedure (detuned ultrasound)  **Dose:** single treatment  **Providers:** chiropractor  **Further information available on:** demographic details | **Results**   - % improved: pain reduction of at least 20 mm on VAS  \|  \| **Ischaemic compression (IC)** \| **TrP pressure release** \| **Sham** \| **p** \| \| --- \| --- \| --- \| --- \| --- \| \| **% improved (VAS)** \| 60.0% \| 46.7% \| 26.7% \| IC versus sham <0.05 \| \| **Neck pain (VAS, mm)** \| 22.93 SD12.76 \| 27.13 SD16.40 \| 22.67 SD8.21 \| NS \| \| **PPT (kg/m^2^)** \| 4.45 SD1.69 \| 3.77 SD1.76 \| 3.37 SD1.62 \| NS \| \| **Lateral cervical flexion (°)** \| 50.5 SD8.6 \| 49.1 SD10.4 \| 49.1 SD8.3 \| NS \|   ***Specific adverse effects:*** not reported |
| Nagrale 2010  India  **Focus:** RCT comparing the effects of muscle energy techniques versus an integrated neuromuscular inhibition technique in deactivating upper trapezius trigger points (improvement in pain, range of motion, disability)  **Duration:** 4 weeks  **Follow-up:** no post-intervention follow-up  **Quality:** medium  **PARTICIPANTS:**  **N:** 60 (58% female)  **Age:** 27.6 SD4.3 to 28.2 SD4.8 years  **Inclusion:** 18 to 55 years, non-specific neck pain of <3 months’ duration, active upper trapezius trigger points | **Intervention type:** physiotherapy  **Intervention 1 (n=30):** muscle energy (MET) treatment as per Lewit’s post-isometric relaxation approach  **Intervention 2 (n=30):** integrated neuromuscular inhibition technique (INIT): ischaemic compression plus strain-counterstrain plus muscle energy technique  **Dose:** 3 times per week for 4 consecutive weeks  **Providers:** not stated | **Results** (4 weeks)   \|  \| **MET** \| **INIT** \| **p** \| \| --- \| --- \| --- \| --- \| \| **Pain (VAS)** \| 6.10 SD0.68 \| 5.28 SD0.47 \| <0.01 \| \| **Neck disability index** \| 31.88 SD4.4 \| 27.19 SD3.7 \| <0.01 \| \| **Lateral cervical flexion (°)** \| 29.33 SD1.72 \| 34.44 SD1.2 \| <0.01 \|   ***Specific adverse effects:*** not reported |
| Sarrafzadeh 2012  Iran  **Focus:** RCT comparing the effects of pressure release, phonophoresis of hydrocortisone (1%), and ultrasound on upper trapezius latent myofascial trigger point  **Duration:** 6 sessions  **Follow-up:** no post-intervention follow-up  **Quality:** low  **PARTICIPANTS:**  **N:** 60 (all female)  **Age:** 21.8 years  **Inclusion:** at least one myofascial trigger point in the upper trapezius; exclusions: fibromyalgia, history of whiplash injury or cervical spine surgery | **Intervention type:** physiotherapy  **Intervention 1 (n=15):** pressure release on the myofascial trigger point (90 sec)  **Intervention 2 (n=15):** phonophoresis of 1% hydrocortisone over the affected area (5 min)  **Intervention 3 (n=15):** ultrasound (5 min)  **Control (n=15):** no intervention  **Dose:** 6 sessions, time span not indicated  **Providers:** not reported | **Results**  After the intervention:   - Pain significantly decreased in all groups except in the control group - Pain pressure threshold increased significantly in all groups (p<0.001) - The range of active ipsilaterial and contralateral flexion significantly increased in all groups, except the control groups (<0.001) - Not reported whether there were significant differences between the intervention groups   ***Specific adverse events:*** not reported |
| **Asthma** |  |  |
| Mehl-Madrona 2007  USA  **Focus:** RCT of acupuncture, cranio-sacral therapy, a combination of the two, attention control, waiting list control in adults with asthma  **Duration:** 12 weeks  **Follow-up:** 6 months  **Quality:** medium  **PARTICIPANTS:**  **N:** 89 (73.5% female)  **Age:** median 37 years  **Inclusion:** adults with asthma (definition National Heart, Lung and Blood Institute), class II to IV asthma sufferers | **Intervention type:** osteopathy  10 to 16 participants per group  **Intervention 1:** 12 treatments of acupuncture (45 min sessions, twice weekly)  **Intervention 2:** 12 treatments of cranio-sacral therapy (45 min sessions, twice weekly)  **Intervention 3:** combination of cranio-sacral therapy with acupuncture (6 sessions each, 45 mins, one each weekly)  **Control 1:** attention control (6 sham cranio-sacral therapy and 6 educational classes)  **Control 2:** waiting list control (instructed to maintain normal asthma care regimens)  **Dose:** see above  **Providers:** acupuncturists, trained cranio-sacral therapists | **Results**   - Due to small numbers and no significant differences between intervention groups or control groups, groups were collapsed into ‘intervention’ and ‘control’ - No change in pulmonary function measures - Asthma Quality of Life score significantly more improved in intervention groups than control groups post-treatment (p=0.004), difference not significant any more at 6 months; QoL was improved significantly more post-treatment in groups with a single practitioner (i.e. not combination treatment, p=0.016) - Medication use was significantly reduced in the intervention groups compared to control, both post-treatment (p<0.001) and at 6 months follow-up (p=0.043) - No changes in the Beck Depression Scale - Overall no difference in Beck Anxiety Inventory intervention versus control, but there was a tendency for the groups with a single practitioner (i.e. longer treatment) to have reduced anxiety levels (p=0.031 at 3 months post-intervention)   ***Specific adverse effects:*** no adverse effects seen |
| Shaw 2006  UK  **Focus:** qualitative study of complementary therapy use in patients with asthma  **Duration:** single interviews  **Quality:** high  **PARTICIPANTS:**  **N:** 50 (54% female)  **Age:** age not reported, 21 adults, 29 children (with parents)  **Inclusion:** children and adults with asthma, variety of healthcare settings and socio-demographic backgrounds | **Intervention type:** various (chiropractic, osteopathy)  **Intervention:** complementary therapies including chiropractic, osteopathy, cranial osteopathy; of the participants, 31 used complementary therapy for asthma, 6 for other problems, 13 were non-users  **Dose:** not reported  **Providers:** settings: GP practice in affluent suburb, GP practice in deprived inner city area, NHS outpatient respiratory clinic, NHS outpatient homeopathic hospital, private complementary therapists | **OUTCOME ASSESSMENT**  ***Interviews:*** interviews with adults 25 mins to 1 h; paired interviews with children and parents 30 mins to 1.5 h (first half focused on child, second on parent); interviews recorded and transcribed, thematic analysis  **RESULTS**  **Interviews:**  ***Reasons for non-use:***   - Scepticism about complementary therapies: lack of scientific evidence, strong belief in “scientific medicine” - Trusted and wanted to follow advice from conventional doctors - Interested and open to trying complementary therapies but had not yet done so (no perceived need, not got round to it, financial cost, certain trigger factors could prompt use)   ***Complementary therapy use:***   - Mainly breathing techniques (e.g. Buteyko Method) and homeopathy - *Types:* last resort users (tried all conventional treatments first, escalation of medication with lack of benefit); pragmatic users (“shop around” to see whatever treatments will help in parallel to conventional medicine); committed users: complementary therapies are preferred first port-of-call; but all still using conventional medication - *Conventional medicine (push factors):* concerns about side effects, steroids, dislike of dependence on medication, concerns of escalation of medication - *Complementary therapy (pull factors):* desire for “natural” or “non-invasive” treatments, quality of complementary therapy consultations (holistic approach, listening, time), personal commitment to alternative philosophies of health, experience of effectiveness of complementary therapies - Benefits of self-help and taking control - Exploring a broader range of causes of asthma |
| **ADHD / learning disabilities** |  |  |
| Bierent-Vass 2005  Germany  **Focus:** RCT of the effects of osteopathic treatment for children with ADHD  **Duration:** 6 weeks  **Follow-up:** 4 weeks after the last treatment  **Quality:** low  **PARTICIPANTS:**  **N:** 77 (% female not reported)  **Age:** 6 to 14 years (details not reported)  **Inclusion:** children with attention deficit with or without hyperactivity (ADD / ADHD) | **Intervention type:** osteopathy  **Intervention (n=50):** osteopathic treatment; 4 treatments with intervals of 2 weeks  **Comparison (n=27):** no osteopathic treatment  **Dose:** see above  **Providers:** osteopath | **Results**   - Connor’s Scale (-3 – ‘severe worsening’ to +3 – ‘significant improvement’), p-values not reported  \|  \| **Osteopathic**  **(n=50)** \| **Control (n=27)** \| \| --- \| --- \| --- \| \| **-3** \| 0.4% \| 0% \| \| **-2** \| 0.6% \| 1.1% \| \| **-1** \| 4.4% \| 11.1% \| \| **0** \| 45.1% \| 78.5% \| \| **+1** \| 35.6% \| 9.3% \| \| **+2** \| 12.8% \| 0 \| \| **+3** \| 1.2% \| 0 \|   ***Specific adverse effects:*** not reported |
| Hubmann 2006  Austria  **Focus:** RCT of the effects of osteopathic treatment for children with ADHD  **Duration:** 2 months  **Follow-up:** no post-intervention follow-up  **Quality:** low  **PARTICIPANTS:**  **N:** 30 (% female not reported)  **Age:** 6 to 10 years (details not reported)  **Inclusion:** ADHD, treated with ritalin or other ADHD-specific drugs | **Intervention type:** osteopathy  **Intervention (n=15):** osteopathic treatment; 3 treatments with intervals of 4 weeks  **Comparison (n=15):** no osteopathic treatment  **Dose:** see above  **Providers:** osteopath  **Further information available on:** behavioural details | **Results**   - Connor’s Scale (0 – ‘not at all’ to 3 – ‘very much’); p-values not reported  \|  \| **Osteopathic**  **(n=15)** \| **Control (n=15)** \| \| --- \| --- \| --- \| \| Restless or overactive \| -21.43% \| -8.00% \| \| Excitable, impulsive \| -31.03% \| -7.69% \| \| Disturbs other children \| -13.04% \| +16.67% \| \| Fails to finish things – short attention span \| -32.14% \| -3.57% \| \| Constantly fidgeting \| -14.81% \| 0 \| \| Inattentive, easily distracted \| -31.43% \| -10.00% \| \| Demands must be met immediately, easily frustrated \| -14.29% \| +7.41% \| \| Cries often and easily \| -24.14% \| -4.35% \| \| Mood changes quickly and drastically \| -12.00% \| +13.04% \| \| Temper outburst, explosive and unpredictable behaviour \| -8.00% \| +4.00% \|   ***Specific adverse effects:*** not reported |
| **Cancer care** |  |  |
| Cantarero-Villanueva 2011  Spain  **Focus:** RCT of the effects of a multimodal programme combining core stability exercises and massage – myofascial release in breast cancer survivors  **Duration:** 8 weeks  **Follow-up:** 6 months  **Quality:** high  **PARTICIPANTS:**  **N:** 78 (all female)  **Age:** 48 to 49 years  **Inclusion:** patients 25 to 65 years with a diagnosis of breast cancer, finished coadjuvant treatment except hormone therapy, no active disease, with 4 or 5 of: neck or shoulder pain, reduced range of motion in the neck-shoulder region, reduced physical capacity, psychological problems, increased fatigue, sleep disturbances, any problem in coping with physical or psychosocial functioning; exclusions: chemo- or radiotherapy at the time of study, chronic or orthopaedic diseases not permitting programme | **Intervention type:** physiotherapy  **Intervention:** multi-modal programme: individual physical training (aerobic exercise progression, resistance exercises, neck-shoulder mobility exercises) followed by stretching and massage (myofascial release); after the end of 8 weeks supervised training, participants received an instructional DVD with the me exercise programme  **Comparison:** usual care  **Dose:** intervention: 24 h if individual physical training and 12 h of recovery procedures; 3 times a week for 90 min  **Providers:** not reported | **Results**  Post-intervention (Profile of Mood States)   \| **Change in outcome** \| **Multimodal programme**  **(mean, 95% CI)** \| **Control (mean, 95% CI)** \| **p** \| \| --- \| --- \| --- \| --- \| \| Tension-anxiety \| -9.66  (-13.45, -5.83) \| -0.34  (-2.95, 2.26) \| <0.05 \| \| Depression-dejection \| -7.36  (-11.15, -3.57) \| -0.02  (-2.84, 2.79) \| <0.05 \| \| Anger-hostility \| -7.87  (-12.16, -3.59) \| +1.31  (-2.05, 4.04) \| <0.05 \| \| Vigour \| +5.29  (3.40, 8.29) \| 0.17  (-2.57, 2.22) \| <0.05 \| \| Fatigue \| -8.03  (-11.19, -4.86) \| -1.93  (-5.06, 0.20) \| <0.05 \| \| Confusion \| -4.68  (-7.71, -1.55) \| -1.40  (-4.55, 1.11) \| <0.05 \| \| Total mood disturbance \| 3442.85  (1623.71, 5353.11) \| 491.31  (-905.90, 1608.76) \| <0.05 \|  - Significant improvements in the intervention group compared to control also in the multiple sit-to-stand test and in the trunk curl static endurance test - Differences were maintained at 6 months, but were somewhat smaller   ***Specific adverse events:*** none |
| Fernández-Lao 2012  Spain  **Focus:** RCT of the effects of myofascial massage on mood and fatigue in breast cancer survivors  **Duration:** 2 weeks  **Follow-up:** no post-intervention follow-up  **Quality:** low  **PARTICIPANTS:**  **N:** 20 (all female)  **Age:** 49 years  **Inclusion:** patients 25 to 65 years with a diagnosis of breast cancer, finished coadjuvant treatment except hormone therapy, no active disease, had interest in improving lifestyle, moderate to high fatigue; exclusions: chemo- or radiotherapy at the time of study | **Intervention type:** physiotherapy  **Intervention:** myofascial massage focusing on the shoulder-neck area using the Barnes approach  **Comparison:** usual care plus special attention (advice on improving quality of life after breast cancer)  **Dose:** two 40 min sessions separated by two weeks  **Providers:** physical therapists with >5 years clinical experience in manual therapy and >2 years treating breast cancer survivors | **Results**  Post-intervention (Profile of Mood States)   \| **Change in outcome** \| **Myofascial massage**  **(mean, 95% CI)** \| **Control**  **(mean, 95% CI)** \| **p** \| \| --- \| --- \| --- \| --- \| \| Tension-anxiety \| -7.4  (-11.1, -3.6) \| -3.2  (-5.8, -0.5) \| ? \| \| Depression-dejection \| --1.7  (-3.6, 0.7) \| -0.5  (-2.0, 0.7) \| ? \| \| Anger-hostility \| -3.8  (-6.5, -1.1) \| -1.9  (-5.1, 1.2) \| ? \| \| Vigour \| +1.0  (-2.5, 4.5) \| +2.0  (-4.01, -0.08) \| ? \| \| Fatigue \| -6.1  (-11.2, -5.0) \| -2.4  (-6.3, 2.6) \| ? \| \| Confusion \| -2.5  (-5.0, -0.1) \| -0.8  (-2.4, 0.7) \| ? \| \| Mood disturbance \| 2475.0  (1332.5, 3617.5) \| 920.0  (-11.9, 1851.9) \| ? \|  - Significant improvement seen in the intervention group with respect to parameters of heart rate variability, tension-anxiety, fatigue and mood disturbance but not stated if there was a significant difference to the control group   ***Specific adverse events:*** none |
| López-Sendin 2012  Spain  **Focus:** RCT of the effects of physical therapy (including massage and exercise) on patients with advanced terminal cancer  **Duration:** 2 weeks  **Follow-up:** no post-intervention follow-up  **Quality:** low  **PARTICIPANTS:**  **N:** 24 (25% female)  **Age:** 54 to 55 years  **Inclusion:** oncology patients >18 years, diagnosed with any tumour in stage III-IV, pain >4 on a numerical pain rating scale; exclusions: fragile tissue, systemic status such as neutropenia, hypercalcaemia etc., unconscious, projected to have less than 20 days to live | **Intervention type:** physiotherapy  **Intervention:** therapeutic massage techniques:  effleurage, petrissage, and strain/counterstrain techniques over the tender points; additionally:  passive mobilisation, active-assisted or active-resisted exercises, and local- and global-resisted exercises, as well as proprioceptive neuromuscular facilitation (PNF) applied over joints and tight/painful muscles; risk areas were avoided  **Comparison:** simple hand contact or 'simple touch', placed on areas of pain and maintained for the same period as in the intervention group; the treated areas included the lower cervical area, shoulder, interscapular area, heels, dorsal foot, and gastrocnemius; risk areas were avoided  **Dose:** 6 sessions of 30-35 min over 2 weeks  **Providers:** not reported | **Results**   - a significant group by time interaction with greater improvements in the intervention group was found for Brief Pain Inventory (BPI) worst pain (F = 3.5, p = 0.036), BPI pain right now (F = 3.94, p = 0.027), and BPI index (F = 13.2, p < 0.001), as well as for Memorial Symptom Assessment Scale (MSAS) Psychological (F = 8.480, p = 0.001) - no significant difference between groups for BPI least pain, BPI pain on average, MSAS Physical, Global Distress Index   ***Specific adverse events:*** not reported |
| Pace do Amaral 2012  Brazil  **Focus:** RCT of the effects of manual therapy and upper limb exercises in women with impaired shoulder range of motion after axillary lymph node dissection for breast cancer  **Duration:** 1 month  **Follow-up:** 18 months  **Quality:** medium  **PARTICIPANTS:**  **N:** 131 (all female)  **Age:** 55 to 57 years  **Inclusion:** women who underwent axillay lymph node dissection for breast cancer treatment and had flexion and/or abduction range of motion of ≤100° of the ipsilateral shoulder ; exclusions: bilateral axillary lymph node dissection, immediate reconstruction, previous radiotherapy, bone metastases, muscukloskeletal shoulder dysfunction before surgery, palliative care | **Intervention type:** physiotherapy  **Intervention:** manual therapy after the exercise sessions (see below): mobilisation (scapular and glenohumeral joint) and therapeutic massage. Mobilisation techniques: gliding, oscillation, and traction for the glenohumeral joint; and adduction, abduction, elevation, depression, and internal and external rotation for the scapula. Therapeutic massage (friction maneuvers and deep gliding) was used in the presence of wound adherence or lymphatic cording  **Comparison:** exercise only  **Both groups:** active upper limb exercises (group setting): 19 movements of flexion, extension, abduction, adduction, internal, and external rotation of the upper limb, alone or combined  **Dose:** exercise: about 12 45-min sessions over 1 month; 8 20-min sessions over 1 month  **Providers:** 7 physical therapists received special training for applying the manual therapy protocol | **Results**  At 18 months   \| **Outcome** \| **Manual therapy + exercise**  **(mean, SD)** \| **Exercise only**  **(mean, SD)** \| **p** \| \| --- \| --- \| --- \| --- \| \| Flexion \| 165.9 (19.9) \| 174.4 (11.4) \| NS \| \| Abduction \| 165.7 (13.7) \| 170.9 (12.3) \| NS \| \| Functional score \| 31.1 (5.1) \| 32.3 (5.6) \| NS \|   ***Specific adverse events:*** not reported |
| Wu 2010  Taiwan  **Focus:** prognosis of patients with osteosarcoma who had prior manipulative therapy  **Study design:** prospective controlled cohort study  **Duration:** mean 2.8 weeks  **Follow-up:** mean follow-up 50.2 months in the control group and 41.8 months in the manipulation group  **Quality:** moderate  **PARTICIPANTS:**  **N:** 134 (31% female)  **Age:** 18.2 to 21.5 years (range 5 to 67)  **Inclusion:** osteosarcoma, 2 groups had similar symptom duration (4 months), tumour location, and tumour volume (276 to 285 ml) | **Intervention type:** various  **Intervention:** providers: bone-setters (51%), Chinese medical practitioners (46%), physiotherapists (3%)  **Comparison:** no manipulation  **Dose:** 2.6 manipulative sessions over mean of 2.8 weeks  **Providers:** see above  **Further information available on:** demographic details, co-interventions | \|  \| **Manipulative therapy** \| **No manipulative therapy** \| **p** \| \| --- \| --- \| --- \| --- \| \| Skip lesions \| 11% \| 0 \| 0.005 \| \| Primary lung metastasis \| 32% \| 3% \| 0.003 \| \| Lung metastasis rate \| 51.4% \| 18.8% \| <0.001 \| \| Local recurrence \| 29% \| 6% \| 0.001 \| \| 5-year survival rate \| 58% \| 92% \| 0.004 \| |
| **Cerebral palsy** |  |  |
| Duncan 2004  USA  **Focus:** RCT of osteopathic manipulation or acupuncture as an adjunct to therapy for children with spastic cerebral palsy  **Duration:** 6 months  **Follow-up:** no post-intervention follow-up  **Quality:** low  **PARTICIPANTS:**  **N:** 50 (24% female)  **Age:** 11 months to 12 years  **Inclusion:** children with cerebral palsy; Gross Motor Functional Classification System 22% classified level I (mildest disturbance), 58% levels IV or V (most severe disturbance) | **Intervention type:** osteopathy  **Intervention 1 (n=23):** osteopathy: cranio-sacral and myofascial release techniques  **Intervention 2 (n=19):** acupuncture: combination of scalp, body and auricular acupuncture  **Intervention 3 (n=8):** combination of osteopathy and acupuncture  **Comparison (n=19):** non-therapeutic time with a volunteer  **Dose:** unclear  **Providers:** acupuncture: Traditional Chinese Practitioner; osteopathy: osteopathic physician  **Further information available on:** anatomical lesions / restrictions | **Results**   - No statistical evaluations reported, all results based on parents’ reports - Only 2 of 17 parents in control arm reported any improvement, compared with 21 of 23 parents in the osteopathic arm, all of the parents in the control arm, and all of the parents in the combination arm (parents presumably not blinded)  \| **Improvement in…** \| **Osteopathic**  **(n=23)** \| **Acupuncture (n=19)** \| **Control (n=17)** \| \| --- \| --- \| --- \| --- \| \| Leg or hand use \| 61% \| 68% \| 0 \| \| Sleep \| 39% \| 53% \| 0 \| \| Improved mood \| 30% \| 32% \| 12% \| \| Worsened mood \|  \|  \| 29% \| \| Speech or drooling \| 4% \| 37% \| 6% \| \| Bowel movements \| 26% \| 21% \| 0 \| \| Cognition \| 4% \| 21% \| 0 \| \| VAS muscle stiffness reduced >10 \| 43% \| 61% \| 39% \| \| VAS happiness increased >10 \| 38% \| 17% \| 22% \|   ***Specific adverse effects:*** not reported |
| Duncan 2008  USA  **Focus:** RCT of osteopathic manipulation or acupuncture as an adjunct to therapy for children with moderate to severe spastic cerebral palsy  **Duration:** 6 months  **Follow-up:** no post-intervention follow-up  **Quality:** medium  **PARTICIPANTS:**  **N:** 55 (24% female)  **Age:** 20 months to 12 years  **Inclusion:** children with moderate to severe spastic cerebral palsy; Gross Motor Functional Classification System (GMFCS) 20% classified level I (mildest disturbance), 62% levels IV or V (most severe disturbance) | **Intervention type:** osteopathy  **Intervention 1 (n=26):** osteopathy: use of direct or indirect techniques in the cranial field, myofascial release, or both; 10 sessions of 1 h over 24 weeks (once weekly weeks 1-4, once biweekly weeks 5-8, once monthly weeks 9 to 24)  **Intervention 2 (n=27):** acupuncture: combination of scalp, body and auricular acupuncture; 30 sessions of 30 min over 24 weeks (three times a week weeks 1-4, twice a week weeks 5-8, once a week weeks 9-12, once biweekly weeks 13-24)  **Comparison (n=22):** 11 h of non-specific non-therapeutic play time  **Dose:** see above  **Providers:** acupuncture: Traditional Chinese Practitioner; osteopathy: osteopathic physician  **Further information available on:** modified Ashworth Scale biceps and hamstring, parent / guardian rating of arched back, parent / guardian rating of startle reflex | **Results**   \|  \| **Osteopathic** \| **Acupuncture** \| **Control** \| **p** \| \| --- \| --- \| --- \| --- \| --- \| \| GMFCS \| 3.4 SD1.8 \| 3.2 SD1.4 \| 4.2 SD1.3 \| NS \| \| GMFM percent \| 58.0 SD32.3 \| 50.9 SD37.9 \| 33.5 SD31.2 \| p<0.05 for OMT \| \| PEDI mobility \| 28.7 SD21.0 \| 27.7 SD22.3 \| 18.6 SD20.2 \| NS \| \| PEDI self-care \| 31.7 SD26.5 \| 30.8 SD23.1 \| 19.5 SD20.4 \| NS \| \| WeeFIM mobility \| 15.9 SD10.1 \| 14.6 SD11.2 \| 10.7 SD9.3 \| p<0.05 for OMT \| \| WeeFIM self-care \| 24.3 SD18.5 \| 22.2 SD17.6 \| 16.3 SD15.1 \| NS \| \| Doctor rating of spasticity \| 48.8 SD25.7 \| 57.1 SD24.8 \| 69.5 SD21.6 \| NS \|   GMFM: Gross Motor Function Measurement; PEDI: Paediatric Evaluation Disability Inventory; WeeFIM: Functional Independence Measure for Children  ***Specific adverse effects:*** not reported |
| Wyatt 2011  UK  **Focus:** RCT of cranial osteopathy in children cerebral palsy  **Duration:** 6 months  **Follow-up:** no post-intervention follow-up  **Quality:** medium  **PARTICIPANTS:**  **N:** 142 (42% female)  **Age:** 7.8 years (5 to 12)  **Inclusion:** children aged 5 to 12 with varying levels of function (categories II to V of the Gross Motor Function Classification System) | **Intervention type:** osteopathy  **Intervention (n=71):** cranial osteopathy; 6 sessions (3 in the first 10 weeks, remaining sessions within 6 months; average length of session 21 mins); each child was assigned to 1 of 37 osteopaths who planned the course of therapy according to child’s individual needs  **Comparison (n=71):** partial attention waiting list (parents taking part in 2 semistructured interviews)  **Dose:** see above  **Providers:** osteopaths  **Further information available on:** modified Ashworth Scale biceps and hamstring, parent / guardian rating of arched back, parent / guardian rating of startle reflex | **Results**   - No significant difference between groups after 6 months for gross motor function (GMFM-66) or child quality of life (CHQ) - No significant difference between groups after 6 months for time to sleep, time spent asleep, parental assessment of child’s pain, main carer’s quality of life - Significantly more parents in the intervention group rated their child’s global health as ‘better’ after six months than in the control group (38% versus 18%,p<0.05)   ***Specific adverse effects:*** no serious side effects occurred |
| **Cervicogenic dizziness / balance** |  |  |
| Hawk 2009  USA  **Focus:** pilot RCT to compare the effect of a limited and extended course of chiropractic care on balance, chronic pain, and associated dizziness in a sample of older adults with impaired balance  **Duration:** 8 weeks to 12 months  **Follow-up:** 12 months  **Quality:** low  **PARTICIPANTS:**  **N:** 34 (59% female)  **Age:** 80 years (65 to 93)  **Inclusion:** ≥ 65 years, able to stand steadily without assistance on one leg for <5 seconds (averaging time for both legs), indicating increased risk of falls | **Intervention type:** chiropractic  **Intervention 1 (n=13):** chiropractic care for 8 weeks with 2 visits per week (limited schedule); spinal manipulative therapy using diversified technique (incl. extravertebral manipulation to the hip, knee, ankle and foot; soft tissue treatments such as massage and trigger point therapy; hot packs)  **Intervention 2 (n=15):** chiropractic care for 8 weeks with 2 visits per week, followed by 10 months with one visit per month (extended schedule)  **Comparison (n=6):** instructed on doing home exercises  **All groups:** lifestyle advice (brochure with health recommendations, home hazard checklist, pamphlet on balance exercises)  **Dose:** see above  **Providers:** chiropractors  **Further information available on:** demographic details | **Results (after 12 months)**   - Unequal reporting of falls as patients were asked at each treatment / assessment visit and there were unequal numbers of visits between groups: 6 patients with falls in intervention 1, 9 in intervention 2, none in the comparison group) - No significant difference between groups in scores on Berg Balance Scale, depression, Pain Disability Index, dizziness   ***Specific adverse effects:*** 3 patients reported minor treatment-related effects (lightheadedness, stiffness, joint popping sound) but none lasted longer than 24 h |
| **Chronic pelvic pain** |  |  |
| FitzGerald 2009 / 2013  USA  **Focus:** determining the feasibility of an RCT to compare myofascial physical therapy and global therapeutic massage  **Duration:** 10 weeks  **Follow-up:** 12 weeks  **Quality:** medium  **PARTICIPANTS:**  **N:** 47 (51% female)  **Age:** 43 SD13 years  **Inclusion:** adults with a clinical diagnosis of interstitial cystitis / painful bladder syndrome (IC/PBS, men and women) and chronic prostatitis / chronic pelvic pain (CP/CPPS, men), pain / discomfort in the pelvic region for at least 3 months in the last 6 months, current symptoms present for <3 years | **Intervention type:** physiotherapy  **Intervention (n=23):** myofascial physical therapy; connective tissue manipulation, manual trigger point release techniques; home exercises offered  **Comparison (n=24):** general massage therapy: full body Western massage  **Dose:** 10 weekly treatments lasting of 1 h each  **Providers:** physical therapists, massage therapists  **Further information available on:** details of adverse events, demographic details, details of global response assessment | **Results**   - Global response assessment (GRA, “Compared to before therapy, how would you rate you symptoms?”: 1 – ‘markedly worse’ to 7 ‘markedly improved’); responders: scores 6 and 7, rest nonresponders - IC symptom and problem index (ICSI, ICPI), sexual function index (FSFI, gender-specific), quality of life (SF-12)  \|  \| **Myofascial therapy** \| **Massage** \| **p** \| \| --- \| --- \| --- \| --- \| \| **GRA responders** \| 57% \| 21% \| 0.03 \| \| **GRA responders IC/PBS** \| 50% \| 7% \| 0.03 \| \| **GRA responders CP/CPPS** \| 64% \| 40% \| NS \| \| **Pain (0-10)** \| -2.5 \| -0.9 \| NS \| \| **Urinary urgency** \| -2.7 \| -0.8 \| NS \| \| **Urinary frequency** \| -3.6 \| -1.2 \| NS \| \| **ICSI** \| -4.6 \| 0 \| 0.01 \| \| **ICPI** \| -4.7 \| -1.3 \| 0.04 \| \| **FSFI** \| +5.0 \| +1.4 \| NS \| \| **SF-12 physical** \| +1.3 \| -4.4 \| NS \| \| **SF-12 mental** \| +6.2 \| +1.8 \| NS \|   ***Specific adverse effects:*** adverse events reported by 5 patients in the massage group and 12 patients in the myofascial therapy group, pain was most commonly reported, adverse events mostly mild |
| Heyman 2006  Sweden  **Focus:** RCT of the effects of distension of painful pelvic structures for chronic pelvic pain in women  **Duration:** 2 to 3 weeks  **Follow-up:** no post-intervention follow-up  **Quality:** low  **PARTICIPANTS:**  **N:** 50 women  **Age:** median 33 years (range 19 to 54)  **Inclusion:** >19 years, women with chronic pelvic pain of at least 6 months’ duration with continuous or intermittent pain at least 2 days per week | **Intervention type:** physiotherapy  **Intervention (n=10):** treatment procedure: patient lay in a prone position and the physician placed his index finger deep in the patient’s rectum and previously identified painful structures were treated as follows in the given order: At a point two fingerwidths lateral of the sacrum, the physician used his index finger to exert strong pressure against the sacrotuberous/spinal ligaments for 15 s to elicit pain. Thereafter, the musculature of the pelvic floor and the joint between the coccyx and sacrum were concurrently forcefully distended dorsally for 60 s using the index finger. This procedure was repeated after 2 to 3 weeks  **Comparison (n=10):** counselling  **Dose:** see above  **Providers:** physicians | **Results**  VAS symptom scales ( 0 – no complaints, 100 – worst complaints)   \|  \| **Intervention** \| **Control** \| **p** \| \| --- \| --- \| --- \| --- \| \| Pelvic pain \| -35 SD31 \| +0.8 SD9.2 \| 0.001 \| \| Painful intercourse \| -19 SD38 \| +0.13 SD10.7 \| 0.035 \| \| Low back pain \| -21 SD39 \| +5 SD32.2 \| 0.018 \| \| Sleep disturbance \| -6 SD21 \| +11 SD25.2 \| 0.019 \| \| Quality of sleep \| -11 SD23 \| +4.0 SD21.7 \| 0.029 \| \| Mental fatigue \| -11 SD27 \| +15.2 SD25 \| 0.001 \| \| Depression \| -11 SD18 \| -0.8 SD17.7 \| NS \| \| Mood \| -9 SD22 \| +2.1 SD25.6 \| NS \| \| Anger \| -10 SD23 \| -5.9 SD27.9 \| 0.05 \|   ***Specific adverse effects:*** not reported |
| Marx 2009  Germany  **Focus:** RCT of the effects of osteopathic treatment in men with chronic prostatitis / chronic pelvic pain syndrome  **Duration:** 8 weeks  **Follow-up:** 6 weeks after the end of therapy, 1.5 years for intervention patients only  **Quality:** low  **PARTICIPANTS:**  **N:** 35 men  **Age:** 47 years (range 29 to 70)  **Inclusion:** men with chronic prostatitis / chronic pelvic pain syndrome, significant symptoms without significant urological abnormalities (no sonographic abnormalities, prostate size <45 cm^3^, negative bacteriology of urine or ejaculate, PSA <4 µg/L, residual urine <100 ml) | **Intervention type:** osteopathy  **Intervention (n=20):** osteopathic care; osteopathic examination and treatment at the therapist’s discretion (could include manipulation, muscle energy techniques, myofascial techniques, visceral and cranial techniques, “balanced ligamentous tension”); 5 treatments of 45 mins, weekly treatments in the first 3 weeks, then after 2 weeks and another 3 weeks  **Comparison (n=15):** simple exercise programme (warming up, pelvic floor exercises, breathing exercises)  **Dose:** 6 weekly treatments lasting up to 45 mins  **Providers:** osteopaths | **Results**   - Outcomes: International Prostate Symptom Score (IPSS 0 to 35), Chronic Prostatitis Symptom Index (NIH-CPSI, 0 to 43), quality of life (0 to 6) (scores are for least to worst symptoms)   6 weeks after the last treatment:   \|  \| **Intervention** \| **Control** \| **p** \| \| --- \| --- \| --- \| --- \| \| **IPPS** \| -9.50 \| +0.54 \| <0.0005 \| \| **NIH-CPSI** \| -15.65 \| +1.23 \| <0.0005 \| \| **QoL** \| -2.65 \| +0.16 \| <0.0005 \|   ***Specific adverse effects:*** no serious adverse effects seen (some reported tiredness on the day of the treatment) |
| **Cystic fibrosis** |  |  |
| Sandsund 2011  UK  **Focus:** RCT of response of patients with cystic fibrosis to physiotherapy musculoskeletal techniques (designed as exploratory pilot study)  **Duration:** 6 weeks  **Follow-up:** 12 weeks  **Quality:** medium  **PARTICIPANTS:**  **N:** 20 (50% female)  **Age:** median age 27 years  **Inclusion:** adults with cystic fibrosis; reported awareness of postural changes including stiffness, discomfort and/or pain of musculoskeletal origin in the thoracic spine or chest wall; stable clinical state | **Intervention type:** physiotherapy  **Intervention (n=10):** usual care plus musculoskeletal treatments: specific mobilisations to the rib cage and thoracic spine; treatment of specific muscle dysfunction or tight muscle groups; and postural awareness, education and advice based on the principles of the Alexander technique  **Comparison (n=10):** usual care  **Dose:** 6 weekly treatments lasting up to 45 mins  **Providers:** not reported  **Further information available on:** anatomical lesions / restrictions | **Results**   - No significant difference between groups after the end of the study in changes from baseline for pain (VAS), FEV1, thoracic index, modified shuttle test, chest wall excursion - Quality of life (Cystic Fibrosis Quality of Life questionnaire) significantly more increased in the intervention group than in the control group at 12 weeks (p=0.002)   ***Specific adverse effects:*** no adverse effects seen |
| **Dysfunctional voiding** |  |  |
| Nemett 2008  USA  **Focus:** RCT of effect of manual physical therapy based on an osteopathic approach added to standard therapy on dysfunctional voiding in children  **Duration:** 10 weeks  **Follow-up:** ≥3 months  **Quality:** low  **PARTICIPANTS:**  **N:** 21 (67% female)  **Age:** 6.8 years SD 2.2  **Inclusion:** children with post-void residuals (PVR), daytime urinary incontinence (DI), recurrent urinary tract infections (UTI), dyssynergic voiding (DYS) or vesicoureteal reflux (VUR); 41% had VUR, 64% had DI, 9% had both VUR and DI, 59% had recurrent UTIs, 77% had DYS | **Intervention type:** osteopathy  **Intervention (n=10):** manual physical therapy based on an osteopathic approach (MPT-OA), customised to each child, included gentle mobilisation of body tissues to relieve movement restrictions, and thereby achieve balanced alignment and mobility and postural symmetry, with particular attention to the thoracolumbar spine, thoracic and pelvic diaphragms, pelvis, pelvic organs, and lower extremities; plus standard therapy as below  **Comparison (n=11):** standard care as appropriate (could include medications, establishment of timed voiding and evacuation schedules, dietary modifications, behavior modification, pelvic floor muscle retraining, biofeedback training, and treatment of constipation)  **Dose:** standard treatment: four clinic appointments lasting 1 h at 2-week intervals; osteopathy: four 1 h treatment sessions coinciding with clinic appointments  **Providers:** not reported  **Further information available on:** anatomical lesions / restrictions | **Primary**  ***Proportion of outcomes improved (of VUR, days wet, PVR, UTI, DYS) by diagnosis:***   \| **Diagnosis** \| **MPT-OA** \| **Control** \| **p** \| \| --- \| --- \| --- \| --- \| \| all together \| 60% \| 31% \| 0.008 \| \| VUR (no DI) \| 62.5% \| 33.3% \| NS \| \| DI (no VUR) \| 58.3% \| 31.8% \| 0.065 \| \| VUR and DI \| - \| 25% \| - \|   NR=not reported  ***Specific adverse effects:*** not reported |
| **Menopausal symptoms** |  |  |
| Cleary 1994  UK  **Focus:** RCT of the effects of “Fox’s low force osteopathic techniques” on menopausal symptoms  **Duration:** 10 weeks  **Follow-up:** 5 weeks post-intervention  **Quality:** low  **PARTICIPANTS:**  **N:** 30 women  **Age:** 51.3 SD13.1 to 53.9 SD10.1 years  **Inclusion:** women aged 50 to 60 years who had menstruated less than 4 times in the previous 12 months; exclusions: hormone replacement therapy | **Intervention type:** osteopathy  **Intervention (n=15):** Fox’s low force technique: spine, cranium and pelvis examined for areas of joint strain; treatment of spine and pelvis in the following manner: a finger or thumb was used to deliver the low-force to the spinous process in a direction thought to relieve the restriction, relaxing the joint’s protective mechanism, via the muscle spindle, by increasing the resting length of the muscle, thereby improving mobility; the ‘force’ required to relax the muscle is so low that it does not extend to adjacent joints or surrounding tissues; patients are not required to assist the practitioner by adopting a particular position, or use their own muscle power; also use of cranial techniques  **Comparison (n=15):** placebo: employing the same method, but with the force delivered to a joint adjacent to a restricted joint, where it will have no effect  **Dose:** 30 min once a week for 10 consecutive weeks  **Providers:** osteopaths  **Further information available on:** hormone levels | **Results**   - *Menopausal symptoms (questionnaire):* after the intervention, significant reduction in hot flushes, night sweats, urinary frequency, and depression compared to control, but not insomnia and irritability; at 5 weeks post-intervention, difference remained significant for hot flushes and night sweats and became significant for insomnia - *Back and neck pain:* at the 5 week follow-up, reduction in neck pain was significantly greater for the intervention group (p=0.04) (n=8 and n=6 with neck pain in intervention and control groups respectively), and nearly so for back pain (p=0.06) (n=8 and n=4 with back pain in intervention and control groups respectively)   ***Specific adverse effects:*** not reported |
| **Gastrointestinal disorders** |  |  |
| Florance 2012  France  **Focus:** RCT of osteopathic treatment effects compared to standard therapy in adults with irritable bowel syndrome  **Duration:** 1 week  **Follow-up:** 4 weeks  **Quality:** low  **PARTICIPANTS:**  **N:** 30 (77% female)  **Age:** 45.8 years  **Inclusion:** adults with diagnosis of irritable bowel syndrome (Rome III criteria); symptoms of irritable bowel syndrome had to be present more than 25% of the time (evaluated by diary) | **Intervention type:** osteopathy  **Intervention (n=20):** osteopathy using direct and indirect techniques on the spine and abdomen  **Comparison (n=10):** sham treatment (gentle massage involving spine and abdomen)  **Dose:** 2 sessions with a 1 week interval; 60 min sessions  **Providers:** an osteopath | **Results**  At 28 days   \| **Change in outcome** \| **Osteopathy**  **(mean, SD)** \| **Sham**  **(mean, SD)** \| **p-value** \| \| --- \| --- \| --- \| --- \| \| Irritable bowel syndrome severity score \| -22.9 (34.3) \| -18.9 (24.4) \| NS \| \| Impact of abdominal pain severity on quality of life \| -6.6 (50.8) \| -2.5 (41.0) \| NS \| \| Fatigue impact scale \| -7.4 (62.3) \| -19.0 (44.6) \| NS \| \| Hospital Anxiety and Depression Scale \| 1.1 (18.7) \| 1.8 (23.1) \| NS \| \| Beck Depression Inventory \| -23.8 (61.3) \| -36.5 (65.5) \| NS \| \| Satisfied with treatment \| 70% \| 40% \| 0.03 \|  - After the intervention (at 7 days), results for irritable bowel syndrome severity and impact of abdominal pain severity on quality of life were significantly better for the osteopathy group than for the sham group   ***Specific adverse effects:*** procedures well tolerated, no adverse effects |
| Hundscheid 2006  The Netherlands  **Focus:** RCT of osteopathic treatment effects compared to standard therapy in adults with irritable bowel syndrome  **Duration:** 6 months  **Follow-up:** 6 months  **Quality:** low  **PARTICIPANTS:**  **N:** 39 (59% female)  **Age:** 44 years  **Inclusion:** adults with diagnosis of irritable bowel syndrome (Rome II criteria) with abdominal complaints (moderate severity) of at least 3 days of the week prior to trial entry. Patients with somatic pathology or conditions explaining abdominal complaints were excluded | **Intervention type:** osteopathy  **Intervention (n=20):** osteopathy using individual black box method; 5 sessions once per 2-3 weeks for 6 months; no use of medications  **Comparison (n=19):** standard care of 6 months consisted of fibre rich diet; in cases of constipation and diarrhoea, laxative and loperamide were added respectively; in case of cramps, mebeverine was prescribed  **Dose:** see above  **Providers:** an osteopath | **Results**   \| **Change in outcome** \| **Osteopathy** \| **Control** \| **p-value** \| \| --- \| --- \| --- \| --- \| \| Overall assessment \| 68% \| 18% \| <0.006 \| \| FBDSI score \| 100 \| 52 \| 0.02 \| \| Quality of life \| 18 \| 12 \| <0.05 \| \| Symptom score [endpoint] \| 6.8 \| 10 \| 0.02 \|   ***Specific adverse effects:*** not observed |
| **Hypertension** |  |  |
| Cerritelli 2011  Italy  **Focus:** effects of osteopathic manipulative treatment on hypertension  **Study design:** CCT  **Duration:** 12 months  **Follow-up:** no post-intervention follow-up  **Quality:** medium  **PARTICIPANTS:**  **N:** 63 (51% female)  **Age:** 50 SD6 years  **Inclusion:** grade 1+ hypertension and vascular abnormalities (B-ultrasound morphology classified as II, III, IV) | **Intervention type:** osteopathy  **Intervention (n=31):** osteopathic manipulative treatment (OMT) plus standard pharmacological therapy (calcium channel blockers, ACE-inhibitors, beta-blockers, diuretics, combination); OMT techniques: fascial, cranial and balanced ligamentous techniques  **Comparison (n=32):** standard pharmacological therapy only  **Dose:** OMT treatment every 2 weeks  **Providers:** osteopath  **Further information available on:** blood lipids, endothelial parameters | **Results (12 months)**   \|  \| **OMT** \| **Control** \| **p** \| \| --- \| --- \| --- \| --- \| \| **Systolic BP (mmHg)** \| -26.48 SD3.71 \| -21.69 SD2.57 \| <0.0001 \| \| **Diastolic BP** \| -11.65 SD3.84 \| -9.16 SD2.41 \| 0.003 \| \| **Intima media thickness (carotid / femoral bifurcations)** \| -0.53 SD0.30 \| -0.00 SD0.10 \| <0.001 \|  - After adjustment for BMI and baseline systolic blood pressure, OMT was significantly related to decreases in intima media thickness and systolic blood pressure, but not diastolic blood pressure   ***Specific adverse effects:*** not reported |
| **Peripheral arterial disease** |  |  |
| Ramos-González 2012  Spain  **Focus:** RCT of the effectiveness of myofascial release manual therapy on venous insufficiency in postmenopausal women  **Duration:** 10 weeks  **Follow-up:** no post-intervention follow-up  **Quality:** medium  **PARTICIPANTS:**  **N:** 65 (all postmenopausal women)  **Age:** 62.2 to 65.8 years  **Inclusion:** 40 to 75 years and the presence of stage I or II chronic venous insufficiency; exclusion: more advanced stage of venous insufficiency, uncompensated cardiorespiratory insufficiency, recent venous thrombosis | **Intervention type:** physiotherapy  **Intervention (n=33):** myofascial release (longitudinal sliding in cranial direction in all compartments (anterior, posterior, external lateral and internal lateral sides of the thigh); hands crossed on external lateral and anterior compartment of the thigh; and myofascial release of the triceps surae fascia) plus kinesiotherapy  **Comparison (n=32):** kinesiotherapy only  **Both groups:**  kinesiotherapy: flexion and extension of metacarpophalangeal and interphalangeal articulations while seated; flexion and extension of the tibioperoneal joint while standing; isometric contractions of both quadriceps with knees extended while seated; and a final period of abdomino-diaphragmatic breathing, lengthening inhalations while standing and lengthening exhalations in supine position.  **Dose:** instructions to perform kinesiotherapy exercises at home twice a day for 10 weeks; myofascial therapy: 2 sessions per week for 10 weeks (50 min sessions)  **Providers:** experienced physiotherapist | **Results**  At 10 weeks   - There was a greater reduction versus baseline in basal metabolism (p<0.039) and intracellular water levels (p<0.046) in the myofascial release group than in the control group - The myofascial release group showed significant improvements in diastolic blood pressure versus control (p<0.046), venous blood flow velocity versus control (p<0.048) and VAS-assessed pain versus controls (p<0.039) - There were significant differences between the groups on the following SF-36 dimensions: physical function (F=4.55; p<0.044) body pain (F=4.21; p<0.040) and emotional role (F=4.70; p<0.047)   ***Specific adverse effects:*** not reported |
| Lombardini 2009  Italy  **Focus:** effects of osteopathic manipulative treatment in combination with lifestyle modification and pharmacological therapy in patients with intermittent claudication  **Study design:** CCT  **Duration:** 6 months  **Follow-up:** no post-intervention follow-up  **Quality:** medium  **PARTICIPANTS:**  **N:** 30 men  **Age:** 69 SD8 years  **Inclusion:** Fontaine stage II monolateral intermittent claudication, male, clinical onset of peripheral arterial disease less than 1 year, low compliance with physical training programme, ankle/brachial pressure index <0.90 at rest, stable maximum walking time of 170-250 s during standard treadmill test | **Intervention type:** osteopathy  **Intervention (n=15):** osteopathic manipulative treatment (OMT) plus standard pharmacological therapy; OMT techniques: myofascial release, strain/counterstrain, muscle energy, soft tissue techniques, high velocity low amplitude (thoracolumbar region), lymphatic pump, craniosacral manipulation; 30 min sessions  **Comparison (n=15):** standard pharmacological therapy only  **Dose:** months 1 and 2: one OMT session every 2 weeks, month 3: assessment of response and adjustment of OMT techniques if necessary, months 4 to 6: one OMT session every 3 weeks  **Providers:** osteopath  **Further information available on:** blood lipids, endothelial parameters | **Results (6 months)**   \|  \| **OMT** \| **Control** \| **p** \| \| --- \| --- \| --- \| --- \| \| **ABPI rest** \| 0.87 SD0.05 \| 0.78 SD0.05 \| OMT <0.05 vs BL \| \| **ABPI exercise** \| 0.79 SD0.06 \| 0.57 SD0.04 \| OMT <0.05 vs BL \| \| **CPT (min)** \| 3.7 SD0.4 \| 2.9 SD0.3 \| OMT <0.05 vs BL \| \| **TWT (min)** \| 4.7 SD0.4 \| 4.5 SD0.8 \| OMT <0.05 vs BL \| \| **Physical function** \| 72.8 SD3.7 \| 37.5 SD4.7 \| <0.05 \| \| **Role limitations / physical** \| 60.5 SD22.6 \| 29.3 SD16.5 \| <0.05 \| \| **Bodily pain** \| 86.5 SD19.7 \| 66.5 SD15.8 \| <0.05 \| \| **General health** \| 67.8 SD7.6 \| 53.2 SD12.0 \| <0.05 \| \| **Mental health** \| 75.9 SD9.6 \| 73.5 SD11.3 \| NS \| \| **Role limitations / emotional** \| 86.4 SD8.7 \| 83.5 SD11.0 \| NS \| \| **Social function** \| 82.7 SD10.4 \| 79.0 SD8.5 \| NS \| \| **Vitality** \| 65.7 SD10.2 \| 60.8 SD10.6 \| NS \|   ABPI: ankle-brachial pressure index, BL: baseline; CPT: claudication time pain, TWT: total walking time  ***Specific adverse effects:*** transient muscle tenderness in 3 patients |
| **Parkinson’s disease** |  |  |
| Wells 1999  USA  **Focus:** effect of osteopathic manipulative treatment on gait in patients with Parkinson’s disease  **Study design:** CCT  **Duration:** single session  **Follow-up:** immediately after treatment  **Quality:** low  **PARTICIPANTS:**  **N:** 20 (% female not reported)  **Age:** 45 to 68 years  **Inclusion:** Parkinson’s disease (mild to moderate; Unified Parkinson’s Rating Scale motor score average 14.3) | **Intervention type:** osteopathy  **Intervention (n=10):** 30 minute protocol of osteopathic manual therapy (1. Lateral (and anteroposterior) translation of vertebrae in the thoracic/lumbar spine performed with the patient in a seated position; 2. Active myofascial stretch to the thoracic spine with the patient in a seated position; 3. Occipito-atlanto (OA) release; 4. Translation of cervical spine performed with the patient in a supine position; 5. Muscle energy techniques of the cervical spine; 6. Spencer technique applied to the shoulder bilaterally; 7. Supination/pronation of the forearm bilaterally; 8. Circumduction of the wrist bilaterally; 9. Sacroiliac joint gapping bilaterally; 10. Muscle energy technique applied to adductor muscles of lower extremity bilaterally; 11. Psoas muscle energy technique applied bilaterally; 12. Hamstring muscle energy technique applied bilaterally; 13. Articulatory technique applied to the ankle bilaterally; and 14. Muscle energy technique applied to the ankle in dorsi and plantar flexion bilaterally)  **Comparison (n=10):** sham procedure (examination of the patient’s voluntary range of motion in each joint to which manipulation would have been applied without the manipulation procedure, some passive motion of limbs without reaching patient’s range of motion limit)  **Dose:** single 30 min session  **Providers:** student physician with special training in osteopathic manipulative technique under the direction of an osteopathic physician | ***Gait parameters***   - significant improvement in the following gait parameters in comparison to control: stride length difference, cadence difference, upper limb velocities (shoulder, wrist), lower limb velocities (hip, knee, ankle)   ***Specific adverse effects:*** not reported |
| **Pneumonia and other respiratory diseases** |  |  |
| Noll 2008b  USA  **Focus:** RCT of the effects of osteopathic manipulative treatment in elderly patients with chronic obstructive pulmonary disease (COPD)  **Duration:** single session  **Follow-up:** 1 day after the intervention  **Quality:** medium  **PARTICIPANTS:**  **N:** 35 (49% women)  **Age:** 69.6 SD6.6 to 72.2 SD7.1 years  **Inclusion:** known history of COPD, ≥65 years, airflow obstruction | **Intervention type:** osteopathy  **Intervention (n=18):** osteopathic manipulative medicine protocol (massage of paraspinal muscles, rib raising, doming the abdominal diaphragm, suboccipital decompression, myofascial release to the thoracic inlet, pectoral traction, thoracic lymphatic pump with activation)  **Comparison (n=17):** sham light touch protocol  **Dose:** single 20 min session  **Providers:** osteopaths  **Further information available on:** 21 lung function parameters | **Results**   - *Absolute pulmonary function parameters:* statistically significant differences in 8 of 21 lung function parameters in the OMT group compared to control (forced expiratory flow after 25% and 50% of FEV had been exhaled (FEF_25%_, FEF_50%_) , forced expiratory flow at the midexpiratory phase (FEF_25% 75%_) and expiratory reserve volume (ERV) significantly lower and lung volume parameters significantly higher, airway resistance decreased) - *Percent change in lung function parameters from baseline to post-treatment:* FEF_50%_ and FEF_25% 75%_ significantly lower, lung volume parameters significantly higher - Patients in both groups felt that they had benefitted from the manipulative treatment, that they breathed better, enjoyed the treatment and would recommend it to others (71 to 94% in the intervention group, 59 to 82% in the sham group)   ***Specific adverse effects:*** only minor adverse events, no difference between groups (n=2 intervention, n=4 control) |
| Zanotti 2012  Italy  **Focus:** RCT of the effects of osteopathic manipulative treatment in patients with severe chronic obstructive pulmonary disease (COPD)  **Duration:** 4 weeks  **Follow-up:** no post-intervention follow-up  **Quality:** high  **PARTICIPANTS:**  **N:** 20 (25% women)  **Age:** 64 years  **Inclusion:** patients with stable stage III COPD (no exacerbations in past 3 months) | **Intervention type:** osteopathy  **Intervention (n=10):** osteopathic manipulative treatment plus pulmonary rehabilitation programme  **Comparison (n=10):** pulmonary rehabilitation programme only  **Both groups:** pulmonary rehabilitation programme: exercise training, educational support, psychological counselling and nutritional intervention  **Dose:** pulmonary rehabilitation: 5 days a week for 4 weeks, 30 min sessions; osteopathic treatment: once a week for 4 weeks, 45 min sessions  **Providers:** osteopaths | **Results**  At 4 weeks   \| **Change in outcome** \| **Osteopathy**  **(mean, 95% CI)** \| **Control**  **(mean, 95% CI)** \| **p-value** \| \| --- \| --- \| --- \| --- \| \| Vital capacity (l) \| +0.11 (-0.15, 0.37) \| +0.02 (-0.19, 0.23) \| NS \| \| FEV1 (l) \| +0.14 (0, 0.26) \| +0.01 (-0.12, 0.14) \| NS \| \| Forced vital capacity (l) \| +0.09 (-0.49, 0.33) \| +0.04 (-0.07, 0.15) \| NS \| \| Residual volume (l) \| -0.5 (-1, 0) \| -0.06 (-0.11, 0.01) \| 0.001 \| \| 6 min walk test (m) \| +72.5 (33.9, 111.1) \| +23.7 (-3.5, 50.9) \| 0.04 \|   ***Specific adverse effects:*** no adverse events or side effects; both treatments well tolerated |
| **Pregnancy / obstetric care / neonatal care** |  |  |
| Cameron 2005  UK  **Focus:** RCT of manual physical therapy (PT) effects compared to no PT in preterm infants with very low birth weight (VLBW)  **Duration:** 4 months  **Follow-up:** 4 months  **Quality:** medium  **PARTICIPANTS:**  **N:** 60 (40% female)  **Age:** 29 weeks [gestational age]  **Inclusion:** infants with 24 weeks <gestational age < 32 weeks and birth weight < 1500 g; exclusions were cortical blindness or retinopathy causing blindness, musculoskeletal/congenital abnormality, oxygen dependency, severe hydrocephalus, signs of drug withdrawal, or family history of social problems. | **Intervention type:** physiotherapy  **Intervention (n=34):** neonatal developmental PT consisting of handling, positioning techniques to promote symmetry, muscle balance, and movement using postural support and facilitation techniques  **Comparison (n=38):** no PT  **Dose:** each session of 60 minutes (PT) daily on weekdays  **Providers:** paediatric physical therapists | **Results**   \| **Change in outcome** \| **Physical therapy (interquartile range)** \| **No physical therapy (interquartile range)** \| **p-value** \| \| --- \| --- \| --- \| --- \| \| 4-month median percentile rank on the AMIS \| 65.0  (42.0) \| 72.5  (32.5) \| NS \|   ***Specific adverse effects:*** not reported |
| Peterson 2012  USA  **Focus:** RCT to compare the efficacy of exercise, spinal manipulation, and Neuro Emotional Technique for the treatment of pregnancy-related low back pain  **Duration:** until end of pregnancy  **Follow-up:** no post-intervention follow-up  **Quality:** medium  **PARTICIPANTS:**  **N:** 57 (all female)  **Age:** 29 to 31 years  **Inclusion:** women with singleton pregnancies and back pain of unknown origin that started during pregnancy; exclusions: health conditions contraindicating exercise | **Intervention type:** chiropractic  **Intervention 1 (n=15):** spinal manipulation (high velocity low amplitude thrust)  **Intervention 2 (n=20):** Neuro Emotional Technique (NET) (mind-body technique that combines desensitisation procedures (such as relaxed breathing and visualisation) with elements of Five Element Chinese medicine (such as the association of emotions with certain organs or meridians) and chiropractic medicine)  **Intervention 3 (n=22):** recommended to exercise according to exercise booklet; practise and demonstrations during study visits  **Dose:** exercise: requested to exercise 5 times per week; other treatments paralleled the prenatal care schedule (once monthly up to 28 weeks gestation, twice monthly until 36 weeks, weekly thereafter)  **Providers:** chiropractor with 15 years experience | **Results**  After the intervention   \| **Outcome** \| **Spinal manipulation**  **(mean, SD)** \| **NET**  **(mean, SD)** \| **Exercise**  **(mean, SD)** \| **p-value** \| \| --- \| --- \| --- \| --- \| --- \| \| Roland-Morris disability questionnaire \| 4.1 (4.3) \| 5.7 (4.7) \| 6.1 (5.9) \| NS \| \| Pain (numeric rating scale) \| 1.9 (1.7) \| 2.4 (1.6) \| 2.4 (1.8) \| NS \|  - At least 50% of participants in each treatment group experienced clinically meaningful improvement in symptoms for the Roland Morris Disability Questionnaire - At least 50% of the exercise and spinal manipulation participants also experienced clinically meaningful improvement in pain   ***Specific adverse effects:*** none; 6% of exercise and spinal manipulation visits produced soreness, 18% of NET study visits produced soreness |
| Pizzolorusso 2011  Italy  **Focus:** Effect of osteopathic manipulation treatment (OMT) on gastrointestinal (GI) function and length of hospital stay (LOS) in preterm infants  **Design:** CCT  **Duration:** 2 weeks  **Follow-up:** 2 months  **Quality:** medium  **PARTICIPANTS:**  **N:** 350 (49% female)  **Age:** 29-37 weeks [gestational age]  **Inclusion:** preterm infants with gestational age between 29 and 37 weeks; exclusions were infants with HIV, drug addicted mother, genetic disorders, congenital abnormalities, cardiovascular abnormalities, neurological disorders, enterocolitis, abdominal obstruction, pre-/post-surgery, atelectasis | **Intervention type:** osteopathy  **Intervention (n=162):** OMT consisting of indirect myofascial sutural spread, balanced membranous/ligamentous tension  **Comparison (n=188):** no OMT  **Dose:** session of 20-30 minutes twice per week  **Providers:** certified osteopaths | \| **Change in outcome** \| **OMT** \| **No OMT** \| **OR**  **(95% CI)** \| \| --- \| --- \| --- \| --- \| \| Average daily occurrence of gut symptoms  ≤0.44 versus  > 0.44 \| 134 (82.7%) versus 28 (17.3%) \| 128 (68.1%) versus 60 (32%) \| 0.45 (0.26, 0.74) \| \| Length of stay  < 28 days versus  ≥ 28 days \| 134 (82.7%) versus 28 (17.3%) \| 133 (70.7%) versus 55 (29.3%) \| 0.22 (0.09, 0.51) \|   **Results**  ***Specific adverse effects:*** not reported |
| **Rehabilitation** |  |  |
| Hunter 2011  UK  **Focus:** RCT of manual therapy effects compared to standard physiotherapy in adults with stroke  **Duration:** 2 weeks  **Follow-up:** 2 weeks  **Quality:** medium  **PARTICIPANTS:**  **N:** 76 (50% female)  **Age:** 72.5 years  **Inclusion:** adults with stroke (infarct or haemorrhage in the anterior cerebral circulation) 8-84 days prior to trial entry; paralysed or paretic upper limb (<61/100 on Motricity Index on arm section); no clinically important upper limb pain or visible upper-limb movement deficits due to causes other than stroke | **Intervention type:** physiotherapy  **Intervention:** 3 doses of manual therapy (joint/soft tissue mobilisation, massage, tactile stimulation, active-assisted movements, soft tissue stretch, and/or compression) for 2 weeks  **Intervention 1 (n=18):** 30 min manual therapy as above  **Intervention 2 (n=19):** 60 min manual therapy as above  **Intervention 3 (n=20):** 120 min manual therapy as above  **Comparison (n=19):** conventional physiotherapy  **Dose:** see above  **Providers:** clinical physiotherapists | **Results**   \| **Change in outcome** \| **Standard physiotherapy** \| **Manual therapy**  **30 min** \| **Manual therapy**  **60 min** \| **Manual therapy**  **120 min** \| **p-value** \| \| --- \| --- \| --- \| --- \| --- \| --- \| \| MI (mean) \| 12.4 \| 10.2 \| 17.0 \| 15.7 \| NS \| \| N (%) With  MI > 1 \| 11 (58%) \| 9 (50%) \| 12 (67%) \| 14 (70%) \| NS \| \| ARAT (mean) \| 6.5 \| 6.8 \| 6.6 \| 9.8 \| NS \| \| N (%) With ARAT increase of >5.7 \| 7 (37%) \| 5 (29%) \| 8 (44%) \| 9 (45%) \| NS \|   ***Specific adverse effects:*** not observed |
| Sleszynski 1993  USA  **Focus:** RCT of manual therapy effects compared to incentive spirometry in cholecystectomy adults  **Duration:** Not reported  **Follow-up:** 1 year  **Quality:** medium  **PARTICIPANTS:**  **N:** 42 (81% female)  **Age:** 46 years  **Inclusion:** cholecystectomy adults; participants with any incision other than subcostal or presence of structural deformity was excluded | **Intervention type:** osteopathy  **Intervention (n=21**): thoracic lymphatic pump (TLP) – manual therapy  **Comparison (n=21):** incentive spirometry (IS)  **Dose:** 3 times daily sessions until discharge  **Providers:** osteopaths, students | **Results**   \| **Change in outcome** \| **Thoracic lymphatic pump** \| **Incentive spirometry** \| **p-value** \| \| --- \| --- \| --- \| --- \| \| N (%) with atelectasis \| 2/21 (5%) \| 2/21 (5%) \| NS \| \| FVC \| 0.28 SD0.18 \| 0.39 SD0.10 \| <0.05 \| \| FEV \| 0.29 SD0.19 \| 0.40 SD0.10 \| <0.05 \|   ***Specific adverse effects:*** not observed (other than atelectasis) |
| Goldstein 2005  USA  **Focus:** RCT of manual therapy effects compared to morphine in post-abdominal hysterectomy in women  **Duration:** each session of 10 minutes (OMT), 6 minutes (morphine injection)  **Follow-up:** 48 hours  **Quality:** low  **PARTICIPANTS:**  **N:** 39 (100% female)  **Age:** Not reported  **Inclusion:** adults (age > 18 years) after abdominal hysterectomy hospitalised for at least 48 hours, naïve to manipulation therapy, able to self-report pain levels; exclusions were participants with liver/kidney disease, use of antidepressants | **Intervention type:** osteopathy  **Intervention:** osteopathic manipulation therapy (OMT) administered on patient’s both sides in 3 sessions (sacral myofascial release, gentle thoracic and lumbar myofascial soft tissue techniques); morphine – 10 mg in 1 mL  **Intervention 1 (n=10):** pre-operative morphine + post-operative OMT; see above  **Intervention 2 (n=10):** pre-operative morphine + post-operative placebo (OMT); see above  **Intervention 3 (n=10):** pre-operative placebo (morphine) + post-operative OMT; see above  **Comparison (n=9):** pre-operative placebo (morphine) + post-operative placebo (OMT)  **Dose:** see above  **Providers:** Not reported | **Results**   \| **Change in outcome** \| **Morphine + OMT**  **(95% CI)** \| **Morphine**  **+ placebo**  **(95% CI)** \| **Placebo**  **+ OMT**  **(95% CI)** \| **Placebo**  **+ placebo**  **(95% CI)** \| **p-value** \| \| --- \| --- \| --- \| --- \| --- \| --- \| \| Pain score  (0 – 10) \| NR \| NR \| NR \| NR \| >0.05 \| \| Nausea score (0 – 3) \| NR \| NR \| NR \| NR \| >0.05 \| \| Vomiting score (0 – 3) \| NR \| NR \| NR \| NR \| >0.05 \| \| 24 hour post-operative mean dose of morphine \| 0.17  (0.06, 0.28) \| 0.51  (0.26, 0.77) \| 0.36  (0.11, 0.61) \| 0.43  (0.17, 0.70) \| Int 1 versus Int 2 (p=0.02) \| \| 48 hour post-operative mean dose of morphine \| 0.42  (0.16, 0.68) \| 1.14  (0.72, 1.55) \| 0.72  (0.10, 1.34) \| 0.98  (-0.18, 2.13) \| Int 1 versus Int 2 (p=0.01) \|   ***Specific adverse effects:*** not reported |
| Crow 2009  USA  **Focus:** effect of osteopathic manipulation treatment (OMT) on length of hospital stay in patients with ileus after abdominal surgery  **Design:** retrospective chart review  **Duration:** not reported  **Follow-up:** not reported  **Quality:** low  **PARTICIPANTS:**  **N:** 331 (52% female)  **Age:** not reported  **Inclusion:** ileus post abdominal surgery; multiple surgeries were excluded | **Intervention type:** osteopathy  **Intervention:** OMT  **Comparison:** no OMT  **Dose:** not reported  **Providers:** osteopathic medical students, family practice residents  **Further information available on:** ethnicity | \| **Outcome** \| **Osteopathic manipulation treatment (95% CI)** \| **No osteopathic manipulation treatment (95% CI)** \| **p-value** \| \| --- \| --- \| --- \| --- \| \| Length of hospital stay (days) \| 11.8  (10.2, 13.4) \| 14.6  (12.7, 16.4) \| difference: 2.7 days, p=0.029 \|   **Results**  ***Specific adverse effects:*** not reported |
| Yurvati 2005  USA  **Focus:** Effect of osteopathic manipulation treatment (OMT) on cardiac haemodynamics after coronary artery bypass graft (CABG) surgery  **Design:** CCT  **Duration:** 25-30 minutes of session (OMT)  **Follow-up:** 5-10 minutes after OMT (OMT group) versus 2 hours post-surgery (control group)  **Quality:** low  **PARTICIPANTS:**  **N:** 29 (27.6% female)  **Age:** 56-79 years (range)  **Inclusion:** post-CABG surgery adults | **Intervention type:** osteopathy  **Intervention:** OMT consisting of balanced ligamentous tension, indirect myofascial release of the sternum, indirect release of the respiratory diaphragm, occipito-atlantal decompression, rib raising, Sibson’s fascial release  **Comparison:** no OMT  **Dose:** 25-30 minutes of session (OMT)  **Providers:** osteopathic physicians | \| **Change in outcome** \| **OMT (95% CI)** \| **No OMT (95% CI)** \| **p-value** \| \| --- \| --- \| --- \| --- \| \| Mixed venous oxygen saturation (%) \| 3.7%  (2.69, 4.7)1 \| –3.28%  (-4.88, -1.68) \| ≤0.005 (in favour of OMT) \| \| Cardiac index (mean) \| 0.51  (0.38, 0.64) \| 0.14  (0.06, 0.22) \| ≤0.02 (in favour of OMT) \|   **Results**  ***Specific adverse effects:*** not reported |
| Jarski 2000  USA  **Focus:** Effect of osteopathic manipulation treatment (OMT) on pain perception, length of hospital stay, independent negotiation of stairs, and distance ambulated in adults post-knee/hip arthroplasty surgery  **Design:** CCT  **Duration:** 4 days (OMT)  **Follow-up:** 5 days post-surgery  **Quality:** medium  **PARTICIPANTS:**  **N:** 76 (60% female)  **Age:** 66-71 years (mean range)  **Inclusion:** adults post-knee/hip arthroplasty surgery, use of English, mental orientation to follow instructions and questionnaire items | **Intervention type:** osteopathy  **Intervention:** OMT consisting of high velocity low amplitude, muscle energy, myofascial, lymphatic pump, counterstrain, and traction techniques  **Comparison:** no OMT  **Dose:** 5-15 minute sessions of OMT for 4 days  **Providers:** osteopathic family practice residents | \| **Change in outcome** \| **Osteopathic manipulation treatment** \| **No Osteopathic manipulation treatment** \| **p-value** \| \| --- \| --- \| --- \| --- \| \| Time to negotiate stairs (days) \| 4.3 SD1.2 \| 5.4 SD1.6 \| 0.006 \| \| Distance ambulated (m) \| 24.3 SD18.3 \| 13.9 SD14.4 \| NS \| \| Need for supplemental intramuscular analgesics  N (%) \| 14/38 (37%) \| 19/38 (50%) \| NS \| \| Length of hospital stay (days) \| 5.9 SD1.5 \| 6.1 SD2.2 \| NS \| \| Pain perception after OMT  N (%) \| Decreased 15/23 (65%)  No change  8/23 (35%)  Increased  0/23 (0%) \| NA \| NA \|   **Results**  ***Specific adverse effects:*** not reported |
| **Systemic sclerosis** |  |  |
| Maddali Bongi 2009a  Italy  **Focus:** effect of a rehabilitation programme for systemic sclerosis patients  **Duration:** 9 weeks  **Follow-up:** 18 weeks (9 weeks post-intervention)  **Quality:** low/moderate  **PARTICIPANTS:**  **N:** 20 (65% female)  **Age:** 57.1 SD15.0 years  **Inclusion:** systemic sclerosis; 10 had lung involvement, none had arthritis or myositis; all had flexion contractures, 7 had hand oedema, 7 had fingertip ulcers | **Intervention type:** physiotherapy  **Intervention (n=10):** 1. *Hand involvement* treated with a combination of *connective tissue massage* and *McMennell joint manipulation* (1 hour/session, twice a week). Patients with oedematous hands were also treated with supplementary sessions of *manual lymphatic drainage* (1 hour/session, twice a week). 2. For *face involvement* a combination of *Kabat’s* method, *connective tissue massage* and *kinesitherapy* was used (1 hour/session, twice a week). 3. The global rehabilitation programmes include *Hydrokinesytherapy,* performed by patients without ulcers. The patients with ulcers (n= 3) were assigned to a *land-based rehabilitation.* In both cases, patients performed *respiratory rehabilitation* exercises (1 hour/session, once a week). [detailed procedures described]  **Comparison (n=10):** Patients of the observational group (controls) were followed up and recommended not to start any new physical or pharmacological therapy during the study period.  **All:** educational recommendation on general measures (nutrition, skin warming and skin and mucosal protection); all patients continued pharmacological treatments without change  **Dose:** see above  **Providers:** not reported | - significance of results seems to refer to change from baseline, not comparison to control group; just reported that the control group did not show any significant improvement in general health condition and hands and face measures - significant improvement in the following parameters both at end of intervention and follow-up: Hand Mobility in Scleroderma Test, mouth opening (cm) - significant improvement in the following parameters only at end of intervention but not at follow-up: Physical Synthetic Index (SF-36), Mental Synthetic Index (SF-36), Health Assessment Questionnaire Disability Index, Duruoz Hand Index, fist closure (cm), FACE VAS - no significant improvement either at end of intervention or at end of follow-up: hand opening (cm) - decrease in oedema in patients with hand oedema (n=4) - overall satisfaction was high   ***Specific adverse effects:*** not reported |
| Maddali Bongi 2009b  Italy  **Focus:** effect of a rehabilitation programme for systemic sclerosis patients  **Duration:** 9 weeks  **Follow-up:** 18 weeks (9 weeks post-intervention)  **Quality:** low/moderate  **PARTICIPANTS:**  **N:** 40 (75% female)  **Age:** 57.8 SD11.8years  **Inclusion:** systemic sclerosis; 16 had lung involvement; none had arthritis or myositis; all had flexion contractures; 18 had fingertip ulcers | **Intervention type:** physiotherapy  **Intervention (n=10):** connective tissue massage and McMennell joint manipulation plus daily home exercises (hand and arm)  **Comparison (n=10):** daily home exercise programme only  **All:** educational recommendation on general measures (nutrition, skin warming and skin and mucosal protection); all patients continued pharmacological treatments without change  **Dose:** manual therapy: two 1 h sessions per week; home exercises: 20 min daily  **Providers:** not reported | - significance of results seems to refer to change from baseline, not comparison to control group; in the exercise only group, only fist closure was improved after the end of the intervention, but not after the end of follow-up - significant improvement in the following parameters both at end of intervention and follow-up: Hand Mobility in Scleroderma Test, Cochin hand functional disability scale, fist closure, Health Assessment Questionnaire Disability Index - significant improvement in the following parameters only at end of intervention but not at follow-up: Mental Synthetic Index (SF-36), Physical Synthetic Index (SF-36), - no significant improvement either at end of intervention or at end of follow-up: hand opening   ***Specific adverse effects:*** not reported |
| **Adverse events** |  |  |
| Boyle 2008  Canada  **Focus:** to determine if at an ecological level, the annual rates of chiropractor utilisation were associated with annual incidence rates of hospitalisations with vertebrobasilar artery (VBA) stroke in two Canadian Provinces  **Design:** cohort study  **Duration:** NA  **Follow-up:** 1993-2004  **Quality:** low  **PARTICIPANTS:**  **N:** NA (ecological study)  **Age:** not reported  **Inclusion:** hospitalised/discharged with VBA stroke | **Intervention:** chiropractic utilisation rate  **Comparison:** different chiropractic utilisation rates  **Dose:** NA  **Providers:** chiropractors | \| Change in outcome \| **Ontario**  **1993-2002** \| **Saskatchewan**  **1993-2002** \| \| --- \| --- \| --- \| \| N of hospitalisations with VBA stroke \| 818 \| 82 \| \| Crude cumulative incidence per 100,000 person-years \| 0.750 \| 0.855 \| \| Males  Females \| 0.964  0.542 \| 1.545  0.559 \| \| Age <=45 years  Age >45 years \| 0.145  1.846 \| 0.098  2.184 \|   **Results**  Saskatchewan  In 2000, there was 360% increase in annual incidence of VBA stroke hospitalisations (up to 1.8 per 100,000 population); during the study period, chiropractic utilisation rates were stable  Ontario  In 2000, there was 38% increase in annual incidence of VBA stroke hospitalisations (up to 1.0 per 100,000 population); during the study period, chiropractic utilisation rates steadily declined  At ecological level, there was no correlation between the chiropractic utilisation rates and annual incidence of VBA stroke.  ***Specific adverse effects:*** VBA stroke |
| Hayes 2006  USA  **Focus:** Effect of osteopathic manipulation treatment (OMT) on in paediatric population (17 years or younger)  **Design:**  cohort study  **Duration:** at least two office visits  **Follow-up:** 1 year  **Quality:** low  **PARTICIPANTS:**  **N:** 346 (50% female)  **Age:** 7.37 years (SD=5.51)  **Inclusion:** paediatric patients 19 years or younger with at least two visits to osteopathic manipulative medicine offices | **Intervention:** OMT consisting of cranial manipulation, myofascial release/soft tissue technique, or both.  **Comparison:** none  **Dose:** at least 2 visits to osteopathic physicians  **Providers:** osteopathic physicians | \| OMT associated aggravation \| **N of patients** \| **Incidence % (95% CI)** \| \| --- \| --- \| --- \| \| Worsening symptoms \| 7 \| 2.0 (0.8, 4.1) \| \| Behaviour problems \| 5 \| 1.4 (0.5, 3.3) \| \| Irritability \| 5 \| 1.4 (0.5, 3.3) \| \| Pain \| 4 \| 1.2 (0.3, 2.9) \| \| Soreness \| 4 \| 1.2 (0.3, 2.9) \| \| Headache \| 2 \| 0.6 (0.1, 2.1) \| \| Dizziness \| 1 \| 0.3 (0.0, 1.6) \| \| Flu-like symptoms \| 1 \| 0.3 (0.0, 1.6) \| \| Treatment reaction \| 1 \| 0.3 (0.0, 1.6) \| \| Tiredness \| 1 \| 0.3 (0.0, 1.6) \|   **Results**  ***Specific adverse effects:*** no documented treatment-associated complications (cerebrovascular accidents, dislocation, fracture, pneumothorax, sprains/strains, or death)  31 patients had treatment-associated aggravations (9.0%, 95% CI: 6.2, 12.5)  The authors’ conclusion: in paediatric patients, the incidence of iatrogenic reactions after osteopathic manipulation is low and this treatment appears to be safe if administered by physicians specialised in osteopathic manipulation |
| Miller 2008  UK  **Focus:** To follow-up and document parental reports of adverse events in children younger than 3 years after receiving chiropractic manual treatment  **Design:**  cohort study  **Duration:** 2 years  **Follow-up:** Not reported  **Quality:** low  **PARTICIPANTS:**  **N:** 697 (41% female)  **Age:** 5-8 weeks (range)  **Inclusion:** paediatric patients younger than 3 years with colic and/or irritability presenting to a chiropractic teaching clinic within the study period | **Intervention:** paediatric spinal manipulative therapy (PSMT) applied to full spine, decompression, pelvis, upper/lower extremity, massage, other  **Comparison:** no comparison  **Dose:** Not reported  **Providers:** osteopathic specialists | **Outcomes:** any adverse events reported by a patient’s parent  **Results**  No parent reported serious adverse event; parents of 7 of 697 (1.0%) children reported an adverse event; the events (increased crying for six children and not feeding well/mild distress for one child) were mild-and transient in nature requiring no medical care  ***Specific adverse effects:*** crying, not feeding well, mild distress |
| Rajendran 2009  UK  **Focus:** To explore the feasibility of conducting a follow-up study and collecting the most often reported adverse events by patients after receiving osteopathic manual treatment (OMT)  **Design:**  cohort study  **Duration:** Not reported  **Follow-up:** 7 days post-treatment  **Quality:** low  **PARTICIPANTS:**  **N:** 60 (57% female)  **Age:** mean: 43.5 (SD: 13.0) years; 19-71 years (range)  **Inclusion:** Adults (> 18 years) with a new complain (pain in lower back, head/neck, upper limb, pelvis/hip buttock, lower limb, upper/mid back, stomach/abdomen, lack of mobility) with no prior manual treatment within the past 6 months | **Intervention:** OMT consisting of high velocity low amplitude thrust manipulation, direct techniques (articulatory, muscle energy, direct soft tissue), indirect techniques (functional, balanced ligament tension, counterstrain), other techniques (cranial visceral manipulation, Chapman’s reflexes, lymph-pump technique)  **Comparison:** no comparison  **Dose:** Not reported (treatment delivery according to normal clinic procedures)  **Providers:** 4^th^ year osteopathic students | **Outcomes:** any adverse events (i.e., additional effects of treatment) reported by a patient using a 15-item check-list  **Results**   \| Number of reported adverse events [cumulative] \| **7 days of follow-up** \| \| --- \| --- \| \| Local pain \| 130 \| \| Local stiffness \| 98 \| \| Worsening of complain \| 63 \| \| Radiating pain \| 40 \| \| Unexpected tiredness \| 39 \| \| Pain/discomfort \| 38 \| \| Stiffness/reduced mobility \| 32 \| \| Headaches \| 24 \| \| Fainting/dizziness/vertigo \| 20 \| \| Numbness/tingling (legs/feet) \| 17 \| \| Muscle weakness \| 11 \| \| Vision disturbance \| 8 \| \| Tinnitus \| 7 \| \| Numbness/tingling (arms/hands) \| 5 \| \| Nausea/vomiting \| 3 \| \| Total \| 535 \| |
| Choi 2011  Canada  **Focus:** To describe demographic characteristics, health care utilisation, and co-morbidities of VBA stroke cases  **Design:**  case series  **PARTICIPANTS:**  **N:** 93 (49.5% female)  **Age:** mean: 57.6 (SD: 16.1) years  **Inclusion:** patients hospitalised (between April 1993 and March 2002) for VBA stroke, who had consulted a chiropractor within the year before their stroke | **Intervention:** chiropractic care within the year before stroke | **Outcomes:** VBA stroke  **Results**  About 96% of the VBA stroke cases had consultations with a primary care physician and 75.3% had one or more co-morbidities   \| Co-morbidities \| **VBA cases (n=93)** \| \| --- \| --- \| \| Neck pain and headaches \| 62 (66.7%) \| \| Circulatory system diseases \| 59 (63.4%) \| \| Nervous system diseases \| 44 (47.3%) \| \| Musculoskeletal system and connective tissue diseases \| 41 (44.1%) \| \| Respiratory system diseases \| 36 (38.7%) \| \| Hypertension \| 34 (36.6%) \| \| Accidents, violence, poisoning \| 33 (35.5%) \| \| Heart disease \| 28 (30.1%) \| \| Digestive system disease \| 28 (30.1%) \| \| Upper respiratory tract infections \| 28 (30.1%) \| \| Endocrine, nutritional metabolic diseases \| 26 (28.0%) \| \| Skin diseases \| 25 (26.9%) \| \| Genitourinary system diseases \| 23 (24.7%) \| \| Mental disorders \| 18 (19.4%) \| \| Diabetes \| 15 (160.1%) \| \| Cerebrovascular disease \| 14 (15.1%) \| \| Neoplasms \| 12 (12.9%) \| |
| Sweeney 2010  Ireland  **Focus:** to document the use of manual therapy (i.e., manipulation and mobilisation) by the chartered physiotherapists in Ireland and describe adverse events associated with the use of these techniques  **Design:** survey  **PARTICIPANTS:**  **N:** 127 physiotherapists responders  **Age:** mean: 33.3 (SD: 7.05) years  **Mean number of years of experience:** 13.81 years (SD 7.23)  **Education:** 40 (32%) had no post-graduate qualification in manual therapy, 23 (18%) had Master’s degree in manual therapy, 14 (11%) had a higher Diploma in Manipulative Therapy, 13 (10%) had a general Master’s degree, and 37 (29%) had attended a variety of short courses (e.g., Cyriax, McKenzie, Kaltenborn, Mulligan, myofascial techniques, muscle energy, etc…)  **Inclusion:** practicing current members of the chartered physiotherapists in Ireland | **Survey:** 44-item self-administered postal survey containing 4 sections (demographic data, use of HVTT/non-HVTT techniques, the occurrence of adverse events); reminders sent to non-responders 4 weeks after the initial survey | **Results**  **Response rate:** 127/259 (49%);  **Intervention:** All 127 (100%) responders used non-High Velocity Thrust Techniques (non-HVTT) and 34 (27%) used High Velocity Thrust Techniques (HVTT)  **Vertebrobasilar Insufficiency (VBI) assessment:** 18 (53%) of the responders administering HVTT and 44 (40%) of those administering non-HVTT techniques  **Adverse events:** 33/127 (26%) reported an adverse event. For HVTT technique, 5/34 (15%) reported an adverse event (mostly of mild nature); for non-HVTT technique, 26/127 (20%) reported an adverse event (mostly mild but three serious adverse events such as drop attack, fainting, transient ischemic attack); for cervical traction, 2/99 (2%) reported an adverse event   \| Technique \| **N of responders** \| **Adverse event** \| \| --- \| --- \| --- \| \| HVTT \| 1 (3%) \| Headache \| \| 2 (6%) \| No details \| \| 1 (3%) \| Dizziness/soreness of cervical muscle \| \| 1 (3%) \| Dizziness \| \| Non-HVTT \| 10 (30%) \| Transient dizziness, nausea, symptoms \| \| 6 (18%) \| No details \| \| 1 (3%) \| Drop attack \| \| 1 (3%) \| Fainting \| \| 1 (3%) \| Transient ischemic attack \| \| 7 (20%) \| Paresthesia, whiplash, dizziness, blurred vision, nausea, irritability, upper limb/neck pain increase, disorientation, sensory loss \| \| Cervical traction \| 1 (3%) \| Speaking gibberish \| \| 1 (3%) \| Awake but non-responsive/talk with difficulty \| |
| Alcantara 2009  USA  **Focus:** to document the use and evaluate the safety of paediatric chiropractic through surveying chiropractors and parents of paediatric patients  **Design:** survey  **PARTICIPANTS:**  **Chiropractors**  **N:** 21 responders  **Age:** not reported  **Mean number of years of experience:** not reported  **Education:** not reported  **Inclusion:** Chiropractor in good standing with the Board of Chiropractor Examiners, agree to terms of participation in the survey, maintaining patient confidentiality  **Parents of paediatric patients**  **N:** 239 responders  **Age:** see Results in Table  **Mean number of years of experience:** NA  **Education:** see Results in Table  **Inclusion:** parents of paediatric patients (aged 18 years or younger) who received chiropractic care (1-12 visits) | **Chiropractor survey:** The survey sent to chiropractors included information on patient demographic data (e.g., age, gender, number of visits), presenting complaints, chiropractic technique/spinal regions used for patient care, treatment-associated aggravations (defined as worsening of symptoms or complaints following treatment), and treatment-associated complications (defined as cerebrovascular accidents, dislocation, fracture, pneumothorax, sprains/strains, or death as a result of treatment)  **Parent survey:** The parent survey included information on parents’/guardians’ gender, age, level of education as well as treatment-associated aggravations, and treatment-associated complications | **Results**  **Chiropractor survey**  Response rate: 21 chiropractor responders provided data on 577 paediatric patients  Demographics of patients: mean age 7.45 years; 273 females and 304 males, mean number of office visits: 9.4  Presentation of patients: wellness care (46%), musculoskeletal complaints (26%), digestion/elimination problems (7%), ear/nose/throat problems (6%), neurological problems (6%), immune dysfunction (5%), and other (4%).  Intervention: The chiropractic techniques used were regional or full spine manipulation using diversified technique, Gonstead technique, Thompson technique, activator methods, cranial techniques, and others  Adverse events:  The chiropractors’ survey revealed three reports of treatment-associated aggravations (based on 5,438 visits) such as ‘muscle stiffness,’ ‘spine soreness through the seventh visit,’ and ‘stiff/sore’. No treatment-associated complications were reported  **Parent survey**  Response rate: 239 parents of paediatric patients provided data on 239 paediatric patients  Demographics of parents: mean age 35.6 years, 222 females and 16 males; PhD (n=7), Master’s degree (n=29), Baccalaureate (n=73), college certification (n=35), some college (n=61), high school graduates (n=26), some high school (n=3), unknown (n=5)  Presentation of patients: wellness care (47%), musculoskeletal complaints (22.6%), ear/nose/throat problems (4.2%), neurological problems (3%), colic (2.5%), immune dysfunction (1.2%), digestion/elimination problems (3.7%), birth trauma (2.9%), and other (10.9%)  Adverse events:  The parent survey revealed two reports of treatment-associated aggravations (soreness of the knee and stiffness of the cervical spine). There was no report of treatment-associated complications |
