# Supplementary material for: Clinical effectiveness of manual therapy for the management of musculoskeletal and non-musculoskeletal conditions: systematic review and update of UK evidence report
Source: Chiropr Man Therap. 2014 Mar 28;22:12. doi: 10.1186/2045-709X-22-12 (PMC3997823; doi:10.1186/2045-709X-22-12)
Supplement: Additional file 4 — Quality assessments of all included papers. [file 2045-709X-22-12-S4.docx]

# Additional file 4. Quality assessment of all included papers

## Table A. Quality assessment of included systematic reviews

| **Study** | **Was an ‘a priori’ design provided?** | **Was there duplicate study selection and data extraction?** | **Was a comprehensive literature search performed?** | **Was the status of publication (i.e. grey literature) used as an inclusion criterion?** | **Was a list of studies (included and excluded) provided?** | **Were the characteristics of the included studies provided?** | **Was the scientific quality of the included studies assessed and documented?** | **Was the scientific quality of the included studies used appropriately in formulating conclusions?** | **Were the methods used to combine the findings of studies appropriate?** | **Was the likelihood of publication bias assessed?** | **Was the conflict of interest stated?** | **Overall** |
| --- | --- | --- | --- | --- | --- | --- | --- | --- | --- | --- | --- | --- |
| **Musculoskeletal** |  |  |  |  |  |  |  |  |  |  |  |  |
| *Mid-back pain* |  |  |  |  |  |  |  |  |  |  |  |  |
| Vanti 2008 | + | ? | + | – | – | + | ? | ? | + | – | – | 4/11 |
| *Ankle and foot conditions* |  |  |  |  |  |  |  |  |  |  |  |  |
| Brantingham 2012 | + | +/– | + | – | + | + | + | + | + | – | + | 8.5/11 |
| Lin 2012 | + | + | + | – | + | + | + | + | + | – | + | 9/11 |
| *Carpal tunnel syndrome* |  |  |  |  |  |  |  |  |  |  |  |  |
| Ellis 2008 | + | +/– | + | ? | +/– | + | + | + | + | – | – | 7/11 |
| Huisstede 2010 | + | + | + | ? | +/– | + | + | + | + | – | + | 8.5/11 |
| Hunt 2009 | + | + | + | – | +/– | + | + | + | + | – | + | 8.5/11 |
| Muller 2004 | + | +/– | + | + | + | + | + | + | + | – | +/– | 9/11 |
| *Lateral epicondylitis* |  |  |  |  |  |  |  |  |  |  |  |  |
| Herd 2008 | + | ? | + | – | + | + | + | + | + | – | – | 7/11 |
| Kohia 2008 | – | ? | – | – | + | + | + | + | + | – | – | 5/11 |
| Nimgade 2005 | + | ? | + | – | + | + | + | + | + | – | – | 7/11 |
| Trudel 2004 | + | ? | – | – | + | + | + | + | + | – | + | 7/11 |
| *Shoulder conditions* |  |  |  |  |  |  |  |  |  |  |  |  |
| Brantingham 2011 | + | +/– | + | ? | +/– | + | + | + | + | – | + | 8/11 |
| Braun 2009 | + | ? | + | ? | + | + | + | + | + | – | + | 8/11 |
| Camarinos 2009 | + | +/– | + | ? | +/– | + | + | + | + | – | – | 7/11 |
| Pribicevic 2010 | + | ? | + | ? | +/– | + | + | + | + | – | + | 7.5/11 |
| **Headache** |  |  |  |  |  |  |  |  |  |  |  |  |
| *Cervicogenic headache* |  |  |  |  |  |  |  |  |  |  |  |  |
| Posadzki 2011 | + | + | + | – | + | + | + | + | + | – | + | 9/11 |
| *Miscellaneous headache* |  |  |  |  |  |  |  |  |  |  |  |  |
| Bryans 2011 | + | + | + | – | + | + | + | + | + | – | + | 9/11 |
| *Fibromyalgia* |  |  |  |  |  |  |  |  |  |  |  |  |
| Terhorst 2011 | + | +/– | + | – | +/– | + | + | + | + | – | + | 8/11 |
| *Myofascial pain syndrome* |  |  |  |  |  |  |  |  |  |  |  |  |
| de las Peñas 2005 | + | +/– | + | ? | +/– | + | + | + | + | – | – | 7/11 |
| Richards 2006 | + | – | + | ? | +/– | + | + | + | + | – | – | 6.5/11 |
| **Non-musculoskeletal** |  |  |  |  |  |  |  |  |  |  |  |  |
| *Asthma* |  |  |  |  |  |  |  |  |  |  |  |  |
| Kaminskyi 2010 | + | + | + | – | +/– | + | + | + | + | – | – | 7.5/11 |
| *ADHD / learning disabilities* |  |  |  |  |  |  |  |  |  |  |  |  |
| Karpouzis 2010 | + | +/– | + | – | + | + | + | + | + | – | + | 8.5/11 |
| *Cancer care* |  |  |  |  |  |  |  |  |  |  |  |  |
| Alcantara 2011 | + | +/– | + | ? | – | +/– | – | – | +/– | – | – | 3.5/11 |
| *Cervicogenic dizziness* |  |  |  |  |  |  |  |  |  |  |  |  |
| Lystad 2011 | + | + | + | ? | + | + | + | + | + | – | + | 9/11 |
| *Chronic fatigue / fibromyalgia* |  |  |  |  |  |  |  |  |  |  |  |  |
| Porter 2010 | + | + | + | – | + | + | + | + | + | – | + | 9/11 |
| *Chronic pelvic pain* |  |  |  |  |  |  |  |  |  |  |  |  |
| Franke 2013 | + | + | + | – | + | + | + | + | + | – | + | 9/11 |
| Loving 2012 | + | + | + | – | +/– | + | + | + | + | – | + | 8.5/11 |
| *Infantile colic* |  |  |  |  |  |  |  |  |  |  |  |  |
| Dobson 2012 | + | + | + | – | + | + | + | + | + | + | + | 10/11 |
| *Paediatric nocturnal enuresis* |  |  |  |  |  |  |  |  |  |  |  |  |
| Huang 2011 | + | + | + | ? | + | + | + | + | + | – | + | 9/11 |
| *Pneumonia* |  |  |  |  |  |  |  |  |  |  |  |  |
| Heneghan 2012 | + | +/– | + | – | +/– | + | + | + | + | + | ? | 8/11 |
| Yang 2010/2013 | + | + | + | – | + | + | + | + | + | +/– | + | 9.5/11 |
| *Infantile colic* |  |  |  |  |  |  |  |  |  |  |  |  |
| Alcantara 2011 (colic) | + | +/– | + | – | – | +/– | – | – | +/– | – | – | 3.5/11 |
| Perry 2011 | + | +/– | + | ? | +/– | + | + | + | + | – | + | 8/11 |
| *Gastrointestinal disorders* |  |  |  |  |  |  |  |  |  |  |  |  |
| Ernst 2011 | + | +/– | + | – | – | +/– | + | + | +/– | – | + | 6.5/11 |
| *Hypertension* |  |  |  |  |  |  |  |  |  |  |  |  |
| Mangum 2012 | + | +/– | + | ? | +/– | +/– | + | + | + | – | + | 7.5/11 |
| *Insomnia* |  |  |  |  |  |  |  |  |  |  |  |  |
| Kingston (2010) | + | +/– | ? | ? | – | – | – | ? | ? | – | + | 2.5/11 |
| *Otitis media* |  |  |  |  |  |  |  |  |  |  |  |  |
| Gleberzon 2012 | + | +/– | + | + | + | + | + | + | + | – | – | 8.5/11 |
| Pohlmann 2012 | + | + | + | ? | + | + | + | + | + | – | + | 9/11 |
| *Pregnancy / obstetric care / neonatal care* |  |  |  |  |  |  |  |  |  |  |  |  |
| Khorsan 2009 | + | ? | + | – | + | + | + | + | + | – | – | 7/11 |
| *Adverse events* |  |  |  |  |  |  |  |  |  |  |  |  |
| Carlesso 2010 | + | + | + | + | – | + | + | + | + | – | – | 8/11 |
| Carnes 2009  Carnes 2010 | + | + | + | – | + | + | + | + | + | – | + | 9/11 |
| Gouveia 2009 | + | + | – | – | + | + | – | – | + | – | + | 6/11 |
| Haldeman 1999 | + | ? | + | ? | + | + | – | – | ? | – | – | 4/11 |
| Miley 2008 | + | ? | + | ? | – | – | – | ? | + | – | + | 4/11 |
| Stevinson 2002 | – | ? | + | ? | + | + | – | – | – | – | – | 3/11 |
| Stuber 2012 | + | ? | + | – | + | + | + | + | + | – | + | 8/11 |

+ ‘Yes’; – ‘No’; +/– Partial ‘Yes’; ? ‘Not clear’; *Rating (by criteria fulfilled, i.e. ‘yes’ response):* 9 to 11 high quality, 5 to 8 medium quality, 0 to 4 low quality.

## Table B. Quality assessment of included randomised controlled trials

| **Study** | **Adequate sequence generation** | **Adequate allocation concealment** | **Blinding (especially outcome assessment)** | **Incomplete outcome data addressed** | **Free of selective reporting** | **Free of other bias (e.g. similarity at baseline, power assessment, conflict of interest)** | **Overall** |
| --- | --- | --- | --- | --- | --- | --- | --- |
| **Musculoskeletal** |  |  |  |  |  |  |  |
| *Sciatica* |  |  |  |  |  |  |  |
| McMorland 2010 | + | ? | ? | + | + | + | 4/6 |
| Paatelma 2008 | + | + | ? | + | + | + | 5/6 |
| *Neck pain* |  |  |  |  |  |  |  |
| Aquino 2009 | + | ? | + | + | + | – | 4/6 |
| Gemmell 2010 | + | + | – | + | + | – | 4/6 |
| Klein 2013 | + | + | + | + | + | + / – | 5/6 |
| Leaver 2010 | + | + | ? | + | + | + | 5/6 |
| Martel 2011 | ? | + | – | + | + | + | 4/6 |
| Puentedura 2011 | ? | + | + | + | + | – | 4/6 |
| Schomacher 2009 | ? | ? | ? | + | + | ? | 2/6 |
| *Ankle and foot disorders* |  |  |  |  |  |  |  |
| Kuhar 2007 | – | – | ? | ? | ? | ? | 0/6 |
| Joseph 2010 | + | ? | ? | ? | + | + / – | 2.5/6 |
| du Plessis 2011 |  |  |  |  |  |  |  |
| Renan-Ordine 2011 | + | ? | + / – | – | + | + | 3.5/6 |
| *Carpal tunnel syndrome* |  |  |  |  |  |  |  |
| Hains 2010 | + | + | – | + | + | + / – | 4.5/6 |
| *Lateral epicondylitis* |  |  |  |  |  |  |  |
| Ajimsha 2012 | ? | ? | + / – | ? | + | ? | 1.5/6 |
| Blanchette 2011 | ? | – | – | + | + | ? | 2/6 |
| Nagrale 2009 | ? | ? | + | + | + | ? | 3/6 |
| Viswas 2012 | ? | ? | ? | + | + | ? | 2/6 |
| *Shoulder disorders* |  |  |  |  |  |  |  |
| Bialoszewski 2011 | ? | ? | ? | – | + | + / – | 1.5/6 |
| Bron 2011 | + | + | + / – | + | + | + | 5.5/6 |
| Fink 2012 | + | + / – | + / – | ? | + | + | 4/6 |
| Yang 2012 | + | + | + / – | + | + | + / – | 5/6 |
| *Temporomandibular disorders* |  |  |  |  |  |  |  |
| Craane 2012 | + | + | + | + / – | + | + / – | 5/6 |
| Cuccia 2010 | ? | ? | ? | ? | + | – | 1/6 |
| Guarda-Nardini 2012 | ? | ? | ? | ? | + | + / – | 1.5/6 |
| Kalamir 2010 | + | + | + | + | + | ? | 5/6 |
| Yoshida 2005 | ? | ? | ? | ? | + | ? | 1/6 |
| **Headache and other** |  |  |  |  |  |  |  |
| *Cervicogenic headache* |  |  |  |  |  |  |  |
| von Piekartz 2011 | + | – | + | + | + | + | 5/6 |
| Youssef 2013 | + | + | ? | ? | + | + / – | 3.5/6 |
| *Tension-type headache* |  |  |  |  |  |  |  |
| Ajimsha 2011 | ? | ? | + / – |  |  |  |  |
| Anderson 2006 | + | ? | + | + | + | ? | 4/6 |
| Castien 2011  Castien 2009 | ? | + | + | + | + | – | 4/6 |
| van Ettekoven 2006 | + | + | + | + | – | + | 5/6 |
| Vernon 2009 | ? | + | + | – | + | – | 3/6 |
| *Miscellaneous headache* |  |  |  |  |  |  |  |
| de Hertogh 2009 | ? | + | + | + | + | – | 4/6 |
| Foster 2004 | ? | ? | – | + | + | + | 3/6 |
| *Fibromyalgia* |  |  |  |  |  |  |  |
| Castro-Sanchez 2011a (Clin Rehab) | + | ? | + / – | ? | + | + / – | 3/6 |
| Castro-Sanchez 2011b (EB CAM) | ? | ? | + / – | – | + | + / – | 2/6 |
| *Myofascial pain syndrome* |  |  |  |  |  |  |  |
| Gemmell 2008a | + | – | + / – | + | + | + | 4.5/6 |
| Gemmell 2008b | + | ? | + / – | + | + | + | 4.5/6 |
| Nagrale 2010 | + | ? | + / – | + | + | + / – | 4/6 |
| Sarrafzadeh 2012 | ? | ? | ? | ? | + | + / – | 1.5/6 |
| **Non-musculoskeletal** |  |  |  |  |  |  |  |
| *Asthma* |  |  |  |  |  |  |  |
| Mehl-Madrona 2007 | + | + | + / – | – | + / – | + / – | 3.5/6 |
| *ADHD / learning disabilities* |  |  |  |  |  |  |  |
| Bierent-Vass 2005 | ? | ? | ? | ? | ? | ? | 0/6 |
| Hubmann 2006 | ? | ? | ? | ? | ? | ? | 0/6 |
| *Cancer care* |  |  |  |  |  |  |  |
| Cantarero-Villanueva 2011 | + | + | + | + | + | + | 6/6 |
| Fernández-Lopez 2012 | – | – | + / – | ? | + | + / – | 2/6 |
| López-Sendin 2012 | + | ? | + / – | + | + | + | 4.5/6 |
| Pace do Amaral 2012 | + | ? | + / – | ? | + | + | 3.5/6 |
| *Cerebral palsy* |  |  |  |  |  |  |  |
| Duncan 2004 | ? | ? | ? | – | + | ? | 1/6 |
| Duncan 2008 | + / – | + | + / – | + | + | + / – | 4.5/6 |
| Wyatt 2011 | + | ? | + / – | ? | + | + | 3.5/6 |
| *Cervicogenic dizziness / balance* |  |  |  |  |  |  |  |
| Hawk 2009 | ? | ? | + / – | + / – | + | + / – | 2.5/6 |
| *Chronic pelvic pain* |  |  |  |  |  |  |  |
| FitzGerald 2009 | + | ? | + / – | + | + | + | 4.5/6 |
| Heyman 2006 | ? | ? | – | ? | + | + | 2/6 |
| Marx 2009 | + | ? | ? | – | + | + / – | 2.5/6 |
| *Cystic fibrosis* |  |  |  |  |  |  |  |
| Sandsund 2011 | + | ? | + / – | + | + | ? | 3.5/6 |
| *Dysfunctional voiding* |  |  |  |  |  |  |  |
| Nemett 2008 | – | – | – | – | + | ? | 1/6 |
| *Menopausal symptoms* |  |  |  |  |  |  |  |
| Cleary 1994 | – | ? | + / – | ? | + | ? | 1.5/6 |
| *Gastrointestinal disorders* |  |  |  |  |  |  |  |
| Florance 2012 | ? | ? | + / – | + / – | + | ? | 2/6 |
| Hundscheid 2006 | – | – | – | + | + | ? | 2/6 |
| *Parkinson’s disease* |  |  |  |  |  |  |  |
| Wells 1999 | ? | ? | + | NA | ? | ? | 1/6 |
| *Respiratory* |  |  |  |  |  |  |  |
| Noll 2006 | ? | ? | + | + | + | ? | 3/6 |
| Zanotti 2012 | + | + | + | + | + | +/– | 5.5/6 |
| *Peripheral vascular disease* |  |  |  |  |  |  |  |
| Ramos-Gonzáles 2012 | + | + | + / – | ? | + | +/– | 4/6 |
| *Pregnancy / obstetric care / neonatal care* |  |  |  |  |  |  |  |
| Goldstein 2005 | – | – | + | ? | + | ? | 2/6 |
| Peterson 2012 | + | + | – | + | + | ? | 4/6 |
| *Rehabilitation* |  |  |  |  |  |  |  |
| Hunter 2011 | – | + | ? | + | + | – | 3/6 |
| Sleszynski 1993 | – | ? | + | + | + | ? | 3/6 |
| Goldstein 2005 | – | – | + | ? | + | ? | 2/6 |
| *Systemic sclerosis* |  |  |  |  |  |  |  |
| Maddali Bongi 2009 a | + | ? | ? | NA + | + | +/– | 3.5/6 |
| Maddali Bongi 2009 a | + | ? | ? | NA + | + | +/– | 3.5/6 |

+ ‘Yes’; – ‘No’; +/– Partial ‘Yes’; ? ‘Not clear’; *Rating (by criteria fulfilled, i.e. ‘yes’ response):* 5 to 6 high quality, 3 to 4 medium quality, 0 to 2 low quality

## Table C. Quality assessment of included controlled cohort studies

| **Study** | **Sufficient description of the groups and the distribution of prognostic factors?** | **Groups assembled at a similar point in their disease progression?** | **Intervention/treatment reliably ascertained?** | **Groups comparable on all important confounding factors?** | **Adequate adjustment for the effects of these confounding variables?** | **Outcome assessment blind to exposure status?** | **Follow-up long enough for the outcomes to occur?** | **Adequate proportion of the cohort followed up?** | **Drop-out rates and reasons for drop-out similar across intervention and unexposed groups?** | **Overall** |
| --- | --- | --- | --- | --- | --- | --- | --- | --- | --- | --- |
| **Musculoskeletal** |  |  |  |  |  |  |  |  |  |  |
| *Lateral epicondylitis* |  |  |  |  |  |  |  |  |  |  |
| Amro 2010 | + | + | + | ? | – | – | – | ? | ? | 3/9 |
| Cleland 2004 | + | ? | + | – | – | – | + | ? | ? | 3/9 |
| Rompe 2001 | – | ? | + | ? | – | – | + | ? | ? | 2/9 |
| **Non-musculoskeletal** |  |  |  |  |  |  |  |  |  |  |
| *Osteosarcoma* |  |  |  |  |  |  |  |  |  |  |
| Wu 2010 | + | + | + | + | ? | – | + | ? | ? | 5/9 |
| *Hypertension* |  |  |  |  |  |  |  |  |  |  |
| Cerretelli 2011 | + | + | + | + | + | – | + | ? | ? | 6/9 |
| *Peripheral arterial disease* |  |  |  |  |  |  |  |  |  |  |
| Lombardini 2009 | + | + | + | + | – | +/– | + | + | ? | 6.5/9 |
| *Pregnancy / obstetric care / neonatal care* |  |  |  |  |  |  |  |  |  |  |
| Pizzolorusso 2011 | + | + | + | – | ? | ? | – | + | + | 5/9 |
| *Rehabilitation* |  |  |  |  |  |  |  |  |  |  |
| Crow 2009 | – | + | + | ? | – | – | + | ? | ? | 3/9 |
| Yurvati 2005 | + | ? | + | – | – | – | + | ? | ? | 3/9 |
| Jarski 2000 | + | + | + | + | – | ? | – | + | + | 6/9 |
| *Adverse events* |  |  |  |  |  |  |  |  |  |  |
| Boyle 2008 | – | + | – | ? | – | – | + | ? | ? | 2/9 |
| Hayes 2006 | – | – | + | – | – | – | + | + | – | 3/9 |
| Miller 2008 | – | – | + | – | – | – | ? | + | – | 2/9 |
| Rajendran 2009 | – | – | + | – | – | – | – | – | – | 1/9 |

+ ‘Yes’; – ‘No’; +/– Partial ‘Yes’; ? ‘Not clear’; *Rating (by criteria fulfilled, i.e. 'yes' response):* 7 to 9 high quality, 4 to 6 medium quality, 0 to 3 low quality

## Table D. Quality assessment of included qualitative studies

| **Study** | **Clear statement of the aims of the research?** | **Qualitative methodology appropriate?** | **Research design appropriate to address the aims of the research?** | **Recruitment strategy appropriate to the aims of the research?** | **Data collected in a way that addressed the research issue?** | **Relationship between researcher and participants adequately considered?** | **Ethical issues taken into consideration?** | **Data analysis sufficiently rigorous?** | **Clear statement of findings?** | **Contributions and implications of the research discussed?** | **Overall** |
| --- | --- | --- | --- | --- | --- | --- | --- | --- | --- | --- | --- |
| **Non-musculoskeletal** |  |  |  |  |  |  |  |  |  |  |  |
| *Asthma* |  |  |  |  |  |  |  |  |  |  |  |
| Shaw 2006 | + | + | + | + | + | ? | ? | + | + | + | 8/10 |

+ ‘Yes’; – ‘No’; +/– Partial ‘Yes’; ? ‘Not clear’; *Rating (by criteria fulfilled, i.e. 'yes' response):* 8 to 10 high quality, 5 to 7 medium quality, 0 to 4 low quality
